# Prevalence of fish parasites from Lake Hawassa, Ethiopia

**DOI:** 10.1371/journal.pone.0357816

**Published:** 2026-09-11

**Authors:** Debela Abdeta Efa, Megersa Akasa, Yobsan Tamiru

**Affiliations:** 1 College of Veterinary Medicine and Agriculture, Addis Ababa University, Bishoftu, Ethiopia; 2 Department of Veterinary Bioscience, Melbourne Veterinary School, Melbourne University, Melbourne, Australia; 3 College of Natural Resource and Agriculture, Ambo University, Gudar, Ethiopia; 4 School of Veterinary Medicine, Wollega University, Nekemte, Ethiopia; University of Uyo, NIGERIA

## Abstract

**Background:**

An estimated 200 species of fishes of marine and freshwater fish are known to occur in Ethiopia. Fish parasites are the main cause of the significant threat to the fish biodiversity of Ethiopia’s freshwater islands. Lake Hawassa plays a central role in the local economy of the city which supports the artisanal fishing sector. The parasite of the fishes at lake Hawassa are hitherto poorly known and there are limited reports on the type of parasites across different fish hosts such as size, age, sex, condition factor, or other biological or ecological traits.

**Methods:**

A cross-sectional study was carried out in 2023–2024 to estimate the prevalence of fish parasites and host factors. In the current survey *Oreochromis niloticus* (n = 135*)*, *Clarias gariepinus* (n = 87), and *Labeobarbus intermedius* (n = 43) were sampled. Internal and external organs was inspected for the presence of the parasite affecting their health and wellbeing. Some external parasites were extracted from the fresh water immediately after fish were caught. The randomly selected host was classified based on species, sex, body weight, and the total body length. Parasites are identified morphologically at genera level using standard identification keys and pictorial guide. Analysed data were presented as frequencies, percentages, mean intensity, and mean abundance. Chi-square and P-values were considered for statistical association.

**Results:**

The parasite distribution varies with sex, body weight, and standard length of the studied fish. The overall infection/infestation rate is 88.30% (n = 234). A total of 1748 parasites classified under Nematodes (e.g., Contracaecum), Digenean trematodes (e.g., Clinostomum, Euclinostomum, Diplostomum, encysted metacercariae), Monogeneans (Dactylogyrus), Myxozoans (Myxobolus spp.), Crustacean ectoparasites (Argulus, Dolops), Hirudinea (leeches)was collected from *O. niloticus* (788), *C. gariepinus* (642) and *L. intermedius* (318). The internal parasites recovered from *O. niloticus* were *Clinostomum* (65.16%), *Euclinostomum* (34.07%), *Contracaecum* (26.67%)*,* and *Diplostomum* (24.44%). Higher number of *Myxobolus* species, leeches, *Argulus*, and *Dolops* species was also recorded. *Digenean encysted metacercaria* (68.97%) and *Contracaecum species* (50.57%) are the most common parasite species infecting *C. gariepinus*. *Diplostomum species*, *Dactylogyrus species*, leeches, *Argulus species*, and *Dolops species* pose a serious threat to fish health in lake Hawassa. The mean intensity of the parasites in *L. intermedius*, C. *gariepinus* and *O*. *niloticus was found to be 8.59, 7.92 and 6.79* while mean abundance is 7.40, 7.37 and 5.84 respectively.

**Conclusion:**

This study found that various body parts are contaminated with one or more parasites. Some of those parasites have established zoonosis or cause cross infection to other fishes requiring urgent cooperation in performing pre-stock assessment for optimal production and maintaining the heath of fishery and hence human being.

## Introduction

Fish play a vital role for food security, economic growth and improvement of the livelihood particularly in low- and medium-income countries [1,2]. In Ethiopia, fisheries depend almost entirely on inland water bodies, including lakes, rivers, reservoirs, and dams, which support commercially important fish species [3]. Although Ethiopia has considerable potential to expand its inland fisheries, parasitic infections in wild fish populations remain a major constraint to sustainable fish production [4]. Fish parasites adversely affect growth, reproduction, survival, and marketability, resulting in reduced fish yield, economic losses, and diminished biodiversity in natural aquatic ecosystems. Several helminth parasites, including *Clinostomum*, *Euclinostomum*, *Contracaecum*, *Eustrongylides*, *Ligula intestinalis*, and *Proteocephalus*, have been reported from commercially important fish species such as *Oreochromis niloticus*, *Clarias gariepinus*, *Labeobarbus* spp., and *Cyprinus carpio* in Ethiopian lakes and reservoirs, with particularly high prevalence in Lakes Tana, Hawassa, Hayk, Chamo, and Ziway [5]. Beyond their detrimental effects on fish health and fisheries productivity, several of these parasites have zoonotic importance and may pose public health risks through the consumption of raw or inadequately cooked fish [4,6].

The demand for fish meat is steadily rising due to its high nutritional value, particularly essential fatty acids, which are ideal for human consumption [7–9]. Fish resources are vital to support human well-being and economic development [2]. Fish provides quality protein, various vitamins and minerals essential for human consumption. According to FAO [10] more than 17% of animal protein for global population is obtained from fish [11].

Despite the growing importance of fisheries and aquaculture in Ethiopia, parasitic infections in wild fish populations remain a significant but often overlooked challenge. Fish parasites can reduce growth, survival, and reproductive performance, leading to declines in fish productivity and biodiversity while compromising the sustainability of inland fisheries. In Ethiopia’s major lakes and reservoirs, where wild fish constitute the primary source of fish production, parasitic diseases can negatively affect fish health, increase post-harvest losses, and reduce the economic returns of fishing communities [5,6]. Some fish parasites also possess zoonotic potential, posing public health risks through the consumption of raw or inadequately cooked fish [12].

Lake Hawassa is one of the major lakes in the Ethiopian Rift Valley, situated close to Hawassa town and features an endorheic basin system. The water from the lake is essential to the nearby towns for recreational use, residential water use, farming, and fish harvesting [13]. This lake is one of the most fished water bodies in Ethiopia where *O. niloticus* accounts for 90% of the annual catch [14]. The Nile tilapia (*O. niloticus*), African catfish (*Clarias gariepinus*), and African big barb (*Labeobarbus intermedius* also known as *Barbus intermedius*) are the fish species that have been found in Lake Hawassa thus far. The black lampeye (*Aplocheilichthys antinorii*), the stone lapping minnow (G*ara quadrimaculata*), and the straight fin barb (*Barbus paludinosis*) are the other tiny fish present in the lake [15].

Parasites are ubiquitous organisms that occur on virtually all living hosts, with aquatic vertebrates, particularly fish, harbouring a remarkably high diversity of parasitic species [16–18]. Fish serve as hosts to a wide range of parasites, including trematodes (flukes, e.g., *Opisthorchis* spp.), nematodes (roundworms, e.g., *Anisakis* spp.), cestodes (tapeworms, e.g., *Diphyllobothrium* spp.), protozoa such as *Cryptosporidium* spp., arthropods, crustaceans, and leeches [19–24]. Although many host–parasite relationships exist in a state of ecological equilibrium and represent one of the most common biological interactions in aquatic ecosystems [25,26] parasite burdens can increase under favourable environmental conditions, poor husbandry, or host stress, resulting in significant adverse effects on fish health.

Parasites impair fish through several mechanisms, including mechanical tissue damage, nutrient depletion, blood feeding, obstruction of vital organs, and immune-mediated pathological changes. These effects compromise normal physiological functions, leading to reduced growth rates, poor feed conversion efficiency, impaired reproduction and fecundity, altered behaviour, increased susceptibility to secondary infections, and reduced survival [6]. Heavy parasitic infestations may cause severe disease outbreaks and mass mortalities in both wild and cultured fish populations, thereby reducing fisheries production and causing substantial economic losses. Moreover, several fish parasites are of zoonotic importance and can be transmitted to humans and other animals through the consumption of raw or inadequately cooked infected fish, posing a significant public health concern [23,27].

Studies have indicated that biotic factors such as season, sex and body size could influence the burden of monogenean prevalence and intensity on *Gambusia affinis*. Accordingly, female had significantly higher prevalence and mean intensity than males, longer fishes have higher prevalence and mean intensity. Additionally, females may have compromised immune system specially during reproductive season [28].

Fish is a good host for parasite multiplication that can be zoonotic to humans or other animals that feed fishes [29,30]. Larval stages of some fish-borne parasites, including certain nematodes and trematodes, may be transmitted to humans through the consumption of raw or undercooked fish, posing important zoonotic risks [12,22,23]. However, in low- and middle-income countries like Ethiopia, people known little about fish borne zoonotic disease such as trematodiasis and anisakiasis [12,31]. Yet these parasitic infections are commonly reported from different freshwater body fishes [4] and the cause of significant human infections worldwide [17]. To date, fewer studies have been conducted on fish parasites in Ethiopia, but they did not quantify the prevalence of parasites across different fish species, body weight, fish lengths and anatomical position in relation to parasite acquisition.

Accordingly, the study aimed to estimate the prevalence, intensity, and abundance of parasites in *O. niloticus*, *C. gariepinus*, and *L. intermedius*, while assessing the influence of host-related factors on parasitic infection. Improved understanding of the diversity, prevalence, and distribution of fish parasites in Ethiopia’s wild fish populations will provide valuable evidence for fisheries management, aquatic ecosystem conservation, food safety, and the development of effective disease surveillance and control strategies.

## Materials and methods

### Description of the study area

One of the eight lakes in Ethiopia’s Rift Valley is Lake Hawassa. Situated 275 km south of Addis Ababa in the Sidama region, it borders Hawassa city’s eastern side. Geographically, the lake is located 1680 meters above sea level between 6°33' and 7°33' N and 38°22' and 38°29' E. The lake has an approximate water volume of 1.3 billion cubic meters (45.9 billion feet^3^) and stretches 16 kilometres from northeast to southwest and 8 kilometres from northwest to southeast. The lake’s mean depth is 11 meters, and its maximum depth is 21.6 meters (70.9 feet). The lake’s surface area, which can reach 99.3 Km^2^ during the rainy season, makes up 93.6 Km^2^ of the catchment’s total area of 1455 Km^2^ [32]. According to estimates, Lake Hawassa releases 58 × 106 m^3^ of groundwater annually into nearby basins. The location of the lake and the city is found at https://en-us.topographic-map.com/map-lllbf3/Lake-Hawassa/.

### Study design and sampling technique

A cross-sectional study was conducted from October 2023 to July 2024 on the fish community at Lake Hawassa to identify the prevalence of major internal and external parasites infesting fishes supported by antemortem and postmortem inspection on fish. The simple random sampling where each fish systematically selected (every specified fish from the cage) was used to select fishes for parasites identification. The assessment was conducted over a period of ten months, from October 2023 to July 2024. Throughout the manuscript, the terms “infection” and “infestation” have been used interchangeably in sections that summarise or discuss both internal and external parasites. To improve clarity and maintain consistency, we have used parasitic infections and infestations” when referring collectively to both endoparasites and ectoparasites.

### Study population

The study population in the current study are Nile tilapia (*Oreochromis niloticus*), African catfish (*Clarias gariepinus*), and African big barb (*L. intermedius*) that are commercially important and found in Lake Hawassa thus far.

### Sample size determination

The sample size was determined according to [33] at 95% confidence interval with a 5% marginal error. Accordingly, Zekarias and Yimer [34] reported prevalence of (79%) of fish parasites from Lake Hawassa. We have applied collective sample size for all the fishes sampled using the formula n = z^2^ x p (1-p)/d^2^, where n = sample size, z = z statistic for a level of confidence (z = 1.96 at 95% CI), p = prevalence (p = 0.79), d = precision (if 5%, d = 0.05), the sample size 265 is included in the current study. In this formula collective sample size was used including *O. niloticus* (n = 135), *L. intermedius (*n = 87) and *C. gariepinus* (n = 43).

### Methods of data collection

#### Description of the sampled fish.

During the sampling, we have randomly picked those fishes from different sampling points. The selection of sampling sites was mainly based on accessibility and the feasibility of obtaining the necessary field support. The species, sex, possible anatomical region of the parasite attachment, body weight, and total and standard lengths of individual fish were initially assessed and recorded. The sex of each fish was tentatively assessed externally and confirmed post-dissection by noting the presence of testes or ovaries [35]. Up on arrival at the Hawassa University, the total length was measured by centimetres using measuring tape and the weight measured using digital balance. The size of fish was separately recorded for each fish species and classified into class I, class II, and class II [36]. Classifying fish species based on size and body weight during the parasite studies is crucial because this factor can influence parasite infection patterns, host vulnerability and the overall dynamics of parasite host interaction impacting both fish health and environmental ecosystem [37]. The selected size breakpoints for length and weight classes reflect biologically meaningful transitions in growth and life-history stages. For example, in *C. gariepinus*, the length thresholds of 50 cm and 75 cm correspond approximately to ontogenetic stages associated with sexual maturation and trophic shifts. Individuals below 50 cm are typically juveniles or subadults with developing gonads and a diet dominated by invertebrates and small prey. Fish between 50–75 cm generally represents sexually mature adults, while individuals exceeding 75 cm are fully mature, larger-bodied predators with broader dietary niches and higher trophic positioning. These ontogenetic changes influence exposure risk to parasites through altered feeding behaviour, habitat use, and immune competence. Therefore, the selected size classes reflect biologically relevant developmental stages that may affect parasite acquisition dynamics within this ecosystem, rather than being purely arbitrary statistical categories [38].

### Antemortem and postmortem identification of parasite

A total of 265 commercially important fishes *O. niloticus, L. intermedius* also known as *L. intermedius*, and C. *gariepinus* each accounting 135, 87, and 43 fishes respectively were first examined alive (skin and gill scraping and picking with forceps followed by freshly dissected samples). The randomly selected fish were immediately transported in an icebox to the Parasitology unit of Hawassa University, for parasitological examination. Fish were initially examined alive for external abnormalities immediately upon capture. They were then humanely euthanized at the sampling site and placed in an icebox containing crushed ice for transport to the laboratory. Fish were euthanized using a percussive blow to the head (concussion) immediately followed by brain destruction through pithing to ensure rapid loss of consciousness and death. To further ensure that no central nervous system activity remained, spinal pithing was performed as a final step. These procedures were applied to minimize distress and ensure humane euthanasia during sample collection [39].

Each sampled fish was externally visualized, palpated, and examined for any ectoparasites on the entire fish skin, the fins, and opercular cavity including the gills. The external parasite was harvested to physiological salt solution from fish body and the fresh water [40]. Scraping from fish skin were taken and smeared on to a clean microscope slide or hand lens. The sample was then examined under compound microscope at 10X, 40X and 100 X magnifications. Then postmortem examination was done using appropriate postmortem kits and standard evisceration technique [41,42] and all body cavities was observed for presence of any encysted or free parasite larvae. The gill filaments, stomach, intestine, liver, heart, gall bladder, kidney and gonads were separately taken out and placed on clean petridishes that contain 10millitre of physiological solution made of (0.9% sodium chloride w/v) [30]. The head of *C. gariepinus* was dissected longitudinally, fixed in 70% ethanol first, and the cranial cavity was washed away into a petridish using a water dropper and checked for parasites especially *Diplostomum* species. To clear their cuticle, the parasite was placed on in the 99% lactophenol for 24 hours and identified at genus level.

The fillet digested in the pepsin HCl artificial digestion method is used to harvest any encysted larva in the fish muscle. This method was specifically optimized for the recovery of infective larval nematodes (e.g., L3 stages of Anisakidae) from the musculature is relevant here to show why this muscle digestion is necessary [43]. Artificial digestion solution was prepared from 10gram of pepsin powder, 16mL of 25% HCl in 2 litres of tap water prewarmed at 42°C in water bath. Briefly fish flesh was cut in to 1–2milimeter of small pieces using sharp knife. One-hundred-gram small piece of individual fish flesh was placed in 3liter beaker. The digestion solution was fully submerged the flesh; the beaker was covered with aluminium foil and heated on magnetic stirrer at 4^0°C^ for 4 hours to fully homogenise the tissue sample. We turned off the magnetic stirrer once observing the dis-appearance of any visible insoluble tissue. The sample was strained by laboratory sieve with approximate pore size of 70–100 μm. The content retained on the sieve was examined for any larvae of nematode under stereomicroscope. The collected worms were counted, recoded and preserved in 70% ethanol [44–50].

The morphological characteristics of anterior and posterior parts, including boring tooth, the ratio of ventricular appendix to intestinal caecum and the morphology of their tail were referred for characterization. The taxonomic identification of parasites was limited to genus level as the fish harbors mostly larval stages of many nematodes and trematode [51,52]. The helminthic parasites were identified using parasitological and morphologically approach using standard identification keys and pictorial guide [49].

### Prevalence and intensity of the parasite

*The prevalence report is calculated considering multiple parasite involvement from single host, i.e., one fish could be infected by different parasite groups or free from any parasite.* The prevalence and intensity of the parasite was calculated according to the formula [53].

Accordingly,


$$\begin{array}{c} \text{Prevalence}=\frac{\text{Number of individual of a host infected with a particular parasite species}}{\text{Number of hosts examined }}\;\times \text{1}\text{0}\text{0} \end{array}$$

$$\begin{array}{c} \text{Mean intensity}=\frac{\text{Sum of individuals of parasite species in a host sample }}{\text{Total no}.\text{ of infected individuals of the host in the sample }} \end{array}$$

$$\begin{array}{c} \text{Mean abundance}=\frac{\text{number of collected parasite }}{\text{Total number examined fishes }} \end{array}$$

### Data management and analysis

All collected raw data was entered into a Microsoft Excel database system and imported to be analysed using SPSS statistical software (version-17). Accordingly, descriptive statistics including frequencies, percentages, mean intensity and abundant intensity were presented in tubular form. The chi-square test (χ^2^) was used for a possible explanation of associations between the prevalence of parasites and the selected variables (body weight, length, sex, and fish species) for the sampled fish. P-values less than 0.05 were taken as statistically significant.

### Ethical statements

All procedures were conducted in accordance with relevant guidelines and regulations. Ethical approval was obtained from the Research Ethics Review Committee of the School of Veterinary Medicine, Wollega University dated 20/04/2023 with minute number SVM.RERC/0023.

An official letter describing the objectives of the study was submitted to the Hawassa City Municipality and relevant fisheries authorities. The respective authorities reviewed the proposed study, acknowledged its relevance, and granted the necessary permission to conduct the research. Furthermore, all methods are reported in accordance with “ARRIVE” guidelines (https://arriveguidelines.org) for the reporting of animal experiments.

## Result

### Prevalence of fish parasite in relation to sex, body weight, total and standard length

**Fig 1:**
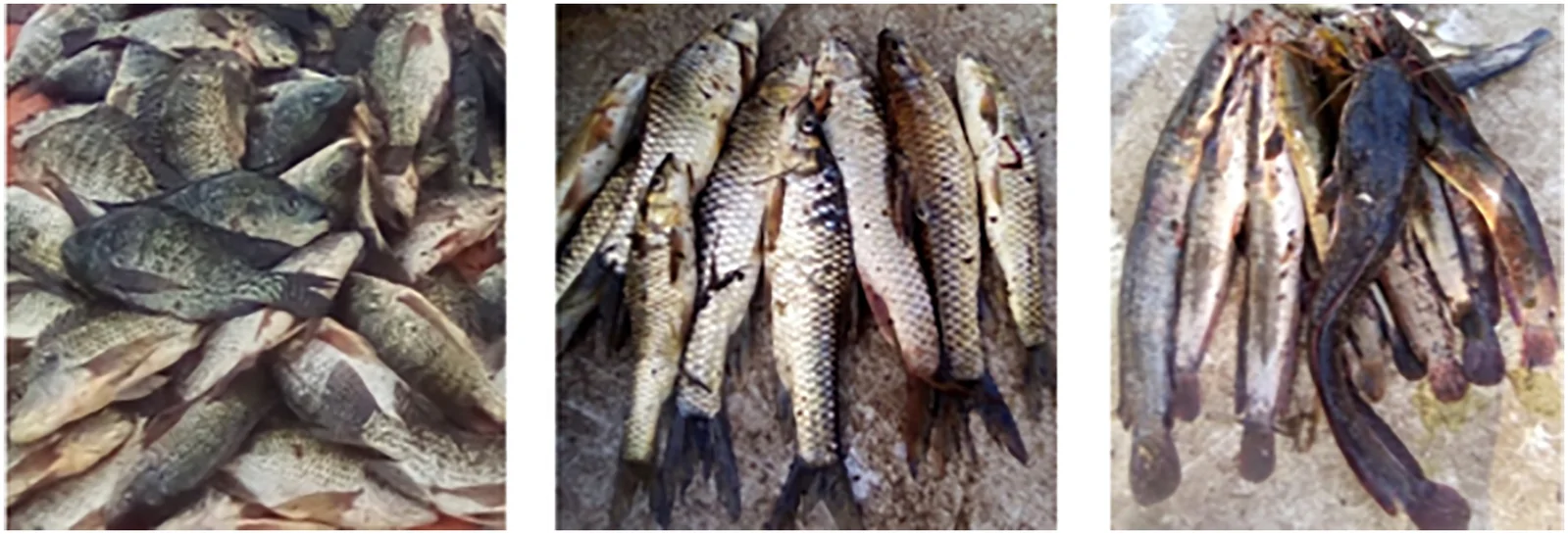
The commercially important fish species in Lake Hawassa. The left panel indicates *O. niloticus* (Nile tilapia or Koroso) while the middle panel shows *L. intermedius* also called *B. intermedius*, and finally the right panel shows *C. gariepinus* (Catfish or Ambaza).

During the sampling period, 265 fishes belonging to *O. niloticus* (tilapia) (n = 135), *L. intermedius (‘nech asa’*) also known as *Barbus intermedius* (n = 87), *C. gariepinus* (African catfish) (43) were sampled (Fig 1). The total length of the fish ranges from 10 cm to over 80 cm and the total weight ranges from 100g-2 Kg.

### Prevalence, mean abundance and mean intensity of parasites in fish

Of a total of 265 examined fish, 234 (88.30%) were found infested by at least one parasite group. The highest level of parasite occurrence was reported in *C. gariepinus* (93.10%) followed by *L. intermedius* (86.05%) and *O. niloticus* (85.93%). The minimum and maximum mean intensity of 6.8 and 8.6 is recorded in *O. niloticus and L. intermedius* respectively. The lower and higher mean abundance of 5.8 and 7.4 the parasite is also recorded in *O. niloticus and L. intermedius* respectively. The variations in parasite occurrence by fish species were not statistically significant (χ2 = 2.89; df = 2, *p* = 0.24) (Table 1). *O. niloticus* had the lowest mean intensity (MI = 6.79±0.17), and *L. intermedius* had the highest (MI = 8.59±0.37).

**Table 1:** Prevalence of parasitic occurrence with respect to fish species studied.

Species of fish studied	No. of fish affected	Number of parasites from each fish	Total parasite collected	Prevalence (%)	Mean intensity 95%CI	Mean abundance	χ2	*P- value*
***O. niloticus (n* = *135)***	116	6-9	788	85.93	6.79 ± 0.17	5.84 ± 0.47	2.89	0.24
***C. gariepinus (n* = *87)***	81	4-11	642	93.10	7.92 ± 0.23	7.37 ± 0.42		
Labeobarbus **i*ntermedius* (n = 43)**	37	5-10	318	86.05	8.59 ± 0.37	7.40 ± 0.40		
**Overall (N = 265)**	**234**	**4-10**	**1748**	**88.30**	**7.77**	**6.87**		

### Prevalence, mean abundance and mean intensity of parasites in fish

The result indicated that 56.3% of male and 43.7% of female in *O. niloticus*, 52.9% male and 47.3% female in *C. gariepinus*, and 55.8% male and 47.1% female in *L. intermedius* were affected by one or more parasites.

The smallest body weighted fishes are highly affected by one or more parasites (90.37% in 100-300g, 75.86% in 190-400g and 79.09% in 100-200g body weight in *O. niloticus*, *C. gariepinus*, and *L. intermedius*), respectively. This body weight class is the lower body weight in respective of fish species. Conversely, the parasite occurrence increases as body length of the fish host increase. Accordingly, 70.37%, 95.40% and 93.02% of *O. niloticus*, *C. gariepinus*, and *L. intermedius* respectively are infected by fish parasites at the larger standard body length. The distribution of parasites with sex, body weight, and the total and standard length of *O. niloticus*, *C. gariepinus* and *L. intermedius* fish showed not statistically significant association (Table 2).

**Table 2:** Parasite occurrence in relation to sex, bodyweight, total and standard length of fishes.

Species of Fish studied	Variable category		Number of examined	Number of fishes infected by parasites	Prevalence (%)	Fisher’s exact test	*p-value*
***O. niloticus* (n = 116)**	Sex	Male	76 (56.3)	64	84.21	0.68	0.481
		Female	59 (43.7	52	88.14		
	Body Weight (gram)	100-300	122 (90.37)	105	86.07	3.37	0.304
		301-500	11 (8.15)	10	90.91		
		>500	2 (1.48)	1	50.00		
	Total Length (in centimetres)	10-16	32 (23.70)	27	84.38	0.29	0.865
		17-25	92 (68.15)	79	85.87		
		>25	11 (8.15)	10	90.91		
	Standard Length (in centimetres)	<10	4 (2.96)	3	75.00	0.74	0.722
		10-15	36 (26.67)	30	83.33		
		>15	95 (70.37)	83	87.37		
***C. gariepinus* (n = 81)**	Sex	Male	46 (52.87)	42	91.30	0.48	0.483
		Female	41 (47.13)	39	95.12		
	Body Weight	190-400	66 (75.86)	62	93.94	0.67	0.737
		401-2000	19 (21.84)	17	89.47		
		>2000	2 (2.29)	2	100.00		
	Total Length (in centimetres)	28-50	85 (97.70)	80	94.12	5.90	0.492
		51-75	2 (2.29)	1	50.00		
		>75	0 (0.00)	0	0.00		
	Standard Length (in centimetres)	<25	4 (4.59)	3	75.00	2.2	0.343
		25-70	0 (0.00)	0	0.00		
		>70	83 (95.40)	78	93.98		
***L. intermedius* (37)**	Sex	Male	24 (55.81)	21	87.50	0.30	0.757
		Female	19 (44.19)	16	84.20		
	Body Weight	100-200	34 (79.07)	31	91.18	4.9	0.125
		201-460	7 (16.28)	5	71.43		
		>460	2 (4.65)	1	50.00		
	Total Length (in centimetres)	15-20	3 (6.98)	2	66.67	1.00	0.598
		21-26	31 (72.09)	27	87.09		
		>26	9(20.93)	8	88.89		
	Standard Length (in centimetres)	<10	1 (2.33)	1	100.00	2.10	0.302
		10-20	2 (4.65)	1	50.00		
		>20	40 (93.02)	35	87.50		

### Prevalence of parasitic occurrence with respect to anatomical locations

A total of 265 fish were examined, including *Oreochromis niloticus* (n = 135), *Clarias gariepinus* (n = 87), and *Barbus intermedius* (n = 43), with an overall infection/infestation rate of 88.3% (n = 234). In total, 1,748 parasites were collected and classified into nematodes, digenean trematodes, monogeneans, myxozoans, crustacean ectoparasites, and leeches. The most common nematodes were Contracaecum spp., particularly prevalent in *C. gariepinus* (50.57%). Digenean trematodes included Clinostomum, Euclinostomum, Diplostomum, and encysted metacercariae, with the latter reaching 68.97% in *C. gariepinus*. Monogenean infections with *Dactylogyrus* spp. occurred mainly in *O. niloticus* (28.15%). *Myxobolus* spp., leeches, Argulus, and Dolops species were also recorded across hosts. Mean parasite intensity was 8.59 in *L. intermedius*, 7.92 in *C. gariepinus*, and 6.79 in *O. niloticus*, while mean abundance was 7.40, 7.37, and 5.84, respectively.

**Fig 2:**
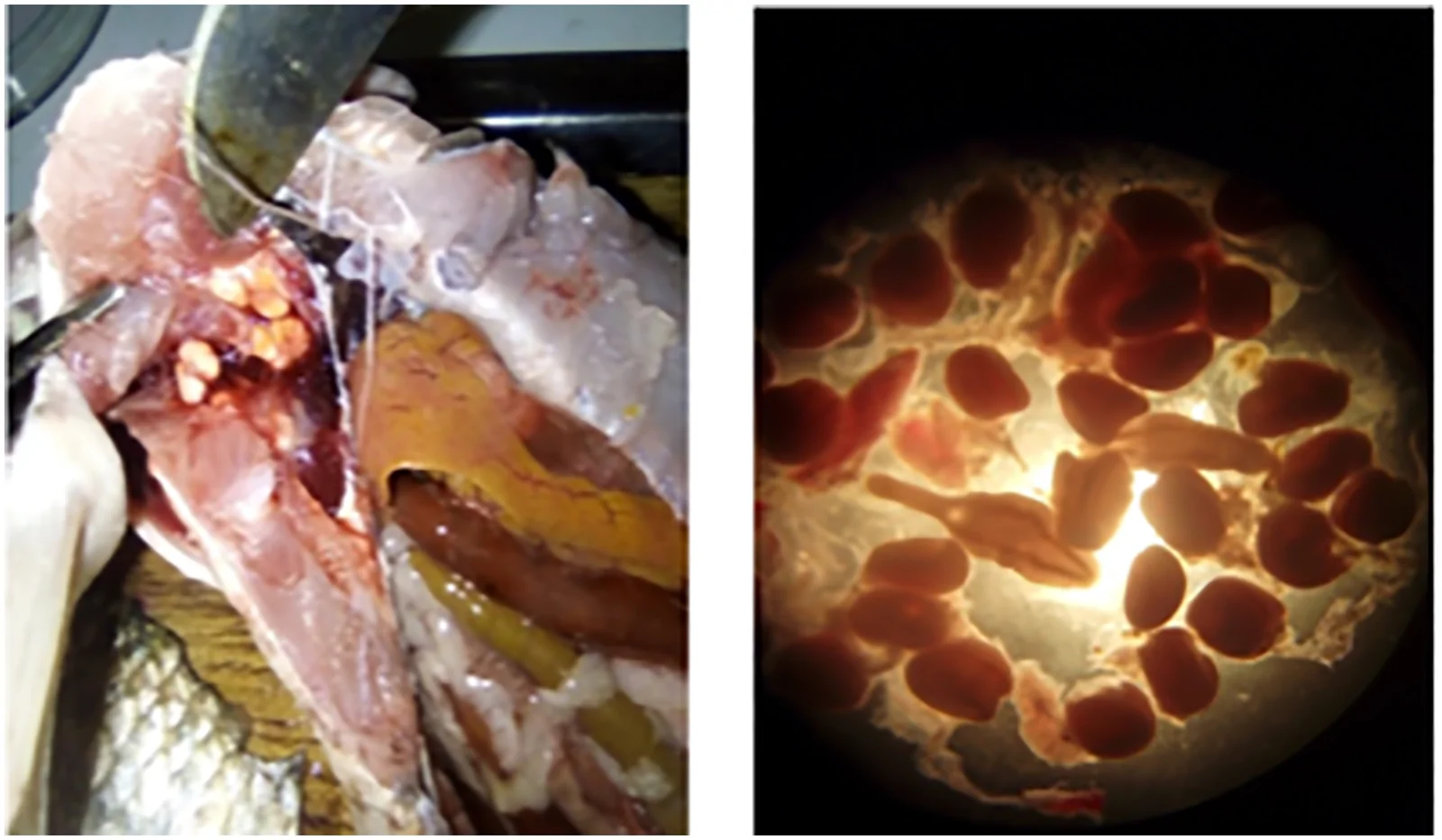
Encysted (left) and Excysted (right) *Clinostomum* species. The left panel shows the parasite enclosed within its cyst, while the right panel shows the metacercaria after excystation, exposing its characteristic elongated body morphology.

**Fig 3:**
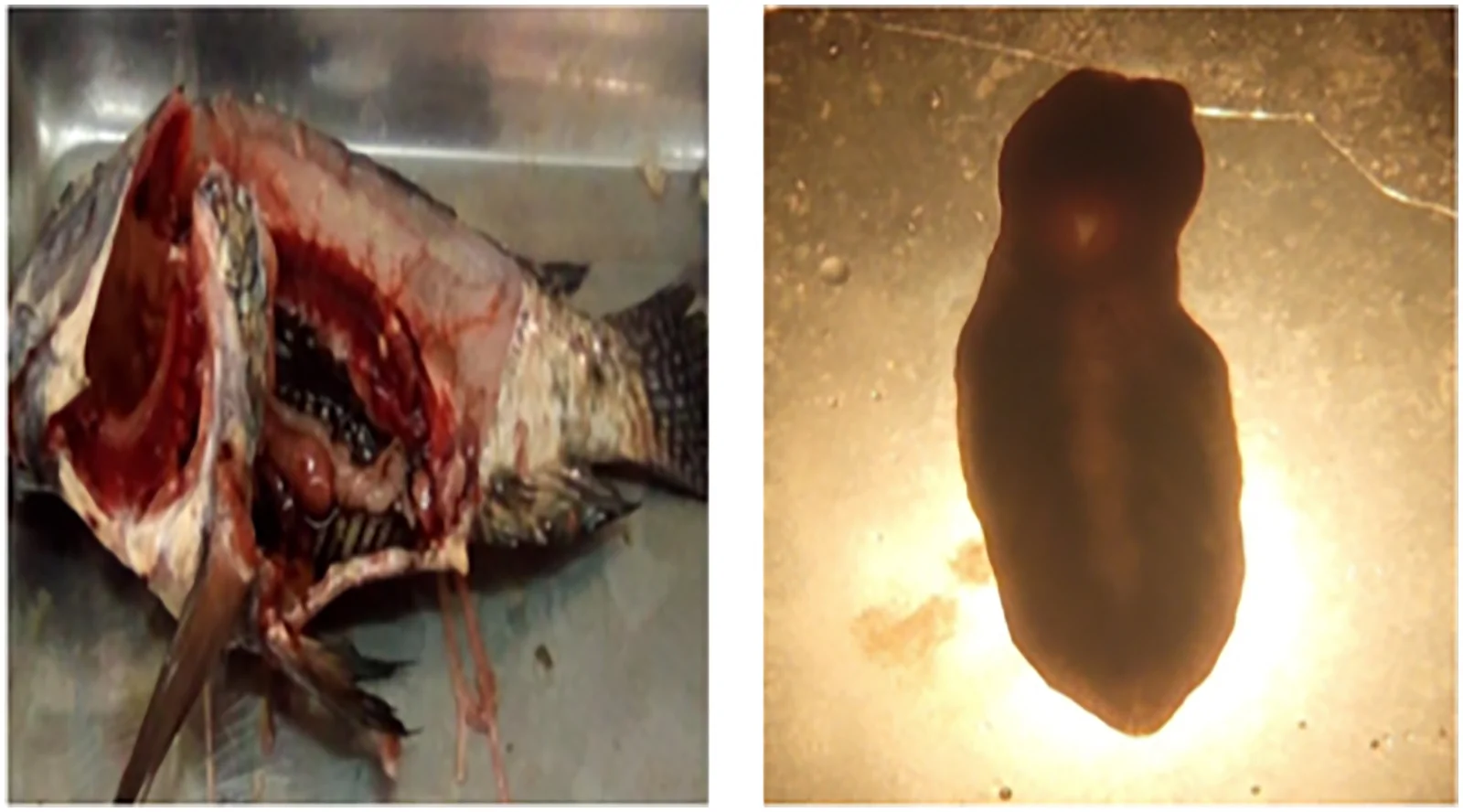
Encysted (left) and excysted (right) *Euclinostomum* spp.recovered from the kidney of *O. niloticus.* The encysted metacercaria is enclosed within a transparent cyst, whereas the excysted metacercaria has emerged from the cyst, revealing the elongated body morphology.

**Fig 4:**
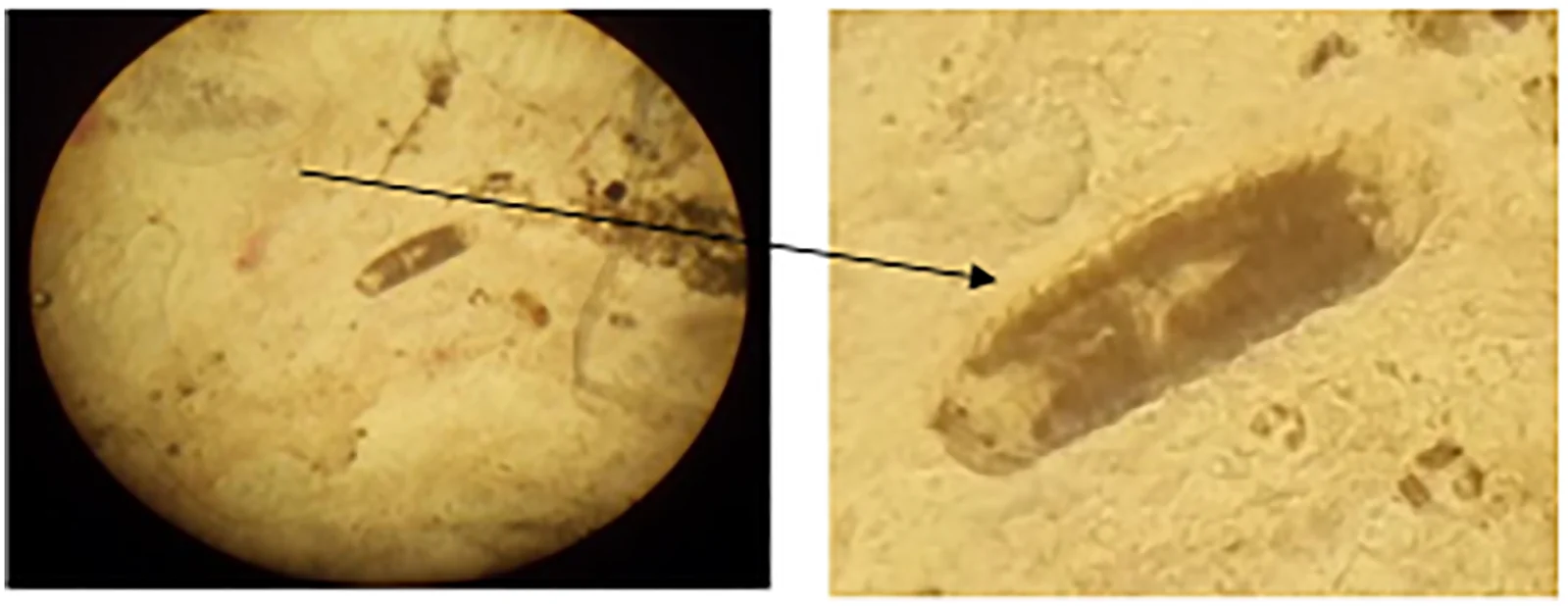
*Dactylogyrus* spp. recovered from the gill of Nile tilapia *(O. niloticus)*, showing the characteristic morphology of the monogenean parasite, including the elongated body and posterior attachment organ (haptor) equipped with sclerotized hooks used for attachment to the gill tissue.

**Fig 5:**
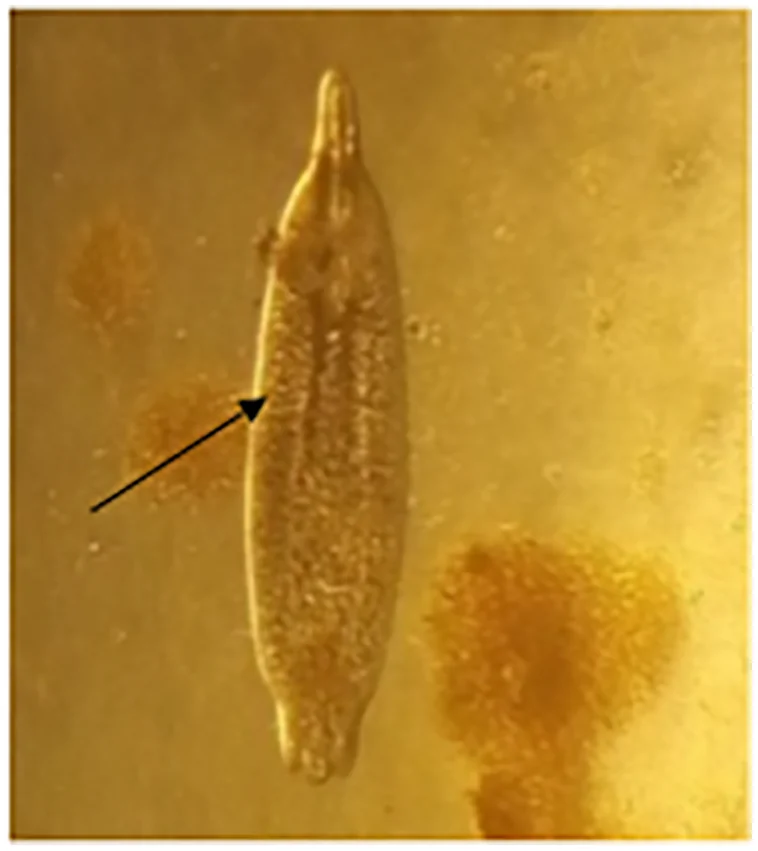
*Diplostomum* spp. recovered from the brain tissue of African catfish.

**Fig 6:**
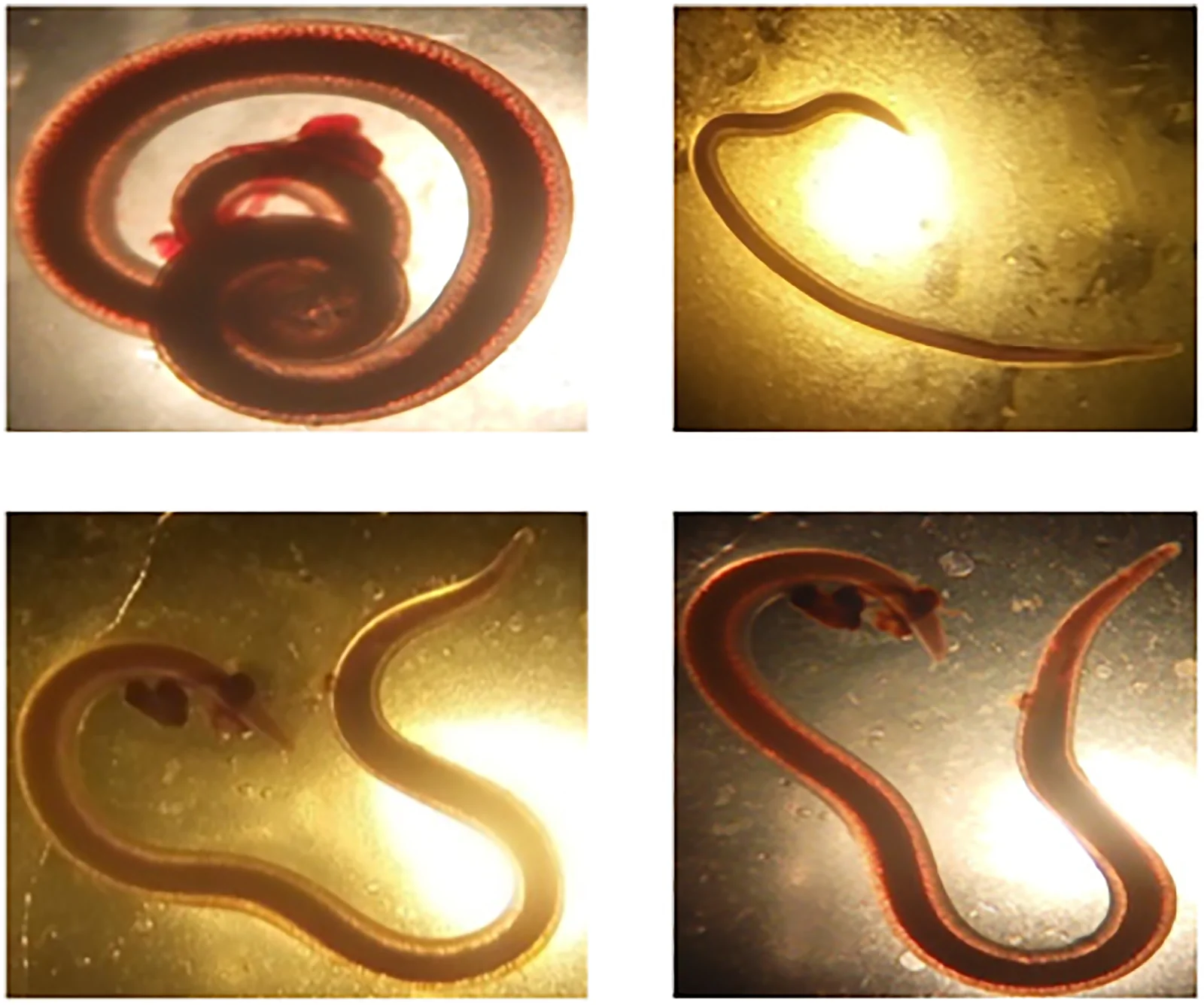
Representative photographs of Contracaecum spp. recovered from two freshwater fish hosts. The top two panels show *Contracaecum* spp. recovered from the heart of *O. niloticus*, while the bottom two panels show *Contracaecum* spp. recovered from the mesentery of African catfish *C. gariepinus*. The images illustrate the gross morphology of the recovered nematodes.

*Clinostomum* species (Fig 2) were the dominant trematodes in *O. niloticus* with a prevalence of 65.19% infecting brachial cavity, pericardial cavity, peritoneal and ocular cavity, followed by *Euclinostomum* species (Fig 3) with 34.07% which infects kidney, liver and brachial cavity. *Dactylogyrus* species (Fig 4) from the gill and *Diplostomum* species (Fig 5) from the brain tissue also showed higher prevalence, accounting 28.15% and 24.44%, respectively. *Contracaecum* species (Fig 6) one of the zoonotic anisakidae infecting 26.67% of *O. niloticus* species (Table 3).

**Table 3:** Prevalence with respect to the recovered parasites (n = number fishes sampled in each group).

Variables	Site of localization	Parasite species recovered	Number of infected fish	Prevalence (%)
***O. niloticus*** **(n = 135)**	*Clinostomum* species	Branchial cavity, pericardial cavity, between cranium and pharyngeal teeth, ocular cavity	88	65.19
	*Euclinostomum* species	Kidney, liver, branchial cavity	46	34.07
	*Contracaecum* species	Heart, pericardial Cavity	36	26.67
	*Diplostomum* species	Brain tissue	33	24.44
	*Dactylogyrus* species	Gill	38	28.15
	*Myxobolus* species	Intestinal contents	20	14.81
	Leeches	Skin, gills, mouth	16	11.85
	*Argulus* species	Skin, fins	9	6.67
	*Dolops* species	Gill, fins	5	3.70
***C. gariepinus*** **(n = 87)**	*Digenean encysted metacercaria*	Musculature	60	68.97
	*Contracaecum* species	Mesentery	44	50.57
	*Diplostomum* species	Brain tissue	41	47.13
	Unidentified parasite	Mesentery	1	1.15
	*Dactylogyrus* species	Gill	41	47.13
	Encysted cestode larvae	Stomach	5	5.75
	Leeches	Skin, gills, mouth	7	8.04
	*Argulus* species	Skin, gill, fins and oral cavity	18	20.69
	*Dolops* species	Skin, gill, fins, Oral cavity	38	43.68
***L. intermedius* (n = 43)**	*Contracaecum* species	Mesentery	16	37.21
	Unidentified parasite	Mesentery	2	4.65
	*Dactylogyrus* species	Gill	27	62.79
	Leeches	Skin, gills, mouth	5	11.63
	*Argulus* species	Skin, fins	3	6.98
	*Dolops* species	Skin, fins	4	9.30

**Fig 7:**
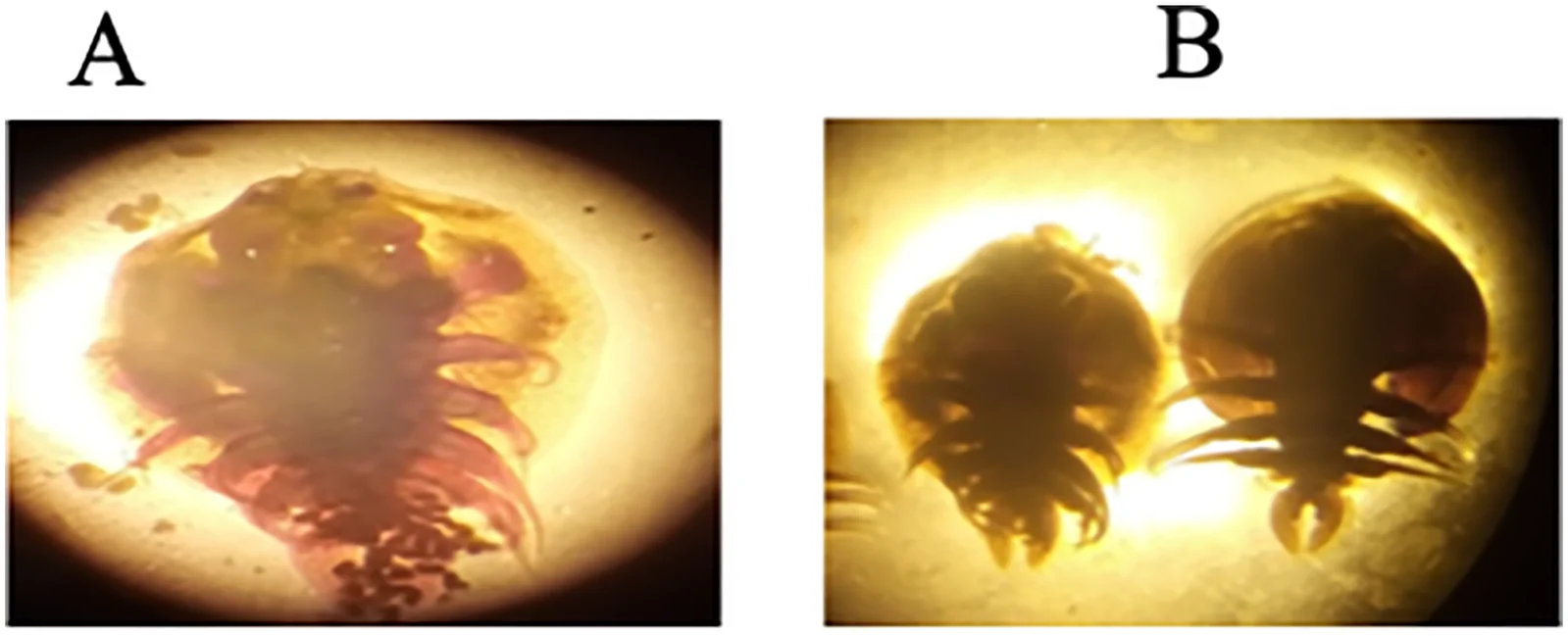
*Argulus* spp. collected from C. *gariepinus.* (A) *Argulus* specimen attached to the gill. (B) *Argulus* specimen recovered from the fin of *C. gariepinus*.

**Fig 8:**
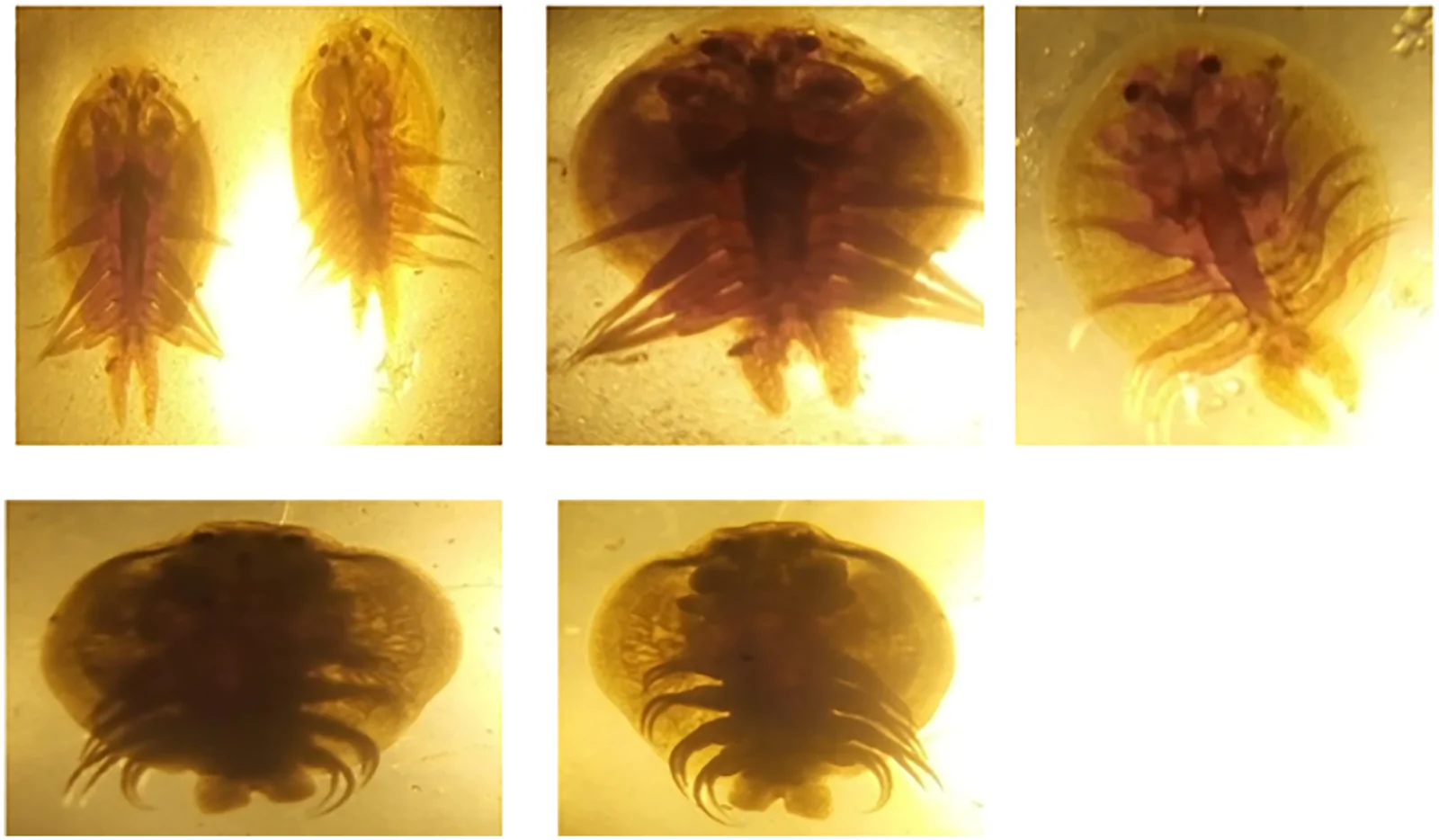
Representative images of Dolops sp. (Branchiura: Argulidae) collected from Clarias gariepinus. The top three panels show *Dolops* sp. attached to the gill filaments, illustrating the parasite’s characteristic dorsoventrally flattened body and firm attachment to the gill tissue. The bottom two panels present the dorsal (left) and ventral (right) views of specimens collected from the skin of *C. gariepinus*, highlighting the key morphological features and attachment structures used for taxonomic identification.

*Digenean encysted metacercaria 68.97%, Contracaecum* species *50.57% and Diplostomum species 47.13% infecting musculature, mesentery and brain tissues respectively are the dominant helminths infecting C. gariepinus groups. Contracaecum* species (37.21%) and *Dactylogyrus* species (62.79%) are the dominant parasites affecting *L. intermedius* infecting mesentery and gills respectively. The dominant helminth in *C. gariepinus* was recorded as digenean encysted metacercaria (68.97% prevalence). Identification to genus level was not possible due to heavy encapsulation within host tissue, which obscured key morphological features required for precise taxonomic determination. Given the high intensity and overlapping morphology of the cysts, these parasites were conservatively categorized as digenean metacercariae. In contrast, *O. niloticus* harboured trematodes such as *Clinostomum* and *Euclinostomum*, which were identifiable based on well-preserved sclerotized structures. The common ectoparasites infecting fish species in Lake Hawassa are Argulus species (Fig 7A and 7B) and *Dolops* species (Fig 8) (Table 3).

### Distribution, location and number of ectoparasites on the fishes sampled

*Contracaecum* species is the most common parasites found on *O. niloticus with* 373 total number of parasite collected from 224 infected fishes with mean intensity of 3 parasites per fish. Different parasites have different affinity for the body of the fishes where the external parasites mostly affecting gills, skin and gills. *Leeches* are also found infecting all the three fishes studied whereas *Monogenea* and *Myxosporea* parasites are infecting gills and intestinal contents (Table 4).

**Table 4:** Anatomical distribution of the different parasites.

Type of parasite	Parasite class	Anatomical location of occurrence	Number of fish infected	Number of parasites collected
***Clinostomum* species**	Trematoda	Branchial cavity, pericardial cavity, between cranium and pharyngeal teeth, ocular cavity	88	255
***Euclinostomum* species**	Trematoda	Kidney, liver, branchial cavity	46	136
***Contracaecum* species**	Nematoda	Heart, pericardial cavity	124	373
***Diplostomum* species**	Trematoda	Brain tissue	74	200
***Dactylogyrus* species**	Monogenea	Gill	106	276
***Myxobolus* species**	Myxosporea	Intestinal contents	20	88
***Digenean encysted metacercaria***	Trematoda	Musculature	60	189
**Encysted cestode larvae**	Cestode	Stomach	5	19
***Argulus* species**	Crustaceans	Skin, fins	30	79
***Dolops* species**	Crustaceans	Gill, fins	47	96
**Leeches**	Hirudinea	Skin, gills, mouth	28	34
**Unidentified internal parasite**	Not identified	Mesentery	1	3
**Total collected parasites**		**1748**		

## Discussions

Parasitic diseases are a major constraint to the sustainable development of aquaculture worldwide [18]. Among aquatic diseases, parasitic infections are the most prevalent, accounting for approximately 54% of cases, compared to 22% attributed to bacterial infections [54]. Some of these parasites are known zoonotic pathogens to humans [23]. Most animal parasites are found in fish and even apparently health wild fish have some parasites burdens [55].

In the current study the commercially important fish species including O. *niloticus, C. gariepinus,* and *L. intermedius* from the Hawassa assessed for the presence of any external and internal parasites such as nematodes, cestodes, trematodes, crustaceans, protozoan families *Monogenea*, *Myxosporea,* and leeches and the associated risk factors (Tables 1–4, Figs 2–7). In Ethiopia, endoparasites are commonly reported in fish from Lake Awasa, Lake Lugo, and Lake Tana [5]. The current prevalence of fish parasites was found to be 88.30%, differing with fish species, sex, body weight, standard length. This result agreed with Mitiku [56] who indicated 83.4% prevalence of parasites from central Ethiopia from freshwater culture and capture. Similar prevalence report of 77.4% to 88.3% was reported in Victoria Lake [57] Kenya, 88.2%−91.2% by Mgbemena et al [58] from Nigeria. The current finding is found greater than earlier reports of 79% by [34] at Lake Hawassa, 69.25% by [59] at Lake Ziway, 66.3% by [19] at Koka reservoir.

The present prevalence 88.30% was markedly higher than previous reports from several Ethiopian and regional water bodies, including Lakes and farms Taita Taveta county of Kenya (52.3%) [60], Lake Hashange of Ethiopia (37.6%) [61], Lake Baringo (34.8%) [27] (Kiprono, 2017), Jimma (30.9%) [62], Lake Chamo (21.4%) [12], and Lake Ziway (20.8%) [63]. Lower prevalence rates were reported from Kiri Reservoir, Nigeria (37.5%) [64]. These findings indicate that the current study area exhibits comparatively elevated infection levels relative to earlier investigations. Furthermore, higher prevalence reports of 93% were reported from Midmar reservoir, Adwa, Northen Ethiopia by [24] in *O. niloticus*. The difference in parasite prevalence among different studies may be attributed to ecological, methodological, and host-related factors. Variations in water quality, agroecological conditions, and seasonal changes can influence parasite survival, intermediate hosts, and transmission dynamics. Differences in fish species composition, feeding habits, age, sex, body condition, and immune status may also contribute to varying susceptibility to internal and external parasites [65].

The prevalence, mean intensity and mean abundance of the parasites vary with species of the fishes sampled. Accordingly, prevalence rate of 85.9%, 93.1% and 86.1% was registered in O. *niloticus, C. gariepinus,* and *L. intermedius* respectively. The slightly higher prevalence rate in *C. gariepinus* is comparable with report by [35] and [19] in which the rate of parasitic infection is higher in *C. gariepinus*. Moreover, Truter et al [66] reported the presence of over 107 metazooan with well characterized life cycle and general morphology infecting *C. gariepinus* in Africa.

Mean intensity and apparent parasite density varied among fish species, with the highest values recorded in *L. intermedius* (8.59; 7.40), followed by *C. gariepinus* (7.92; 7.37) and *O. niloticus* (6.79; 5.84). Similar findings have been reported by [67] who observed that parasite intensity and prevalence differ according to host size. These variations are influenced by abiotic factors such as water temperature and host-related factors including behaviour, age, sex, resistance, and mortality [68–70].

The higher prevalence of internal helminths in the current study could be attributed to a number of factors, including the lack of an effective waste disposal and management system, which led to the processing of post-harvest fish and the disposal of the waste (scraps and gastrointestinal contents) into the lake and its shorelines; environmental pollution from detergents used for washing clothes on the lake shore; and some birds that could serve as intermediate or final hosts for helminths [71]. This shows that the host-parasite interaction and changes in abiotic factors, including sewage, irrigation, sanitation, and flooding, frequently causes the distribution of fish parasites to vary from one habitat to another, and that leads to environmental stresses that impair the host’s immune system [72]. Compared to mammals, fish have a lower immune response to foreign proteins [73]. Several parasites are known to alter the behaviour of their hosts, predisposing them to predation [74]. The difference in prevalence from different lakes in Ethiopia and other parts of the world may be attributed to the difference in behaviour of different fish species, the quality of water, the presence of other stressors such as pollution and climate change [54]. The increase in global temperature leads to fluctuation in aquatic environment whereas the elevated water temperature could potentially compromise the fish immune system leading to fish more vulnerable to disease conditions [75,76]. Fish parasite vaccines remain limited, and many important fish pathogens have developed resistance to commonly used antimicrobial agents, creating significant challenges for disease prevention and treatment in aquaculture [77].

In the present study, parasite prevalence was higher in males than females across *O. niloticus*, *C. gariepinus*, and *L. intermedius*. These values were lower than reports from Nigeria [58] but higher than those from Lake Hashenge, Ethiopia [61], and were comparable to findings from Nigeria and Upper Egypt [78]. Although some studies report higher infection in females, this has been attributed to behavioural factors such as increased shoaling and prolonged parasite exposure [78,79].

In the current study, parasite occurrence varied according to fish body weight and standard length. However, body size alone is not a reliable proxy for age, maturity, or cumulative exposure to parasites. Heavily infected fish may appear heavier due to parasite biomass [80,81], whereas fish with lower body weight may be more severely affected by one or more parasite species. This finding is consistent with previous studies indicating a general trend that smaller fish or fish with poor body condition are often more susceptible to parasitic infections, although this relationship is not universally observed. Such susceptibility may be associated with limited energy allocation for immune defence and reduced behavioural adaptations in fish with poor body condition [78,82]. However, several biological and ecological factors determine which parasite groups are more closely associated with host size [83].

In general, larger fish tend to harbour a greater diversity and abundance of parasites because they provide more resources and a larger habitat for parasite establishment. In contrast, smaller fish may be more vulnerable to specific larval stages of parasites, potentially due to increased exposure to predation [84]. Although parasites can negatively affect host health, parasitic infection may also occur because of compromised host condition [82]. Furthermore [85], reported that host size and age are generally positively associated with parasite species richness and infection intensity in fish hosts. Similarly, Nikolaev et al [86] concluded that older fish have a higher likelihood of accumulating parasites throughout their lifetime.

Identification of parasites with significant host and tissue specificity is made much easier by knowledge of fish hosts, while other parasites are detected due to their widespread presence and lack of host specificity [55]. In line with this, *Contracaecum* was the most frequently encountered nematode parasite found in the current study but lower than previous reports by [34] in Lake Hawassa, who reported 39.67% in *O. niloticus*, 80.5% in *C. gariepinus*, and 36% in *L. intermedius*. Moreover, Kaba et al [12] reported much lower prevalence of 21.4% from *O. niloticus* and *Lates niloticus* from Chamo Lake, Arbaminch. However, it is higher as compared to Gulelat *et al* [19] at Koka reservoir, 24.82% in *C. gariepinus* and 16.49% in *L. intermedius*. This result suggests that the distribution of *Contracaecum's* parasites can vary from one habitat to another due to host parasite relationships and changes in water quality parameters. Recently *Contracaecum bancrofti* a unique Australian species and warming the fish tissue leads to emergence of small *Contracaecum* larvae <1 mm from intestinal tissue [87]. *Contracaecum* are economically important parasites with zoonotic and one health significance where human infection through ingestion of infected tissue is the major route [12]. Adult *Contracaecum* spp. are found in the stomach of marine mammals or piscivorous birds, and larval stages infect a wide range of invertebrates and fish species [87].

The host distribution of *Clinostomum* species was discovered in the current study to be much higher than previous studies by [19,78,88] who revealed a prevalence of 27.4%, 18.8%, and 15.2% in Lake Abaya, Koka Reservoir, and Lake Hashange, respectively. However, the prevalence of this parasite is lower than that found by [34] who reported 75.7% prevalence from Lake Hawassa. The variations may result from variations in the physiochemical characteristics of water bodies and the intermediate hosts of the parasite. Digenean fluke, *Euclinostomum* species from *O. niloticus* were detected in the kidney, liver, and branchial cavity. This finding was in line to previous reports from Ethiopian lakes and water bodies [34,56]. These variations are likely influenced by differences in parasite–host life cycles and environmental conditions affecting transmission dynamics.

Khan et al [89] reported that prevalence of 70–92% metacercarial infection belonging to *C. complanatum* was reported concluding the seasonality of the infection. He stated that the high prevalence of infection of the parasite during summer may be because of the time of emergence of cercariae in high temperature from snail vector and the environment. Digenetic trematodes, or flukes, belonging to the genera *Clinostomum* and *Euclinostomum* infect many fish species all over the world. These parasites are found in freshwater and saltwater habitats worldwide [90]. The coexistence of *Clinostomum* and *Euclinostomum* in some fish host could imply the shared intermediate hosts, environmental conditions, or ecological overlap [91]. The *digenetic trematode Clinostomum* spp. commonly referred to as yellow grubs when in their larval form have complex and involves three hosts including freshwater snails as intermediate host, other fish or amphibians and piscivorous birds as definitive host. Humans are also accidental hosts if they consume undercooked or raw fish meal a practice widely known in Ethiopia [31].

In fish, encysted metacercaria are not merely superficial, they often embed deeply into critical tissues such as musculature, gills and buccal cavity, and cranial and pericardial regions, cranial and pericardial regions causing necrosis, inflammation, fibrosis and oxidative response which could compromise immune function and metabolism [71].

Lower prevalence rate of *Diplostomum* species, 24.44 and 47.13% respectively was found in the brain tissue of *O. niloticus* and *C. gariepinus*. This finding is lower as compared to the previous studies 77.93% of the gill cavity of *O. niloticus* at Lake Baringo, Kenya [27]*;*83.3% from cranial cavity of *C. gariepinus* sampled at Tanzania [92]; 100% of the cranial cavity of *C. gariepinus* was from Lake Hawassa [93]; 77.93% of the cranial cavity of *C. gariepinus* [19]. Significantly lower prevalence rate of 1.03% *Diplostomum* species was reported from the eye of *L. intermedius* from Lake Abaya [19]. According to the current findings, Degenian encysted metacercaria frequently infect *C. gariepinus* muscle. This finding is in line with the findings of [94] and [95], who conclude that these parasites commonly affect fish in coastal marine systems, which exhibit complex life cycles that typically involve snails as first intermediate hosts, macroinvertebrates, such as bivalves and crustaceans, or vertebrates as second intermediate hosts, and fish, birds, or mammals as final hosts. Moreover, *Clinostomum* and *Euclinostomum* spp. are known to infect fish-eating birds as definitive hosts, where they can have important health and ecological implications [71].

External parasites have strategic survival reasons to inhibit specific microhabitat on fish body. This specific microhabitat may provide availability of food, reproductive success, enhanced attachment or reduce the pressure of predator [26]. in the current finding, the crustacean parasites *Argulus* and *Dolops* species were found infecting gills, fins, skin, and oral cavity of the three fish species. This finding is lower as compared to the previous study by [34] in Lake Hawassa, who found 5.33% and 8.33% in *O. niloticus*; 44.5% and 63.5% in *C. gariepinus,* and 6% and 17 in *L. intermedius.* Moreover, Mitiku [56] reported 7.8% of external parasites from *O. niloticus* at Sebeta ponds. Argus species is more commonly known as fish lice, are member of large groups of branchiuran parasite that infest and cause fish disease. They are crustaceans and crabs, lobsters, and shrimp. There are over 100 species infecting freshwater fishes with *Argulus foliaceus, A. coregoni*, *A. japonicus* common in freshwater fishes [96]. According to a study by [66] *A. africanus* and *A. rhipidiophorus* are prevalent crustacean parasites in Ethiopian fish, especially those found in Lake Hawassa. *Argulus japonicus* is an alien species that has been introduced globally and is known to damage fish such as *C. gariepinus*, while it is not specifically listed as being present in Ethiopian fishes [34,66].

*Dactylogyrus* species was find residing in the gills of fishes. The current prevalence report of 3.7–43.6% was found higher than the reported 3.96% by [97] in *O. niloticus* from *Gilgel* Gibe I Dam, and 31% by [98] in common carp in the northeast of Iran from the gill of sampled fish. *Myxobolus* species from the intestinal contents of *O. niloticus* were also found in the current study, which was not reported elsewhere in Ethiopia. How ever the prevalence rate of *Myxobolus* species in the current finding was lower than previous reports by [99] in the Nile River, Egypt, and [100] in Cameroon. Higher prevalence rate of *Myxobolus* infection including two new species was reported from Botswana [101].

In addition to the infections caused by parasites and other pathogens, several factors are threatening the quality of Hawassa lake from which human practice along the edge of the lake is the dominant factor. Memberu [13] have observed that the quality of the Hawassa lake is inadequate for maximum production of fishes and needs mitigation measures to control eutrophication and pollution flow. Anthropogenic activities, illegal fishing practices such as the use of narrow mesh nets, a lack of community awareness regarding fishery management, and a lack of fish stocks are the main conservation issues facing Ethiopian fish and fisheries sector [102].

The presence of parasites in edible part of the fish represent a public health threat since the presence of those parasites are responsible for causing infection in humans with significant outcome [103]. Knowledge of how parasites such as *Contracaecum* species, flukes and other pathogenic parasites are transmitted, their reservoir animal, their specific reservoir animals, the specific pathogen involved, directly impacts prevention and control strategy [104]. The focus of control methods should be on health education to promote appropriate control measures including safely disposing of infected fish [23]. As prevention and control methods for each pathogen varies, however several practices are recognised as effective for reducing the risks at the community and personal level. Safe and appropriate guidelines for animal care in the agricultural sector helps to reduce the potential for food borne zoonotic disease outbreaks through ingestion of animal food or some vegetables. Standards for clean drinking water and waste disposal as well as protection for surface water in the natural environment are important and effective control means [104].

### Limitation of the study

This study has not stablished the characterisation of the parasite at species level, and it only focuses describing the parasite at genera level. Furthermore, it is necessary to evaluate how the evaluation of water quality affects fish parasites. Another drawback is that parasites can only be identified at the genus level. The pepsin–HCl artificial digestion method could have some limitation as it was not equally applied to all organs; particularly the gastrointestinal tract, where parasites deeply embedded within tissues. We acknowledge that, this methodological limitation may have reduced the detection of a broader range of parasites and could have led to an underestimation of parasite diversity and burden.

## Conclusions

In the current study, internal and external parasites are found in fishes with respect to different anatomical location, body size and sexes. The parasites recovered belongs to *crustaceans, monogenean, digeneans, nematode, cestode,* and one protozoan and *Hirudinea* having a direct effect on fish health and meat quality and, thus, a direct effect on fish production and the economy. Among these*,* the *Dactylogyrus* species was the dominant external parasite followed by Argulus and Dolops species. It is found that parasite intensity and abundance vary with the host fish species. Anatomical and physiological variables of the fish such as body size, age, body weight and gender also found determining different prevalences.

The epidemiology of zoonotic fish-borne parasites such as *Contracaecum* species and flukes is strongly influenced by the ecology of intermediate hosts, environmental conditions, and human behavioural practices. Freshwater snails and fish serve as essential intermediate hosts in the life cycle of many trematodes and cestodes, and their abundance is highly dependent on ecological and climatic conditions. Variations in aquatic vegetation, water temperature, dissolved oxygen, and nutrient availability can significantly affect the survival and reproduction of these hosts, thereby influencing parasite transmission dynamics [17,105]. In lake ecosystems, eutrophication caused by agricultural runoff, organic pollution, and human activities may further increase the density of snail populations and alter fish community structures, creating favourable conditions for parasite persistence and amplification. Such environmental changes have been associated with increased transmission of zoonotic helminths in several endemic freshwater ecosystems worldwide.

Seasonal transmission dynamics also play a critical role in determining infection prevalence among fish and human populations. Rainfall patterns, flooding, and seasonal temperature fluctuations can alter the distribution and breeding patterns of both intermediate and definitive hosts. During wet seasons, increased water flow and habitat expansion may enhance the spread of infective stages, while warmer temperatures may accelerate parasite development within hosts. Similar observations have been reported in endemic regions where prevalence rates vary considerably between dry and rainy seasons due to ecological and environmental variability [106]. Understanding these seasonal patterns is important for designing effective surveillance systems and implementing timely public health interventions.

The findings of zoonotic parasites in communities with a tradition of consuming raw or undercooked fish raise important One Health concerns [12]. The close interaction among humans, animals, and aquatic environments facilitates the maintenance and transmission of these parasites. Consumption of raw fish is a well-recognized risk factor for fish-borne zoonotic infections, particularly in areas with poor sanitation [31] inadequate food safety awareness, and limited veterinary and public health control measures [104]. Therefore, integrated One Health approaches involving public health professionals, veterinarians, environmental experts, and local communities are essential to reduce transmission risks. Public education on safe fish preparation practices, environmental management to control intermediate hosts, and routine monitoring of aquatic ecosystems should be prioritized to minimize the burden of zoonotic parasitic diseases.

## Supporting information

S1 Table Presents the species categories of fish, including their body weight, standard length, total length, and presence of the parasite. Parasites were recorded as either internal or external. Fish length was measured using a measuring tape and ranged from 10 cm to more than 80 cm, while body weight ranged from 100 g to 2 kg. Parasite present ranged from single over 11.
(XLSX)
