# Supplementary material for: Prevalence of fish parasites from Lake Hawassa, Ethiopia
Source: PLoS One. 2026 Sep 11;21(9):e0357816. doi: 10.1371/journal.pone.0357816 (PMC13568496; doi:10.1371/journal.pone.0357816)
Supplement: Supplementary file 1 [file pone.0357816.s001.pdf]

PLOS ONE

Prevalence of fish parasites from Lake Hawassa, Ethiopia

--Manuscript Draft--

Manuscript Number:	PONE-D-25-59453
Article Type:	Research Article
Full Title:	Prevalence of fish parasites from Lake Hawassa, Ethiopia
Short Title:	Prevalence of Oreochromis niloticus, Clarias gariepinus, and Barbus intermedius
Corresponding Author:	DEBELA ABDETA EFA, Doctor of Veterinary Medicine<br>Addis Ababa University<br>Bishoftu, ETHIOPIA
Keywords:	Fish; Lake Hawassa; Parasites; Prevalence
Abstract:	Background<br><br>Fish provides quality protein, variety of vitamins, and minerals to human being. More than 17% of animal protein for global population is obtained from fish. An estimated 200 species of fishes are known to occur in Ethiopia. Fish parasites are the main cause of the significant threat to the fish biodiversity of Ethiopia's freshwater islands. Fish parasite of the lake Hawassa are hitherto poorly known and there are limited reports on the type of pastes across the different fish hosts.<br><br>Methods<br><br>a cross-sectional study was carried out in 2023- 2024 to estimate the prevalence of fish parasites and host factors. Internal and external organs was inspected for the presence of the parasite infection. Some external parasites were extracted from the fresh water immediately after fish were caught. The randomly selected host was classified based on species, sex, body weight, and the total body length. Parasites are identified morphologically at genera level using standard identification keys and pictorial guide. Analysed data were presented as frequencies, percentages, mean intensity, and mean abundance. Chi-square and P-values were considered for statistical association.<br><br>Results<br><br>in the current survey Oreochromis niloticus(n=135), Clarias gariepinus (n = 87), and Barbus intermedius (n = 43) were included. The parasite distribution varies with sex, body weight, and standard length of the studied fish. The overall infection/infestation rate is 88.30% (n = 234). A total of 1748 parasites classified under trematodes, nematodes, protozoa, crustaceans and Hirudinea was collected from O. niloticus (787), C. gariepinus (642) and B. intermedius (318). The internal parasites recovered from O. niloticus were Clinostomum (65.16%), Euclinostomum (34.07%), Contracaecum (26.67%), Dactylogyrus species (28.15%), and Diplostomum (24.44%). Significant infection level with Myxobolus species, leeches, Argulus, and Dolops species was also recorded. Digenean encysted metacercaria (68.97%) and Contracaecum species (50.57%) are the most common parasite species infecting C. gariepinus. Diplostomum species, Dactylogyrus species, leeches, Argulus species, and Dolops species pose a serious threat to fish health in lake Hawassa. The mean intensity of the parasites in B. intermedius, C. gariepinus and O. niloticus is found to be 8.59, 7.92 and 6.79 while mean abundance is 7.40, 7.37 and 5.84 respectively.<br><br>Conclusion<br><br>This study found that various body parts are contaminated with one or more parasites. Some of those parasites have established zoonosis or cause cross infection to fishes requiring urgent cooperation in performing pre-stock assessment for optimal production and maintaining the heath of fishery and hence human being.
Order of Authors:	DEBELA ABDETA EFA, Doctor of Veterinary Medicine

Powered by Editorial Manager® and ProduXion Manager® from Aries Systems Corporation

	Megersa Akasa
	Yobsan Tamiru
**Additional Information:**	
**Question**	**Response**
**Financial Disclosure**<br><br>Enter a financial disclosure statement that describes the sources of funding for the work included in this submission. Review the submission guidelines for detailed requirements. View published research articles from *PLOS ONE* for specific examples.<br><br>This statement is required for submission and **will appear in the published article** if the submission is accepted. Please make sure it is accurate.<br><br>**Funded studies**<br>Enter a statement with the following details:<br>• Initials of the authors who received each award<br>• Grant numbers awarded to each author<br>• The full name of each funder<br>• URL of each funder website<br>• Did the sponsors or funders play any role in the study design, data collection and analysis, decision to publish, or preparation of the manuscript?<br><br>Did you receive funding for this work?	The author(s) received no specific funding for this work.
**Competing Interests**<br><br>Use the instructions below to enter a competing interest statement for this submission. On behalf of all authors, disclose any competing interests that could be perceived to bias this work—acknowledging all financial support and any other relevant financial or non-financial competing interests.<br><br>This statement is required for submission and **will appear in the published article** if	The authors have declared that no competing interests exist.

Powered by Editorial Manager® and ProduXion Manager® from Aries Systems Corporation

<table>
<tr><td>the submission is accepted. Please make sure it is accurate and that any funding sources listed in your Funding Information later in the submission form are also declared in your Financial Disclosure statement.

View published research articles from PLOS ONE for specific examples.

NO authors have competing interests

Enter: The authors have declared that no competing interests exist.

Authors with competing interests

Enter competing interest details beginning with this statement:

I have read the journal's policy and the authors of this manuscript have the following competing interests: [insert competing interests here]

* typeset</td><td></td></tr>
<tr><td>Ethics Statement

Enter an ethics statement for this submission. This statement is required if the study involved:

• Human participants
• Human specimens or tissue
• Vertebrate animals or cephalopods
• Vertebrate embryos or tissues
• Field research

Write "N/A" if the submission does not require an ethics statement.

General guidance is provided below. Consult the submission guidelines for detailed instructions. Make sure that all information entered here is included in the Methods section of the manuscript.</td><td>N/A</td></tr>
</table>

**Format for specific study types**<br><br>**Human Subject Research (involving human participants and/or tissue)**<br>• Give the name of the institutional review board or ethics committee that approved the study<br>• Include the approval number and/or a statement indicating approval of this research<br>• Indicate the form of consent obtained (written/oral) or the reason that consent was not obtained (e.g. the data were analyzed anonymously)<br><br>**Animal Research (involving vertebrate animals, embryos or tissues)**<br>• Provide the name of the Institutional Animal Care and Use Committee (IACUC) or other relevant ethics board that reviewed the study protocol, and indicate whether they approved this research or granted a formal waiver of ethical approval<br>• Include an approval number if one was obtained<br>• If the study involved *non-human primates*, add *additional details* about animal welfare and steps taken to ameliorate suffering<br>• If anesthesia, euthanasia, or any kind of animal sacrifice is part of the study, include briefly which substances and/or methods were applied<br><br>**Field Research**<br><br>Include the following details if this study involves the collection of plant, animal, or other materials from a natural setting:<br>• Field permit number<br>• Name of the institution or relevant body that granted permission	
**Data Availability**<br><br>Authors are required to make all data underlying the findings described fully available, without restriction, and from the time of publication. PLOS allows rare exceptions to address legal and ethical concerns. See the PLOS Data Policy and FAQ for detailed information.	Yes - all data are fully available without restriction

A Data Availability Statement describing where the data can be found is required at submission. Your answers to this question constitute the Data Availability Statement and **will be published in the article**, if accepted.<br><br>**Important:** Stating 'data available on request from the author' is not sufficient. If your data are only available upon request, select 'No' for the first question and explain your exceptional situation in the text box.<br><br>Do the authors confirm that all data underlying the findings described in their manuscript are fully available without restriction?	
**Describe where the data may be found in full sentences. If you are copying our sample text, replace any instances of XXX with the appropriate details.**<br><br>• If the data are **held or will be held in a public repository**, include URLs, accession numbers or DOIs. If this information will only be available after acceptance, indicate this by ticking the box below. For example: *All XXX files are available from the XXX database (accession number(s) XXX, XXX.).*<br>• If the data are all contained **within the manuscript and/or Supporting Information files**, enter the following: *All relevant data are within the manuscript and its Supporting Information files.*<br>• If neither of these applies but you are able to provide **details of access elsewhere**, with or without limitations, please do so. For example:<br><br>*Data cannot be shared publicly because of [XXX]. Data are available from the XXX Institutional Data Access / Ethics Committee (contact via XXX) for researchers who meet the criteria for access to confidential data.*<br><br>*The data underlying the results presented in the study are available from (include the name of the third party*	All relevant data are within the manuscript and its Supporting Information files

and contact information or URL).<br>• This text is appropriate if the data are owned by a third party and authors do not have permission to share the data.<br><br>* typeset	
Additional data availability information:	Tick here if the URLs/accession numbers/DOIs will be available only after acceptance of the manuscript for publication so that we can ensure their inclusion before publication.

Powered by Editorial Manager® and ProduXion Manager® from Aries Systems Corporation

Click here to access/download;Manuscript;Debela et al_vp.docx

Prevalence of fish parasites from Lake Hawassa, Ethiopia

Debela Abdeta[1,4]*, Megersa Akasa[2], Yobsan Tamiru[3],
[1]College of Veterinary Medicine and Agriculture, Addis Ababa University, Bishoftu, Ethiopia
[2]College natural resource and agriculture, Ambo University, Gudar, Ethiopia
[3]School of Veterinary Medicine, Wollega University, Nekemte, Ethiopia
[4]Department of Veterinary Bioscience, Melbourne Veterinary School, Melbourne University, Melbourne, Australia
*Correspondence: Debela Abdeta
Tel. +251 913125925
Email: debela.abdeta@aau.edu.et, or debelaabdeta.efa@student.unimelb.edu.au

ABSTRACT

Background: Fish provides quality protein, variety of vitamins, and minerals to human being. More than 17% of animal protein for global population is obtained from fish. An estimated 200 species of fishes are known to occur in Ethiopia. Fish parasites are the main cause of the significant threat to the fish biodiversity of Ethiopia's freshwater islands. Fish parasite of the lake Hawassa are hitherto poorly known and there are limited reports on the type of pastes across the different fish hosts.

Methods: a cross-sectional study was carried out in 2023- 2024 to estimate the prevalence of fish parasites and host factors. Internal and external organs was inspected for the presence of the parasite infection. Some external parasites were extracted from the fresh water immediately after fish were caught. The randomly selected host was classified based on species, sex, body weight, and the total body length. Parasites are identified morphologically at genera level using standard identification keys and pictorial guide. Analysed data were presented as frequencies, percentages, mean intensity, and mean abundance. Chi-square and P-values were considered for statistical association.

Results: in the current survey *Oreochromis niloticus(n=135)*, *Clarias gariepinus* (n = 87), and *Barbus intermedius* (n = 43) were included. The parasite distribution varies with sex, body weight, and standard length of the studied fish. The overall infection/infestation rate is 88.30% (n = 234). A total of 1748 parasites classified under trematodes, nematodes, protozoa, crustaceans and *Hirudinea* was collected from O. *niloticus* (787), *C. gariepinus* (642) and *B. intermedius* (318). The internal parasites recovered from *O. niloticus* were *Clinostomum* (65.16%), *Euclinostomum* (34.07%), *Contracaecum* (26.67%), *Dactylogyrus* species (28.15%), and *Diplostomum* (24.44%). Significant infection level with *Myxobolus* species, leeches, *Argulus*, and *Dolops* species was also recorded. *Digenean encysted metacercaria* (68.97%) and *Contracaecum species* (50.57%) are the most common parasite species infecting *C. gariepinus*. *Diplostomum species*, *Dactylogyrus species*, leeches, *Argulus species*, and *Dolops species* pose a serious threat to fish health in lake Hawassa. The mean intensity of the parasites in *B. intermedius*, C. *gariepinus* and *O. niloticus* is found to be 8.59, 7.92 and 6.79 while mean abundance is 7.40, 7.37 and 5.84 respectively.

Conclusion: This study found that various body parts are contaminated with one or more parasites. Some of those parasites have established zoonosis or cause cross infection to fishes requiring urgent cooperation in performing pre-stock assessment for optimal production and maintaining the heath of fishery and hence human being.

Keywords: *Fish, Lake Hawassa, Parasites, Prevalence*

1. INTRODUCTION

The fish farming is one of the developing sectors in low- and medium-income countries offering significant potential for food security, growth of the economy and improvement of the livelihood (Ogello et al., 2013; Boyd et al., 2022). The demand for fish meat is steadily rising due to its high nutritional value, particularly essential fatty acids, which are ideal for human consumption (Ljubojevic et al., 2015a, 2015b, Brye, 2023). Fish resources are vital to support human well-being and economic development (Boyd et al., 2022). Aquaculture provides year-round jobs, supports resilient working water fronts and coastal communities and can provide sustainable economic growth. They also drive employment in other areas of industry such as seafood processing, feed and equipment manufacturing in different countries including Ethiopia (NOAA Fisheries, 2020).

Ethiopia is a landlocked nation that depends on its inland waterways for fish production. Some of the lakes and rivers in the country are used for fish production to feed its population while maintaining the nation's food security (Desalegn and Shitaw, 2021). The Ethiopian government has suggested aquaculture to increase fish output and as a substitute for food security and poverty alleviation (Deng, 2020). The data from the Ministry of Agriculture and Rural Development of Ethiopia, published in 2011, indicate that the total annual fish catch in Ethiopia rose from 14,000 tons in 1998 to 24,257 tons in 2011. Since then, there has been a plan to increase annual fish production by 65,000 tons per year. To achieve the plan the country constructed big dams such as Tendaho reservoir, Tekeze dam, Galgal Gibe I, II and III, and Renaissance dam in addition to the previously naturally used lakes (Gebremedhn and Tsegay, 2017). Today, aquaculture is seen as a crucial component of plans and strategies for rural and agricultural development in the country. The Ethiopian renaissance dam is emerging with a major fishery hub producing over 14,000kg of fishes daily (Food Business, 2025).

Lake Hawassa is one of the major lakes in the Ethiopian Rift Valley, situated close to Hawassa town and features an endorheic basin system. The water from the lake is essential to the nearby towns for recreational use, residential water use, farming, and fish harvesting (Memberu et al., 2021). This lake is one of the most fished water bodies in Ethiopia where *O. niloticus* accounts 90% of the annual catch (Tesgera and Bayou, 2021).

The freshwater fish fauna of Ethiopia is a mixture of Nilo Sudanic, East African and endemic forms (Skelton et al., 1991). The Nilo Sudanic forms those species represented under *Alestes, Labeo, Bagrus, Citharinus, Hydrocynus, Hyperopisus* and *Protopterus* are some representatives from Ghibe-Omo, Baro-Akabo, and Abay Basins (Melak and Getahun, 2012). The highland east African forms are found in the northern rift valley lakes (Hawassa, Ziway, Langano, and the high land lakes (Tana and Haiq). *Barbus, Clarias, Garra, Oreochromis* and *Varicosrinus* are the common genera of fish common to this area. They are related to fishes of eastern and southern African and Arabian Peninsula (Skelton et al., 1991). The fishes of high mountain torrential streams largely belong to *Cyprinidae* adapted to flowering flood waters that occur seasonally. Two genera of fishes (Barbus and Garra) are dominant in this stream (Getahun, 2002; Mengesha, 2015).

In Ethiopia, eating fish is a unique experience. As usual, it is consumed by hand with injera or bread and a hot sauce to dip into. The grilled fins are very delicious since, bear a few bones. Fish tibs are chunks of fish marinated in lime juice and chilli spices, followed by the addition of shredded ginger and garlic and sesame oil, olive oil, and paprika (CNN travel, 2023). Those practices could expose the community for public hazard as some of those parasites have zoonotic risk.

Fish production is challenged by variety problems from which fish disease is the dominant. In Ethiopia, fish disease particularly those caused by parasites and bacteria are concern with studies indicating high prevalence of parasites in commercially important fish species in lakes like Ziway, Hawassa, and Tana (Gebremedhn and Tsegay, 2017). Parasitic species can be found everywhere and on every living organism. The rate of parasitic infection is high among vertebrates residing in aquatic environment (Chai et al., 2005). Their presence in their host is generally at equilibrium in aquatic organisms and the most common lifestyle on the planet (Marcogliese, 2005, Tesfay et al, 2023, Chew et al, 2024).

More than one-third of all species on Earth are parasites, and they contribute significantly to ecosystem biomass. Due to this host-parasite interaction is one of the most prevalent biotic relationships in nature (Hasegawa et al., 2025). Parasites affect fish health through mechanical, physical and reproductive damage. These changes can reduce growth, fecundity and survival, change behavioural and sexual characteristics, and result in many other maladaptive alterations of the infected host (Hailu and Mitiku, 2021). Parasites are one of the main threats facing the fisheries industry. This could be from economic losses due to fish rejection at markets, mortality in aquaculture, impact on fish health, or zoonotic concerns (Chai et al., 2005, Ziarati et al, 2022). In terms of host biology, population dynamics, and ecosystem health, parasites play a significant role. Any fish species, as well as any aquatic culture system, can contain them (Gulelat *et al*., 2013). The most common parasitic agents of fish are trematodes (fluke, e.g., *Opisthorchis spp*.), nematodes (round worm, e.g., *Anisakis spp*.), and cestodes (tapeworm, e.g., *Diphyllobothrium spp*.). Furthermore, protozoan organisms like *Cryptosporidium* species are regarded as zoonotic infections originating from fish (Ziarati et al, 2022). If left unmanaged, these illnesses can cause massive fish deaths, limit fish productivity, and in some situations, can spread infections to humans and other animals who consume diseased or infected fish (Kiprono, 2017). In addition, arthropods and crustaceans' species are known to infect fish (Janda and Abbott, 2010). Different species of leeches are also known to infect fishes (Kaygorodova and Sorokovikova, 2014, Tesfay et al, 2023).

Studies have indicated that biotic factors such as sex, body length and height influence the burden of parasitism. Accordingly, female had significantly higher prevalence and mean intensity than males, longer fishes have higher prevalence and mean intensity. Additionally, females may have compromised immune system specially during reproductive season (Renner and Duggan., 2024).

Fish is a good host for parasite multiplication that can be acquired by animals and humans through the ingestion of raw or inadequately cooked fish containing the larvae of the parasite (Kelsey., 1996). Fish harbor parasite as the intermediate or reservoir hosts for larval stages and as definitive hosts in which sexual maturity is attained for others. The parasite larvae for example anisakid nematodes, trematodes or others can be ingested by humans with under-cooked fish flesh which

then develop to morphologically mature but sexually immature worms thus, showing zoonotic potential (Kaba et al, 2024).

Knowledge about parasites of fish has been progressed due to their impact on growth and behaviour of fish and economic losses in recent years. Parasites cause an economic threat to the market value of fish, through consumer attitudes towards the presence of these parasites within food products (Thakkar, 2023). Important determinants of parasite exposure include the distribution of infected hosts, host immune status, and the spatial and temporal distribution of infective stage and intermediate hosts, and this can be influenced by the host's diet or trophic position in a food chain (Valtonen *et al*., 2010). These elements slow down growth and weight gain, impact fish production yield, spread infections among humans and animals, delay in fish sexual maturation, and raise in fish mortality rates. Additionally, by posing marketing issues for commercially harvested species, these cause the nutritional devaluation of fish and resultant economic losses (Gebreegziabher *et al*., 2020). Although these parasites are an important part of biodiversity and can help maintain stability in natural population (Shafiq et al., 2023) they can also lead to extinction often interacting with other stressors such as habitat loss (Brian, 2023). The extinction can be population level or species level.

Moreover, in developing countries like Ethiopia, people known little about fish borne zoonotic disease such as trematodiasis and anisakiasis (Kaba et al, 2024). Yet these parasitic infections are commonly reported from different freshwater body fishes (Tasfay et al., 2023) and the cause of significant human infections worldwide (Chai et al., 2005). To date, fewer studies have been conducted on fish parasites in Ethiopia, but they did not quantify the prevalence of parasites across different fish species, body weight, fish lengths and anatomical position in relation to parasite infestation. The current study hypothesised that commercially important fish species from lake Hawassa are infected by internal and external parasites. Hence, the present study aimed at estimating the prevalence of internal and external parasites, calculating the intensity, abundance of *O. niloticus*, *C. gariepinus*, and *B. intermedius* in Lake Hawassa. We have also examined how host factors are related to the parasite prevalence or its burden.

2. MATERIALS AND METHODS

2.1. Description of the Study Area

Lake Hawassa is one of the of the eight lakes in the Rift valley of Ethiopia. It is Located in the Sidama region, bordering the eastern side of Hawassa city, which is located 275 Km south of Addis Ababa. Geographically, the lake lies between 6°33'–7°33' N and 38°22'–38°29' E at an altitude of 1680 meter above sea level. The Lake stretches 16 Km from the Northeast to Southwest direction and extends 8Km from northwest to southeast direction having an approximate water volume of 1.3-billion-meter cube (45.9 billion feet^3). The maximum depth of the Lake is 21.6m (70.9 feet) with a mean depth of 11m. The catchment has a total area of 1455 Km^2 of which 93.6 Km^2 is the surface area of the Lake that may increase up to 99.3 Km^2 in the rainy season. The annual net groundwater outflow from Lake Hawassa to adjacent basins is estimated at 58 ×106 m^3 (Hundie *et al*., 2021).

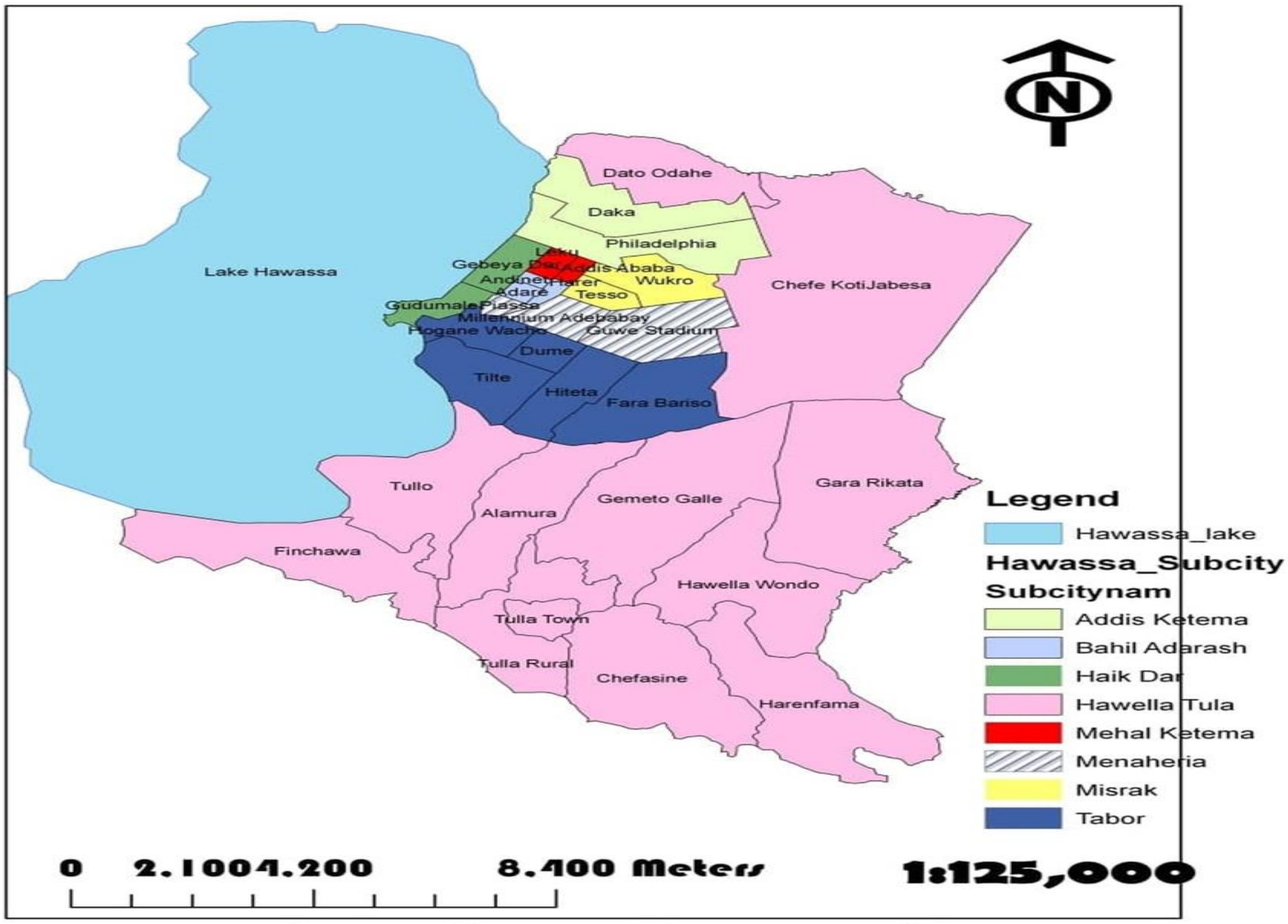

Figure 1: Map of Hawassa city, taken from Paulos et al, 2022

2.2. Study Design and sampling technique

A cross-sectional study was conducted from October 2023 to July 2024 on the fish community at Lake Hawassa to identify the prevalence of major internal and external parasites infesting fishes supported by antemortem and postmortem inspection on fish. The systematic random sampling technique was used to select fishes for parasites identification. The assessment was conducted in all for over 10 months.

2.3. Study Population

The fish community so far reported that inhabits Lake Hawassa were Nile tilapia (*Oreochromis niloticus*), the African catfish (*Clarias gariepinus*), the African big barb *(Labeobarbus intermedius).* The other small sized fishes found in the lake are the straight fin barb (*Barbus paludinosis),* the black lampeye *(Aplocheilichthys antinorii*) and the stone lapping minnow *(Gara quadrimaculata)* (Dadebo, 2000). *While O. niloticus, C. gariepinus* and *L. intermedius* are the commercially important species in the country; the other three minnow fish species are not fished due to their small size. *Oreochromis niloticus* constitutes about 90% of the total production, while *C. gariepinus* and *L. intermedius* contribute approximately about 7-8% and 2-3%, respectively (LFDP, 1996).

2.4. Sample Size Determination

The sample size was determined according to Thrusfield, (2005) at 95% confidence interval with a 5% marginal error. Accordingly, Zekarias and Yimer (2007) reported prevalence of (79%) of fish parasites from Lake Hawassa. We have applied collective sample size for all the fishes sampled using the formula $n = z^2 \times p\ (1-p)/d^2$, where n= sample size, z = z statistic for a level of confidence (z =1.96 at 95% CI), p = prevalence (p = 0.79), d = precision (if 5%, d= 0.05), the sample size 265 is included in the current study.

2.5. Methods of data collection

2.5.1. Description of the sampled fish

During the sampling, we have randomly picked those fishes from different sampling points. The species, sex, possible anatomical region of the parasite attachment, body weight, and total and standard lengths of individual fish were initially assessed and recorded. The sexes of each fish were determined after dissection and noting the presence of testes or ovaries (Imam and Dewu, 2010). Up on arrival at the Hawassa University, the total length was measured by centimetres using measuring tape and the weight measured using digital balance. Accordingly, the length of fish was categorized into three, for *C. gariepinus* from 28–50 cm as class I, from 51–75 cm as class II and >75 cm as class III; for *O. niloticus,* from 10–16 cm as class I, 17–25 cm as class II and >25 cm as class III; for *B. intermedius*, from 15–20 cm as class I, 21–26 cm as class II and >26 cm as class III. Their weight was also categorized into three classes, for *C. gariepinus* 190–400 g as class I, 401–2,000 g as class II and >2,000 g as class III; for *O. niloticus*, from 100–300 g as class I, 301–500 g as class II and >500 g as class III; for *B. intermedius,* from 100–200 g as class I, 201–460 g as class II and >460 g as class III (Tesfaye *et al*., 2020). Classifying fish species based on size and body weight during the parasite studies is crucial because this factor can influence parasite infection patterns, host vulnerability and the overall dynamics of parasite host interaction impacting both fish health and environmental ecosystem (Lima et al., 2012).

2.5.2. Antemortem and postmortem identification of parasite

A total of 265 fish were first examined alive (skin and gill scraping and picking with forceps followed by freshly dissected samples). The randomly selected fish were immediately transported in an icebox to the Parasitology unit of Hawassa University, for parasitological examination.

Each sampled fish was externally visualized, palpated, and examined for any ectoparasites on the entire fish skin, the fins, and opercular cavity including the gills. The external parasite was harvested to physiological salt solution from fish body and the fresh water (Kubitza, 2016). Scraping from fish skin were taken and smeared on to a clean microscope slide or hand lens. The sample was then examined under compound microscope at 10X, 40X and 100 X magnifications. Then postmortem examination was done using appropriate postmortem kits and standard evisceration technique (Zhokhov *et al*., 2007; Klimpel et al., 2019) and all body cavities was observed for presence of any encysted or free parasite larvae. The gill filaments, stomach, intestine, liver, heart, gall bladder, kidney and gonads were separately taken out and placed on clean Petridishes that contain 10millitre of physiological solution made of (0.9% sodium chloride w/v) (Younis et al., 2017). The head of *C. gariepinus* was dissected longitudinally and the cranial cavity

was washed away into a petridish using a water dropper and checked for parasites especially *Diplostomum* species. To clear their cuticle, the parasite was placed on in the 99% lactophenol for 24 hours and identified at genus level.

The fillet was digested in the pepsin HCl artificial digestion method is used to harvest any encysted larva in the fish muscle. Artificial digestion solution was prepared from 10gram of pepsin powder, 16mL of 25% HCl in 2 litres of tap water prewarmed at 42^0C in water bath. Briefly fish flesh was cut in to 1-2milimeter of small pieces using sharp knife. One-hundred-gram small piece of individual fish flesh was placed in 3liter beaker. The digestion solution was fully submerged the flesh; the beaker was covered with aluminium foil and heated on magnetic stirrer at 40^0C for 20 minutes to fully homogenise the tissue sample. We turned off the magnetic stirrer once observing the dis-appearance of any visible insoluble tissue. The sample was strained by laboratory sieve with approximate pore size of 70-100µm. The content retained on the sieve was examined for any larvae of nematode under stereomicroscope. The collected worms were counted, recoded and preserved in 70% ethanol (Paperna, 1996; Klinger and Floyd, 1998; Roberts, 2001; Roy, 2002; Amlacher, 2005; Pouder *et al*., 2005; Fraulo et al., 2014).

The morphological characteristics of anterior and posterior parts, including boring tooth, the ratio of ventricular appendix to intestinal caecum and the morphology of their tail were referred for characterization. The taxonomic identification of parasites was limited to genus level as the fish harbors mostly larval stages of many nematodes and trematode (Poulin and Presswell, 2022; Hoffman, 2019). The helminthic parasites were identified using parasitological and morphologically approach using standard identification keys and pictorial guide (Pouder et al., 2005).

2.5.3. Prevalence and intensity of the parasite

The prevalence and intensity of the parasite was calculated according to the formula (El-Shahawy et al., 2017).

Accordingly,

$$\text{Prevalence}= \frac{\text{Number of individual of a host infected with a particular parasite species}}{\text{Number of hosts examined}} \text{X100}$$

$$\text{Mean intensity}=\frac{\text{Sum of individuals of parasite species in a host saple}}{\text{Total of infected individuals of the host in the sample}}$$

$$\text{Mean abundance}=\frac{\text{number of collected parasite}}{\text{Total number examined fishes}}$$

2.6. Data Management and Analysis

All collected raw data was entered into a Microsoft Excel database system and imported to be analysed using SPSS statistical software (version-17). Accordingly, descriptive statistics including frequencies, percentages, mean intensity and abundant intensity were presented in tubular form. The chi-square test (χ^2) was used for a possible explanation of associations between the prevalence

of parasites and the selected variables (body weight, length, sex, and fish species) for the sampled fish. P-values less than 0.05 were taken as statistically significant.

3. RESULT

Prevalence of fish parasite in relation to sex, body weight, total and standard length

During the sampling period, 265 fishes belonging to *O. niloticus* (tilapia), *B. intermedius ('nech asa'*) also known as *Labeobarbus intermedius*, *C. gariepinus* (African catfish) were sampled (Figure 2A, 2B, 2C). The total length of the fish ranges from 10cm to 80cm and the total weight ranges from 100g-2Kg.

Prevalence, mean abundance and mean intensity of parasites in fish

Of a total of 265 examined fish, 234 (88.30%) were found infested by at least one parasite group (Figure 3-9). The highest level of parasite infestation was reported in *C. gariepinus* (93.10%) followed by *B. intermedius* (86.05%) and *O. niloticus* (85.93%). The minimum and maximum mean intensity of 5.37 and 8.08 is recorded in *C. gariepinus* and *B. intermedius* respectively. The lower and higher mean abundance of the parasite is also recorded in *C. gariepinus* and *B. intermedius* respectively. The variations in parasite infestations by fish species were not statistically significant ($\chi2 = 2.893$; df = 2, $P>0.05$) (Table 1).

Prevalence, mean abundance and mean intensity of parasites in fish

Table 1: Prevalence of parasitic infestation with respect to fish species studied

Species of fish studied	No. of fish affected	Number of parasites from each fish	Total parasite collected	Prevalence (%)	Mean intensity	Mean abundance	**χ2**	**P value**
O. niloticus (n=135)	116	6-9	788	85.93	6.79	5.84	2.89	0.24
C. gariepinus (n=87)	81	4-6	642	93.10	7.92	7.37		
B. intermedius (43)	37	5-10	299	86.05	8.59	7.40		
**Overall(N=265)**	**234**	**4-10**	**1748**	**88.30**	**7.77**	**6.87**		

The result indicated that 56.3% of male and 43.7% of female in *O. niloticus*, 52.87% male and 47.3% female in *C. gariepinus*, and 55.81% male and 47.13% female in *B. intermedius* were affected by one or more parasites. The smallest body weighted fishes are highly affected by one or more parasites (90.37% in 100-300g, 75.86% in 190-400g and 79.09% in 100-200g body weight in *O. niloticus*, *C. gariepinus*, and *B. intermedius*), respectively. This body weight class is the lower body weight in respective of fish species. Conversely, the parasite infestation increases as body length of the fish host increase. Accordingly, 70.37%, 95.40% and 93.02% of *O. niloticus*, *C. gariepinus*, and *B. intermedius* respectively are infected by fish parasites at the larger standard body length. The distribution of parasites with sex, body weight, and the total and standard length of *O. niloticus*, *C. gariepinus* and *B. intermedius* fish showed not statistically significant association (Table 2).

Table 2: Parasitic infestation in relation to sex, bodyweight, total and standard length of fishes

Species of Fish studied	Variable category		Number of examined	Number of fishes infected by parasites	Prevalence (%)	χ2	P-value
O. niloticus (n=116)	Sex	Male	76 (56.3)	64	84.21	0.423	0.515
		Female	59 (43.7	52	88.14		
	Body Weight (gram)	100-300	122 (90.37)	105	86.07	2.382	0.304
		301-500	11 (8.15)	10	90.91		
		>500	2 (1.48)	1	50.00		
	Total Length (in which measurement?)	10-16	32 (23.70)	27	84.38	0.289	0.865
		17-25	92 (68.15)	79	85.87		
		>25	11 (8.15)	10	90.91		
	Standard Length	<10	4 (2.96)	3	75.00	0.757	0.685
		10-15	36 (26.67)	30	83.33		
		>15	95 (70.37)	83	87.37		
C. gariepinus (n=81)	Sex	Male	46 (52.87)	42	91.30	0.493	0.483
		Female	41 (47.13)	39	95.12		
	Body Weight	190-400	66 (75.86)	62	93.94	0.609	0.737
		401-2000	19 (21.84)	17	89.47		
		>2000	2 (2.29)	2	100.00		
	Total Length	28-50	85 (97.70)	80	94.12	5.923	0.052
		51-75	2 (2.29)	1	50.00		
		>75	0 (0.00)	0	0.00		
	Standard Length	<25	4 (4.59)	3	75.00	2.140	0.343
		25-70	0 (0.00)	0	0.00		
		>70	83 (95.40)	78	93.98		
B. intermedius (37)	Sex	Male	24 (55.81)	21	87.50	0.096	0.757
		Female	19 (44.19)	16	84.20		
	Body Weight	100-200	34 (79.07)	31	91.18	4.155	0.125
		201-460	7 (16.28)	5	71.43		
		>460	2 (4.65)	1	50.00		
	Total Length	15-20	3 (6.98)	2	66.67	1.027	0.598
		21-26	31 (72.09)	27	87.09		
		>26	9(20.93)	8	88.89		
	Standard Length	<10	1 (2.33)	1	100.00	2.397	0.302
		10-20	2 (4.65)	1	50.00		
		>20	40 (93.02)	35	87.50		

Prevalence of parasitic infestations with respect to anatomical locations

Clinostomum species (figure 3 in the annex 1) were the dominant trematodes in *O. niloticus* with a prevalence of 65.19% infecting brachial cavity, pericardial cavity, premium and ocular cavity, followed by *Euclinostomum* species (figure 4 in the annex1) with 34.07% which infects kidney,

liver and brachial cavity (figure 3). *Dactylogyrus* species (figure 5 in the annex 1) from the gill and *Diplostomum* species (figure 6) from the brain tissue also showed higher prevalence, accounting 28.15% and 24.44%, respectively. *Contacaecum* species (figure 7) one of the zoonotic anisakidae infecting 26.67% of *O. niloticus* species (Table 3). *Digenean encysted metacercaria* 68.97%, *Contacaecum* species 50.57% and *Diplostomum species* 47.13% infecting musculature, mesentery and brain tissues respectively are the dominant helminths infecting *C. gariepinus* groups. This prevalence report is calculated considering multiple parasite infestation from single host i.e, one fish could be infected by different parasite groups or free from any parasite. *Contracecum* species (37.21%) and *Dactylogyrus* species (62.79%) are the dominant parasites affecting *B. intermedius* infecting mesentery and gills respectively. The common ectoparasites infecting fish species in Lake Hawassa are Argulus species (figure 8) and *Dolops* species (figure 9 ‘a’ and ‘b’ in annex 1) (Table 3).

Table 3: Prevalence with respect to the recovered parasites (n=number fishes sampled in each group).

Variables	Site of localization	Parasite species reco vered	Number of infected fish	Prevalence (%)
O. niloticus (n=135)	*Clinostomum* species	Branchial cavity, pericardial cavity, between cranium and pharyngeal teeth, ocular cavity	88	65.19
	Euclinostomum species	Kidney, liver, branchial cavity	46	34.07
	Contracaecum species	Heart, pericardial Cavity	36	26.67
	Diplostomum species	Brain tissue	33	24.44
	Dactylogyrus species	Gill	38	28.15
	Myxobolus species	Intestinal contents	20	14.81
	Leeches	Skin, gills, mouth	16	11.85
	Argulus species	Skin, fins	9	6.67
	Dolops species	Gill, fins	5	3.70
C. gariepinus (n=87)	*Digenean encysted metacercaria*	Musculature	60	68.97
	Contracaecum species	Mesentery	44	50.57
	Diplostomum species	Brain tissue	41	47.13
	Unidentified parasite	Mesentery	1	1.15
	Dactylogyrus species	Gill	41	47.13
	Encysted cestode larvae	Stomach	5	5.75
	Leeches	Skin, gills, mouth	7	8.04
	Argulus species	Skin, gill, fins and oral cavity	18	20.69
	Dolops species	Skin, gill, fins,	38	43.68

		Oral cavity		
B. intermedius (n=43)	*Contracaecum* species	Mesentery	16	37.21
	Unidentified parasite	Mesentery	2	4.65
	Dactylogyrus species	Gill	27	62.79
	Leeches	Skin, gills, mouth	5	11.63
	Argulus species	Skin, fins	3	6.98
	Dolops species	Skin, fins	4	9.30

Distribution, location and number of ectoparasites on the fishes sampled.

Contracaecum species is the most common parasites found on *O. niloticus with* 373 total number of parasite collected from 224 infected fishes with mean intensity of 3 parasites per fish. Different parasites have different affinity for the body of the fishes where the external parasites mostly affecting gills, skin and gills. *Leeches* are also found infecting all the three fishes studied (Table 4).

Table 4: Anatomical distribution of the different parasites

**Type of parasite**	**Parasite group**	**Anatomical location of infection**	**Number of fish infected**	**Number of parasites collected**
Clinostomum species	Trematode	Branchial cavity, pericardial cavity, between cranium and pharyngeal teeth, ocular cavity	88	255
Euclinostomum species	Trematode	Kidney, liver, branchial cavity	46	136
Contracaecum species	Nematode	Heart, pericardial cavity	124	373
Diplostomum species	Trematode	Brain tissue	74	200
Dactylogyrus species	Trematode	Gill	106	276
Myxobolus species	Protozoa	Intestinal contents	20	88
Digenean encysted metacercaria	Trematode	Musculature	60	189
Encysted cestode larvae	Cestode	Stomach	5	19
Argulus species	Crustaceans	Skin, fins	30	79
Dolops species	Crustaceans	Gill, fins	47	96
Leeches	Hurindae	Skin, gills, mouth	28	34
Unidentified internal parasite		Mesentery	1	3
**Total collected parasites**				**1748**

Discussions

The commercially important fish species: O. *niloticus, C. gariepinus,* and *B. intermedius* from the Hawassa lake are known to be infected with parasites from nematodes, cestodes, trematodes, crustaceans, protozoan families and leeches (table 1-4, figure 3-8). Some of those parasites are also pathogenic to humans and animals. Some of these parasites are known zoonotic pathogens to humans (Ziarati et al., 2022). The majority of animal parasites are found in fish and even apparently health wild fish have some parasites burdens (Petty et al., 2022). Various disease and parasites are common and the major limitation to the development and sustainably of aquaculture (Shafiq et al., 2023). According to reports, fish endoparaites from Lake Awasa, Lake Lugo, and Lake Tana are common in Ethiopia (Gebremedhin et al., 2020). Prior research reports from other world indicated that parasites, which cause castrations and restrict the growth of their hosts (de Montaudouin et al., 2012), have an ecologically significant role in marine environments (Marcogliese, 2004; Poulin et al., 2014; Sures and Nachev, 2022). Parasitic disease are the common infections in aquatic environment accounting 54% followed by bacterial infections which accounts only 22% (Okon et al., 2024).

The current prevalence of fish parasites was found to be 88.30%, differing with fish species, sex, body weight, standard length. This result agreed with Mitiku, (2017) who indicated 83.4% prevalence of parasites from central Ethiopia (which lake? As it is your center of discussion). Similar prevalence report of 77.4% to 88.3% was reported (Outa et al., 2021) inVictoria Lake (Outa et al., 2021), Kenya, 88.2%-91.2% by Mgbemena et al., (2020) from Nigeria. The current finding is found greater than earlier reports of 79% by Zekarias and Yimer, (2007) at Lake Hawassa, 69.25% by Tilahun and Behonegn, (2017) at Lake Ziway, 66.3% by Gulelat *et al*., (2013) at Koka reservoir. However, it is found significantly greater than finding by Tesfaye *et al*., (2017) who reported 37.6% at Lake Hashange, 34.8% by Kiprono, (2017) at Lake Baringo, 30.9% by Gebreegziabher *et al*., (2020) in Jimma, 21.4% by Kaba et el., (2024) in *O. niloticus* and *L. niloticus* from Chamo lake and 20.8% by Bekele and Hussien, (2015) in Lake Ziway. Lower prevalence rate as compared to the current finding of 52.3% was reported by Ageng'o et al., (2025) at farms and lake in Taita Taveta county of Kenya and 37.5% at Kiri reservoir of Nigeria (Vandi and Daniel, 2023). Furthermore, higher prevalence reports of 93% was reported from Midmar reservoir, Adwa, Northen Ethiopia by Tesfay et al, (2024) in *O. niloticus*.

The prevalence, mean intensity and mean abundance of the parasites varies with species of the fish's host. Accordingly, prevalence rate of 85.93%, 93.10% and 86.05% is registered in O. *niloticus, C. gariepinus,* and *B. intermedius* respectively. In the current finding, the prevalence rate is slightly higher in *C. gariepinus* as compared to O. *niloticus* and *B. intermedius*. Similar finding was reported by Tesfaye *et al,* (2020) in which the rate of parasitic infection is higher in *C. gariepinus*. Additionally, *C. gariepinus* species are among the most frequently infected fish species, according to Gulilat et al, (2013). Trute et al., (2023) also reported the presence of over 107 metazooan parasites are known to infect *C. gariepinus* in Africa with well characterized life cycle and general morphology.

The mean intensity and apparent density of parasites also vary with different fish species. Accordingly, 8.59, 7.92, and 6.79 and 7.40, 7.37 and 5.84 was reported in *B. intermedius, C. gariepinus* and O. *niloticus* respectively. Similarly, Oztruk and Alutnel, (2006) discovered that the parasite's mean intensity level and prevalence changed depending on the size of the host fish.

Abiotic variables like water temperature and host-related variables like behaviour, sex, age, resistance, and mortality are important determinants of the seasonal prevalence and mean intensity of parasites (Bakke et al., 2002, Ozer et al., 2004, Khidr et al., 2012).

The higher prevalence of internal helminths in the current study could be attributed to a number of factors, including the lack of an effective waste disposal and management system, which led to the processing of post-harvest fish and the disposal of the waste (scraps and gastrointestinal contents) into the lake and its shorelines; environmental pollution from detergents used for washing clothes on the lake shore; and some birds that could serve as intermediate or final hosts for helminths. This shows that the host-parasite interaction and changes in abiotic factors, including sewage, irrigation, sanitation, and flooding, frequently causes the distribution of fish parasites to vary from one habitat to another, and that leads to environmental stresses that impair the host's immune system (Tibebe et al, 2023). Compared to mammals, fish have a lower immune response to foreign proteins (Pradipta et al, 2012; Ardelli and Woo, 1997). Several parasites are known to alter the behaviour of their hosts, predisposing them to predation (Labaude et al, 2015). The difference in prevalence from different lakes in Ethiopia and other parts of the world may be attributed to the difference in behaviour of different fish species, the quality of water, the presence of other stressors such as pollution and climate change (Okon et al., 2024). The increase in global temperature leads to fluctuation in aquatic environment whereas the elevated water temperature could potentially compromise the fish immune system leading to fish more vulnerable to disease conditions (Grossart et al., 2019; Kaur et al., 2022).

In the current study the prevalence of the parasite is relatively higher in male fishes (56.3%, 52.87%, 55.81%) than female (43.7%, 47.13%, 44.19%) in *O. niloticus, C. gariepinus* and *B. intermedius* respectively. This finding is lower than the prevalence report of 81.8% in male and 93% in female by (Mgbemena et al., 2020) from Nigeria. However, it is significantly higher to finding of Tesfaye et al., (2017) who reported 12.8% in male and 24.8% in female from Lake Hashenge, Ethiopia. Similar prevalence report of 47.73% in male and 52.27% in female was reported by (Vandi and Daniel, 2023) from Nigeria and 60% in male 40% prevalence from female in upper Egypt (Abd-ELrahman et al., 2023). It is stated in other finding that female is more likely exposed to parasitic worms due to behaviours such as higher rate of shoaling which can lead more frequent and prolonged contact with parasites (Simková et al, 2005; Abd-ELrahman et al., 2023).

In the current finding the presence of parasites varies with body weight and standard length. Accordingly, the lower body weighted fishes are highly affected by one or more parasites. This finding is consistent with earlier finding that indicate there is a general trend that smaller fish or those with poor body condition may be susceptible to parasitic infections although it is not universally true. This can be due to several factors including inability to allocate enough defence and behavioural adaptations in lower body condition fishes (Abd-ELrahman et al., 2023; Hasegawa et al., 2025). How ever several biological parameter or ecological processes determine which types of parasites are more likely to correlate closely with host size (Poulin, 2005). In other terms larger fish usually have larger fish population and wider range of parasitic species due to the availability of resources and greater capacity to harbor variety of parasites. Smaller fish however may be more vulnerable to specific larval stages of parasites potentially due to their exposure to predation (Leung, 2022). Although parasite reduces host health, parasite infection can occur due to compromised host health (Hasegawa et al., 2025). Poulin, (2014) indicated host size (and age)

is generally known to be positively associated with parasite species richness and infection levels in host individuals. Nikolaev *et al*, (2006) also concluded that as age increase there is higher chance of accumulating parasites during their lifetime. In the current finding it is found difficult to harvest and investigate old age and large body weight fishes since illegal fishing along with other abiotic factors are highly practiced and may posing threat including decline in number of fish or yield of the lake Hawassa. The research team has clearly observed the illegal fishing practice and the practice of eviscerating fish along the shore of their lake which increase the risk of re-infection.

Identification of parasites with significant host and tissue specificity is made much easier by knowledge of fish hosts, while other parasites are detected due to their widespread presence and lack of host specificity (Petty et al., 2022). In line with this, *Contracaecum* was the most frequently encountered nematode parasite found in the current study. It is found 26.67% in *O. niloticus* to 50.57% in *C. gariepinus.* This finding is lower than previous reports by Zekarias and Yimer, (2007) in Lake Hawassa, who reported 39.67% in *O. niloticus*, 80.5% in *C. gariepinus*, and 36% in *B. intermedius*. Moreover Kaba et al., (2024) reported lower prevalence of 21.4% from *O. niloticus* and *Lates niloticus* from Chamo Lake, Arbaminch. However, it is higher as compared to Gulelat *et al.* (2013) at Koka reservoir, 24.82% in *C. gariepinus* and 16.49% in *B. intermedius*. This result suggests that the distribution of *Contracecum's* parasites can vary from one habitat to another due to host parasite relationships and changes in water quality parameters. Recently *Contracaecum bancrofti* a unique Australian species and warming the fish tissue leads to emergence of small *Contracaecum* larvae <1 mm from intestinal tissue (Shamsi *et al*, 2018). *Contracaecum* are economically important parasites with zoonotic significance where human infection through ingestion of infected tissue is the major route (Kaba et al., 2024). Adult *Contracaecum* spp. are found in the stomach of marine mammals or piscivorous birds, and larval stages infect a wide range of invertebrates and fish species (Shamsi et al, 2017). Several other studies have also reported human infection due to *Contracaecum* larvae following consumption of infected fish (Shamsi *et al*, 2018; Shamsi and Barton, 2023; Kaba et al., 2024).

The host distribution of *Clinostomum* species was discovered in the current study to be much higher than previous studies by Gulelat *et al.* (2013), Reshid *et al.* (2015), and Tesfaye *et al.* (2017), who revealed a prevalence of 27.39%, 18.8%, and 15.2% in Lake Abaya, Koka Reservoir, and Lake Hashange, respectively. However, the prevalence of this parasite is lower than that found by Zekarias and Yimer, (2007) who reported 75.7% prevalence from Lake Hawassa. The variations may result from variations in the physiochemical characteristics of water bodies and the intermediate hosts of the parasite. In other way another digenean fluke, *Euclinostomum* species from *O. niloticus* were detected in the kidney, liver, and branchial cavity. This finding was in line to previous reports from Ethiopian lakes and water bodies. Accordingly, 30% report was made by Zekarias and Yimer, (2007) from Lake Hawassa but significantly lower prevalence rate of 2.73% was reported by Gulelat *et al.* (2013) in Koka Reservoir and 3.9% by Gebreegziabher *et al.* (2020) in Jimma. However, Mitiku, (2017) recorded 49.35% of the *Euclinostomum*. This difference is mostly caused by the parasite and hosts life cycles and other environmental factors. Moreover, Khan et al, 2018 reported that prevalence of 70-92% metacercarial infection belonging to *C. complanatum* was reported concluding the seasonality of the infection. He stated that the high prevalence of infection of the parasite during summer may be because of the time of emergence of cercariae in high temperature from snail vector and the environment. Digenetic trematodes, or flukes, belonging to the genera *Clinostomum* and *Euclinostomum* infect many fish species all over

the world. These parasites are found in freshwater and saltwater habitats worldwide (Senapin et al., 2014).

Lower prevalence rate of *Diplostomum* species, 24.44 and 47.13% respectively was found in the brain tissue of *O. niloticus* and *C. gariepinus*. This finding is lower as compared to the previous studies 77.93% of the gill cavity of *O. niloticus* at Lake Baringo, Kenya (Kiprono, 2017)*;*83.3% from cranial cavity of *C. gariepinus* sampled at Tanzania (Mukama, 2008); 100% of the cranial cavity of *C. gariepinus* was from Lake Hawassa (Tadesse, 2009); 77.93% of the cranial cavity of *C. gariepinus* (Gulelat et al., 2013). Significantly lower prevalence rate of 1.03% *Diplostomum* species was reported from the eye of *B. intermedius* from Lake Abaya (Gulelat et al. (2013). According to the current findings, Degenian encysted metacercaria frequently infect *C. gariepinus* muscle. This finding is in line with the findings of Vidal-Martínez et al. (2012) and Galaktionov et al. (2006), who conclude that these parasites commonly affect fish in coastal marine systems, which exhibit complex life cycles that typically involve snails as first intermediate hosts, macroinvertebrates, such as bivalves and crustaceans, or vertebrates as second intermediate hosts, and fish, birds, or mammals as final hosts.

External parasites have strategic survival reasons to inhibit specific microhabitat on fish body. This specific microhabitat may provide availability of food, reproductive success, enhanced attachment or reduce the pressure of predator (Chew et al., 2024). in the current finding, the crustacean parasites *Argulus* and *Dolops* species were found infecting gills, fins, skin, and oral cavity of the three fish species. This finding is lower as compared to the previous study by Zekarias and Yimer, (2007) in Lake Hawassa, who found 5.33% and 8.33% in *O. niloticus*; 44.5% and 63.5% in *C. gariepinus*, and 6% and 17 in *B. intermedius*. Moreover, Mitiku (2017) reported 7.8% of external parasites from *O. niloticus* at Sebeta ponds. Argus species is more commonly known as fish lice, are member of large groups of branchiuran parasite that infest and cause fish disease. They are crustaceans and crabs, lobsters, and shrimp. There are over 100 species infecting freshwater fishes with *Argulus foliaceus, A. coregoni, A. japonicus* common in freshwater fishes (Stecker and Yanong, 2024). According to a study by Trutera et al, (2022) *A. africanus* and *A. rhipidiophorus* are prevalent crustacean parasites in Ethiopian fish, especially those found in Lake Hawassa. *Argulus japonicus* is an alien species that has been introduced globally and is known to damage fish such as *C. gariepinus*, while it is not specifically listed as being present in Ethiopian fishes (Zekarias and Yimer, 2008, Trutera et al, 2022).

Dactylogyrus species was find residing in the gills of fishes. The current prevalence report of 3.7-43.6% was found higher than the reported 3.96% by Mitiku *et al.* (2018) in *O. niloticus* from *Gilgel* Gibe I Dam, and 31% by Borji *et al.* (2012) in common carp in the northeast of Iran from the gill of sampled fish. *Myxobolus* species from the intestinal contents of *O. niloticus* were also found in the current study, which was not reported elsewhere in Ethiopia. How ever the prevalence rate of *Myxobolus* species in the current finding was lower than previous reports by Abdel-Baki *et al*. (2015) in the Nile River, Egypt, and Folefack *et al*. (2019) in Cameroon. Higher prevalence rate of *Myxobolus* infection including two new species was reported from Botswana (Reed et al, 2002).

In addition to the infections caused by parasites and other pathogens, several factors are threatening the quality of Hawassa lake from which human practice along the edge of the lake is the dominant factor. Memberu et al., (2021) have observed that the quality of the Hawassa lake is inadequate for maximum production of fishes and needs mitigation measures to control

eutrophication and pollution flow. Anthropogenic activities, illegal fishing practices such as the use of narrow mesh nets, a lack of community awareness regarding fishery management, and a lack of fish stocks are the main conservation issues facing Ethiopian fish and fisheries sector (Chimdo, 2022).

The presence of parasites in edible part of the fish represent a public health threat since the presence of those parasites are responsible for causing infection in humans with significant outcome (Audicana and Kennedy, 2008). Knowledge of how parasites such as *Contracaecum* species, flukes and other pathogenic parasites are transmitted, their reservoir animal, their specific reservoir animals, the specific pathogen involved, directly impacts prevention and control strategy (WHO, 2020). The focus of control methods should be on health education to promote appropriate control measures including, and safely disposing of infected (Fomda et al., 2015). As prevention and control methods for each pathogen varies, however several practices are recognised as effective for reducing the risks at the community and personal level. Safe and appropriate guidelines for animal care in the agricultural sector helps to reduce the potential for food borne zoonotic disease outbreaks through ingestion of animal food or some vegetables. Standards for clean drinking water and waste disposal as well as protection for surface water in the natural environment are important and effective control means (WHO, 2020).

Limitation of the study

This study has not stablished the characterisation of the parasite at species level, and it only focuses describing the parasite at genera level. Moreover, water the impact of water quality on the effect of parasites have also not established

4. CONCLUSION AND RECOMMENDATIONS

In the current study, internal and external infestations were the predominant cases infecting fish with 88.03%. The parasites recovered belongs to *crustaceans, monogenean, digeneans, nematode, cestode, and one protozoan and Hirudinea* having a direct effect on fish health and meat quality and, thus, a direct effect on fish production and the economy. Among these, the Dactylogyrus species was the dominant external parasite followed by Argulus and Dolops species. It is found that parasite intensity and abundance vary with the host fish species. Anatomical and physiological variables of the fish such as body size, age, body weight and gender also found determining different prevalences. Our finding also showed that the common zoonotic nematodes such as *Contracaecum* species and flukes are commonly found in the fishes at lake Hawassa necessitating coordinated efforts of public education. We found that human practice along the shore of the lake is also one of anthropogenic factor influencing fish health and productivity.

Based on the above conclusion the following recommendations were forwarded:

- To improve parasite management, it is necessary to investigate and determine how abiotic elements affect the parasite-host interaction.
- Lake Hawassa needs to be kept apart from areas utilised for fishing and pleasure purpose.
- It is necessary to investigate the diversity of parasites over a wide range of hosts and water bodies.

- The wastes products including offals of the fish themselves must be disposed of properly and kept apart from water bodies.
- Molecular detection of each fish parasite to the genetic level needs to be carried out in future as the geographic bias exists in the discovery and genetic characterization of novel parasites.

Limitation of the study

A histopathological diagnosis with greater funding and resources does not support this study. Furthermore, it is necessary to evaluate how the evaluation of water quality affects fish parasites. Another drawback is that parasites can only be identified at the genus level.

Ethical approval and consent to participate

All methods were carried out in "accordance" with relevant guidelines and regulations. Furthermore, all methods are reported in accordance with "ARRIVE" guidelines (https://arriveguidelines.org) for the reporting of animal experiments. Ethical approval was obtained from the Research Ethics Committee of the School of Veterinary Medicine, Wollega University dated 20/04/2023 with minute number SVM.RERC/0023 before starting data collection. Parent/legal guardian provided informed consent on behalf of participants of age below eighteen years whereas oral consent was received from those above eighteen-year fishermen. The Research Ethics Committee of the School of Veterinary Medicine, Wollega University approved all aspects of this study, including the informed oral consent process. Communicative letter was written to Hawassa City municipal and fishery sector leaders explaining the objective of this study. Data was collected after explaining the aims and methodology of the study. During data collection on fish all the welfare of animal was protected. It is assured that information collected was anonymous and that it was only for research purpose.

Data Availability statement: The data that support the findings of this study are available from the corresponding author upon reasonable request.

Conflict of interest: the author declares there is no conflict of interest

Funding importation: No funding was received for this research

Author's contribution: MA and DA collected data from the field, managed and analysed it, and participated in manuscript development and revision; GH, AM, YT, methodology, analysis, writing the original draft and editing final paper; DA, supervision, writing the original draft and editing final paper.

Acknowledgements: the author acknowledges Wollega University, Addis Ababa University and Hawassa University

Figures of fishes and their parasites (figure 2A-9B)

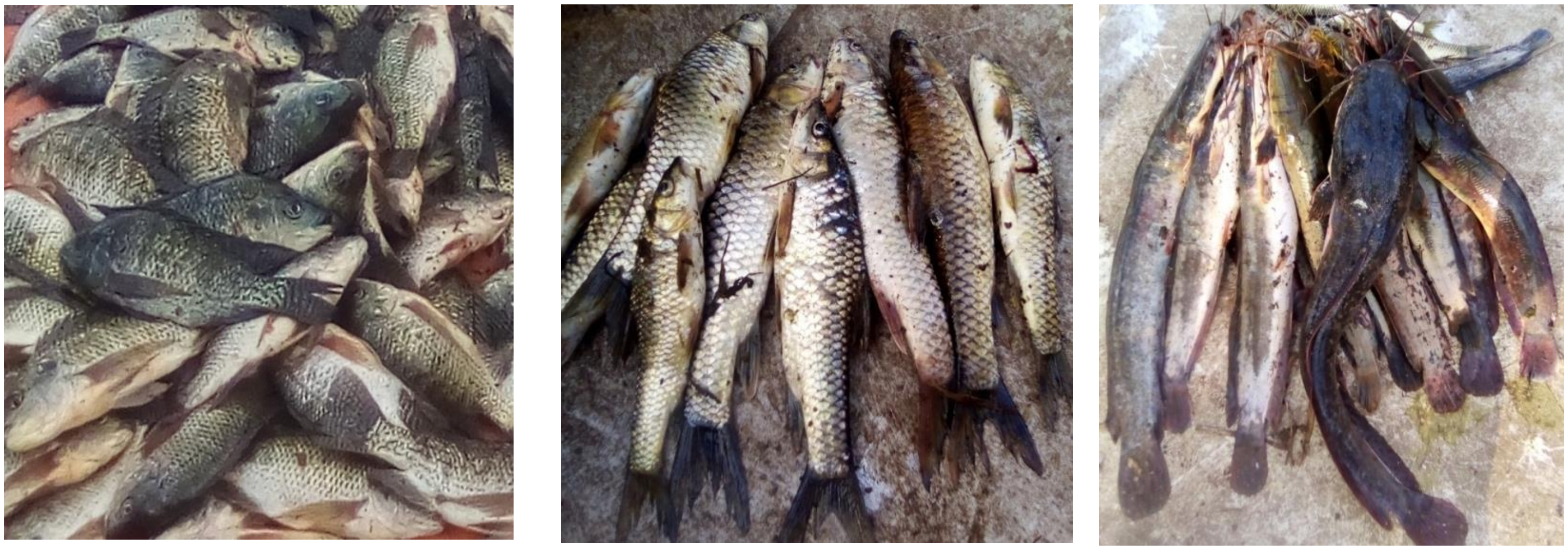

Figure 1: *O. niloticus (*Nile tilapia or Koroso) the first; *Barbus* species, the middle and *C. gariepinus (*Catfish or Ambaza), the third

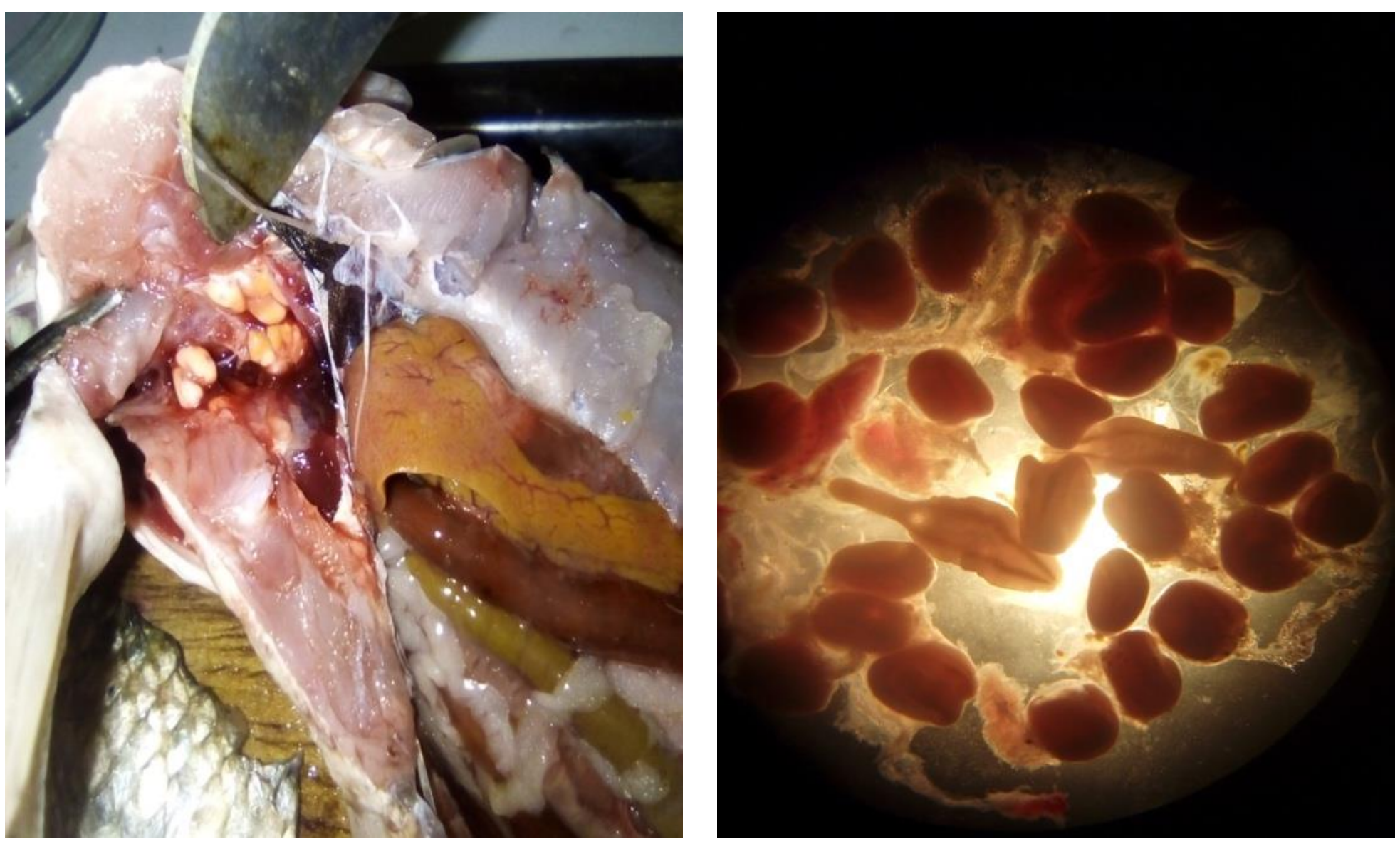

Figure 3: Encysted (left) and Excysted (right) *Clinostomum* species

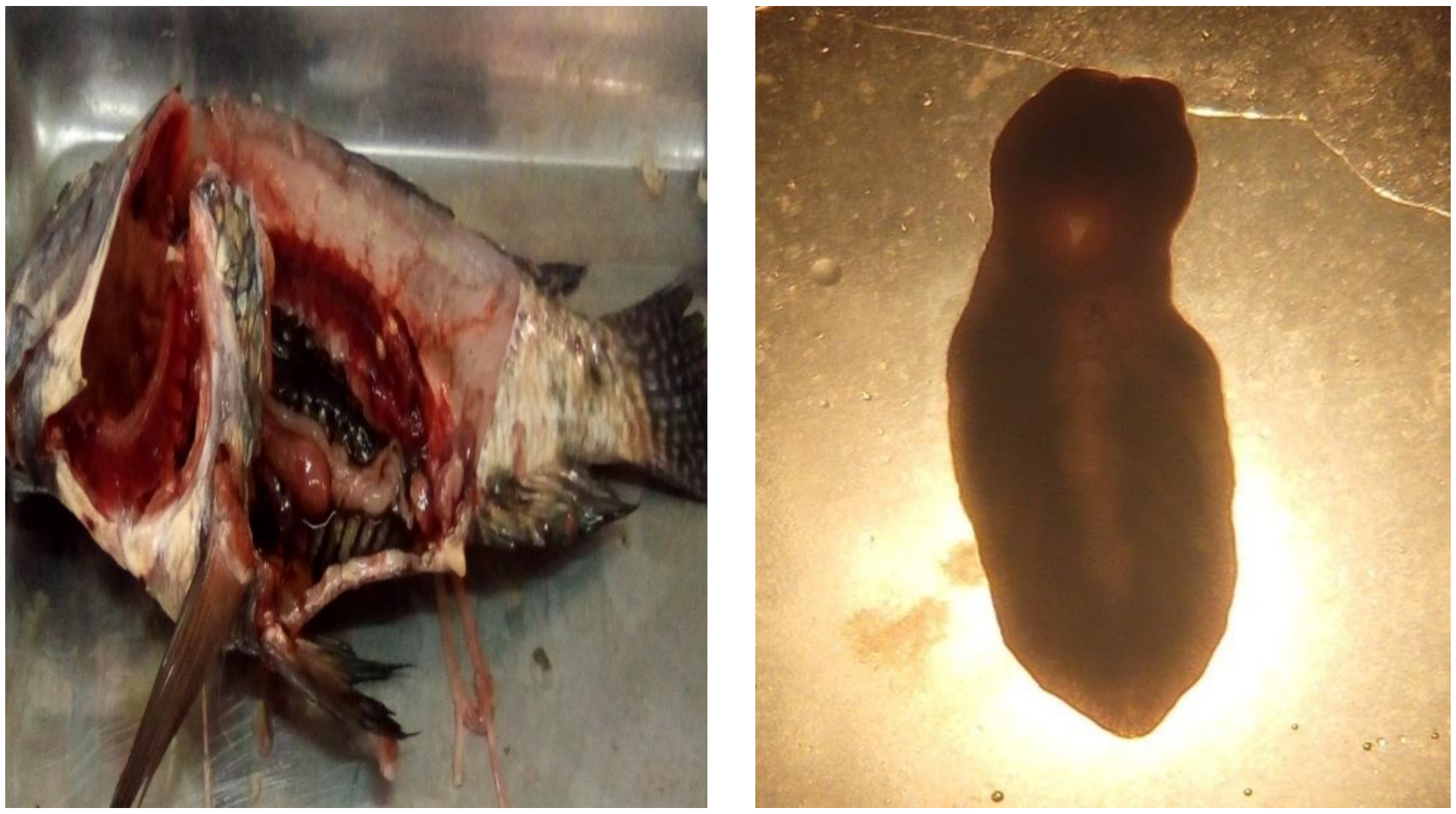

Figure4: Encysted (left) and excysted (right) *Euclinostomum* species on the kidney of *O. niloticus*

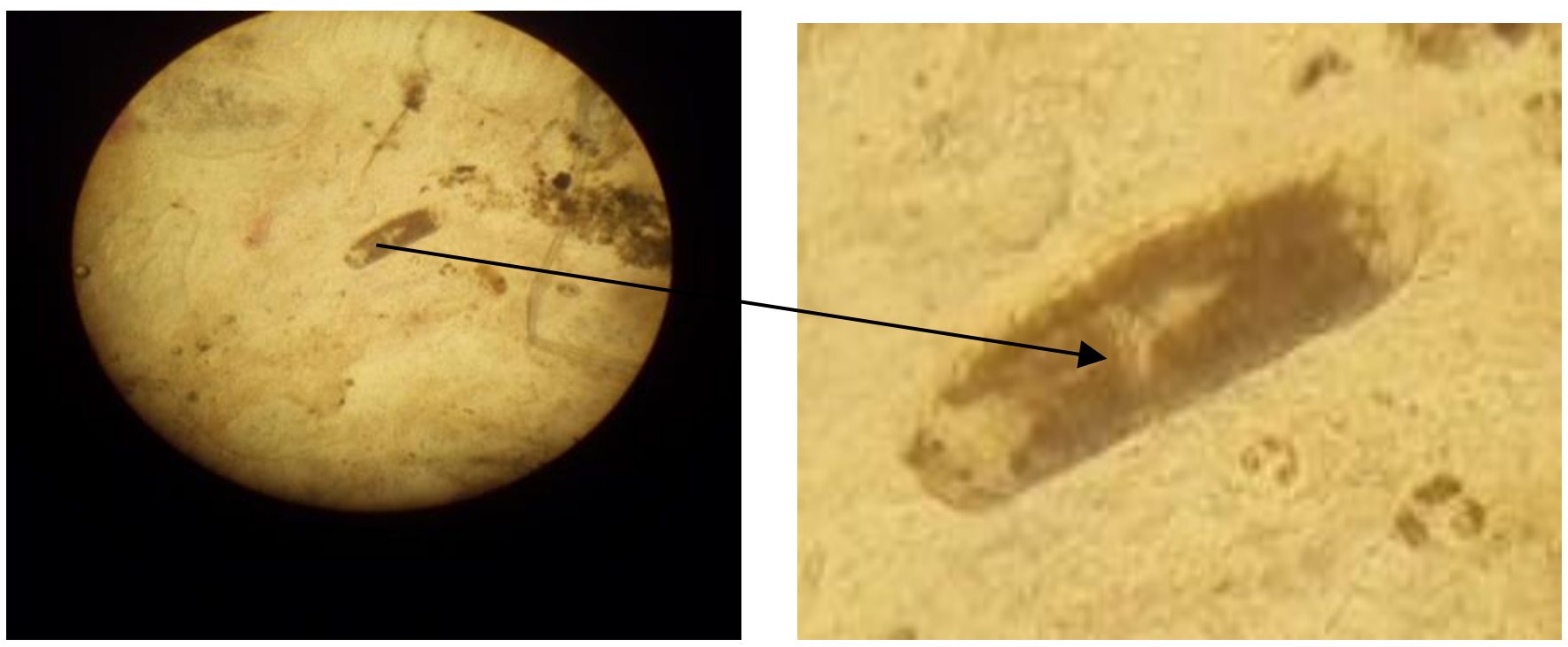

Figure 5: *Dactylogyrus* species from the gill of Nile tilapia *(O.niloticus)*.

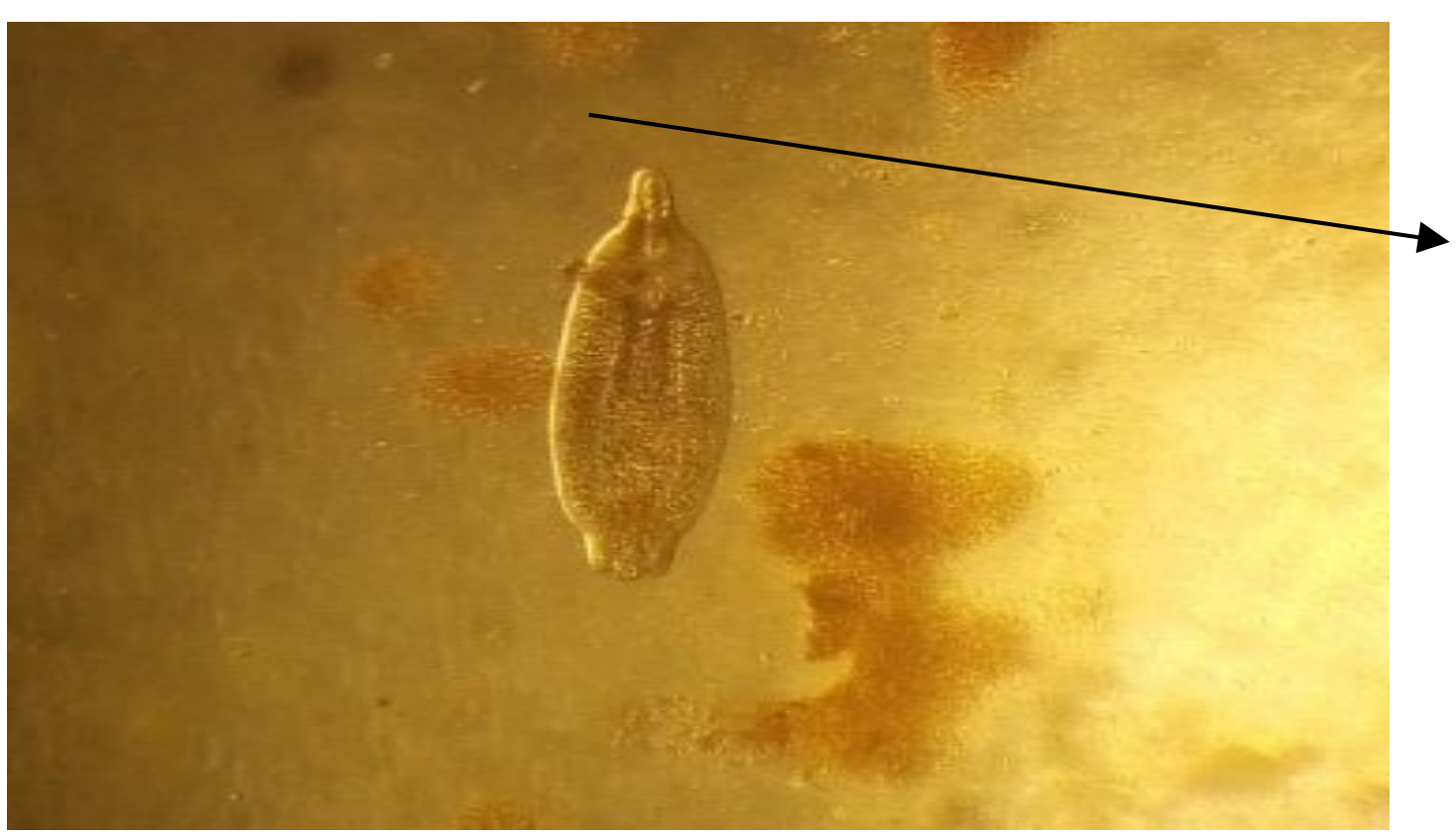

Figure6: *Diplostomum* species from the brain tissue of African catfish

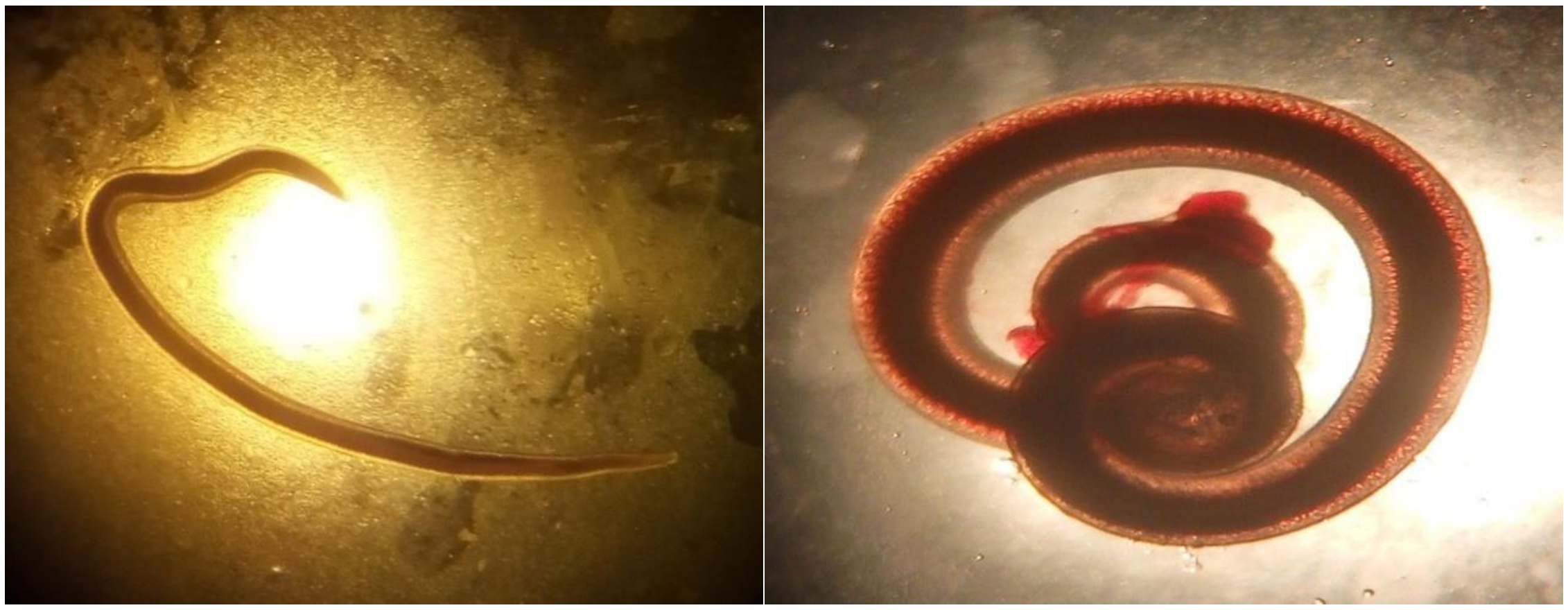

Figure 7a: *Contracaecum* species from the heart of Nile tilapia *(O. niloticus)*.

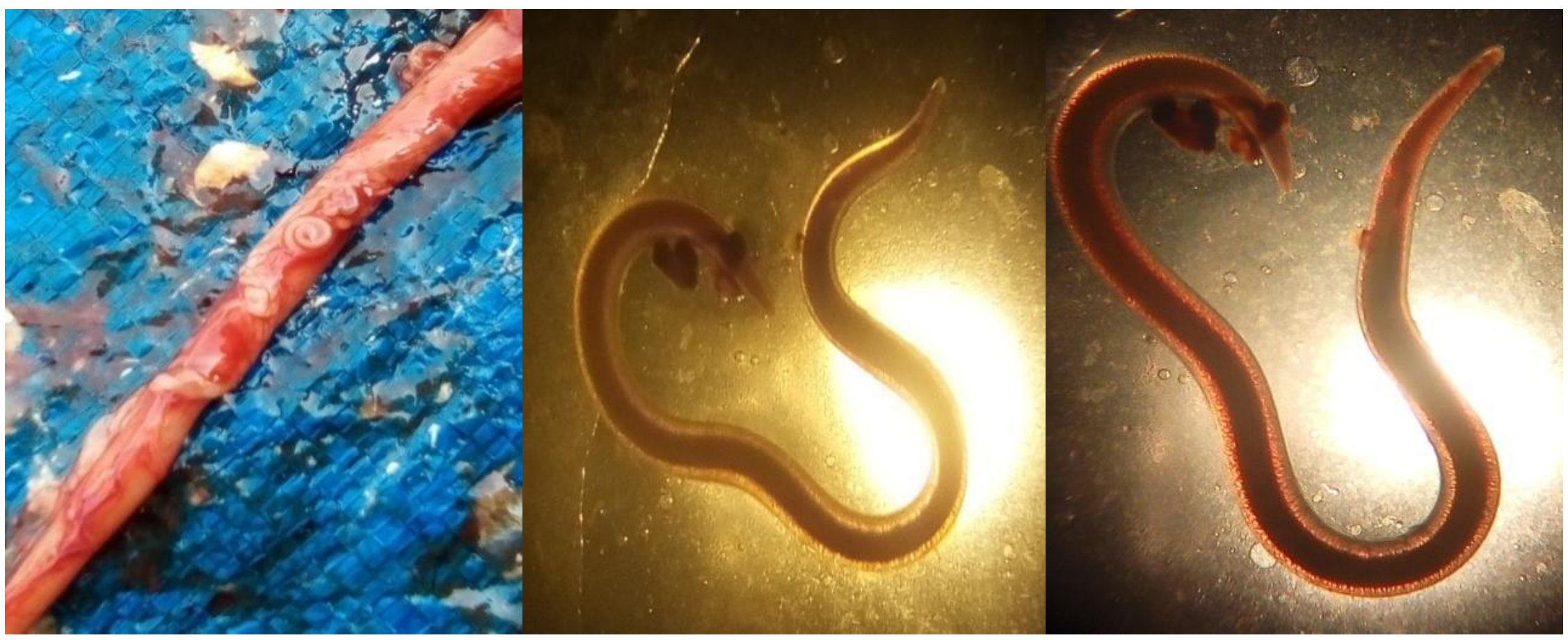

Figure 7b: *Contracaecum* species from mesentery of *C. gariepinus* (African catfish).

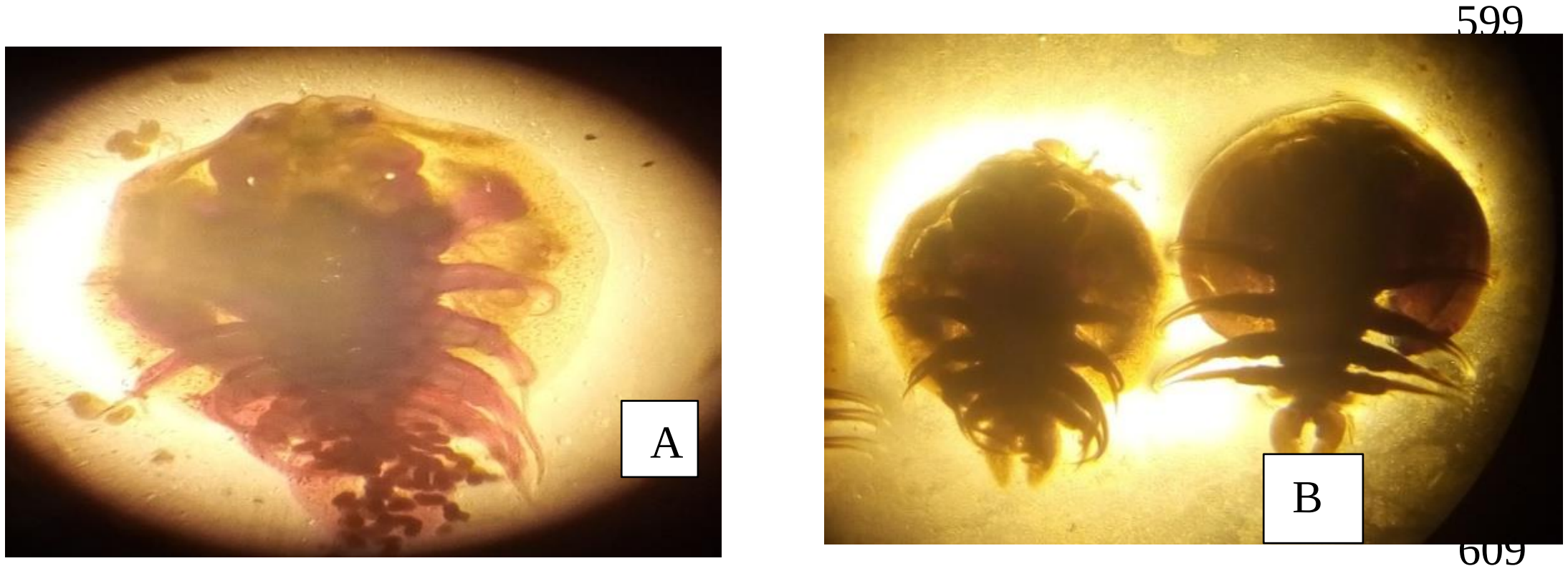

Figure 8: *Argulus* species (A: from the gill B: From fins of *C. gariepinus*)

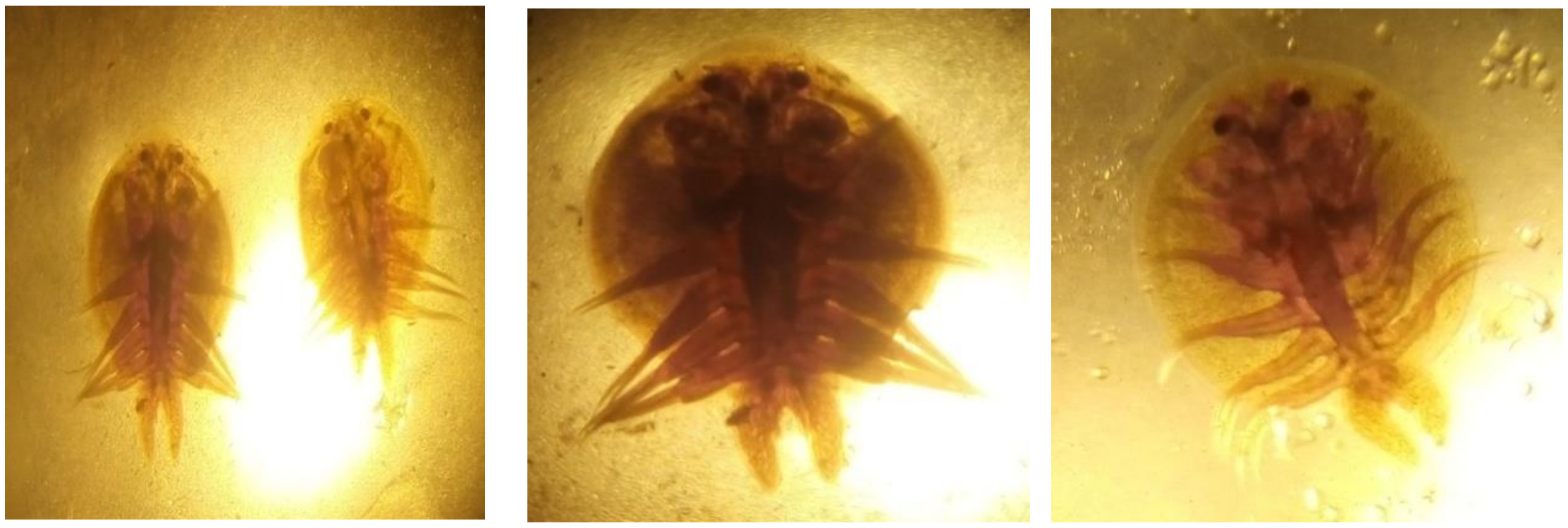

Figure 9a: *Dolops* species from the gill of *C. gariepinus*.

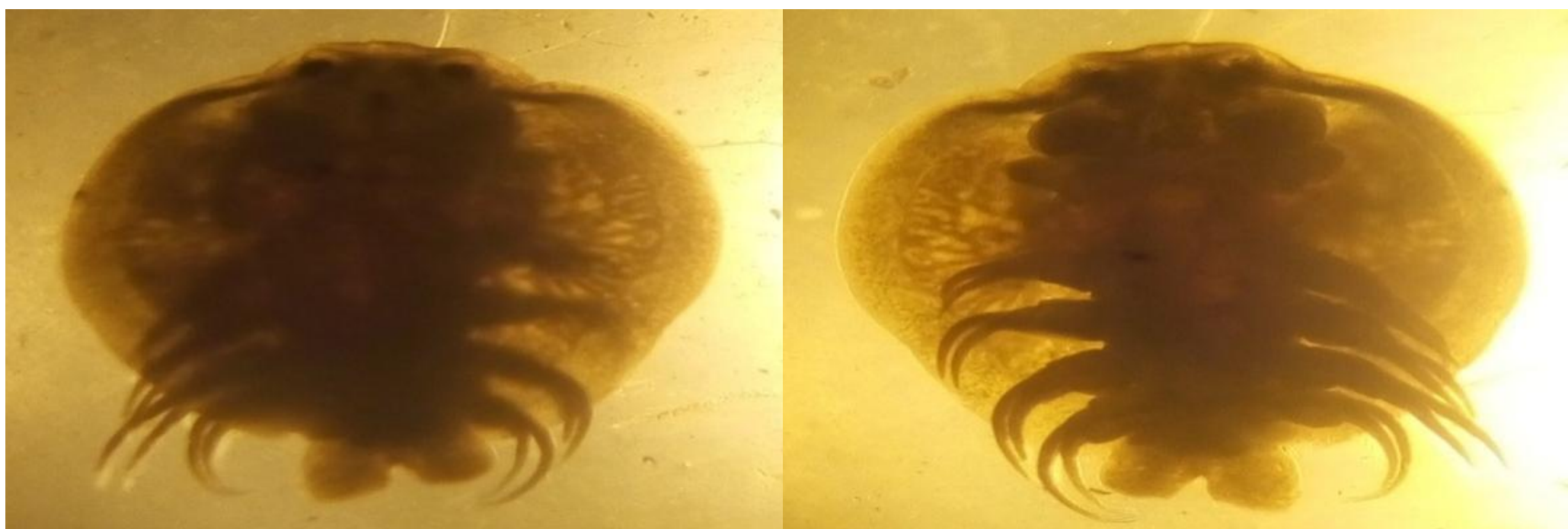

Figure 9b: Dorsal (left) and ventral view (right) of *Dolops* species from the skin of catfish.

5. REFERENCES

Abd-ELrahman, S, M., Gareh, A, Mohamed, H,I.H, I., Alrashdi, B,M.B, M., Dyab, A,K.A, K., El-Khadragy, M,F., Khairy Elbarbary, N., Fouad, A,M., El-Gohary, F,A., Elmahallawy, E,K.E, K., Mohamed, S,A.,2023. Prevalence and Morphological Investigation of Parasitic Infection in Freshwater Fish (Nile Tilapia) from Upper Egypt. Animals (Basel), 18;13(6), https://doi.org/doi: 10.3390/ani13061088

Abdel-Baki, A.A.S., Abdel-Haleem, H.M., Sakran, T., Zayed, E., Ibrahim, K.E. and Al-Quraishy, S., 2015. Two Myxobolus spp. infecting the kidney of Nile tilapia (Oreochromis niloticus) in the River Nile at Beni-Suef governorate, Egypt, and the associated renal changes. *Parasitology research*, **114**(3), pp.1107-1112.

Ageng'o, F, A., Waruiru, R, M.,Wanja, D, W., Nyaga, P, N., Hamisi, M, M.,Ndegwa, J, M.,Ali, S, E., ChadagE., Chadag, M, V.,Mbuthia P, G.P, G.,2025. Parasites of Farmed and Wild Tilapine Fishes from Selected Farms and Lake Jipe in Taita Taveta County, Kenya, https://doi.org/10.1002/aff2.70042

Amlacher, E., 2005. Textbook of Fish Diseases. Narendra Publishing House, India, pp 207–292.

Ardelli, B. F. and Woo P. T. K., 1997. "Protective antibodies and anamnestic response in *Salvelinus fontinalis* to *Cryptobia salmositica* and innate resistance of *Salvelinus*

namaycush to the hemoflagellate," *Journal of Parasitology*, vol. 83, no. 5, pp. 943–946, 1997.

Audicana, M, T., Kennedy, M, W.,2008. Anisakis simplex: from obscure infectious worm to inducer of immune hypersensitivity. Clin Microbiol Rev.;21(2):360-79, doi:10.1128/CMR.00012-07.

Bakke, T,A.,Harris, P,D.,Cable, J., 2002. Host specificity dynamics: observations on gyrodactylid monogeneans, Int. J. Parasitol., 32, pp. 281-308

Bekele, J., Hussein, D., 2015. Prevalence of internal parasites of *Oreochromis niloticus* and *Clarius gariepinus*, journal of aquatic research and development,6(2), *http://dx.doi.org/10.4172/2155-9546.1000308*

Borji, H., Naghibi, A., Nasiri, M.R. and Ahmadi, A., 2012. Identification of Dactylogyrus spp. and other parasites of common carp in northeast of Iran. *Journal of Parasitic Diseases*, **36**(2), pp.234-238.

Boyd CE, McNevin AA, Davis RP. The contribution of fisheries and aquaculture to the global protein supply. Food Secur. 2022;14(3):805-827. https://doi.org/doi: 10.1007/s12571-021-01246-9.

Brian, J.I.,2023. Parasites in biodiversity conservation: Friend or foe? Trends Parasitol.; 39:618–621. https://doi.org/doi: 10.1016/j.pt.2023.05.005.

Brye K, 2023. Aquaculture: A Sustainable Solution for Fish Cultivation and Global Food Security, Journal of Agriculture, 6(4),1-3

Chai, J.Y.; Murrell, K.D.; Lymbery, A.J.,2005. Fish-borne parasitic zoonoses: Status and issues. *Parasitol. Int.* **2005**, *35*, 1233–1254

Chew, X, Z., Cobcroft,J.Cobcroft, J., Hutson, K, S.,2024. Fish ectoparasite detection, collection and curation. Adv Parasitol, 125:105-157. https://doi.org/doi: 10.1016/bs.apar.2024.05.001

Chimdo, A. Review on potential and challenges of aquaculture practice in Ethiopia. *Appl Water Sci* **12**, 218 (2022). https://doi.org/10.1007/s13201-022-01740-1

CNN travel, 2023. Ethiopian food: the 15 best dishes, https://edition.cnn.com/travel/article/ethiopian-food-best-dishes-africa/index.html#:~:text=Eating%20fish%20%E2%80%93%20asa%20%E2%80%93%20in%20Ethiopia,a%20piranha%20on%20your%20plate

Dadebo, E., 2000. Reproductive biology and feeding habits of the catfish Clarias gariepinus (Burchell) (Pisces: Clariidae) in Lake Awassa, Ethiopia. *SINET: Ethiopian Journal of Science*, **23**(2), pp.231-246.

Deng, G, T.2020. Assessment of Factors Affecting Fish Production and Marketing in Gambella Region, Ethiopia. ScientificWorldJournal. 16; 2020:5260693. doi: 10.1155/2020/5260693.

Desalegn, T. and Shitaw, T., 2021.Fishery Resources, Conservation Challenges and Management Strategies in Ethiopia. *Fisheries and Aquaculture Journal*, **12**(3), pp.1a-1a.

El-Shahawy, I.S., El-Seify, M.O., Metwally, A.M., Fwaz, M.M. 2017. Survey on endoparasitic fauna of some commercially important fishes of the River Nile, southern of Egypt (Egypt) Rev. De Med. Vet.; 168:126–134.

Folefack, G.B.L., Kengne, C.M., Dongmo, B.F. and Fomena, A., 2019. Prevalence and mean intensity of Myxobolus spp. Parasitizing Orechromis niloticus in Cameroon. *International Journal of Biology*, **11**(2), pp.35-45.

Food Business, 2025. Ethiopia's Grand Renaissance Dam to increase daily fish production – Ministry of Agriculture, https://www.foodbusinessafrica.com/

Fraulo, P., Morena, C., & Costa, A. (2014). Recovery of Anisakid larvae by means of chloro-peptic digestion and proposal of the method for the official control. *Acta Parasitologica*, **59**(4), 629–634.

Galaktionov, K, V.K, V., Irwin, S, W,S., B.W, B., and Saville, D, HD, H, 2006. One of the most complex life cycles among trematodes: a description of *Parvatrema margaritense* (Ching, 1982) n. comb. (Gymnophallidae) possessing parthenogenetic metacercariae. Parasitology **132**, 733–746

Gebreegziabher, H., Degefu, H. and Tsegay, A.K., 2020. Prevalence of internal helminth parasites of fish in Gilgel-Gibe River and three selected ponds in and around Jimma Town, Southwest Ethiopia. *Turkish Journal of Fisheries and Aquatic Sciences*, **20**(9), pp.693-699.

Getahun, A., 2002. The Nile Basin: Riverine Fish and Fisheries. Dept. of Biology Addis Ababa University, Ethiopia, pp: 19.

Grossart, H, P., Van den, S., Wyngaert, M. Kagami, C. Wurzbacher, M. Cunliffe, K., 2019. Rojas-Jimenez, Nat. Rev. Microbiol., 17, pp. 339-354, 10.1038/s41579-019-0175-8

Gulelat, Y., Yimer, E., Asmare, K. and Bekele, J., 2013. Study on parasitic helminths infecting three fish species from Koka reservoir, Ethiopia. *SINET: Ethiopian Journal of Science*, **36**(2), pp.73-80.

Hailu, M., Mitiku, M, A., 2021. The Effects of Fish Parasites in Freshwater Culture and Capture Fisheries and their Treatment Mechanisms, International Journal of Zoology and Animal Biology (IZAB),4(2), 1-10, https://doi.org/10.23880/izab-16000289.

Hasegawa, R, Otsuki Y, Uemura, Y., Furusawa, C., Naka, M., Kanno, Y., Koizumi, I., 2025. Positive feedback between parasite infection and poor host body condition reduces host survival in the wild, https://doi.org/10.1111/1365-2435.14742

Hoffman GL. 2019. Parasites of North American freshwater fishes. Cornell University Press.

Hundie, K., Berisa, L., Shitaw, T., Asmamaw, B. and Fekansa, T., 2021.Evaluating Household's Willingness to Pay for Catfish Species Conservation in Lake Hawassa, Sidama National Region State, Ethiopia. *International Journal of Oceanography and Aquaculture*, **5**(2):000204.

Imam, T.S., and Dewu, R.A., 2010. Survey of piscine ecto-and intestinal parasites of clarias species sold at Galadima Road Fish Market, Kano metropolis, Nigeria. *Bioscience Research Communications*, **22**(4), pp.209-214.

Janda, J, M., Abbott, S, L.,2010. The genus Aeromonas: taxonomy, pathogenicity, and infection. Clin Microbiol Rev, 23(1):35-73. doi: 10.1128/CMR.00039-09.

Kaba, T, Shurbe, M, Wondimu, A. 2024. Awareness of fish-borne zoonoses and prevalence of Contracaecum in Oreochromis niloticus and Lates niloticus collected from Lake Chamo, Arba Minch, Ethiopia. Zoonoses Public Health, 71(7):790-798. doi: 10.1111/zph.13145

Kaur, K., Reddy, S., Barathe, P., Oak, U., Shriram, V., Kharat, S, S., Govarthanan, M., Kumar, V., 2022. Microplastic-associated pathogens and antimicrobial resistance in environment, Chemosphere, 291, 10.1016/j.chemosphere.2021.133005

Kaygorodova, I, A., Sorokovikova, N, V. 2014. Mass leech infestation of sculpin fish in Lake Baikal, with clarification of disease-prone species and parasite taxonomy. Parasitol Int, ;63(6):754-7. https://doi.org/doi: 10.1016/j.parint.2014.06.009.

Kelsey, D, S., 1996. Enteric Nematodes of Lower Animals Transmitted to Humans: Zoonoses in Medical Microbiology, 4th edition.

Khan, S., Ahmed, S., Serajuddin, M., Saifullah, M, K., 2018. Variation in seasonal prevalence and intensity of progenetic metacercariae of Clinostomum complanatum infection in

Trichogaster fasciatus fish, Beni-Suef University Journal of Basic and Applied Sciences, 7(3),310-316, https://doi.org/10.1016/j.bjbas.2018.03.006.

Khidr, A, A., Said, A, E., Abu Samak, O, A., Abu Sheref,S,E., 2012. The impacts of ecological factors on prevalence, mean intensity and seasonal changes of the monogenean gill parasite, Microcotyloides sp., infesting the Terapon puta fish inhabiting coastal region of Mediterranean Sea at Damietta region, The Journal of Basic & Applied Zoology, 65(2),109-115, https://doi.org/10.1016/j.jobaz.2012.06.003.

Kiprono, S.W., 2017. Fish parasites and fisheries productivity in relation to extreme flooding of Lake Baringo, Kenya. *Published master's Thesis. Kenyatta University*.

Klimpel, S., Thomas, K., Julian, M., Dorian, D. D., Regina, K., Judith, K.,2019. *Parasites of marine fish and cephalopods - A practical guide* (1st ed.). Springer.

Klinger, R.E. and Floyd, R.F., 1998. *Introduction to freshwater fish parasites*. Gainesville: University of Florida Cooperative Extension Service, Institute of Food and Agriculture Sciences, EDIS.

Kubitza, F., 2016. Common salt a useful tool in aquaculture, part 1, Simple ingredient offers multiple benefits to freshwater fish farmers, https://www.globalseafood.org/, accessed April 2025.

Labaude S, Rigaud T, Cézilly F, 2015. Host manipulation in the face of environmental changes: Ecological consequences, International Journal for Parasitology: *Parasites and Wildlife*, **4**(3), PP. 442-451, https://doi.org/10.1016/j.ijppaw.2015.08.001

LEDP, 2006. Lake Fishery development project, Proceeding of the national fishery seminar. Ziway number 1995. Lake Fishery development project working paper number 21AA ministry of agriculture.

Leung, T, L, F.,2022. Economies of parasite body size. Curr Biol. 32(12): R645-R649. https://doi.org/doi: 10.1016/j.cub.2022.01.059.

LFDP, 1996. Fisheries statistical bulletin No.3. Lake Fisheries Development Project Working Paper 20. Ministry of Agriculture, Addis Ababa, Ethiopia

Lima, D, P., Giacomini, H, C., Takemoto, R, M., Agostinho, A, A., Bini, L, M.,2012. Patterns of interactions of a large fish-parasite network in a tropical floodplain. J Anim Ecol.,81(4):905-13. https://doi.org/doi: 10.1111/j.1365-2656.2012.01967. x.

Ljubojevic, D., Novakov, N., Djordjevic, V., Radosavljevic, V., Pelic, M., & Cirkovic, M. (2015a). Potential parasitic hazards for humans in fish meat. *Procedia Food Science*, **5**, 172–175.

Ljubojevic, D., Radosavljevic, V., Puvaca, N., Balos, M. Z., Đorđevic, V., Jovanovic, R., & Cirkovic, M. (2015b). Interactive effects of dietary protein level and oil source on proximate composition and fatty acid composition in common carp (*Cyprinus carpio* L.). *Journal of Food Composition and Analysis*, **37**, 44–50.

Marcogliese, D. J. 2005. Parasites of the superorganism: Are they indicators of ecosystem health? International Journal of Parasitology 35:705-716.

Melak, T., and Getahun, A., 2012. Diversity and relative abundance of fishes in some temporary and perennial water bodies of the Baro basin, Gambella, Ethiopia, Ethiop. J. Biol. Sci. 11(2):193-206,

Menberu, Z., Mogesse, B., Reddythota, D.,2021. Evaluation of water quality and eutrophication status of Hawassa Lake based on different water quality indices, Applied Water Science, 11:61 https://doi.org/10.1007/s13201-021-01385-6

Mengesha T, A., 2015. Fish Species Diversity in Major River Basins of Ethiopia: A Review, World Journal of Fish and Marine Sciences 7 (5): 365-374, 2015, 10.5829/idosi.wjfms.2015.7.5.95195

Mgbemena, A., Arimoro, F., Omalu, I, and Keke, U., 2020. Prevalence of helminth parasites of Clarias gariepinus and Tilapia zillii in relation to age and sex in an afrotropical stream, Egyptian Journal of Aquatic Biology & Fisheries, https://www.ejabf.journals.ekb.eg/

Mitiku, M.A., 2017. Parasite Species Richness of Fish from Fishponds and Fingerling Sources in Central Ethiopia: It's Implication on Aquaculture Development (Doctoral dissertation, Doctoral dissertation, University of Natural Resources and Life Sciences).

Mitiku, M.A., Teklu, A. and Tadesse, Z., 2018. Prevalence of parasite of Nile Tilapia *(Oreochromis niloticus)* and African Big Barb *(Labeobarbus intermidus)* fish in Gigel-Gibe-I Dam, Jimma Zone, Ethiopia, American-Eurasian Journal of Scientific Research **13**(2): 18-24, 2018.

Mukama, S., 2008. Variations in abundance and diversity of parasites infecting catfish, *Clarias gariepinus (Burchell 1822) and tilapia Oreochromis urolepis (Norman 1922) in the Mindu dam: Morogoro municipality, Tanzania* (Doctoral dissertation, Unesco-IHE).

Nikolaev KE, Sukhotin AA and Galaktionov KV (2006). Infection patterns in White Sea blue mussels (*Mytilus edulis* L.) of different age and size with metacercariae of *Himasthla elongata* (Echinostomatidae) and *Cercaria parvicaudata* (Renicolidae). Diseases of Aquatic Organisms **71**, 51–58

NOAA Fisheries, 2020. Aquaculture Supports a Sustainable Earth, https://www.fisheries.noaa.gov/, accessed April 2025.

Ogello EO, Mlingi FT, Nyonje BM *et al*. Can integrated livestock-fish culture be a solution to East Afircan's food insecurity? A Review.*African J Food Agric Nutr Dev.13 (4)*, 8058-8078 (2013).

Okon, E,M., Oyesiji, A,A., Okeleye, E,D., Kanonuhwa, M., Khalifa, N,E., Eissa, E,H., Mathew, R,T., Eissa, M,E,H., Alqahtani, M,A., S, A.lnour, S,A., 2024.The Escalating threat of climate change-driven diseases in fish: Evidence from a global perspective - A literature review. Environ Res, 263(Pt 3):120184. doi: 10.1016/j.envres.2024.120184.

Outa, J.O., Dos Santos, Q.M., Avenant-Oldewage, A. *et al*. 2021. Parasite diversity of introduced fish *Lates niloticus*, *Oreochromis niloticus* and endemic *Haplochromis* spp. of Lake Victoria, Kenya. *Parasitol Res* **120**, 1583–1592. https://doi.org/10.1007/s00436-021-07095-x

Ozer, A., Oztruk, T., Oztruk, M, O.,2002. Prevalence and intensity of *Gyrodactylus arcuatus* Bychowsky, 1933 (Monogenea) infestations on the three-spined stickleback, *Gasterosteus aculeatus* L., 1758, Turk. J. Vet. Anim. Sci, pp. 807-812

Oztruk, M, O., Alutnel, F, N.,2006. Occurrence of Dactylogyrus infection linked to seasonal changes and host fish size on four cyprinid fishes, Acta Academiae, 52 (4), pp. 407-415

Paperna, I., 1996. Parasite infection and diseases of fish in Africa: An update. Technical Paper **31**. Rome, Central Institute of Freshwater Aquaculture. *Food and Agriculture Organization, United Nations*.

Paulose, T, Nkosi, Z, Z, Endriyas, M. 2022. Prevalence of hypertension and its associated factors in Hawassa city administration, Southern Ethiopia: Community based cross-sectional study. PLoS One. 1;17(3): e0264679. doi: 10.1371/journal.pone.0264679.

Petty, B, D., Francis-Floyd, R., YanongR., Yanong, R., P,2022. Parasitic Diseases of Fish, https://www.msdvetmanual.com/, accessed, April, April 2025

Pouder, D.B., Curtis, E.W. and Yanong, R.P.E., 2005. Common freshwater parasite pictorial guide edis. *Online). http://edis. ifas. ufl. edu*.

Poulin R, 2014. Parasite biodiversity revisited: frontiers and constraints, International Journal for Parasitology, 44(9),2014, Pages 581-589, ISSN 0020-7519, https://doi.org/10.1016/j.ijpara.2014.02.003

Poulin R, Presswell B. 2022. Is parasite taxonomy really in trouble? A quantitative analysis, International Journal for Parasitology, 52, 7, 2022, 469-474, https://doi.org/10.1016/j.ijpara.2022.03.001

Poulin, R., 2005. Variation in the intraspecific relationship between fish length and intensity of parasitic infection: biological and statistical causes, https://doi.org/10.1111/j.1095-8649.2000.tb02090.x

Pradipta R. Rauta, Bismita Nayak, Surajit Das, 2012. Immune system and immune responses in fish and their role in comparative immunity study: A model for higher organisms, Immunology Letters,Volume 148, Issue 1,Pages 23-33, ISSN 0165-2478, https://doi.org/10.1016/j.imlet.2012.08.003

Ramos P, Parasites in fishery products - Laboratorial and educational strategies to control, Experimental Parasitology, 211,2020, 107865, ISSN 0014-4894, https://doi.org/10.1016/j.exppara.2020.107865.

Reed, C, C., Basson, L., Van As, L, L.,2002. Myxobolus species (Myxozoa), parasites of fishes in the Okavango River and Delta, Botswana, including descriptions of two new species. Folia Parasitol (Praha);49(2):81-8. doi: 10.14411/fp.2002.016.

Reshid, M., Adugna, M., Redda, Y.T., Awol, N. and Teklu, A., 2015. A study of Clinostomum (trematode) and Contracaecum (nematode) parasites affecting Oreochromis niloticus in Small Abaya Lake, Silite Zone, Ethiopia. *J. Aquac. Res. Dev*, **6**, p.316.

Roberts, R.J., 2001. *Fish Pathology*.3rd edn. Technical Director, Landa catches Ltd. Scotland, pp. 270–300.

Roy, P.E. and Young, E., 2002. Nematode (round worm) infection in fish. *Florida, IFAS*, **91**, pp.1-10.

Senapin, S., Phiwsaiya, K., Laosinchai, P., Kowasupat, C., Ruenwongsa, P., Panijpan, B., 2014. Phylogenetic analysis of parasitic trematodes of the genus Euclinostomum found in Trichopsis and Betta fish. J Parasitol.;100(3):368-71. https://doi.org/doi: 10.1645/13-285.1.

Shafiq A., Abbas F., Hafeez-Ur-Rehman M., Khan B.N., Aihetasham A., Amin I., Hmidullah, Mothana R.A., Alharbi M.S., Khan I., et al. Parasite Diversity in a Freshwater Ecosystem. Microorganisms. 2023; 11:1940.

Shamsi S, Turner A, Wassens S. 2018. Description and genetic characterization of a new Contracaecum larval type (Nematoda: Anisakidae) from Australia. J Helminthol. 2018 Mar;92(2):216-222. doi: https://doi.org/10.1017/S0022149X17000360.

Shamsi, S., Barton, D.P. 2023. A critical review of anisakidosis cases occurring globally. *Parasitol Res* **122**, 1733–1745. https://doi.org/10.1007/s00436-023-07881-9

Simková, A., Jarkovský, J., Koubková, B., Barus, V, Prokes, M.,2005. Associations between fish reproductive cycle and the dynamics of metazoan parasite infection. Parasitol Res, 95(1):65-72. https://doi.org/doi: 10.1007/s00436-004-1261-y.

Skelton, P.H., Tweddle, D., Jackson, P.B.N. 1991. Cyprinids of Africa. In: Winfield, I.J., Nelson, J.S. (eds) Cyprinid Fishes. Fish & Fisheries Series, vol 3. Springer, Dordrecht, https://doi.org/10.1007/978-94-011-3092-9_7

Steckler N., Yanong, Yanong R, PR., 2024. Argulus (Fish Louse) Infections in Fish, https://edis.ifas.ufl.edu/

Sures, B., Nachev, M.,2022. Effects of multiple stressors in fish: how parasites and contaminants interact. Parasitology;149(14):1822-1828. doi: 10.1017/S0031182022001172.

Tadesse, B., 2009. Prevalence and abundance of fish parasites in Bomosa Cage Systems and lakes Babogaya and Awassa, Ethiopia.

Tesgera G, and Bayou K, 2021. Assessment of Current Practices, Major Challenges and Opportunities of the Fishery of Lake Hawassa, Southern Ethiopia, DOI: http://dx.doi.org/10.22192/ijarbs.2021.08.02.004, http://www.ijarbs.com/, 8(2), 1-9

Tesfay, S., Teferi, M., Dejenie, T., Abay T., Hiluf, G., 2024. A survey of ecto and endoparasites of Nile tilapia, Oreochromis niloticus (Linnaeus, 1758) fingerlings in Midmar reservoir, Adwa, northern Ethiopia, Journal of Applied Animal Research, 52:1, 2310158, https://doi.org/DOI: 10.1080/09712119.2024.2310158

Tesfaye, A., Teklu, A., Bekelle, T., Tkue, T., Kebede, E., Gebretsadik, T. and Berhe, N., 2017.A survey on occurrence of internal and external fish parasites and causes of fish population reduction in Lake Hashenge, Tigray, Ethiopia. *Ethiopian Veterinary Journal*, **21**(2), pp.75-91.

Tesfaye, Z., Ferede, B., Pavanasam, N. and Workagegn, K.B., 2020. Prevalence of nematode parasite, Contracaecum, in Nile tilapia, African catfish and Barbus species in Lake Hawassa, Ethiopia.Aquaculture Research, **51**(10), pp.3993-3998.

Thakkar AN, 2023, Seasonal Variation in Prevalence of Nematode Parasites In Coilia Dussumieri From Uran, West Coast of India, Journal of Survey in Fisheries Sciences, 10(1) 4007-4009

Thrusfield, M.V., 2005. Veterinary Epidemiology 2nd ed. Black Well Science LTD, UK. pp. 233-250.

Tibebe, D., Tesfaye, Y. Kassa, Y., 2023.The impact of sewage effluents on water quality of Lake Hawassa, Ethiopia. *BMC Chemistry* **17**, 42. https://doi.org/10.1186/s13065-023-00954-x.

Tilahun, G., and Bihonegn, T., 2017.Study on Helminth Parasites in *Tilapia nilotica* from Lake Zeway, Ethiopia, Int. J. Adv. Res. Biol. Sci, **4**(10): 21-25.

Truter, M., Hadfield, K, A., Smit, N, J., 2023. Review of the metazoan parasites of the economically and ecologically important African sharptooth catfish Clarias gariepinus in Africa: Current status and novel records. Adv Parasitol.;119:65-222. doi: https://doi.org/10.1016/bs.apar.2022.11.001.

Trutera, M., Hadfielda K, A., and Smit N, J., 2022. Review of the metazoan parasites of the economically and ecologically important African sharptooth catfish Clarias gariepinus in Africa: Current status and novel records, 1-158, https://doi.org/10.1016/bs.apar.2022.11.001

Valtonen, E.T., Marcogliese, D.J. and Julkunen, M., 2010. Vertebrate diets derived from trophically transmitted fish parasites in the Bothnian Bay. *Oecologia*, **162**(1), pp.139-152.

Vandi, P., Daniel, J. L.2023. Prevalence of Gastrointestinal Parasites and Condition Factor of Oreochromis niloticus (L.) in Kiri Reservoir, Shelleng, Adamawa State, Nigeria, Biological and Environmental Sciences Journal for the Tropics 20(3), https://dx.doi.org/10.4314/bestj.v20i3.7, 182-188.

Vidal-Martínez, V, M., Aguirre-Macedo, M, L., McLaughlin, J, P., Hechinger, R, F., Jaramillo, A, G., Shaw, J, C., James, A, K., Kuris, A, M., Lafferty, K, D.,2012. Digenean metacercariae of fishes from the lagoon flats of Palmyra Atoll, Eastern Indo-Pacific. J Helminthol, 86(4):493-509. doi: https://doi.org/10.1017/S0022149X11000526

Wakjira,M., Tolemariam,T., Kim, D, J., Kim, K, R., 2013. Aquaculture development in Ethiopia: review on potential and strategy, journal of agriculture, life and environmental sciences, 25(3), 20-24

WHO, 2020. Zoonoses, https://www.who.int/, assessed April 18, 2025.

Zekarias, T. and Yimer, E., 2007. Study on parasites of fish at Lake Awassa, Ethiopia. *Bulletin of Animal Health and Production in Africa*, **55**(3), pp.149-155.

Zhokhov, A.E., Mironovsky, A.N. and Miretskaya, D.A., 2007. Methods of the complete Parasitological dissection of fish. *Freshwater biology group, JERBE, Moscow–Addis-Ababa*, pp.1-12.

Ziarati, M., Zorriehzahra, M, J., Hassantabar, F., Mehrabi, Z., Dhawan, M., Sharun, K., Emran, T, B., Dhama, K., Chaicumpa, W., Shamsi, S.,2022. Zoonotic diseases of fish and their prevention and control. Vet Q.;42(1):95-118. https://doi.org/ doi: 10.1080/01652176.2022.2080298.

Renner, E.D., Duggan, I.C., 2024. Season, size, and sex: factors influencing monogenean prevalence and intensity on *Gambusia affinis* in New Zealand. *Parasitol Res* **123**, 228. https://doi.org/10.1007/s00436-024-08241-x
