# Supplementary material for: Prevalence of fish parasites from Lake Hawassa, Ethiopia
Source: PLoS One. 2026 Sep 11;21(9):e0357816. doi: 10.1371/journal.pone.0357816 (PMC13568496; doi:10.1371/journal.pone.0357816)
Supplement: Supplementary file 3 [file pone.0357816.s005.pdf]

PLOS One
Prevalence of fish parasites from Lake Hawassa, Ethiopia
--Manuscript Draft--

**Manuscript Number:**	PONE-D-25-59453R1
**Article Type:**	Research Article
**Full Title:**	Prevalence of fish parasites from Lake Hawassa, Ethiopia
**Short Title:**	Prevalence of Oreochromis niloticus, Clarias gariepinus, and Barbus intermedius
**Corresponding Author:**	DEBELA ABDETA EFA, Doctor of Veterinary Medicine<br>Addis Ababa University<br>Bishoftu, ETHIOPIA
**Keywords:**	fish; Lake Hawassa; Parasites; Prevalence
**Abstract:**	Background<br><br>Fish provides quality protein, variety of vitamins, and minerals to human being. More than 17% of animal protein for global population is obtained from fish. An estimated 200 species of fishes are known to occur in Ethiopia. Fish parasites are the main cause of the significant threat to the fish biodiversity of Ethiopia's freshwater islands. Fish parasite of the lake Hawassa are hitherto poorly known and there are limited reports on the type of pastes across the different fish hosts.<br><br>Methods<br><br>a cross-sectional study was carried out in 2023- 2024 to estimate the prevalence of fish parasites and host factors. Internal and external organs was inspected for the presence of the parasite infection. Some external parasites were extracted from the fresh water immediately after fish were caught. The randomly selected host was classified based on species, sex, body weight, and the total body length. Parasites are identified morphologically at genera level using standard identification keys and pictorial guide. Analysed data were presented as frequencies, percentages, mean intensity, and mean abundance. Chi-square and P-values were considered for statistical association.<br><br>Results<br><br>in the current survey Oreochromis niloticus(n=135), Clarias gariepinus (n = 87), and Barbus intermedius (n = 43) were included. The parasite distribution varies with sex, body weight, and standard length of the studied fish. The overall infection/infestation rate is 88.30% (n = 234). A total of 1748 parasites classified under trematodes, nematodes, protozoa, crustaceans and Hirudinea was collected from O. niloticus (787), C. gariepinus (642) and B. intermedius (318). The internal parasites recovered from O. niloticus were Clinostomum (65.16%), Euclinostomum (34.07%), Contracaecum (26.67%), Dactylogyrus species (28.15%), and Diplostomum (24.44%). Significant infection level with Myxobolus species, leeches, Argulus, and Dolops species was also recorded. Digenean encysted metacercaria (68.97%) and Contracaecum species (50.57%) are the most common parasite species infecting C. gariepinus. Diplostomum species, Dactylogyrus species, leeches, Argulus species, and Dolops species pose a serious threat to fish health in lake Hawassa. The mean intensity of the parasites in B. intermedius, C. gariepinus and O. niloticus is found to be 8.59, 7.92 and 6.79 while mean abundance is 7.40, 7.37 and 5.84 respectively.<br><br>Conclusion<br><br>This study found that various body parts are contaminated with one or more parasites. Some of those parasites have established zoonosis or cause cross infection to fishes requiring urgent cooperation in performing pre-stock assessment for optimal production and maintaining the heath of fishery and hence human being.
**Order of Authors:**	DEBELA ABDETA EFA, Doctor of Veterinary Medicine

	Megersa Akasa
	Yobsan Tamiru
**Response to Reviewers:**	We thank the editor in chief and the reviewers for their well appreciated comments and suggestion. We have addressed all the comments and provided separate the response letter to each reviewers
**Additional Information:**	
**Question**	**Response**
**Financial Disclosure**<br><br>Enter a financial disclosure statement that describes the sources of funding for the work included in this submission. Review the submission guidelines for detailed requirements. View published research articles from *PLOS ONE* for specific examples.<br><br>This statement is required for submission and **will appear in the published article** if the submission is accepted. Please make sure it is accurate.<br><br>**Funded studies**<br>Enter a statement with the following details:<br>• Initials of the authors who received each award<br>• Grant numbers awarded to each author<br>• The full name of each funder<br>• URL of each funder website<br>• Did the sponsors or funders play any role in the study design, data collection and analysis, decision to publish, or preparation of the manuscript?<br><br>Did you receive funding for this work?	The author(s) received no specific funding for this work.
**Competing Interests**<br><br>Use the instructions below to enter a competing interest statement for this submission. On behalf of all authors, disclose any competing interests that could be perceived to bias this work—acknowledging all financial support and any other relevant financial or non-financial competing interests.	The authors have declared that no competing interests exist.

Powered by Editorial Manager® and ProduXion Manager® from Aries Systems Corporation

This statement is required for submission and **will appear in the published article** if the submission is accepted. Please make sure it is accurate and that any funding sources listed in your Funding Information later in the submission form are also declared in your Financial Disclosure statement.<br><br>View published research articles from *PLOS ONE* for specific examples.<br><br>**NO authors have competing interests**<br><br>Enter: *The authors have declared that no competing interests exist.*<br><br>**Authors with competing interests**<br><br>Enter competing interest details beginning with this statement:<br><br>*I have read the journal's policy and the authors of this manuscript have the following competing interests: [insert competing interests here]*<br><br>* typeset	
**Ethics Statement**<br><br>Enter an ethics statement for this submission. This statement is required if the study involved:<br><br>• Human participants<br>• Human specimens or tissue<br>• Vertebrate animals or cephalopods<br>• Vertebrate embryos or tissues<br>• Field research<br><br>Write "N/A" if the submission does not require an ethics statement.<br><br>General guidance is provided below. Consult the submission guidelines for detailed instructions. **Make sure that all information entered here is included in the Methods section of the manuscript.**	N/A

Powered by Editorial Manager® and ProduXion Manager® from Aries Systems Corporation

**Format for specific study types**<br><br>**Human Subject Research (involving human participants and/or tissue)**<br>• Give the name of the institutional review board or ethics committee that approved the study<br>• Include the approval number and/or a statement indicating approval of this research<br>• Indicate the form of consent obtained (written/oral) or the reason that consent was not obtained (e.g. the data were analyzed anonymously)<br><br>**Animal Research (involving vertebrate animals, embryos or tissues)**<br>• Provide the name of the Institutional Animal Care and Use Committee (IACUC) or other relevant ethics board that reviewed the study protocol, and indicate whether they approved this research or granted a formal waiver of ethical approval<br>• Include an approval number if one was obtained<br>• If the study involved *non-human primates*, add *additional details* about animal welfare and steps taken to ameliorate suffering<br>• If anesthesia, euthanasia, or any kind of animal sacrifice is part of the study, include briefly which substances and/or methods were applied<br><br>**Field Research**<br><br>Include the following details if this study involves the collection of plant, animal, or other materials from a natural setting:<br>• Field permit number<br>• Name of the institution or relevant body that granted permission	
**Data Availability**<br><br>Authors are required to make all data underlying the findings described fully available, without restriction, and from the time of publication. PLOS allows rare exceptions to address legal and ethical concerns. See the PLOS Data Policy and FAQ for detailed information.	Yes - all data are fully available without restriction

A Data Availability Statement describing where the data can be found is required at submission. Your answers to this question constitute the Data Availability Statement and **will be published in the article**, if accepted.<br><br>**Important:** Stating 'data available on request from the author' is not sufficient. If your data are only available upon request, select 'No' for the first question and explain your exceptional situation in the text box.<br><br>Do the authors confirm that all data underlying the findings described in their manuscript are fully available without restriction?	
**Describe where the data may be found in full sentences. If you are copying our sample text, replace any instances of XXX with the appropriate details.**<br><br>• If the data are **held or will be held in a public repository**, include URLs, accession numbers or DOIs. If this information will only be available after acceptance, indicate this by ticking the box below. For example: *All XXX files are available from the XXX database (accession number(s) XXX, XXX.*).<br>• If the data are all contained **within the manuscript and/or Supporting Information files**, enter the following: *All relevant data are within the manuscript and its Supporting Information files.*<br>• If neither of these applies but you are able to provide **details of access elsewhere**, with or without limitations, please do so. For example:<br><br>*Data cannot be shared publicly because of [XXX]. Data are available from the XXX Institutional Data Access / Ethics Committee (contact via XXX) for researchers who meet the criteria for access to confidential data.*<br><br>*The data underlying the results presented in the study are available*	All relevant data are within the manuscript and its Supporting Information files

from (include the name of the third party and contact information or URL).<br>• This text is appropriate if the data are owned by a third party and authors do not have permission to share the data.<br><br>* typeset	
Additional data availability information:	Tick here if the URLs/accession numbers/DOIs will be available only after acceptance of the manuscript for publication so that we can ensure their inclusion before publication.

Powered by Editorial Manager® and ProduXion Manager® from Aries Systems Corporation

clean manuscript

Click here to access/download;Manuscript;Debela et al_PONE-D-25-59453.docx

Prevalence of fish parasites from Lake Hawassa, Ethiopia

Debela Abdeta[1,4]*, Megersa Akasa[2], Yobsan Tamiru[3],

[1]College of Veterinary Medicine and Agriculture, Addis Ababa University, Bishoftu, Ethiopia

[2]College natural resource and agriculture, Ambo University, Gudar, Ethiopia

[3]School of Veterinary Medicine, Wollega University, Nekemte, Ethiopia

[4]Department of Veterinary Bioscience, Melbourne Veterinary School, Melbourne University, Melbourne, Australia

*Correspondence: Debela Abdeta

Tel. +251 913125925

Email: debela.abdeta@aau.edu.et, or debelaabdeta.efa@student.unimelb.edu.au

ABSTRACT

Background: Fish provides quality protein, various vitamins and minerals essential for human consumption. According to FAO, 2018 more than 17% of animal protein for global population is obtained from fish. An estimated 200 species of fishes of marine and freshwater fish are known to occur in Ethiopia. Fish parasites are the main cause of the significant threat to the fish biodiversity of Ethiopia's freshwater islands. Lake Hawassa is the economic heartbeat of the city which supports the artisanal fishing sector. The parasite of the fishes at lake Hawassa are hitherto poorly known and there are limited reports on the type of parasites across different fish hosts.

Methods: a cross-sectional study was carried out in 2023- 2024 to estimate the prevalence of fish parasites and host factors. In the current survey *Oreochromis niloticus* (n=135), *Clarias gariepinus* (n = 87), and *Barbus intermedius* (n = 43) were sampled. Internal and external organs was inspected for the presence of the parasite infection. Some external parasites were extracted from the fresh water immediately after fish were caught. The randomly selected host was classified based on species, sex, body weight, and the total body length. Parasites are identified morphologically at genera level using standard identification keys and pictorial guide. Analysed data were presented as frequencies, percentages, mean intensity, and mean abundance. Chi-square and P-values were considered for statistical association.

Results: The parasite distribution varies with sex, body weight, and standard length of the studied fish. The overall infection/infestation rate is 88.30% (n = 234). A total of 1748 parasites classified under Nematodes (e.g., Contracaecum), Digenean trematodes (e.g., Clinostomum, Euclinostomum, Diplostomum, encysted metacercariae), Monogeneans (Dactylogyrus), Myxozoans (Myxobolus spp.), Crustacean ectoparasites (Argulus, Dolops), Hirudinea (leeches)was collected from *O. niloticus* (788), *C. gariepinus* (642) and *B. intermedius* (318). The internal parasites recovered from *O. niloticus* were *Clinostomum* (65.16%), *Euclinostomum* (34.07%), *Contracaecum* (26.67%), and *Diplostomum* (24.44%). Significant infection level with *Myxobolus* species, leeches, *Argulus*, and *Dolops* species was also recorded. *Digenean encysted*

metacercaria (68.97%) and *Contracaecum species* (50.57%) are the most common parasite species infecting *C. gariepinus*. *Diplostomum species*, *Dactylogyrus species*, leeches, *Argulus species*, and *Dolops species* pose a serious threat to fish health in lake Hawassa. The mean intensity of the parasites in *B. intermedius*, C. *gariepinus* and *O. niloticus* was to be 8.59, 7.92 and 6.79 while mean abundance is 7.40, 7.37 and 5.84 respectively.

Conclusion: This study found that various body parts are contaminated with one or more parasites. Some of those parasites have established zoonosis or cause cross infection to fishes requiring urgent cooperation in performing pre-stock assessment for optimal production and maintaining the heath of fishery and hence human being.
Keywords: *Fish, Lake Hawassa, Parasites, Prevalence*

INTRODUCTION

The fish farming is one of the developing sectors in low- and medium-income countries offering significant potential for food security, growth of the economy and improvement of the livelihood (Ogello et al., 2013; Boyd et al., 2022). The demand for fish meat is steadily rising due to its high nutritional value, particularly essential fatty acids, which are ideal for human consumption (Ljubojevic et al., 2015a, 2015b, Brye, 2023). Fish resources are vital to support human well-being and economic development (Boyd et al., 2022). Aquaculture provides year-round jobs, supports resilient working water fronts and coastal communities and can provide sustainable economic growth. They also drive employment in other areas of industry such as seafood processing, feed and equipment manufacturing in different countries including Ethiopia (NOAA Fisheries, 2020).

Ethiopia is a landlocked nation that depends on its inland waterways for fish production. Several lakes and rivers in the country are used for fish production to feed its population while maintaining the nation's food security (Desalegn and Shitaw, 2021). The Ethiopian government has suggested aquaculture to increase fish output and as a substitute for food security and poverty alleviation (Deng, 2020). The data from the Ministry of Agriculture and Rural Development of Ethiopia, published in 2011, indicate that the total annual fish catch in Ethiopia rose from 14,000 tons in 1998 to 24,257 tons in 2011. Since then, there has been a plan to increase annual fish production by 65,000 tons per year. To achieve the plan the country constructed big dams such as Tendaho reservoir, Tekeze dam, Galgal Gibe I, II and III, and Renaissance dam in addition to the previously naturally used lakes (Gebremedhn and Tsegay, 2017). Today, aquaculture is seen as a crucial component of plans and strategies for rural and agricultural development in the country. The Ethiopian renaissance dam is emerging with a major fishery hub producing over 14,000kg of fishes daily (Food Business, 2025).

Lake Hawassa is one of the major lakes in the Ethiopian Rift Valley, situated close to Hawassa town and features an endorheic basin system. The water from the lake is essential to the nearby

towns for recreational use, residential water use, farming, and fish harvesting (Memberu et al., 2021). This lake is one of the most fished water bodies in Ethiopia where *O. niloticus* accounts for 90% of the annual catch (Tesgera and Bayou, 2021).

The freshwater fish fauna of Ethiopia is a mixture of Nilo Sudanic, East African and endemic forms (Skelton et al., 1991). The Nilo Sudanic forms those species represented under *Alestes, Labeo, Bagrus, Citharinus, Hydrocynus, Hyperopisus* and *Protopterus* are some representatives from Ghibe-Omo, Baro-Akabo, and Abay Basins (Melak and Getahun, 2012). The highland east African forms are found in the northern rift valley lakes (Hawassa, Ziway, Langano, and the high land lakes (Tana and Haiq). *Barbus, Clarias, Garra, Oreochromis* and *Varicosrinus* are the common genera of fish common to this area. They are related to fishes of eastern and southern African and Arabian Peninsula (Skelton et al., 1991). The fishes of high mountain torrential streams largely belong to *Cyprinidae* adapted to flowering flood waters that occur seasonally. Two genera of fishes (Barbus and Garra) are dominant in this stream (Getahun, 2002; Mengesha, 2015).

In Ethiopia, eating fish is a unique experience. As usual, it is consumed by hand with injera or bread and a hot sauce to dip into. The grilled fins are very delicious since, bear a few bones. Fish tibs are chunks of fish marinated in lime juice and chilli spices, followed by the addition of shredded ginger and garlic and sesame oil, olive oil, and paprika (CNN travel, 2023). Those practices could expose the community for public hazard as some of those parasites have zoonotic risk.

Fish production is challenged by variety problems from which fish disease is the dominant. In Ethiopia, fish disease particularly those caused by parasites and bacteria are concern with studies indicating high prevalence of parasites in commercially important fish species in lakes like Ziway, Hawassa, and Tana (Gebremedhn and Tsegay, 2017). Parasitic species can be found everywhere and on every living organism. The rate of parasitic infection is high among vertebrates residing in aquatic environment (Chai et al., 2005). Their presence in their host is generally at equilibrium in aquatic organisms and the most common lifestyle on the planet (Marcogliese, 2005, Tesfay et al, 2023, Chew et al, 2024).

More than one-third of all species on Earth are parasites, and they contribute significantly to ecosystem biomass. Due to this host-parasite interaction is one of the most prevalent biotic relationships in nature (Hasegawa et al., 2025). Parasites affect fish health through mechanical, physical and reproductive damage. These changes can reduce growth, fecundity and survival, change behavioural and sexual characteristics, and result in many other maladaptive alterations of the infected host (Hailu and Mitiku, 2021). Parasites are one of the main threats facing the fisheries industry (Power et al., 2025). This could be from economic losses due to fish rejection at markets, mortality in aquaculture, impact on fish health, or zoonotic concerns (Chai et al., 2005, Ziarati et al, 2022). In terms of host biology, population dynamics, and ecosystem health, parasites play a significant role. Any fish species, as well as any aquatic culture system, can contain them (Gulelat *et al*., 2013). The most common parasitic agents of fish are trematodes (fluke, e.g., *Opisthorchis*

spp.), nematodes (round worm, e.g., *Anisakis* *spp*.), and cestodes (tapeworm, e.g., *Diphyllobothrium* *spp*.). Furthermore, protozoan organisms like *Cryptosporidium* species are regarded as zoonotic infections originating from fish (Ziarati et al, 2022). If left unmanaged, these illnesses can cause massive fish deaths, limit fish productivity, and in some situations, can spread infections to humans and other animals who consume diseased or infected fish (Kiprono, 2017). In addition, arthropods and crustaceans' species are known to infect fish (Janda and Abbott, 2010). Different species of leeches are also known to infect fishes (Kaygorodova and Sorokovikova, 2014, Tesfay et al, 2023).

Studies have indicated that biotic factors such as sex, body length and height influence the burden of parasitism. Accordingly, female had significantly higher prevalence and mean intensity than males, longer fishes have higher prevalence and mean intensity. Additionally, females may have compromised immune system specially during reproductive season (Renner and Duggan., 2024).

Fish is a good host for parasite multiplication that can be zoonotic to humans or other animals that feed fishes (Barber and Wright, 2005). The larval stages of parasites could be found in fishes as they are either intermediate or reservoir of those parasites or sexually mature parasites are found for which they definitive hosts. The larvae of anisakid nematodes, trematodes or others can be ingested by humans with under-cooked fish flesh which then develop to morphologically mature but sexually immature worms thus, zoonotic potential (Kaba et al, 2024).

Parasites cause an economic threat to the market value of fish, through consumer attitudes towards the presence of these parasites within food products (Thakkar, 2023). Important determinants of parasite exposure include the distribution of infected hosts, host immune status, and the spatial and temporal distribution of infective stage and intermediate hosts, and this can be influenced by the host's diet or trophic position in a food chain (Valtonen *et al*., 2010). These elements slow down growth and weight gain, impact fish production yield, spread infections among humans and animals, delay in fish sexual maturation, and raise in fish mortality rates. Additionally, by posing marketing issues for commercially harvested species, these cause the nutritional devaluation of fish and resultant economic losses (Gebreegziabher *et al*., 2020). Although these parasites are an important part of biodiversity and can help maintain stability in natural population (Shafiq et al., 2023) they can also lead to extinction often interacting with other stressors such as habitat loss (Brian, 2023). The extinction can be population level or species level.

Moreover, in low- and middle-income countries like Ethiopia, people known little about fish borne zoonotic disease such as trematodiasis and anisakiasis (Kaba et al, 2024). Yet these parasitic infections are commonly reported from different freshwater body fishes (Tasfay et al., 2023) and the cause of significant human infections worldwide (Chai et al., 2005). To date, fewer studies have been conducted on fish parasites in Ethiopia, but they did not quantify the prevalence of parasites across different fish species, body weight, fish lengths and anatomical position in relation

to parasite infestation. The current study hypothesised that commercially important fish species from lake Hawassa are infected by internal and external parasites. Hence, the present study aimed at estimating the prevalence of internal and external parasites, calculating the intensity, abundance of *O. niloticus, C. gariepinus,* and *B. intermedius* in Lake Hawassa. We have also examined how host factors are related to the parasite prevalence or its burden.

MATERIALS AND METHODS

Description of the Study Area

One of the eight lakes in Ethiopia's Rift Valley is Lake Hawassa. Situated 275 km south of Addis Ababa in the Sidama region, it borders Hawassa city's eastern side. Geographically, the lake is located 1680 meters above sea level between 6°33' and 7°33' N and 38°22' and 38°29' E. The lake has an approximate water volume of 1.3 billion cubic meters (45.9 billion feet^3) and stretches 16 kilometres from northeast to southwest and 8 kilometres from northwest to southeast. The lake's mean depth is 11 meters, and its maximum depth is 21.6 meters (70.9 feet). The lake's surface area, which can reach 99.3 Km^2 during the rainy season, makes up 93.6 Km^2 of the catchment's total area of 1455 Km^2. According to estimates, Lake Hawassa releases 58 ×106 m^3 of groundwater annually into nearby basins (Hundie et al., 2021). The location of the lake and the city is found at https://en-us.topographic-map.com/map-lllbf3/Lake-Hawassa/

Study Design and sampling technique

A cross-sectional study was conducted from October 2023 to July 2024 on the fish community at Lake Hawassa to identify the prevalence of major internal and external parasites infesting fishes supported by antemortem and postmortem inspection on fish. The simple random sampling where each fish systematically selected (every specified fish from the cage) was used to select fishes for parasites identification. The assessment was conducted over a period of ten months, from October 2023 to July 2024.

Study Population

The Nile tilapia (Oreochromis niloticus), African catfish (*Clarias gariepinus*), and African big barb (*Labeobarbus intermedius*) are the fish species that have been found in Lake Hawassa thus far. ~~The black lampeye (*Aplocheilichthys antinorii*), the stone lapping minnow (*Gara quadrimaculata*), and the straight fin barb (*Barbus paludinosis*) are the other tiny fish present in the lake~~ (Dadebo, 2000). The other three species of minnow fish are not fished because of their small size, but *O. niloticus*, *C. gariepinus*, and *L. intermedius* are the commercially significant species in the nation. About 90% of the whole production is made up of *O. niloticus*, with *C.*

gariepinus and *L. intermedius* contributing roughly 7-8% and 2-3%, respectively (Wubie et al., 2023; LFDP, 1996).

Sample Size Determination

The sample size was determined according to Thrusfield, (2005) at 95% confidence interval with a 5% marginal error. Accordingly, Zekarias and Yimer (2007) reported prevalence of (79%) of fish parasites from Lake Hawassa. We have applied collective sample size for all the fishes sampled using the formula $n = z^2 \times p\,(1\text{-}p)/d^2$, where n= sample size, z = z statistic for a level of confidence (z =1.96 at 95% CI), p = prevalence (p = 0.79), d = precision (if 5%, d= 0.05), the sample size 265 is included in the current study.

Methods of data collection

Description of the sampled fish

During the sampling, we have randomly picked those fishes from different sampling points. The species, sex, possible anatomical region of the parasite attachment, body weight, and total and standard lengths of individual fish were initially assessed and recorded. The sex of each fish was tentatively assessed externally and confirmed post-dissection by noting the presence of testes or ovaries (Imam and Dewu, 2010). Up on arrival at the Hawassa University, the total length was measured by centimetres using measuring tape and the weight measured using digital balance. The size of fish was separately recorded for each fish species and classified into class I, class II, and class II (Tesfaye *et al*., 2020). Classifying fish species based on size and body weight during the parasite studies is crucial because this factor can influence parasite infection patterns, host vulnerability and the overall dynamics of parasite host interaction impacting both fish health and environmental ecosystem (Lima et al., 2012). The selected size breakpoints for length and weight classes reflect biologically meaningful transitions in growth and life-history stages. For example, in *C. gariepinus*, the length thresholds of 50 cm and 75 cm correspond approximately to ontogenetic stages associated with sexual maturation and trophic shifts. Individuals below 50 cm are typically juveniles or subadults with developing gonads and a diet dominated by invertebrates and small prey. Fish between 50–75 cm generally represents sexually mature adults, while individuals exceeding 75 cm are fully mature, larger-bodied predators with broader dietary niches and higher trophic positioning. These ontogenetic changes influence exposure risk to parasites through altered feeding behaviour, habitat use, and immune competence. Therefore, the selected size classes reflect biologically relevant developmental stages that may affect parasite acquisition dynamics within this ecosystem, rather than being purely arbitrary statistical categories (Keyombe et al., 2015).

Antemortem and postmortem identification of parasite

A total of 265 fish were first examined alive (skin and gill scraping and picking with forceps followed by freshly dissected samples). The randomly selected fish were immediately transported in an icebox to the Parasitology unit of Hawassa University, for parasitological examination. Fish were initially examined alive for external abnormalities immediately upon capture. They were then humanely euthanized at the sampling site and placed in an icebox containing crushed ice for transport to the laboratory. Fish were euthanized using a percussive blow to the head (concussion) immediately followed by brain destruction through pithing to ensure rapid loss of consciousness and death. To further ensure that no central nervous system activity remained, spinal pithing was performed as a final step. These procedures were applied to minimize distress and ensure humane euthanasia during sample collection (Mocho et al., 2025).

Each sampled fish was externally visualized, palpated, and examined for any ectoparasites on the entire fish skin, the fins, and opercular cavity including the gills. The external parasite was harvested to physiological salt solution from fish body and the fresh water (Kubitza, 2016). Scraping from fish skin were taken and smeared on to a clean microscope slide or hand lens. The sample was then examined under compound microscope at 10X, 40X and 100 X magnifications. Then postmortem examination was done using appropriate postmortem kits and standard evisceration technique (Zhokhov *et al*., 2007; Klimpel et al., 2019) and all body cavities was observed for presence of any encysted or free parasite larvae. The gill filaments, stomach, intestine, liver, heart, gall bladder, kidney and gonads were separately taken out and placed on clean Petridishes that contain 10millitre of physiological solution made of (0.9% sodium chloride w/v) (Younis et al., 2017). The head of *C. gariepinus* was dissected longitudinally, fixed in 70% ethanol first, and the cranial cavity was washed away into a petridish using a water dropper and checked for parasites especially *Diplostomum* species. To clear their cuticle, the parasite was placed on in the 99% lactophenol for 24 hours and identified at genus level.

The fillet was digested in the pepsin HCl artificial digestion method is used to harvest any encysted larva in the fish muscle. Artificial digestion solution was prepared from 10gram of pepsin powder, 16mL of 25% HCl in 2 litres of tap water prewarmed at 42^0C in water bath. Briefly fish flesh was cut in to 1-2milimeter of small pieces using sharp knife. One-hundred-gram small piece of individual fish flesh was placed in 3liter beaker. The digestion solution was fully submerged the flesh; the beaker was covered with aluminium foil and heated on magnetic stirrer at 40^0C for 4 hours to fully homogenise the tissue sample. We turned off the magnetic stirrer once observing the dis-appearance of any visible insoluble tissue. The sample was strained by laboratory sieve with approximate pore size of 70-100μm. The content retained on the sieve was examined for any larvae of nematode under stereomicroscope. The collected worms were counted, recoded and preserved in 70% ethanol (Paperna, 1996; Klinger and Floyd, 1998; Roberts, 2001; Roy, 2002; Amlacher, 2005; Pouder *et al*., 2005; Fraulo et al., 2014).

The morphological characteristics of anterior and posterior parts, including boring tooth, the ratio of ventricular appendix to intestinal caecum and the morphology of their tail were referred for

characterization. This method was specifically optimized for the recovery of infective larval nematodes (e.g., L3 stages of Anisakidae) from the musculature is relevant here to show why this muscle digestion is necessary (Mattiucci et al, 2017). The taxonomic identification of parasites was limited to genus level as the fish harbors mostly larval stages of many nematodes and trematode (Poulin and Presswell, 2022; Hoffman, 2019). The helminthic parasites were identified using parasitological and morphologically approach using standard identification keys and pictorial guide (Pouder et al., 2005).

Prevalence and intensity of the parasite

The prevalence report is calculated considering multiple parasite infestation from single host i.e, one fish could be infected by different parasite groups or free from any parasite. The prevalence and intensity of the parasite was calculated according to the formula (El-Shahawy et al., 2017).

Accordingly,

$$\text{Prevalence}=\frac{\text{Number of individual of a host infected with a particular parasite species}}{\text{Number of hosts examined}}\text{X100}$$

$$\text{Mean intensity}=\frac{\text{Sum of individuals of parasite species in a host sample}}{\text{Total no.of infected individuals of the host in the sample}}$$

$$\text{Mean abundance}=\frac{\text{number of collected parasite}}{\text{Total number examined fishes}}$$

Data Management and Analysis

All collected raw data was entered into a Microsoft Excel database system and imported to be analysed using SPSS statistical software (version-17). Accordingly, descriptive statistics including frequencies, percentages, mean intensity and abundant intensity were presented in tubular form. The chi-square test (χ^2) was used for a possible explanation of associations between the prevalence of parasites and the selected variables (body weight, length, sex, and fish species) for the sampled fish. P-values less than 0.05 were taken as statistically significant.

RESULT

Prevalence of fish parasite in relation to sex, body weight, total and standard length

During the sampling period, 265 fishes belonging to *O. niloticus* (tilapia) (n=135), *B. intermedius ('nech asa'*) also known as *Labeobarbus intermedius* (n=87), *C. gariepinus* (African catfish) (43) were sampled (Figure 1A, 1B, 1C). The total length of the fish ranges from 10cm to 80cm and the total weight ranges from 100g-2Kg.

Prevalence, mean abundance and mean intensity of parasites in fish

Of a total of 265 examined fish, 234 (88.30%) were found infested by at least one parasite group (Figure 2-8). The highest level of parasite infestation was reported in *C. gariepinus* (93.10%) followed by *B. intermedius* (86.05%) and *O. niloticus* (85.93%). The minimum and maximum mean intensity of 5.37 and 8.08 is recorded in *C. gariepinus* and *B. intermedius* respectively. The lower and higher mean abundance of the parasite is also recorded in *C. gariepinus* and *B. intermedius* respectively. The variations in parasite infestations by fish species were not statistically significant ($\chi2 = 2.893$; df = 2, P=0.06) (Table 1). *C. gariepinus* had the lowest mean intensity (MI = 5.37±2.14), and *B. intermedius* had the highest (MI = 8.08±3.21).

Prevalence, mean abundance and mean intensity of parasites in fish

The result indicated that 56.3% of male and 43.7% of female in *O. niloticus*, 52.87% male and 47.3% female in *C. gariepinus*, and 55.81% male and 47.13% female in *B. intermedius* were affected by one or more parasites.

Table 1: Prevalence of parasitic infestation with respect to fish species studied

Species of fish studied	No. of fish affected	Number of parasites from each fish	Total parasite collected	Prevalence (%)	Mean intensity	Mean abundance	χ2	*P- value*
***O. niloticus* (n=135)**	116	6-9	788	85.93	6.79	5.84		
***C. gariepinus* (n=87)**	81	4-11	642	93.10	7.92	7.37	2.89	0.24
***B. intermedius* (n=43)**	37	5-10	318	86.05	8.59	7.40		
**Overall (N=265)**	**234**	**4-10**	**1748**	**88.30**	**7.77**	**6.87**		

The smallest body weighted fishes are highly affected by one or more parasites (90.37% in 100-300g, 75.86% in 190-400g and 79.09% in 100-200g body weight in *O. niloticus*, *C. gariepinus*, and *B. intermedius*), respectively. This body weight class is the lower body weight in respective of fish species. Conversely, the parasite infestation increases as body length of the fish host increase. Accordingly, 70.37%, 95.40% and 93.02% of *O. niloticus*, *C. gariepinus*, and *B. intermedius* respectively are infected by fish parasites at the larger standard body length. The distribution of parasites with sex, body weight, and the total and standard length of *O. niloticus*, *C. gariepinus* and *B. intermedius* fish showed not statistically significant association (Table 2).

Table 2: Parasitic infestation in relation to sex, bodyweight, total and standard length of fishes

**Species of Fish studied**	**Variable category**		**Number of examined**	**Number of fishes infected by parasites**	**Prevalence (%)**	**χ2**	***p-value***
***O. niloticus* (n=116)**	Sex	Male	76 (56.3)	64	84.21	0.423	0.515
		Female	59 (43.7	52	88.14		
	Body Weight (gram)	100-300	122 (90.37)	105	86.07	2.382	0.304
		301-500	11 (8.15)	10	90.91		
		>500	2 (1.48)	1	50.00		
	Total Length (in centimetres)	10-16	32 (23.70)	27	84.38	0.289	0.865
		17-25	92 (68.15)	79	85.87		
		>25	11 (8.15)	10	90.91		
	Standard Length (in centimetres)	<10	4 (2.96)	3	75.00	0.757	0.685
		10-15	36 (26.67)	30	83.33		
		>15	95 (70.37)	83	87.37		
***C. gariepinus* (n=81)**	Sex	Male	46 (52.87)	42	91.30	0.493	0.483
		Female	41 (47.13)	39	95.12		
	Body Weight	190-400	66 (75.86)	62	93.94	0.609	0.737
		401-2000	19 (21.84)	17	89.47		
		>2000	2 (2.29)	2	100.00		
	Total Length (in centimetres)	28-50	85 (97.70)	80	94.12	5.923	0.052
		51-75	2 (2.29)	1	50.00		
		>75	0 (0.00)	0	0.00		
	Standard Length (in centimetres)	<25	4 (4.59)	3	75.00	2.140	0.343
		25-70	0 (0.00)	0	0.00		
		>70	83 (95.40)	78	93.98		
***B. intermedius* (37)**	Sex	Male	24 (55.81)	21	87.50	0.096	0.757
		Female	19 (44.19)	16	84.20		
	Body Weight	100-200	34 (79.07)	31	91.18	4.155	0.125
		201-460	7 (16.28)	5	71.43		
		>460	2 (4.65)	1	50.00		
	Total Length (in centimetres)	15-20	3 (6.98)	2	66.67	1.027	0.598
		21-26	31 (72.09)	27	87.09		
		>26	9(20.93)	8	88.89		
	Standard Length (in centimetres)	<10	1 (2.33)	1	100.00	2.397	0.302
		10-20	2 (4.65)	1	50.00		
		>20	40 (93.02)	35	87.50		

Prevalence of parasitic infestations with respect to anatomical locations

A total of 265 fish were examined, including *Oreochromis niloticus* (n = 135), *Clarias gariepinus* (n = 87), and *Barbus intermedius* (n = 43), with an overall infection/infestation rate of 88.3% (n = 234). In total, 1,748 parasites were collected and classified into nematodes, digenean trematodes, monogeneans, myxozoans, crustacean ectoparasites, and leeches. The most common nematodes were Contracaecum spp., particularly prevalent in *C. gariepinus* (50.57%). Digenean trematodes included Clinostomum, Euclinostomum, Diplostomum, and encysted metacercariae, with the latter reaching 68.97% in *C. gariepinus*. Monogenean infections with *Dactylogyrus* spp. occurred mainly in *O. niloticus* (28.15%). *Myxobolus* spp., leeches, Argulus, and Dolops species were also recorded across hosts. Mean parasite intensity was 8.59 in *B. intermedius*, 7.92 in *C. gariepinus*, and 6.79 in *O. niloticus*, while mean abundance was 7.40, 7.37, and 5.84, respectively.

Clinostomum species (figure 2 in the annex 1) were the dominant trematodes in *O. niloticus* with a prevalence of 65.19% infecting brachial cavity, pericardial cavity, peritoneal and ocular cavity, followed by *Euclinostomum* species (figure 3 in the annex1) with 34.07% which infects kidney, liver and brachial cavity. *Dactylogyrus* species (figure 4 in the annex 1) from the gill and *Diplostomum* species (figure 5) from the brain tissue also showed higher prevalence, accounting 28.15% and 24.44%, respectively. *Contacaecum* species (figure 6) one of the zoonotic anisakidae infecting 26.67% of *O. niloticus* species (Table 3). *Digenean encysted metacercaria* 68.97%, *Contacaecum* species 50.57% and *Diplostomum species* 47.13% infecting musculature, mesentery and brain tissues respectively are the dominant helminths infecting *C. gariepinus* groups. *Contracaecum* species (37.21%) and *Dactylogyrus* species (62.79%) are the dominant parasites affecting *B. intermedius* infecting mesentery and gills respectively. The dominant helminth in *C. gariepinus* was recorded as digenean encysted metacercaria (68.97% prevalence). Identification to genus level was not possible due to heavy encapsulation within host tissue, which obscured key morphological features required for precise taxonomic determination. Given the high intensity and overlapping morphology of the cysts, these parasites were conservatively categorized as digenean metacercariae. In contrast, *O. niloticus* harboured trematodes such as *Clinostomum* and *Euclinostomum*, which were identifiable based on well-preserved sclerotized structures.The common ectoparasites infecting fish species in Lake Hawassa are Argulus species (figure 7) and *Dolops* species (figure 8 ‘a’ and ‘b’ in annex 1) (Table 3).

Table 3: Prevalence with respect to the recovered parasites (n=number fishes sampled in each group).

Variables	Site of localization	Parasite species reco vered	Number of infected fish	Prevalence (%)
***O. niloticus* (n=135)**	*Clinostomum* species	Branchial cavity, pericardial cavity, between cranium and pharyngeal teeth, ocular cavity	88	65.19
	Euclinostomum species	Kidney, liver, branchial cavity	46	34.07
	Contracaecum species	Heart, pericardial Cavity	36	26.67
	Diplostomum species	Brain tissue	33	24.44
	Dactylogyrus species	Gill	38	28.15
	Myxobolus species	Intestinal contents	20	14.81
	Leeches	Skin, gills, mouth	16	11.85
	Argulus species	Skin, fins	9	6.67
	Dolops species	Gill, fins	5	3.70
***C. gariepinus* (n=87)**	*Digenean encysted metacercaria*	Musculature	60	68.97
	Contracaecum species	Mesentery	44	50.57
	Diplostomum species	Brain tissue	41	47.13
	Unidentified parasite	Mesentery	1	1.15
	Dactylogyrus species	Gill	41	47.13
	Encysted cestode larvae	Stomach	5	5.75
	Leeches	Skin, gills, mouth	7	8.04
	Argulus species	Skin, gill, fins and oral cavity	18	20.69
	Dolops species	Skin, gill, fins, Oral cavity	38	43.68
***B. intermedius* (n=43)**	*Contracaecum* species	Mesentery	16	37.21
	Unidentified parasite	Mesentery	2	4.65
	Dactylogyrus species	Gill	27	62.79
	Leeches	Skin, gills, mouth	5	11.63
	Argulus species	Skin, fins	3	6.98
	Dolops species	Skin, fins	4	9.30

Distribution, location and number of ectoparasites on the fishes sampled.

Contracaecum species is the most common parasites found on *O. niloticus with* 373 total number of parasite collected from 224 infected fishes with mean intensity of 3 parasites per fish. Different parasites have different affinity for the body of the fishes where the external parasites mostly affecting gills, skin and gills. *Leeches* are also found infecting all the three fishes studied (Table 4).

Table 4: Anatomical distribution of the different parasites

**Type of parasite**	**Parasite group**	**Anatomical location of infection**	**Number of fish infected**	**Number of parasites collected**
***Clinostomum* species**	Trematode	Branchial cavity, pericardial cavity, between cranium and pharyngeal teeth, ocular cavity	88	255
***Euclinostomum* species**	Trematode	Kidney, liver, branchial cavity	46	136
***Contracaecum* species**	Nematode	Heart, pericardial cavity	124	373
***Diplostomum* species**	Trematode	Brain tissue	74	200
***Dactylogyrus* species**	Trematode	Gill	106	276
***Myxobolus* species**	Protozoa	Intestinal contents	20	88
Digenean encysted metacercaria	Trematode	Musculature	60	189
**Encysted cestode larvae**	Cestode	Stomach	5	19
***Argulus* species**	Crustaceans	Skin, fins	30	79
***Dolops* species**	Crustaceans	Gill, fins	47	96
**Leeches**	Hurindae	Skin, gills, mouth	28	34
**Unidentified internal parasite**		Mesentery	1	3
**Total collected parasites**				**1748**

Discussions

The commercially important fish species: O. *niloticus, C. gariepinus,* and *B. intermedius* from the Hawassa lake are known to be infected with parasites from nematodes, cestodes, trematodes, crustaceans, protozoan families and leeches (table 1-4, figure 2-7). Some of these parasites are known zoonotic pathogens to humans (Ziarati et al., 2022). The majority of animal parasites are found in fish and even apparently health wild fish have some parasites burdens (Petty et al., 2022).

Parasitic diseases are a major constraint to the sustainable development of aquaculture worldwide (Shafiq et al., 2023). In Ethiopia, endoparasites are commonly reported in fish from Lake Awasa, Lake Lugo, and Lake Tana (Gebremedhin et al., 2020). Globally, parasites are recognized not only as pathogens but also as ecologically significant organisms that can impair host growth and reproduction, including causing parasitic castration (de Montaudouin et al., 2012; Marcogliese, 2004; Poulin et al., 2014; Sures and Nachev, 2022). Among aquatic diseases, parasitic infections are the most prevalent, accounting for approximately 54% of cases, compared to 22% attributed to bacterial infections (Okon et al., 2024).

The current prevalence of fish parasites was found to be 88.30%, differing with fish species, sex, body weight, standard length. This result agreed with Mitiku, (2017) who indicated 83.4% prevalence of parasites from central Ethiopia from freshwater culture and capture. Similar prevalence report of 77.4% to 88.3% was reported in Victoria Lake (Outa et al., 2021), Kenya, 88.2%-91.2% by Mgbemena et al., (2020) from Nigeria. The current finding is found greater than earlier reports of 79% by Zekarias and Yimer, (2007) at Lake Hawassa, 69.25% by Tilahun and Behonegn, (2017) at Lake Ziway, 66.3% by Gulelat *et al*., (2013) at Koka reservoir.

The present prevalence (88.30%) was markedly higher than previous reports from several Ethiopian and regional water bodies, including Lakes and farms Taita Taveta county of Kenya (52.3%), Lake Hashange (37.6%) (Tesfaye et al, 2024), Lake Baringo (34.8%) (Kiprono, 2017), Jimma (30.9%)(Gebreegziabher et al., 2020), Lake Chamo (21.4%)(Kaba et al., 2024), and Lake Ziway (20.8%)(Bekele and Hussein, 2015). Similarly, lower prevalence rates were reported from Taita Taveta County, Kenya (37.6%), and Kiri Reservoir, Nigeria (37.5%) (Vandi and Daniel, 2023). These findings indicate that the current study area exhibits comparatively elevated infection levels relative to earlier investigations. Furthermore, higher prevalence reports of 93% was reported from Midmar reservoir, Adwa, Northen Ethiopia by Tesfay et al, (2024) in *O. niloticus*.

The difference in parasite prevalence among different studies may be attributed to ecological, methodological, and host related factors. difference in water quality, agroecological and seasonal variation can influence parasite survival, intermediate host, and transmission dynamics. Variation in fishing species composition, feeding habitats, age, sex, body structure, and the immune status may also contribute for differing susceptibility (Turner et al., 2021).

The prevalence, mean intensity and mean abundance of the parasites vary with species of the fish's host. Accordingly, prevalence rate of 85.93%, 93.10% and 86.05% is registered in O. *niloticus, C. gariepinus,* and *B. intermedius* respectively. In the current finding, the prevalence rate is

slightly higher in *C. gariepinus* as compared to O. *niloticus* and *B. intermedius*. Similar finding was reported by Tesfaye *et al*, (2020) in which the rate of parasitic infection is higher in *C. gariepinus*. Additionally, *C. gariepinus* species are among the most frequently infected fish species, according to Gulilat et al, (2013). Trute et al., (2023) also reported the presence of over 107 metazooan parasites are known to infect *C. gariepinus* in Africa with well characterized life cycle and general morphology.

Mean intensity and apparent parasite density varied among fish species, with the highest values recorded in *B. intermedius* (8.59; 7.40), followed by *C. gariepinus* (7.92; 7.37) and *O. niloticus* (6.79; 5.84). Similar findings have been reported by Oztruk and Alutnel (2006), who observed that parasite intensity and prevalence differ according to host size. These variations are influenced by abiotic factors such as water temperature and host-related factors including behaviour, age, sex, resistance, and mortality (Bakke et al., 2002; Ozer et al., 2004; Khidr et al., 2012).

The higher prevalence of internal helminths in the current study could be attributed to a number of factors, including the lack of an effective waste disposal and management system, which led to the processing of post-harvest fish and the disposal of the waste (scraps and gastrointestinal contents) into the lake and its shorelines; environmental pollution from detergents used for washing clothes on the lake shore; and some birds that could serve as intermediate or final hosts for helminths. This shows that the host-parasite interaction and changes in abiotic factors, including sewage, irrigation, sanitation, and flooding, frequently causes the distribution of fish parasites to vary from one habitat to another, and that leads to environmental stresses that impair the host's immune system (Tibebe et al, 2023). Compared to mammals, fish have a lower immune response to foreign proteins (Pradipta et al, 2012; Ardelli and Woo, 1997). Several parasites are known to alter the behaviour of their hosts, predisposing them to predation (Labaude et al, 2015). The difference in prevalence from different lakes in Ethiopia and other parts of the world may be attributed to the difference in behaviour of different fish species, the quality of water, the presence of other stressors such as pollution and climate change (Okon et al., 2024). The increase in global temperature leads to fluctuation in aquatic environment whereas the elevated water temperature could potentially compromise the fish immune system leading to fish more vulnerable to disease conditions (Grossart et al., 2019; Kaur et al., 2022).

In the present study, parasite prevalence was higher in males than females across *O. niloticus*, *C. gariepinus*, and *B. intermedius*. These values were lower than reports from Nigeria (Mgbemena et al., 2020) but higher than those from Lake Hashenge, Ethiopia (Tesfaye et al., 2017), and were comparable to findings from Nigeria and Upper Egypt. Although some studies report higher infection in females, this has been attributed to behavioural factors such as increased shoaling and prolonged parasite exposure (Simková et al, 2005; Abd-ELrahman et al., 2023).

In the current finding the presence of parasites varies with body weight and standard length. Accordingly, the lower body weighted fishes are highly affected by one or more parasites. This finding is consistent with earlier finding that indicate there is a general trend that smaller fish or those with poor body condition may be susceptible to parasitic infections although it is not universally true. This can be due to several factors including inability to allocate enough defence

and behavioural adaptations in lower body condition fishes (Abd-ELrahman et al., 2023; Hasegawa et al., 2025). How ever several biological parameter or ecological processes determine which types of parasites are more likely to correlate closely with host size (Poulin, 2005). In other terms larger fish usually have larger fish population and wider range of parasitic species due to the availability of resources and greater capacity to harbor variety of parasites. Smaller fish however may be more vulnerable to specific larval stages of parasites potentially due to their exposure to predation (Leung, 2022). Although parasite reduces host health, parasite infection can occur due to compromised host health (Hasegawa et al., 2025). Poulin, (2014) indicated host size (and age) is generally known to be positively associated with parasite species richness and infection levels in host individuals. Nikolaev *et al*, (2006) also concluded that as age increase there is higher chance of accumulating parasites during their lifetime. In the current finding it is found difficult to harvest and investigate old age and large body weight fishes since illegal fishing along with other abiotic factors are highly practiced and may posing threat including decline in number of fish or yield of the lake Hawassa. The research team has clearly observed the illegal fishing practice and the practice of eviscerating fish along the shore of their lake which increase the risk of re-infection.

Identification of parasites with significant host and tissue specificity is made much easier by knowledge of fish hosts, while other parasites are detected due to their widespread presence and lack of host specificity (Petty et al., 2022). In line with this, *Contracaecum* was the most frequently encountered nematode parasite found in the current study. It is found 26.67% in *O. niloticus* to 50.57% in *C. gariepinus.* This finding is lower than previous reports by Zekarias and Yimer, (2007) in Lake Hawassa, who reported 39.67% in *O. niloticus*, 80.5% in *C. gariepinus*, and 36% in *B. intermedius*. Moreover, Kaba et al., (2024) reported lower prevalence of 21.4% from *O. niloticus* and *Lates niloticus* from Chamo Lake, Arbaminch. However, it is higher as compared to Gulelat *et al.* (2013) at Koka reservoir, 24.82% in *C. gariepinus* and 16.49% in *B. intermedius*. This result suggests that the distribution of *Contracaecum's* parasites can vary from one habitat to another due to host parasite relationships and changes in water quality parameters. Recently *Contracaecum bancrofti* a unique Australian species and warming the fish tissue leads to emergence of small *Contracaecum* larvae <1 mm from intestinal tissue (Shamsi *et al*, 2018). *Contracaecum* are economically important parasites with zoonotic significance where human infection through ingestion of infected tissue is the major route (Kaba et al., 2024). Adult *Contracaecum* spp. are found in the stomach of marine mammals or piscivorous birds, and larval stages infect a wide range of invertebrates and fish species (Shamsi et al, 2017). Study from lake Chamo show that a very high proportion of people (82%) consume raw fish especially locally preferred species such as *Oreochromis niloticus* and *Lates niloticus*, and that consumer awareness of fish-borne zoonoses is generally low (Kaba et al., 2024). Several other studies have also reported human infection due to *Contracaecum* larvae following consumption of infected fish (Shamsi *et al,* 2018; Shamsi and Barton, 2023). In lake Hawassa area similar pattern was practiced where humans eat fresh uncooked fish which might be infected with zoonotic parasite.

The host distribution of *Clinostomum* species was discovered in the current study to be much higher than previous studies by Gulelat *et al.* (2013), Reshid *et al.* (2015), and Tesfaye *et al.* (2017), who revealed a prevalence of 27.39%, 18.8%, and 15.2% in Lake Abaya, Koka Reservoir, and Lake Hashange, respectively. However, the prevalence of this parasite is lower than that found by Zekarias and Yimer, (2007) who reported 75.7% prevalence from Lake Hawassa. The variations may result from variations in the physiochemical characteristics of water bodies and the intermediate hosts of the parasite. In other way another digenean fluke, *Euclinostomum* species from *O. niloticus* were detected in the kidney, liver, and branchial cavity. This finding was in line to previous reports from Ethiopian lakes and water bodies.

A prevalence of 30% was reported by Zekarias and Yimer (2007) from Lake Hawassa, whereas much lower rates were documented in Koka Reservoir (2.73%) and Jimma (3.9%). In contrast, Mitiku (2017) recorded a higher prevalence of 49.35% for *Euclinostomum*. These variations are likely influenced by differences in parasite–host life cycles and environmental conditions affecting transmission dynamics.

Moreover, Khan et al, 2018 reported that prevalence of 70-92% metacercarial infection belonging to *C. complanatum* was reported concluding the seasonality of the infection. He stated that the high prevalence of infection of the parasite during summer may be because of the time of emergence of cercariae in high temperature from snail vector and the environment. Digenetic trematodes, or flukes, belonging to the genera *Clinostomum* and *Euclinostomum* infect many fish species all over the world. These parasites are found in freshwater and saltwater habitats worldwide (Senapin et al., 2014). The coexistence of *Clinostomum* and *Euclinostomum* in some fish host could imply the shared intermediate hosts, environmental conditions, or ecological overlap (Choudhary et al,2022)

The *digenetic trematode* *Clinostomum* spp. commonly referred to as yellow grubs when in their larval form have complex and involves three hosts including freshwater snails as intermediate host, other fish or amphibians and piscivorous birds as definitive host. Humans are also accidental hosts if they consume undercooked or raw fish meal.

In fish, encysted metacercaria are not merely superficial, they often embed deeply into critical tissues such as musculature, gills and buccal cavity, and cranial and pericardial regions, cranial and pericardial regions causing necrosis, inflammation, fibrosis and oxidative response which could compromise immune function and metabolism (Salem et al., 2025).

Lower prevalence rate of *Diplostomum* species, 24.44 and 47.13% respectively was found in the brain tissue of *O. niloticus* and *C. gariepinus*. This finding is lower as compared to the previous studies 77.93% of the gill cavity of *O. niloticus* at Lake Baringo, Kenya (Kiprono, 2017);83.3% from cranial cavity of *C. gariepinus* sampled at Tanzania (Mukama, 2008); 100% of the cranial cavity of *C. gariepinus* was from Lake Hawassa (Tadesse, 2009); 77.93% of the cranial cavity of *C. gariepinus* (Gulelat et al., 2013). Significantly lower prevalence rate of 1.03% *Diplostomum* species was reported from the eye of *B. intermedius* from Lake Abaya (Gulelat et al.

(2013). According to the current findings, Degenian encysted metacercaria frequently infect *C. gariepinus* muscle. This finding is in line with the findings of Vidal-Martínez et al. (2012) and Galaktionov et al. (2006), who conclude that these parasites commonly affect fish in coastal marine systems, which exhibit complex life cycles that typically involve snails as first intermediate hosts, macroinvertebrates, such as bivalves and crustaceans, or vertebrates as second intermediate hosts, and fish, birds, or mammals as final hosts.

External parasites have strategic survival reasons to inhibit specific microhabitat on fish body. This specific microhabitat may provide availability of food, reproductive success, enhanced attachment or reduce the pressure of predator (Chew et al., 2024). in the current finding, the crustacean parasites *Argulus* and *Dolops* species were found infecting gills, fins, skin, and oral cavity of the three fish species. This finding is lower as compared to the previous study by Zekarias and Yimer, (2007) in Lake Hawassa, who found 5.33% and 8.33% in *O. niloticus*; 44.5% and 63.5% in *C. gariepinus*, and 6% and 17 in *B. intermedius*. Moreover, Mitiku (2017) reported 7.8% of external parasites from *O. niloticus* at Sebeta ponds. Argus species is more commonly known as fish lice, are member of large groups of branchiuran parasite that infest and cause fish disease. They are crustaceans and crabs, lobsters, and shrimp. There are over 100 species infecting freshwater fishes with *Argulus foliaceus, A. coregoni*, *A. japonicus* common in freshwater fishes (Stecker and Yanong, 2024). According to a study by Trutera et al, (2022) *A. africanus* and *A. rhipidiophorus* are prevalent crustacean parasites in Ethiopian fish, especially those found in Lake Hawassa. *Argulus japonicus* is an alien species that has been introduced globally and is known to damage fish such as *C. gariepinus*, while it is not specifically listed as being present in Ethiopian fishes (Zekarias and Yimer, 2008, Trutera et al, 2022).

Dactylogyrus species was find residing in the gills of fishes. The current prevalence report of 3.7-43.6% was found higher than the reported 3.96% by Mitiku *et al.* (2018) in *O. niloticus* from *Gilgel* Gibe I Dam, and 31% by Borji *et al.* (2012) in common carp in the northeast of Iran from the gill of sampled fish. *Myxobolus* species from the intestinal contents of *O. niloticus* were also found in the current study, which was not reported elsewhere in Ethiopia. How ever the prevalence rate of *Myxobolus* species in the current finding was lower than previous reports by Abdel-Baki *et al*. (2015) in the Nile River, Egypt, and Folefack *et al*. (2019) in Cameroon. Higher prevalence rate of *Myxobolus* infection including two new species was reported from Botswana (Reed et al, 2002).

In addition to the infections caused by parasites and other pathogens, several factors are threatening the quality of Hawassa lake from which human practice along the edge of the lake is the dominant factor. Memberu et al., (2021) have observed that the quality of the Hawassa lake is inadequate for maximum production of fishes and needs mitigation measures to control eutrophication and pollution flow. Anthropogenic activities, illegal fishing practices such as the use of narrow mesh nets, a lack of community awareness regarding fishery management, and a lack of fish stocks are the main conservation issues facing Ethiopian fish and fisheries sector (Chimdo, 2022).

The presence of parasites in edible part of the fish represent a public health threat since the presence of those parasites are responsible for causing infection in humans with significant outcome (Audicana and Kennedy, 2008). Knowledge of how parasites such as *Contracaecum* species, flukes and other pathogenic parasites are transmitted, their reservoir animal, their specific reservoir animals, the specific pathogen involved, directly impacts prevention and control strategy (WHO, 2020). The focus of control methods should be on health education to promote appropriate control measures including, and safely disposing of infected (Fomda et al., 2015). As prevention and control methods for each pathogen varies, however several practices are recognised as effective for reducing the risks at the community and personal level. Safe and appropriate guidelines for animal care in the agricultural sector helps to reduce the potential for food borne zoonotic disease outbreaks through ingestion of animal food or some vegetables. Standards for clean drinking water and waste disposal as well as protection for surface water in the natural environment are important and effective control means (WHO, 2020).

Limitation of the study

This study has not stablished the characterisation of the parasite at species level, and it only focuses describing the parasite at genera level. Moreover, water the impact of water quality on the effect of parasites have also not established

CONCLUSION AND RECOMMENDATIONS

In the current study, internal and external infestations were the predominant cases infecting fish with 88.03%. The parasites recovered belongs to *crustaceans, monogenean, digeneans, nematode, cestode,* and one protozoan and *Hirudinea* having a direct effect on fish health and meat quality and, thus, a direct effect on fish production and the economy. Among these, the Dactylogyrus species was the dominant external parasite followed by Argulus and Dolops species. It is found that parasite intensity and abundance vary with the host fish species. Anatomical and physiological variables of the fish such as body size, age, body weight and gender also found determining different prevalences. Our finding also showed that the common zoonotic nematodes such as *Contracaecum* species and flukes are commonly found in the fishes at lake Hawassa necessitating coordinated efforts of public education. We found that human practice along the shore of the lake is also one of anthropogenic factor influencing fish health and productivity. One of the limitations of this study is that it depends on visual examination rather than digestion methods or incubation techniques in which some small or deeply encysted parasites, early developmental stages, or low-intensity infections were not detected. It highlights that the parasite diversity and prevalence in Lake Hawassa may be even higher than reported, reinforcing the need for further, more sensitive diagnostic approaches in future research.

Based on the above conclusion the following recommendations were forwarded:

- To improve parasite management, it is necessary to investigate and determine how abiotic elements affect the parasite-host interaction.
- Lake Hawassa needs to be kept apart from areas utilised for fishing and pleasure purpose.
- It is necessary to investigate the diversity of parasites over a wide range of hosts and water bodies.
- The wastes products including offals of the fish themselves must be disposed of properly and kept apart from water bodies.
- Molecular detection of each fish parasite to the genetic level needs to be carried out in future as the geographic bias exists in the discovery and genetic characterization of novel parasites.

Limitation of the study

A histopathological diagnosis with greater funding and resources does not support this study. Furthermore, it is necessary to evaluate how the evaluation of water quality affects fish parasites. Another drawback is that parasites can only be identified at the genus level.

Ethical approval and consent to participate

All methods were carried out in "accordance" with relevant guidelines and regulations. Furthermore, all methods are reported in accordance with "ARRIVE" guidelines (https://arriveguidelines.org) for the reporting of animal experiments. Ethical approval was obtained from the Research Ethics Committee of the School of Veterinary Medicine, Wollega University dated 20/04/2023 with minute number SVM.RERC/0023 before starting data collection. Parent/legal guardian provided informed consent on behalf of participants of age below eighteen years whereas oral consent was received from those above eighteen-year fishermen. The Research Ethics Committee of the School of Veterinary Medicine, Wollega University approved all aspects of this study, including the informed oral consent process. Communicative letter was written to Hawassa City municipal and fishery sector leaders explaining the objective of this study. Data was collected after explaining the aims and methodology of the study. During data collection on fish all the welfare of animal was protected. It is assured that information collected was anonymous and that it was only for research purpose.

Data Availability statement: The data that support the findings of this study are available from the corresponding author upon reasonable request.

Conflict of interest: the author declares there is no conflict of interest

Funding importation: No funding was received for this research

Author's contribution: MA and DA collected data from the field, managed and analysed it, and participated in manuscript development and revision; YT, methodology, analysis, writing the original draft and editing final paper; DA, supervision, writing the original draft and editing final paper.

Acknowledgements: the author acknowledges Wollega University, Addis Ababa University and Hawassa University

Figures of fishes and their parasites (figure 1A-8B)

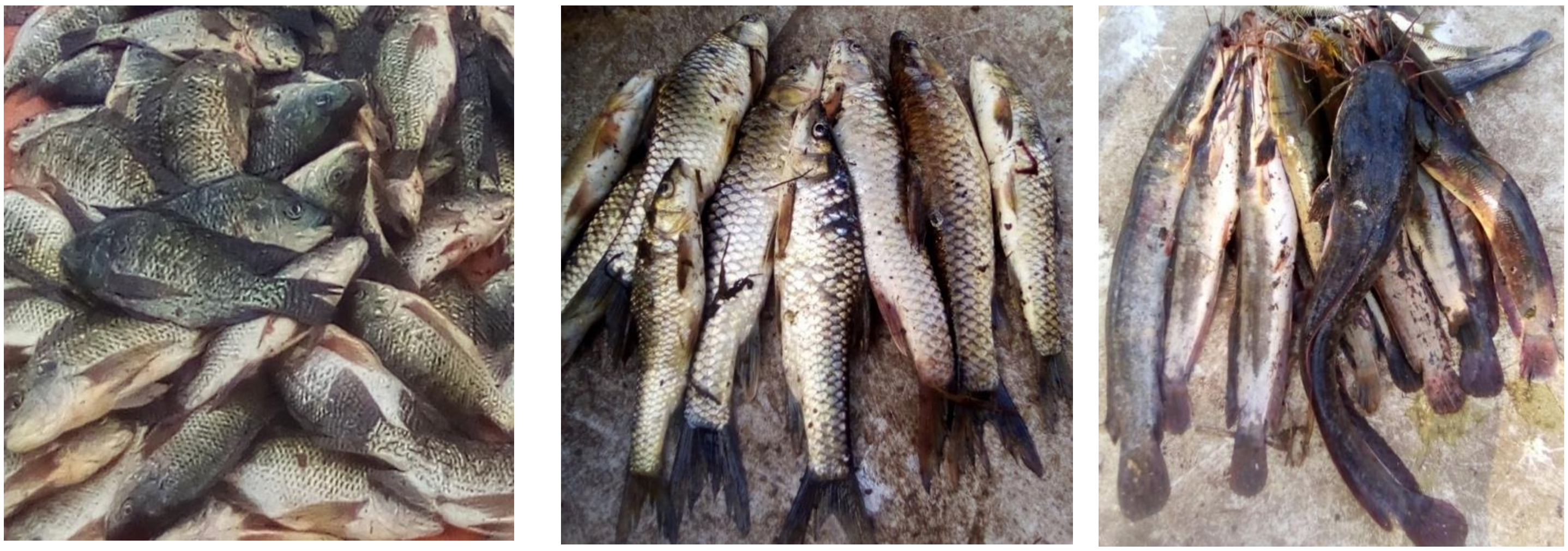

Figure 1: *O. niloticus* (Nile tilapia or Koroso) the first; *Barbus* species, the middle and *C. gariepinus (*Catfish or Ambaza), the third

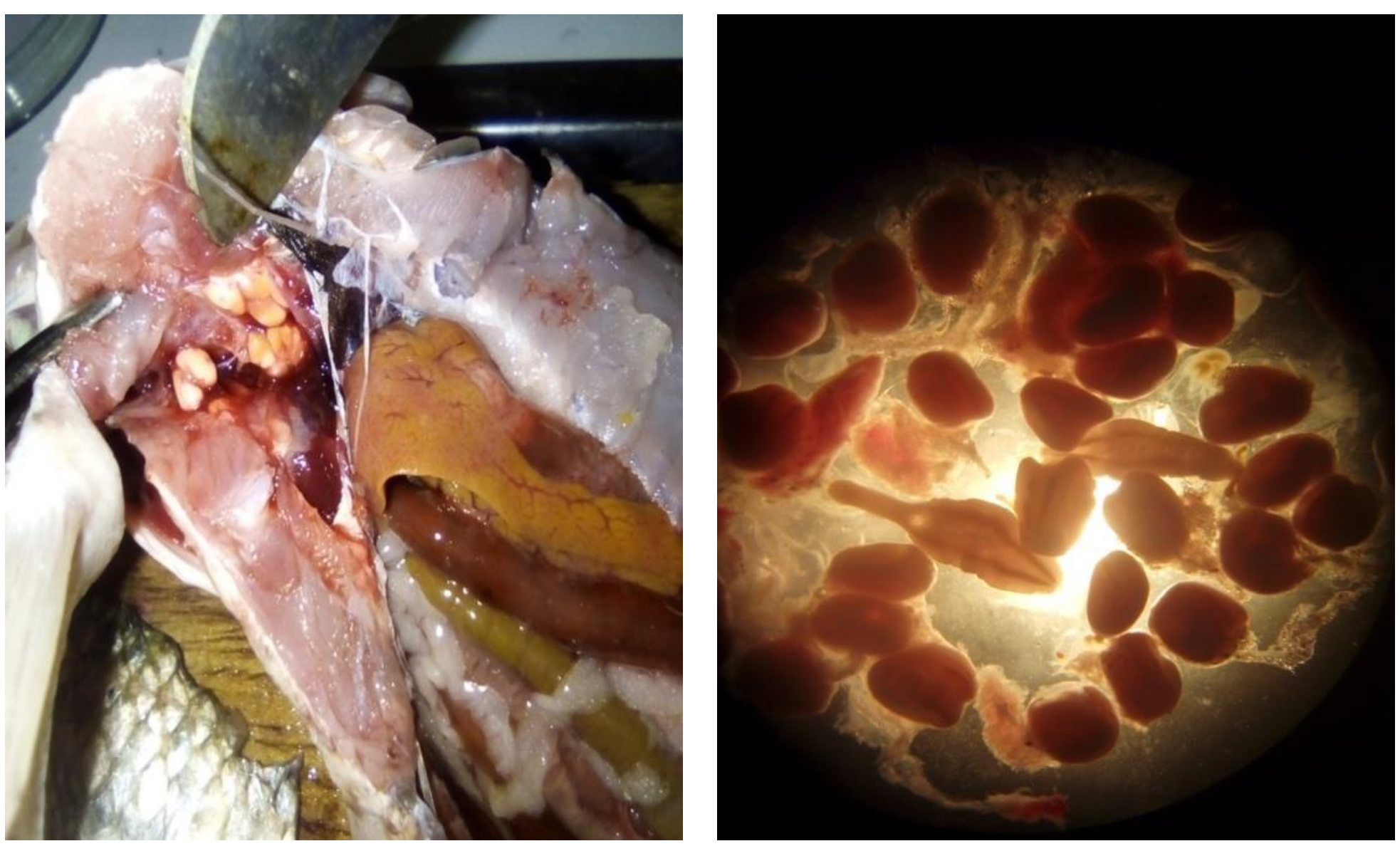

Figure 2: Encysted (left) and Excysted (right) *Clinostomum* species

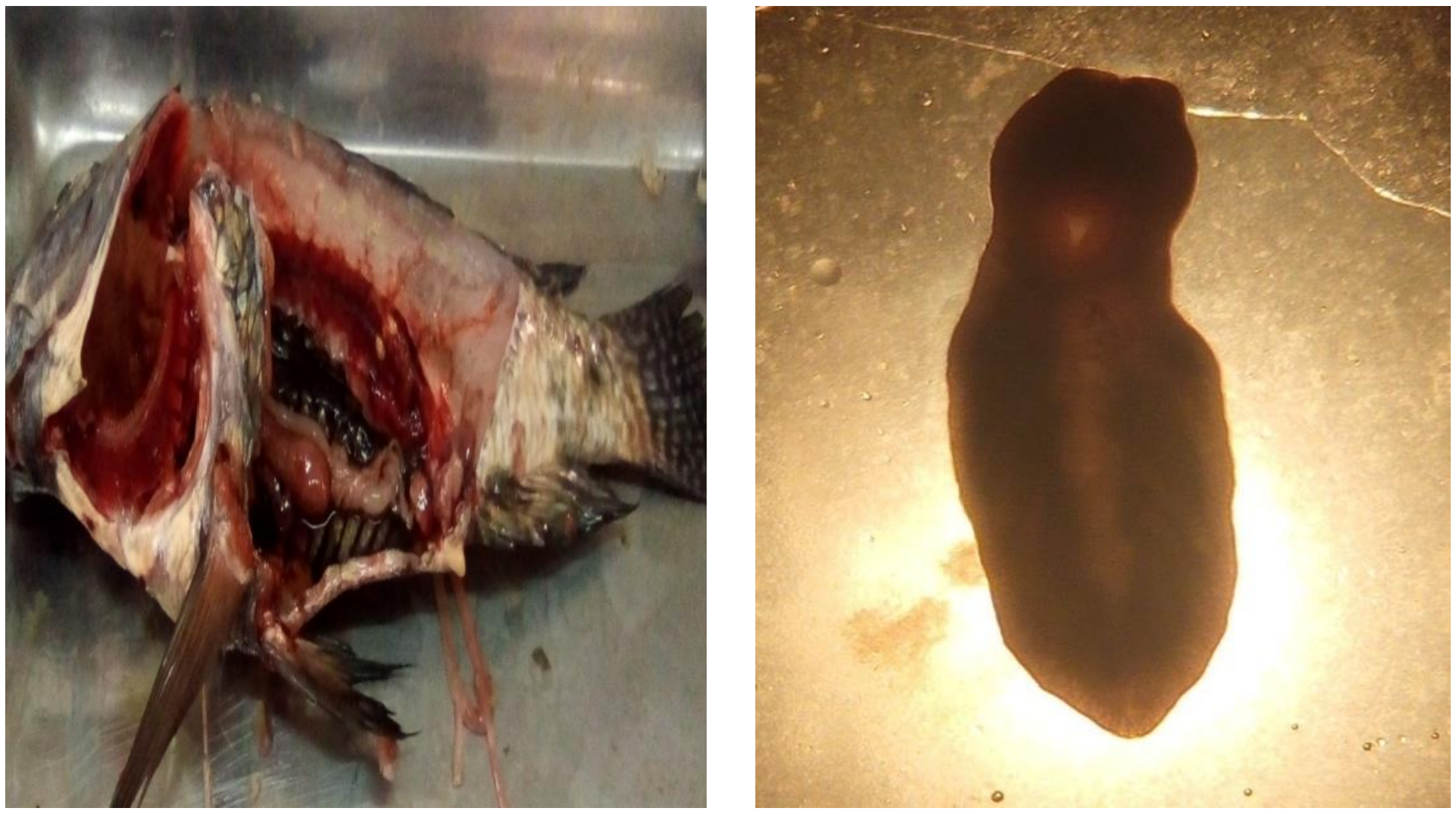

Figure3: Encysted (left) and excysted (right) *Euclinostomum* species on the kidney of *O. niloticus*

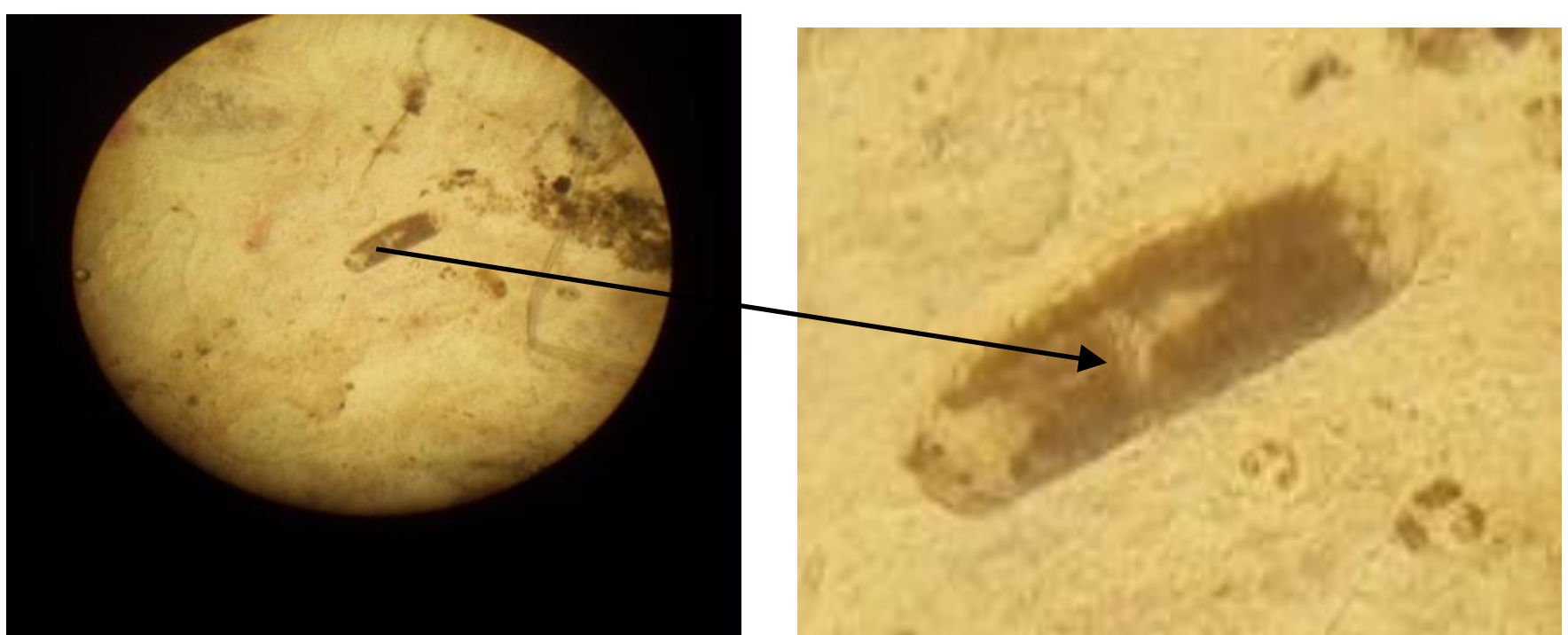

Figure 4: *Dactylogyrus* species from the gill of Nile tilapia *(O.niloticus)*.

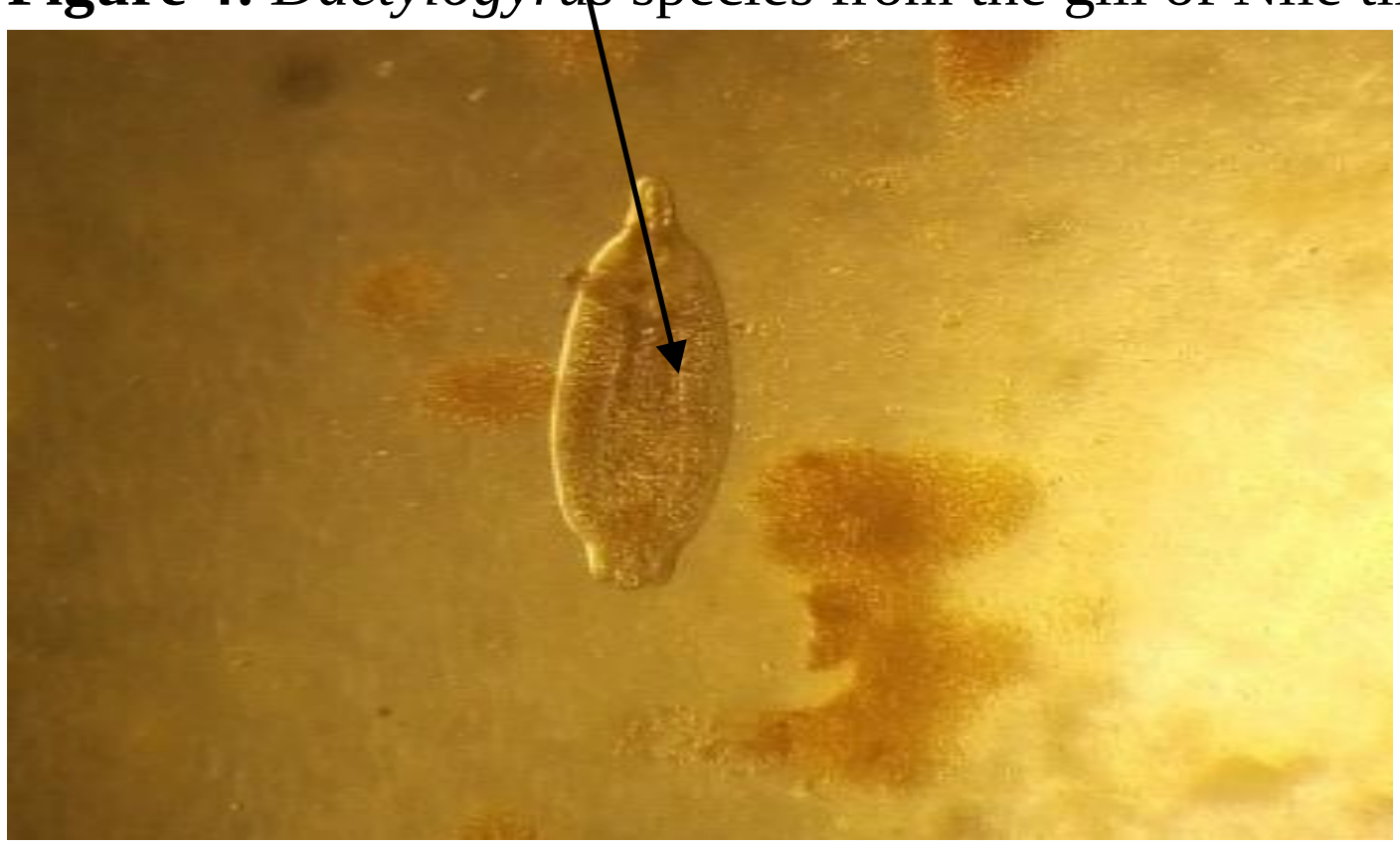

Figure5: *Diplostomum* species from the brain tissue of African catfish

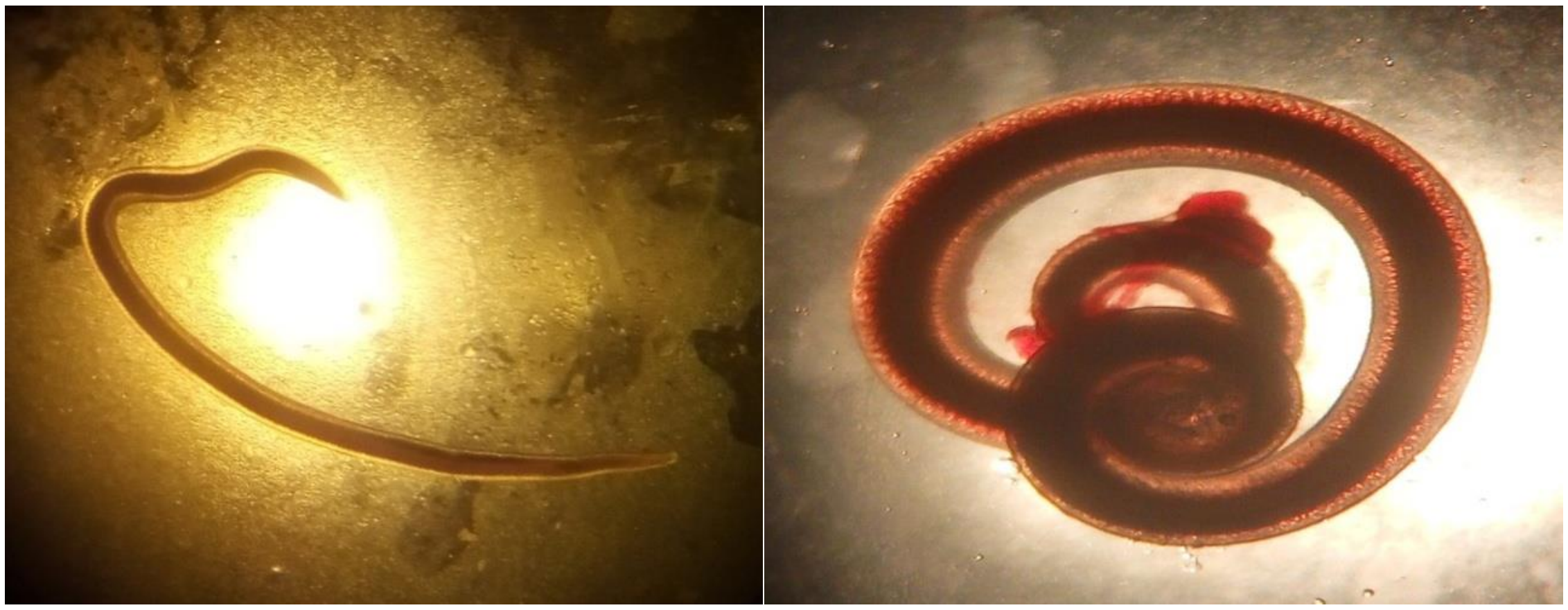

Figure 6a: *Contracaecum* species from the heart of Nile tilapia *(O. niloticus)*.

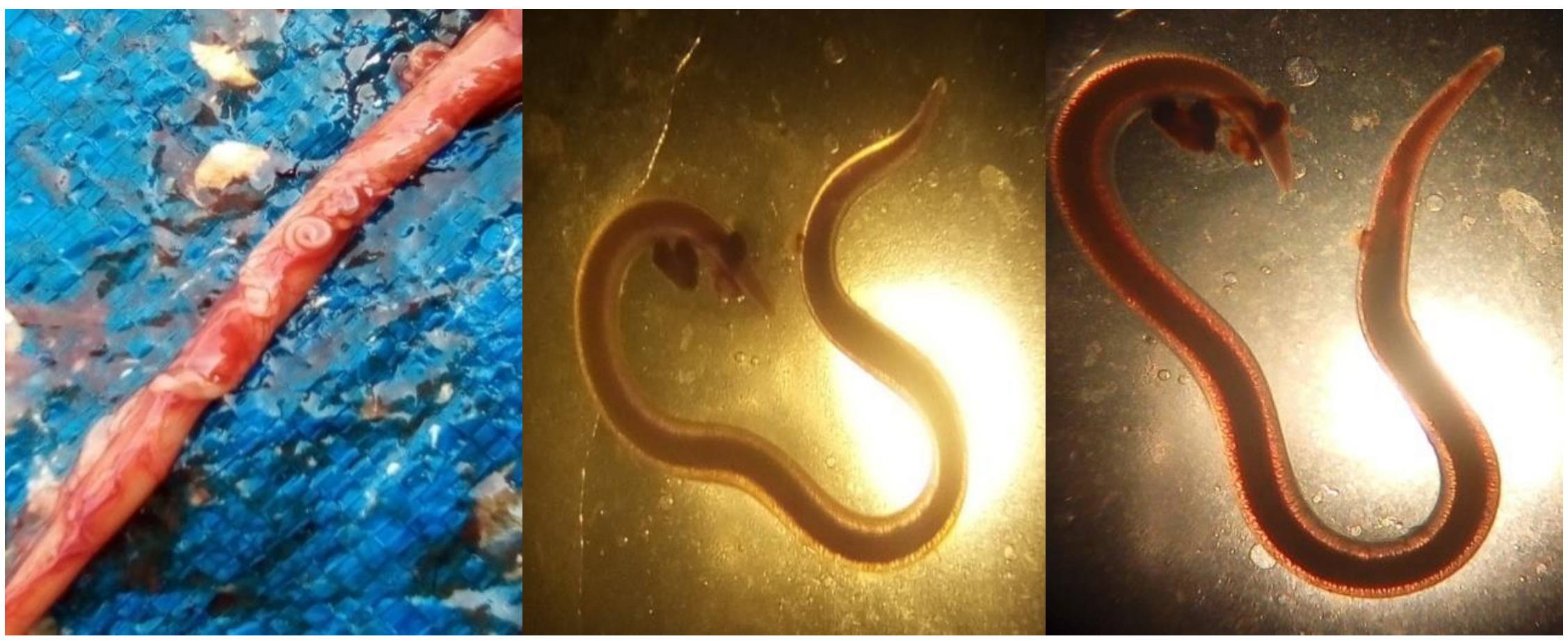

Figure 6b: *Contracaecum* species from mesentery of *C. gariepinus* (African catfish).

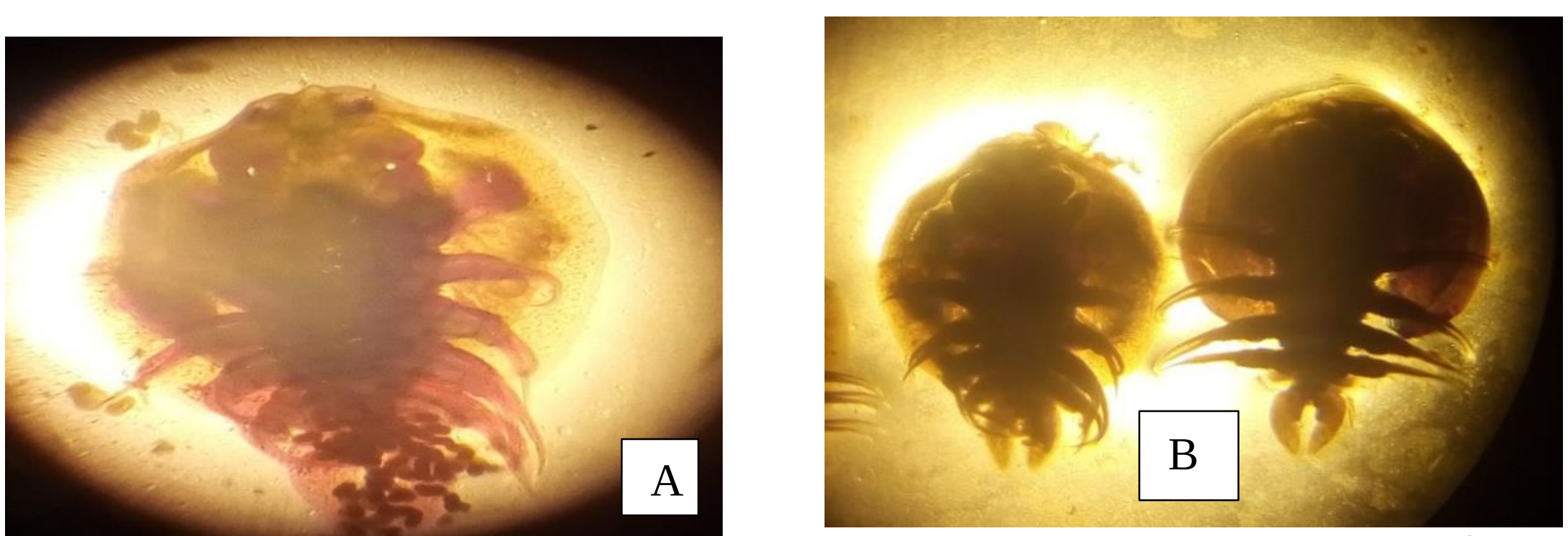

Figure 7: *Argulus* species (A: from the gill B: From fins of *C. gariepinus*)

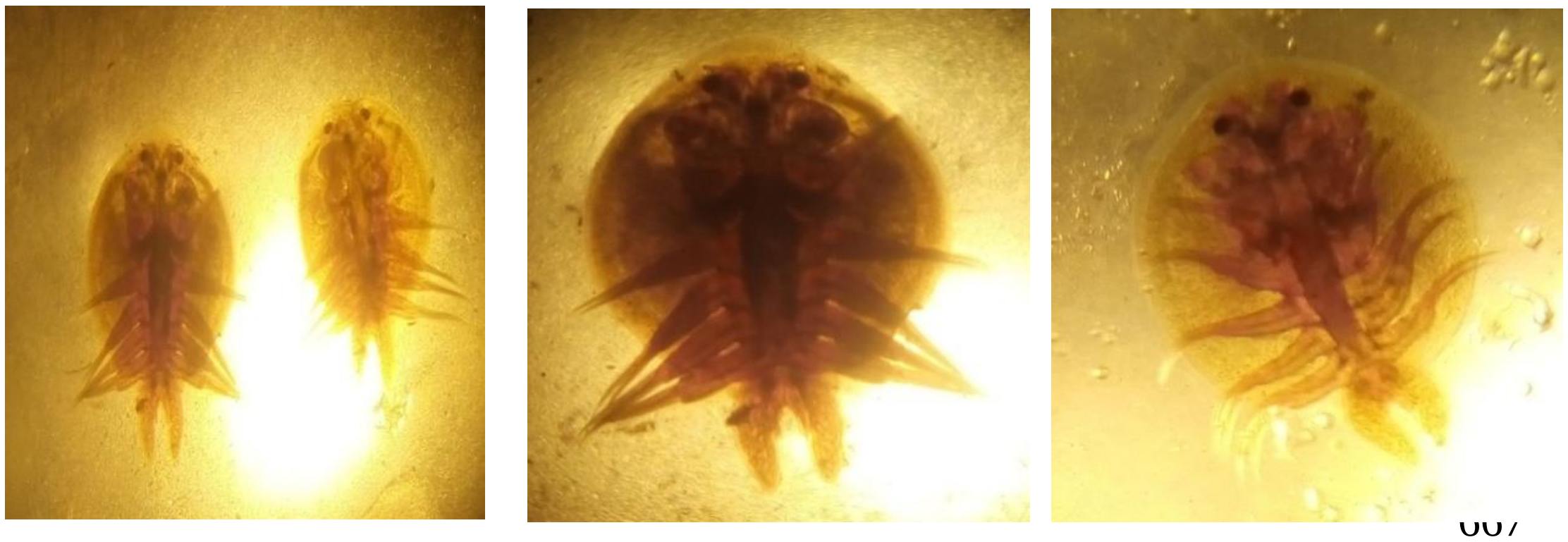

Figure 8a: *Dolops* species from the gill of *C. gariepinus*.

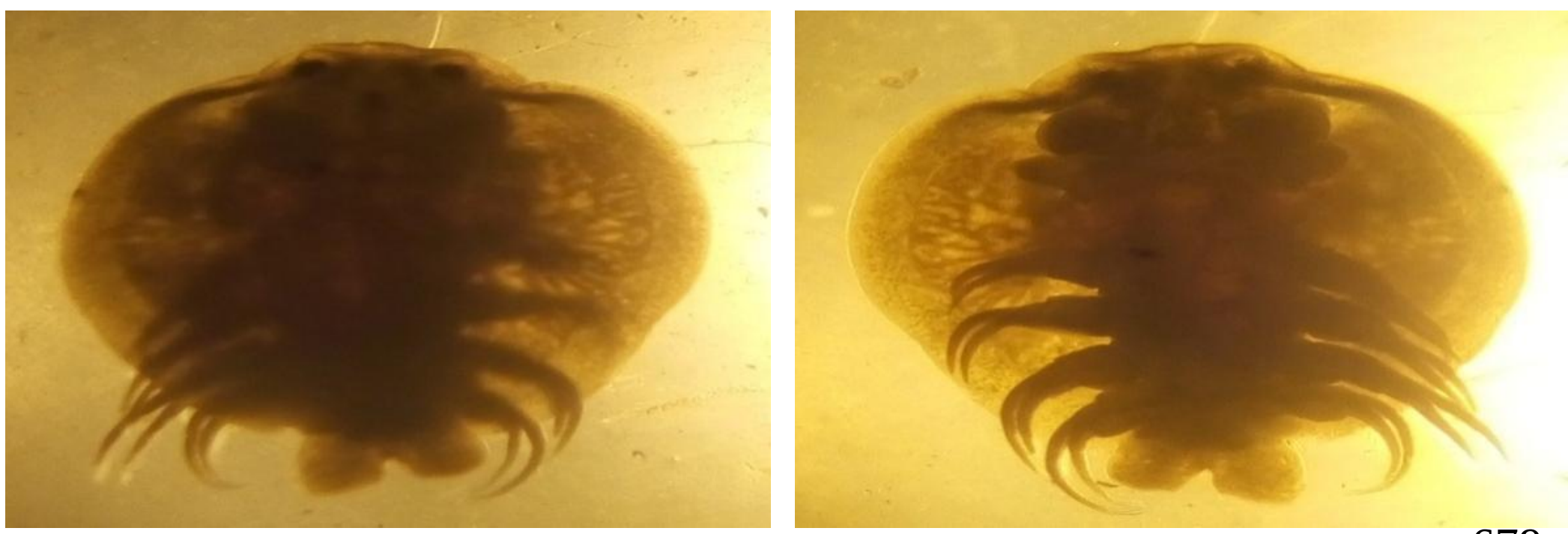

Figure 8b: Dorsal (left) and ventral view (right) of *Dolops* species from the skin of catfish

1. REFERENCES

Abd-ELrahman, S, M., Gareh, A, Mohamed, H,I.H, I., Alrashdi, B,M.B, M., Dyab, A,K.A, K., El-Khadragy, M,F., Khairy Elbarbary, N., Fouad, A,M., El-Gohary, F,A., Elmahallawy, E,K.E, K., Mohamed, S,A.,2023. Prevalence and Morphological Investigation of Parasitic Infection in Freshwater Fish (Nile Tilapia) from Upper Egypt. Animals (Basel), 18;13(6), https://doi.org/doi: 10.3390/ani13061088

Abdel-Baki, A.A.S., Abdel-Haleem, H.M., Sakran, T., Zayed, E., Ibrahim, K.E. and Al-Quraishy, S., 2015. Two Myxobolus spp. infecting the kidney of Nile tilapia (Oreochromis niloticus) in the River Nile at Beni-Suef governorate, Egypt, and the associated renal changes. *Parasitology research*, **114**(3), pp.1107-1112.

Ageng'o, F, A., Waruiru, R, M.,Wanja, D, W., Nyaga, P, N., Hamisi, M, M.,Ndegwa, J, M.,Ali, S, E., ChadagE., Chadag, M, V.,Mbuthia P, G.P, G.,2025. Parasites of Farmed and Wild Tilapine Fishes from Selected Farms and Lake Jipe in Taita Taveta County, Kenya, https://doi.org/10.1002/aff2.70042

Amlacher, E., 2005. Textbook of Fish Diseases. Narendra Publishing House, India, pp 207–292.

Ardelli, B. F. and Woo P. T. K., 1997. "Protective antibodies and anamnestic response in *Salvelinus fontinalis* to *Cryptobia salmositica* and innate resistance of *Salvelinus namaycush* to the hemoflagellate," *Journal of Parasitology*, vol. 83, no. 5, pp. 943–946, 1997.

Audicana, M, T., Kennedy, M, W.,2008. Anisakis simplex: from obscure infectious worm to inducer of immune hypersensitivity. Clin Microbiol Rev.;21(2):360-79, doi:10.1128/CMR.00012-07.

Bakke, T,A.,Harris, P,D.,Cable, J., 2002. Host specificity dynamics: observations on gyrodactylid monogeneans, Int. J. Parasitol., 32, pp. 281-308

Barber, I., Wright, H, A. 2005. Effects of Parasites on Fish Behaviour: Interactions with Host Physiology, Fish Physiology, Academic Press, 24,109-149, https://doi.org/10.1016/S1546-5098(05)24004-9

Bekele, J., Hussein, D., 2015. Prevalence of internal parasites of *Oreochromis niloticus* and *Clarius gariepinus*, journal of aquatic research and development,6(2), *http://dx.doi.org/10.4172/2155-9546.1000308*

Borji, H., Naghibi, A., Nasiri, M.R. and Ahmadi, A., 2012. Identification of Dactylogyrus spp. and other parasites of common carp in northeast of Iran. *Journal of Parasitic Diseases*, **36**(2), pp.234-238.

Boyd, C,E, McNevin AA, Davis RP. The contribution of fisheries and aquaculture to the global protein supply. Food Secur. 2022;14(3):805-827. https://doi.org/doi: 10.1007/s12571-021-01246-9.

Brian, J.I.,2023. Parasites in biodiversity conservation: Friend or foe? Trends Parasitol.; 39:618–621. https://doi.org/doi: 10.1016/j.pt.2023.05.005.

Brye, K, 2023. Aquaculture: A Sustainable Solution for Fish Cultivation and Global Food Security, Journal of Agriculture, 6(4),1-3

Chai, J.Y.; Murrell, K.D.; Lymbery, A.J.,2005. Fish-borne parasitic zoonoses: Status and issues. *Parasitol. Int.* **2005**, *35*, 1233–1254

Chew, X, Z., Cobcroft,J.Cobcroft, J., Hutson, K, S.,2024. Fish ectoparasite detection, collection and curation. Adv Parasitol, 125:105-157. https://doi.org/doi: 10.1016/bs.apar.2024.05.001

Chimdo, A. Review on potential and challenges of aquaculture practice in Ethiopia. *Appl Water Sci* **12**, 218 (2022). https://doi.org/10.1007/s13201-022-01740-1

Choudhary, K., Ray, S., Shamsi, S., Agrawal, N., *2022.* Characterization of *Clinostomum* (Digenea: Clinostomidae) spp. in India. *Parasitol Res* 121, 3083–3089 (2022). https://doi.org/10.1007/s00436-022-07644-y

CNN travel, 2023. Ethiopian food: the 15 best dishes, https://edition.cnn.com/travel/article/ethiopian-food-best-dishes-africa/index.html#:~:text=Eating%20fish%20%E2%80%93%20asa%20%E2%80%93%20in%20Ethiopia,a%20piranha%20on%20your%20plate

Dadebo, E., 2000. Reproductive biology and feeding habits of the catfish Clarias gariepinus (Burchell) (Pisces: Clariidae) in Lake Awassa, Ethiopia. *SINET: Ethiopian Journal of Science*, **23**(2), pp.231-246.

Deng, G, T.2020. Assessment of Factors Affecting Fish Production and Marketing in Gambella Region, Ethiopia. ScientificWorldJournal. 16; 2020:5260693. doi: 10.1155/2020/5260693.

Desalegn, T. and Shitaw, T., 2021.Fishery Resources, Conservation Challenges and Management Strategies in Ethiopia. *Fisheries and Aquaculture Journal*, **12**(3), pp.1a-1a.

El-Shahawy, I.S., El-Seify, M.O., Metwally, A.M., Fwaz, M.M. 2017. Survey on endoparasitic fauna of some commercially important fishes of the River Nile, southern of Egypt (Egypt) Rev. De Med. Vet.; 168:126–134.

FAO, 2018. Food and Agriculture Organization of the United Nations, *The State of World Fisheries and Aquaculture 2018—Meeting the Sustainable Development Goals*; FAO: Rome, Italy, 2018.

Folefack, G.B.L., Kengne, C.M., Dongmo, B.F. and Fomena, A., 2019. Prevalence and mean intensity of Myxobolus spp. Parasitizing Orechromis niloticus in Cameroon. *International Journal of Biology*, **11**(2), pp.35-45.

Food Business, 2025. Ethiopia's Grand Renaissance Dam to increase daily fish production – Ministry of Agriculture, https://www.foodbusinessafrica.com/

Fraulo, P., Morena, C., & Costa, A. (2014). Recovery of Anisakid larvae by means of chloro-peptic digestion and proposal of the method for the official control. *Acta Parasitologica*, **59**(4), 629–634.

Galaktionov, K, V.K, V., Irwin, S, W,S., B.W, B., and Saville, D, HD, H, 2006. One of the most complex life cycles among trematodes: a description of *Parvatrema margaritense* (Ching, 1982) n. comb. (Gymnophallidae) possessing parthenogenetic metacercariae. Parasitology **132**, 733–746

Gebreegziabher, H., Degefu, H. and Tsegay, A.K., 2020. Prevalence of internal helminth parasites of fish in Gilgel-Gibe River and three selected ponds in and around Jimma Town, Southwest Ethiopia. *Turkish Journal of Fisheries and Aquatic Sciences*, **20**(9), pp.693-699.

Getahun, A., 2002. The Nile Basin: Riverine Fish and Fisheries. Dept. of Biology Addis Ababa University, Ethiopia, pp: 19.

Grossart, H, P., Van den, S., Wyngaert, M. Kagami, C. Wurzbacher, M. Cunliffe, K., 2019. Rojas-Jimenez, Nat. Rev. Microbiol., 17, pp. 339-354, 10.1038/s41579-019-0175-8

Gulelat, Y., Yimer, E., Asmare, K. and Bekele, J., 2013. Study on parasitic helminths infecting three fish species from Koka reservoir, Ethiopia. *SINET: Ethiopian Journal of Science*, **36**(2), pp.73-80.

Hailu, M., Mitiku, M, A., 2021. The Effects of Fish Parasites in Freshwater Culture and Capture Fisheries and their Treatment Mechanisms, International Journal of Zoology and Animal Biology (IZAB),4(2), 1-10, https://doi.org/10.23880/izab-16000289.

Hasegawa, R, Otsuki Y, Uemura, Y., Furusawa, C., Naka, M., Kanno, Y., Koizumi, I., 2025. Positive feedback between parasite infection and poor host body condition reduces host survival in the wild, https://doi.org/10.1111/1365-2435.14742

Hoffman GL. 2019. Parasites of North American freshwater fishes. Cornell University Press.

Hundie, K., Berisa, L., Shitaw, T., Asmamaw, B. and Fekansa, T., 2021.Evaluating Household's Willingness to Pay for Catfish Species Conservation in Lake Hawassa, Sidama National Region State, Ethiopia. *International Journal of Oceanography and Aquaculture*, **5**(2):000204.

Imam, T.S., and Dewu, R.A., 2010. Survey of piscine ecto-and intestinal parasites of clarias species sold at Galadima Road Fish Market, Kano metropolis, Nigeria. *Bioscience Research Communications*, **22**(4), pp.209-214.

Janda, J, M., Abbott, S, L.,2010. The genus Aeromonas: taxonomy, pathogenicity, and infection. Clin Microbiol Rev, 23(1):35-73. doi: 10.1128/CMR.00039-09.

Kaba, T, Shurbe, M, Wondimu, A. 2024. Awareness of fish-borne zoonoses and prevalence of Contracaecum in Oreochromis niloticus and Lates niloticus collected from Lake Chamo, Arba Minch, Ethiopia. Zoonoses Public Health, 71(7):790-798. doi: 10.1111/zph.13145

Kaur, K., Reddy, S., Barathe, P., Oak, U., Shriram, V., Kharat, S, S., Govarthanan, M., Kumar, V., 2022. Microplastic-associated pathogens and antimicrobial resistance in environment, Chemosphere, 291, 10.1016/j.chemosphere.2021.133005

Kaygorodova, I, A., Sorokovikova, N, V. 2014. Mass leech infestation of sculpin fish in Lake Baikal, with clarification of disease-prone species and parasite taxonomy. Parasitol Int, ;63(6):754-7. https://doi.org/doi: 10.1016/j.parint.2014.06.009.

Kelsey, D, S., 1996. Enteric Nematodes of Lower Animals Transmitted to Humans: Zoonoses in Medical Microbiology, 4th edition.

Keyombe, J,L., Waithaka, E., Obegi, B., 2015. Length–weight relationship and condition factor of Clarias gariepinus in Lake Naivasha, Kenya, International Journal of Fisheries and Aquatic Studies; 2(6): 382-385,

Khan, S., Ahmed, S., Serajuddin, M., Saifullah, M, K., 2018. Variation in seasonal prevalence and intensity of progenetic metacercariae of Clinostomum complanatum infection in Trichogaster fasciatus fish, Beni-Suef University Journal of Basic and Applied Sciences, 7(3),310-316, https://doi.org/10.1016/j.bjbas.2018.03.006.

Khidr, A, A., Said, A, E., Abu Samak, O, A., Abu Sheref,S,E., 2012. The impacts of ecological factors on prevalence, mean intensity and seasonal changes of the monogenean gill parasite, Microcotyloides sp., infesting the Terapon puta fish inhabiting coastal region of Mediterranean Sea at Damietta region, The Journal of Basic & Applied Zoology, 65(2),109-115, https://doi.org/10.1016/j.jobaz.2012.06.003.

Kiprono, S.W., 2017. Fish parasites and fisheries productivity in relation to extreme flooding of Lake Baringo, Kenya. *Published master's Thesis. Kenyatta University*.

Klimpel, S., Thomas, K., Julian, M., Dorian, D. D., Regina, K., Judith, K.,2019. *Parasites of marine fish and cephalopods - A practical guide* (1st ed.). Springer.

Klinger, R.E. and Floyd, R.F., 1998. *Introduction to freshwater fish parasites*. Gainesville: University of Florida Cooperative Extension Service, Institute of Food and Agriculture Sciences, EDIS.

Kubitza, F., 2016. Common salt a useful tool in aquaculture, part 1, Simple ingredient offers multiple benefits to freshwater fish farmers, https://www.globalseafood.org/, accessed April 2025.

Labaude S, Rigaud T, Cézilly F, 2015. Host manipulation in the face of environmental changes: Ecological consequences, International Journal for Parasitology: *Parasites and Wildlife*, **4**(3), PP. 442-451, https://doi.org/10.1016/j.ijppaw.2015.08.001

LEDP, 2006. Lake Fishery development project, Proceeding of the national fishery seminar. Ziway number 1995. Lake Fishery development project working paper number 21AA ministry of agriculture.

Leung, T, L, F.,2022. Economies of parasite body size. Curr Biol. 32(12): R645-R649. https://doi.org/doi: 10.1016/j.cub.2022.01.059.

LFDP, 1996. Fisheries statistical bulletin No.3. Lake Fisheries Development Project Working Paper 20. Ministry of Agriculture, Addis Ababa, Ethiopia

Lima, D, P., Giacomini, H, C., Takemoto, R, M., Agostinho, A, A., Bini, L, M.,2012. Patterns of interactions of a large fish-parasite network in a tropical floodplain. J Anim Ecol.,81(4):905-13. https://doi.org/doi: 10.1111/j.1365-2656.2012.01967. x.

Ljubojevic, D., Novakov, N., Djordjevic, V., Radosavljevic, V., Pelic, M., & Cirkovic, M. (2015a). Potential parasitic hazards for humans in fish meat. *Procedia Food Science*, **5**, 172–175.

Ljubojevic, D., Radosavljevic, V., Puvaca, N., Balos, M. Z., Đorđevic, V., Jovanovic, R., & Cirkovic, M. (2015b). Interactive effects of dietary protein level and oil source on proximate composition and fatty acid composition in common carp (*Cyprinus carpio* L.). *Journal of Food Composition and Analysis*, **37**, 44–50.

Marcogliese, D. J. 2005. Parasites of the superorganism: Are they indicators of ecosystem health? International Journal of Parasitology 35:705-716.

Mattiucci, S., Paoletti, M., Colantoni, A., Carbone, A., Gaeta, R., Proietti, A., Frattaroli, S., Fazii, P., Bruschi, F., Nascetti, G.2017. Invasive anisakiasis by the parasite Anisakis pegreffii (Nematoda: Anisakidae): diagnosis by real-time PCR hydrolysis probe system and immunoblotting assay. BMC Infect Dis,17(1):530. doi: 10.1186/s12879-017-2633-0.

Melak, T., and Getahun, A., 2012. Diversity and relative abundance of fishes in some temporary and perennial water bodies of the Baro basin, Gambella, Ethiopia, Ethiop. J. Biol. Sci. 11(2):193-206,

Menberu, Z., Mogesse, B., Reddythota, D.,2021. Evaluation of water quality and eutrophication status of Hawassa Lake based on different water quality indices, Applied Water Science, 11:61 https://doi.org/10.1007/s13201-021-01385-6

Mengesha T, A., 2015. Fish Species Diversity in Major River Basins of Ethiopia: A Review, World Journal of Fish and Marine Sciences 7 (5): 365-374, 2015, 10.5829/idosi.wjfms.2015.7.5.95195

Mgbemena, A., Arimoro, F., Omalu, I, and Keke, U., 2020. Prevalence of helminth parasites of Clarias gariepinus and Tilapia zillii in relation to age and sex in an afrotropical stream, Egyptian Journal of Aquatic Biology & Fisheries, https://www.ejabf.journals.ekb.eg/

Mitiku, M.A., 2017. Parasite Species Richness of Fish from Fishponds and Fingerling Sources in Central Ethiopia: It's Implication on Aquaculture Development (Doctoral dissertation, Doctoral dissertation, University of Natural Resources and Life Sciences).

Mitiku, M.A., Teklu, A. and Tadesse, Z., 2018. Prevalence of parasite of Nile Tilapia *(Oreochromis niloticus)* and African Big Barb *(Labeobarbus intermidus)* fish in Gigel-Gibe-I Dam, Jimma Zone, Ethiopia, American-Eurasian Journal of Scientific Research **13**(2): 18-24, 2018.

Mocho, J, P., Blasco, J, R., Lundegaard, P, R., McKimm, R., Jenčič, V., Krogh, K, V.,2025. Methods of humane killing of laboratory fish: FELASA Working Group recommendations. Lab Anim, 59(5):599-613. doi: 10.1177/00236772251351095.

Mukama, S., 2008. Variations in abundance and diversity of parasites infecting catfish, *Clarias gariepinus (Burchell 1822) and tilapia Oreochromis urolepis (Norman 1922) in the Mindu dam: Morogoro municipality, Tanzania* (Doctoral dissertation, Unesco-IHE).

Nikolaev KE, Sukhotin AA and Galaktionov KV (2006). Infection patterns in White Sea blue mussels (*Mytilus edulis* L.) of different age and size with metacercariae of *Himasthla elongata* (Echinostomatidae) and *Cercaria parvicaudata* (Renicolidae). Diseases of Aquatic Organisms **71**, 51–58

NOAA Fisheries, 2020. Aquaculture Supports a Sustainable Earth, https://www.fisheries.noaa.gov/, accessed April 2025.

Obiero, K., Meulenbroek, P., Drexler, S., Dagne, A., Akoll, P., Odong,R., Kaunda-Arara, B., Waidbacher, H.,2019. The Contribution of Fish to Food and Nutrition Security in Eastern Africa: Emerging Trends and Future Outlooks, https://doi.org/10.3390/su11061636

Ogello EO, Mlingi FT, Nyonje BM *et al*. Can integrated livestock-fish culture be a solution to East Afircan's food insecurity? A Review.*African J Food Agric Nutr Dev*.*13 (4),* 8058-8078 (2013).

Okon, E,M., Oyesiji, A,A., Okeleye, E,D., Kanonuhwa, M., Khalifa, N,E., Eissa, E,H., Mathew, R,T., Eissa, M,E,H., Alqahtani, M,A., S, A.lnour, S,A., 2024.The Escalating threat of climate change-driven diseases in fish: Evidence from a global perspective - A literature review. Environ Res, 263(Pt 3):120184. doi: 10.1016/j.envres.2024.120184.

Outa, J.O., Dos Santos, Q.M., Avenant-Oldewage, A. *et al.* 2021. Parasite diversity of introduced fish *Lates niloticus*, *Oreochromis niloticus* and endemic *Haplochromis* spp. of Lake Victoria, Kenya. *Parasitol Res* **120**, 1583–1592. https://doi.org/10.1007/s00436-021-07095-x

Ozer, A., Oztruk, T., Oztruk, M, O.,2002. Prevalence and intensity of *Gyrodactylus arcuatus* Bychowsky, 1933 (Monogenea) infestations on the three-spined stickleback, *Gasterosteus aculeatus* L., 1758, Turk. J. Vet. Anim. Sci, pp. 807-812

Oztruk, M, O., Alutnel, F, N.,2006. Occurrence of Dactylogyrus infection linked to seasonal changes and host fish size on four cyprinid fishes, Acta Academiae, 52 (4), pp. 407-415

Paperna, I., 1996. Parasite infection and diseases of fish in Africa: An update. Technical Paper **31**. Rome, Central Institute of Freshwater Aquaculture. *Food and Agriculture Organization, United Nations*.

Paulose, T, Nkosi, Z, Z, Endriyas, M. 2022. Prevalence of hypertension and its associated factors in Hawassa city administration, Southern Ethiopia: Community based cross-sectional study. PLoS One. 1;17(3): e0264679. doi: 10.1371/journal.pone.0264679.

Petty, B, D., Francis-Floyd, R., YanongR., Yanong, R., P,2022. Parasitic Diseases of Fish, https://www.msdvetmanual.com/, accessed, April, April 2025

Pouder, D.B., Curtis, E.W. and Yanong, R.P.E., 2005. Common freshwater parasite pictorial guide edis. *Online). http://edis. ifas. ufl. edu*.

Poulin R, 2014. Parasite biodiversity revisited: frontiers and constraints, International Journal for Parasitology, 44(9),2014, Pages 581-589, ISSN 0020-7519, https://doi.org/10.1016/j.ijpara.2014.02.003

Poulin R, Presswell B. 2022. Is parasite taxonomy really in trouble? A quantitative analysis, International Journal for Parasitology, 52, 7, 2022, 469-474, https://doi.org/10.1016/j.ijpara.2022.03.001

Poulin, R., 2005. Variation in the intraspecific relationship between fish length and intensity of parasitic infection: biological and statistical causes, https://doi.org/10.1111/j.1095-8649.2000.tb02090.x

Power, C., Shirakashi, S., Bott, N.J., Nowak, B.F. 2025. Parasites in Aquaculture. In: Smit, N.J., Sures, B. (eds) Aquatic Parasitology: Ecological and Environmental Concepts and Implications of Marine and Freshwater Parasites. Springer, Cham. https://doi.org/10.1007/978-3-031-83903-0_23

Pradipta R. Rauta, Bismita Nayak, Surajit Das, 2012. Immune system and immune responses in fish and their role in comparative immunity study: A model for higher organisms, Immunology Letters,Volume 148, Issue 1,Pages 23-33, ISSN 0165-2478, https://doi.org/10.1016/j.imlet.2012.08.003

Ramos P, Parasites in fishery products - Laboratorial and educational strategies to control, Experimental Parasitology, 211,2020, 107865, ISSN 0014-4894, https://doi.org/10.1016/j.exppara.2020.107865.

Reed, C, C., Basson, L., Van As, L, L.,2002. Myxobolus species (Myxozoa), parasites of fishes in the Okavango River and Delta, Botswana, including descriptions of two new species. Folia Parasitol (Praha);49(2):81-8. doi: 10.14411/fp.2002.016.

Renner, E.D., Duggan, I.C., 2024. Season, size, and sex: factors influencing monogenean prevalence and intensity on *Gambusia affinis* in New Zealand. *Parasitol Res* **123**, 228. https://doi.org/10.1007/s00436-024-08241-x

Reshid, M., Adugna, M., Redda, Y.T., Awol, N. and Teklu, A., 2015. A study of Clinostomum (trematode) and Contracaecum (nematode) parasites affecting Oreochromis niloticus in Small Abaya Lake, Silite Zone, Ethiopia. *J. Aquac. Res. Dev*, **6**, p.316.

Roberts, R.J., 2001. *Fish Pathology*.3rd edn. Technical Director, Landa catches Ltd. Scotland, pp. 270–300.

Roy, P.E. and Young, E., 2002. Nematode (round worm) infection in fish. *Florida, IFAS*, **91**, pp.1-10.

Salem, M.A., Mahdy, O.A., El-Saied, M.A., Kamel, M, S., Mohammed, F, F., Ramadan, *R, M.*, 2025. Molecular and pathological insights into gene expression and oxidative stress in *Clinostomum complanatum* and *Euclinostomum heterostomum*. *Sci Rep* **15**, 37586 (2025). https://doi.org/10.1038/s41598-025-16469-5

Senapin, S., Phiwsaiya, K., Laosinchai, P., Kowasupat, C., Ruenwongsa, P., Panijpan, B., 2014. Phylogenetic analysis of parasitic trematodes of the genus Euclinostomum found in Trichopsis and Betta fish. J Parasitol.;100(3):368-71. https://doi.org/doi: 10.1645/13-285.1.

Shafiq A., Abbas F., Hafeez-Ur-Rehman M., Khan B.N., Aihetasham A., Amin I., Hmidullah, Mothana R.A., Alharbi M.S., Khan I., et al. Parasite Diversity in a Freshwater Ecosystem. Microorganisms. 2023; 11:1940.

Shamsi S, Turner A, Wassens S. 2018. Description and genetic characterization of a new Contracaecum larval type (Nematoda: Anisakidae) from Australia. J Helminthol. 2018 Mar;92(2):216-222. doi: https://doi.org/10.1017/S0022149X17000360.

Shamsi, S., Barton, D.P. 2023. A critical review of anisakidosis cases occurring globally. *Parasitol Res* **122**, 1733–1745. https://doi.org/10.1007/s00436-023-07881-9

Simková, A., Jarkovský, J., Koubková, B., Barus, V, Prokes, M.,2005. Associations between fish reproductive cycle and the dynamics of metazoan parasite infection. Parasitol Res, 95(1):65-72. https://doi.org/doi: 10.1007/s00436-004-1261-y.

Skelton, P.H., Tweddle, D., Jackson, P.B.N. 1991. Cyprinids of Africa. In: Winfield, I.J., Nelson, J.S. (eds) Cyprinid Fishes. Fish & Fisheries Series, vol 3. Springer, Dordrecht, https://doi.org/10.1007/978-94-011-3092-9_7

Steckler N., Yanong, Yanong R, PR., 2024. Argulus (Fish Louse) Infections in Fish, https://edis.ifas.ufl.edu/

Sukhdeo, M.V. 2012. Where are the parasites in food webs? *Parasites Vectors* **5**, 239, https://doi.org/10.1186/1756-3305-5-239

Sures, B., Nachev, M.,2022. Effects of multiple stressors in fish: how parasites and contaminants interact. Parasitology;149(14):1822-1828. doi: 10.1017/S0031182022001172.

Tadesse, B., 2009. Prevalence and abundance of fish parasites in Bomosa Cage Systems and lakes Babogaya and Awassa, Ethiopia.

Tesfay, S., Teferi, M., Dejenie, T., Abay T., Hiluf, G., 2024. A survey of ecto and endoparasites of Nile tilapia, Oreochromis niloticus (Linnaeus, 1758) fingerlings in Midmar reservoir, Adwa, northern Ethiopia, Journal of Applied Animal Research, 52:1, 2310158, https://doi.org/DOI: 10.1080/09712119.2024.2310158

Tesfaye, A., Teklu, A., Bekelle, T., Tkue, T., Kebede, E., Gebretsadik, T. and Berhe, N., 2017.A survey on occurrence of internal and external fish parasites and causes of fish population reduction in Lake Hashenge, Tigray, Ethiopia. *Ethiopian Veterinary Journal*, **21**(2), pp.75-91.

Tesfaye, Z., Ferede, B., Pavanasam, N. and Workagegn, K.B., 2020. Prevalence of nematode parasite, Contracaecum, in Nile tilapia, African catfish and Barbus species in Lake Hawassa, Ethiopia.Aquaculture Research, **51**(10), pp.3993-3998.

Tesgera G, and Bayou K, 2021. Assessment of Current Practices, Major Challenges and Opportunities of the Fishery of Lake Hawassa, Southern Ethiopia, DOI: http://dx.doi.org/10.22192/ijarbs.2021.08.02.004, http://www.ijarbs.com/, 8(2), 1-9

Thakkar AN, 2023, Seasonal Variation in Prevalence of Nematode Parasites In Coilia Dussumieri from Uran, West Coast of India, Journal of Survey in Fisheries Sciences, 10(1) 4007-4009

Thrusfield, M.V., 2005. Veterinary Epidemiology 2nd ed. Black Well Science LTD, UK. pp. 233-250.

Tibebe, D., Tesfaye, Y. Kassa, Y., 2023.The impact of sewage effluents on water quality of Lake Hawassa, Ethiopia. *BMC Chemistry* **17**, 42. https://doi.org/10.1186/s13065-023-00954-x.

Tilahun, G., and Bihonegn, T., 2017.Study on Helminth Parasites in *Tilapia nilotica* from Lake Zeway, Ethiopia, Int. J. Adv. Res. Biol. Sci, **4**(10): 21-25.

Truter, M., Hadfield, K, A., Smit, N, J., 2023. Review of the metazoan parasites of the economically and ecologically important African sharptooth catfish Clarias gariepinus in Africa: Current status and novel records. Adv Parasitol.;119:65-222. doi: https://doi.org/10.1016/bs.apar.2022.11.001.

Trutera, M., Hadfielda K, A., and Smit N, J., 2022. Review of the metazoan parasites of the economically and ecologically important African sharptooth catfish Clarias gariepinus in Africa: Current status and novel records, 1-158, https://doi.org/10.1016/bs.apar.2022.11.001

Turner, W, C., Kamath, P, L., van Heerden, H., Huang, Y, H., Barandongo, Z, R., Bruce, S, A., Kausrud, K.,2021 The roles of environmental variation and parasite survival in virulence-transmission relationships. R Soc Open Sci. 2021 Jun 2;8(6):210088. doi: 10.1098/rsos.210088.

Valtonen, E.T., Marcogliese, D.J. and Julkunen, M., 2010. Vertebrate diets derived from trophically transmitted fish parasites in the Bothnian Bay. *Oecologia*, **162**(1), pp.139-152.

Vandi, P., Daniel, J. L.2023. Prevalence of Gastrointestinal Parasites and Condition Factor of Oreochromis niloticus (L.) in Kiri Reservoir, Shelleng, Adamawa State, Nigeria, Biological and Environmental Sciences Journal for the Tropics 20(3), https://dx.doi.org/10.4314/bestj.v20i3.7, 182-188.

Vidal-Martínez, V, M., Aguirre-Macedo, M, L., McLaughlin, J, P., Hechinger, R, F., Jaramillo, A, G., Shaw, J, C., James, A, K., Kuris, A, M., Lafferty, K, D.,2012. Digenean metacercariae of fishes from the lagoon flats of Palmyra Atoll, Eastern Indo-Pacific. J Helminthol, 86(4):493-509. doi: https://doi.org/10.1017/S0022149X11000526

Wakjira,M., Tolemariam,T., Kim, D, J., Kim, K, R., 2013. Aquaculture development in Ethiopia: review on potential and strategy, journal of agriculture, life and environmental sciences, 25(3), 20-24

WHO, 2020. Zoonoses, https://www.who.int/, assessed April 18, 2025.

Wubie, A., Teshome, K., Tesfaye, G., 2023. Potential fish yield and biology of some fish in Lake Hertale, Afar regional state, Ethiopia. Heliyon, 9(8):e18661. doi: 10.1016/j.heliyon. 2023.e18661.

Zekarias, T. and Yimer, E., 2007. Study on parasites of fish at Lake Awassa, Ethiopia. *Bulletin of Animal Health and Production in Africa*, **55**(3), pp.149-155.

Zhokhov, A.E., Mironovsky, A.N. and Miretskaya, D.A., 2007. Methods of the complete Parasitological dissection of fish. *Freshwater biology group, JERBE, Moscow–Addis-Ababa*, pp.1-12.

Ziarati, M., Zorriehzahra, M, J., Hassantabar, F., Mehrabi, Z., Dhawan, M., Sharun, K., Emran, T, B., Dhama, K., Chaicumpa, W., Shamsi, S.,2022. Zoonotic diseases of fish and their prevention and control. Vet Q.;42(1):95-118. https://doi.org/ doi: 10.1080/01652176.2022.2080298.

Raw data

Click here to access/download
Supporting Information
Table s1.xlsx

Prevalence of fish parasites from Lake Hawassa, Ethiopia

Debela Abdeta[1,4]*, Megersa Akasa[2], Yobsan Tamiru[3],
[1]College of Veterinary Medicine and Agriculture, Addis Ababa University, Bishoftu, Ethiopia
[2]College natural resource and agriculture, Ambo University, Gudar, Ethiopia
[3]School of Veterinary Medicine, Wollega University, Nekemte, Ethiopia
[4]Department of Veterinary Bioscience, Melbourne Veterinary School, Melbourne University, Melbourne, Australia
*Correspondence: Debela Abdeta
Tel. +251 913125925
Email: debela.abdeta@aau.edu.et, or debelaabdeta.efa@student.unimelb.edu.au

ABSTRACT

Background: Fish provides quality protein, various vitamins and minerals essential for human consumption~~Fish provides quality protein, variety of vitamins, and minerals to human being~~. According to FAO, 2018 m~~M~~ore than 17% of animal protein for global population is obtained from fish. An estimated 200 species of fishes of marine and freshwater fish are known to occur in Ethiopia. Fish parasites are the main cause of the significant threat to the fish biodiversity of Ethiopia's freshwater islands. Lake Hawassa is the economic heartbeat of the city which supports the artisanal fishing sector. The ~~Fish~~ parasite of the fishes at lake Hawassa are hitherto poorly known and there are limited reports on the type of ~~pastes~~ parasites across ~~the~~ different fish hosts.

Methods: a cross-sectional study was carried out in 2023- 2024 to estimate the prevalence of fish parasites and host factors. In the current survey *Oreochromis niloticus* (n=135), *Clarias gariepinus* (n = 87), and *Barbus intermedius* (n = 43) were sampled. ~~in the current survey *Oreochromis niloticus(n=135), Clarias gariepinus* (n = 87), and *Barbus intermedius* (n = 43) were included.~~ Internal and external organs was inspected for the presence of the parasite infection. Some external parasites were extracted from the fresh water immediately after fish were caught. The randomly selected host was classified based on species, sex, body weight, and the total body length. Parasites are identified morphologically at genera level using standard identification keys and pictorial guide. Analysed data were presented as frequencies, percentages, mean intensity, and mean abundance. Chi-square and P-values were considered for statistical association.

Results: The parasite distribution varies with sex, body weight, and standard length of the studied fish. The overall infection/infestation rate is 88.30% (n = 234). A total of 174~~848~~ parasites classified under Nematodes (e.g., Contracaecum), Digenean trematodes (e.g., Clinostomum, Euclinostomum, Diplostomum, encysted metacercariae), Monogeneans (Dactylogyrus), Myxozoans (Myxobolus spp.), Crustacean ectoparasites (Argulus, Dolops), Hirudinea (leeches) ~~under trematodes, nematodes, protozoa, crustaceans and *Hirudinea*~~ was collected from *O.*~~O.~~

Formatted: Line spacing: Multiple 1.15 li
Formatted: Not Highlight
Formatted: Font: 12 pt
Formatted: Not Highlight
Formatted: Not Highlight
Formatted: Not Highlight
Formatted: Not Highlight
Formatted: Not Highlight
Formatted: Not Highlight
Formatted: Font: Not Italic
Formatted: Not Highlight
Formatted: Font color: Custom Color(RGB(238,0,0))
Formatted: Space Before: Auto, After: Auto, Line spacing: Multiple 1.15 li, Pattern: Clear (White)
Formatted: Font: 12 pt

niloticus (788~~7~~), *C.*~~*C.*~~ *gariepinus* (642) and *B.* ~~*B.*~~ *intermedius* (318). The internal parasites recovered from *O. niloticus* were *Clinostomum* (65.16%), *Euclinostomum* (34.07%), *Contracaecum* (26.67%), ~~*Dactylogyrus* species (28.15%),~~ and *Diplostomum* (24.44%). Significant infection level with *Myxobolus* species, leeches, *Argulus*, and *Dolops* species was also recorded. *Digenean encysted metacercaria* (68.97%) and *Contracaecum species* (50.57%) are the most common parasite species infecting *C. gariepinus*. *Diplostomum species*, *Dactylogyrus species*, leeches, *Argulus species*, and *Dolops species* pose a serious threat to fish health in lake Hawassa. The mean intensity of the parasites in *B. intermedius*, C. *gariepinus* and *O*. *niloticus* ~~is found~~was to be 8.59, 7.92 and 6.79 while mean abundance is 7.40, 7.37 and 5.84 respectively.

Conclusion: This study found that various body parts are contaminated with one or more parasites. Some of those parasites have established zoonosis or cause cross infection to fishes requiring urgent cooperation in performing pre-stock assessment for optimal production and maintaining the heath of fishery and hence human being.

Keywords: *Fish, Lake Hawassa, Parasites, Prevalence*

1. INTRODUCTION

The fish farming is one of the developing sectors in low- and medium-income countries offering significant potential for food security, growth of the economy and improvement of the livelihood (Ogello et al., 2013; Boyd et al., 2022). The demand for fish meat is steadily rising due to its high nutritional value, particularly essential fatty acids, which are ideal for human consumption (Ljubojevic et al., 2015a, 2015b, Brye, 2023). Fish resources are vital to support human well-being and economic development (Boyd et al., 2022). Aquaculture provides year-round jobs, supports resilient working water fronts and coastal communities and can provide sustainable economic growth. They also drive employment in other areas of industry such as seafood processing, feed and equipment manufacturing in different countries including Ethiopia (NOAA Fisheries, 2020).

Ethiopia is a landlocked nation that depends on its inland waterways for fish production. ~~Some of the lakes~~Several lakes and rivers in the country are used for fish production to feed its population while maintaining the nation's food security (Desalegn and Shitaw, 2021). The Ethiopian government has suggested aquaculture to increase fish output and as a substitute for food security and poverty alleviation (Deng, 2020). The data from the Ministry of Agriculture and Rural Development of Ethiopia, published in 2011, indicate that the total annual fish catch in Ethiopia rose from 14,000 tons in 1998 to 24,257 tons in 2011. Since then, there has been a plan to increase annual fish production by 65,000 tons per year. To achieve the plan the country constructed big dams such as Tendaho reservoir, Tekeze dam, Galgal Gibe I, II and III, and Renaissance dam in addition to the previously naturally used lakes (Gebremedhn and Tsegay, 2017). Today, aquaculture is seen as a crucial component of plans and strategies for rural and agricultural

Formatted: Not Highlight

Formatted: Font: 12 pt

Formatted: Not Highlight

Formatted: Font color: Custom Color(RGB(34,34,34))

Formatted: Line spacing: Multiple 1.15 li

Formatted: Not Highlight

development in the country. The Ethiopian renaissance dam is emerging with a major fishery hub producing over 14,000kg of fishes daily (Food Business, 2025).

Lake Hawassa is one of the major lakes in the Ethiopian Rift Valley, situated close to Hawassa town and features an endorheic basin system. The water from the lake is essential to the nearby towns for recreational use, residential water use, farming, and fish harvesting (Memberu et al., 2021). This lake is one of the most fished water bodies in Ethiopia where *O. niloticus* accounts for -90% of the annual catch (Tesgera and Bayou, 2021).

Formatted: Not Highlight

The freshwater fish fauna of Ethiopia is a mixture of Nilo Sudanic, East African and endemic forms (Skelton et al., 1991). The Nilo Sudanic forms those species represented under *Alestes, Labeo, Bagrus, Citharinus, Hydrocynus, Hyperopisus* and *Protopterus* are some representatives from Ghibe-Omo, Baro-Akabo, and Abay Basins (Melak and Getahun, 2012). The highland east African forms are found in the northern rift valley lakes (Hawassa, Ziway, Langano, and the high land lakes (Tana and Haiq). *Barbus, Clarias, Garra, Oreochromis* and *Varicosrinus* are the common genera of fish common to this area. They are related to fishes of eastern and southern African and Arabian Peninsula (Skelton et al., 1991). The fishes of high mountain torrential streams largely belong to *Cyprinidae* adapted to flowering flood waters that occur seasonally. Two genera of fishes (Barbus and Garra) are dominant in this stream (Getahun, 2002; Mengesha, 2015).

In Ethiopia, eating fish is a unique experience. As usual, it is consumed by hand with injera or bread and a hot sauce to dip into. The grilled fins are very delicious since, bear a few bones. Fish tibs are chunks of fish marinated in lime juice and chilli spices, followed by the addition of shredded ginger and garlic and sesame oil, olive oil, and paprika (CNN travel, 2023). Those practices could expose the community for public hazard as some of those parasites have zoonotic risk.

Fish production is challenged by variety problems from which fish disease is the dominant. In Ethiopia, fish disease particularly those caused by parasites and bacteria are concern with studies indicating high prevalence of parasites in commercially important fish species in lakes like Ziway, Hawassa, and Tana (Gebremedhn and Tsegay, 2017). Parasitic species can be found everywhere and on every living organism. The rate of parasitic infection is high among vertebrates residing in aquatic environment (Chai et al., 2005). Their presence in their host is generally at equilibrium in aquatic organisms and the most common lifestyle on the planet (Marcogliese, 2005, Tesfay et al, 2023, Chew et al, 2024).

More than one-third of all species on Earth are parasites, and they contribute significantly to ecosystem biomass. Due to this host-parasite interaction is one of the most prevalent biotic relationships in nature (Hasegawa et al., 2025). Parasites affect fish health through mechanical, physical and reproductive damage. These changes can reduce growth, fecundity and survival, change behavioural and sexual characteristics, and result in many other maladaptive alterations of the infected host (Hailu and Mitiku, 2021). Parasites are one of the main threats facing the fisheries

industry (Power et al., 2025). This could be from economic losses due to fish rejection at markets, mortality in aquaculture, impact on fish health, or zoonotic concerns (Chai et al., 2005, Ziarati et al, 2022). In terms of host biology, population dynamics, and ecosystem health, parasites play a significant role. Any fish species, as well as any aquatic culture system, can contain them (Gulelat *et al*., 2013). The most common parasitic agents of fish are trematodes (fluke, e.g., *Opisthorchis spp*.), nematodes (round worm, e.g., *Anisakis spp*.), and cestodes (tapeworm, e.g., *Diphyllobothrium spp*.). Furthermore, protozoan organisms like *Cryptosporidium* species are regarded as zoonotic infections originating from fish (Ziarati et al, 2022). If left unmanaged, these illnesses can cause massive fish deaths, limit fish productivity, and in some situations, can spread infections to humans and other animals who consume diseased or infected fish (Kiprono, 2017). In addition, arthropods and crustaceans' species are known to infect fish (Janda and Abbott, 2010). Different species of leeches are also known to infect fishes (Kaygorodova and Sorokovikova, 2014, Tesfay et al, 2023).

Studies have indicated that biotic factors such as sex, body length and height influence the burden of parasitism. Accordingly, female had significantly higher prevalence and mean intensity than males, longer fishes have higher prevalence and mean intensity. Additionally, females may have compromised immune system specially during reproductive season (Renner and Duggan., 2024).

Fish is a good host for parasite multiplication that can be zoonotic to humans or other animals that feed fishes (Barber and Wright, 2005). The larval stages of parasites could be found in fishes as they are either intermediate or reservoir of those parasites or sexually mature parasites are found for which they definitive hosts. The larvae of anisakid nematodes, trematodes or others can be ingested by humans with under-cooked fish flesh which then develop to morphologically mature but sexually immature worms thus, zoonotic potential (Kaba et al, 2024).

~~Knowledge about parasites of fish has been progressed due to their impact on growth and behaviour of fish and economic losses in recent years.~~ Parasites cause an economic threat to the market value of fish, through consumer attitudes towards the presence of these parasites within food products (Thakkar, 2023). Important determinants of parasite exposure include the distribution of infected hosts, host immune status, and the spatial and temporal distribution of infective stage and intermediate hosts, and this can be influenced by the host's diet or trophic position in a food chain (Valtonen *et al*., 2010). These elements slow down growth and weight gain, impact fish production yield, spread infections among humans and animals, delay in fish sexual maturation, and raise in fish mortality rates. Additionally, by posing marketing issues for commercially harvested species, these cause the nutritional devaluation of fish and resultant economic losses (Gebreegziabher *et al*., 2020). Although these parasites are an important part of biodiversity and can help maintain stability in natural population (Shafiq et al., 2023) they can also lead to extinction often interacting with other stressors such as habitat loss (Brian, 2023). The extinction can be population level or species level.

Formatted: Font: Times New Roman

Formatted: Not Highlight

Moreover, in low and middle income developing countries like Ethiopia, people known little about fish borne zoonotic disease such as trematodiasis and anisakiasis (Kaba et al, 2024). Yet these parasitic infections are commonly reported from different freshwater body fishes (Tasfay et al., 2023) and the cause of significant human infections worldwide (Chai et al., 2005). To date, fewer studies have been conducted on fish parasites in Ethiopia, but they did not quantify the prevalence of parasites across different fish species, body weight, fish lengths and anatomical position in relation to parasite infestation. The current study hypothesised that commercially important fish species from lake Hawassa are infected by internal and external parasites. Hence, the present study aimed at estimating the prevalence of internal and external parasites, calculating the intensity, abundance of *O. niloticus*, *C. gariepinus*, and *B. intermedius* in Lake Hawassa. We have also examined how host factors are related to the parasite prevalence or its burden.

Formatted: Not Highlight

2. MATERIALS AND METHODS

2.1. Description of the Study Area

One of the eight lakes in Ethiopia's Rift Valley is Lake Hawassa. Situated 275 km south of Addis Ababa in the Sidama region, it borders Hawassa city's eastern side. Geographically, the lake is located 1680 meters above sea level between 6°33' and 7°33' N and 38°22' and 38°29' E. The lake has an approximate water volume of 1.3 billion cubic meters (45.9 billion feet^3) and stretches 16 kilometres from northeast to southwest and 8 kilometres from northwest to southeast. The lake's mean depth is 11 meters, and its maximum depth is 21.6 meters (70.9 feet). The lake's surface area, which can reach 99.3 Km^2 during the rainy season, makes up 93.6 Km^2 of the catchment's total area of 1455 Km^2 (Figure 1). According to estimates, Lake Hawassa releases 58 ×106 m^3 of groundwater annually into nearby basins (Hundie et al., 2021). The location of the lake and the city is found at https://en-us.topographic-map.com/map-lllbf3/Lake-Hawassa/

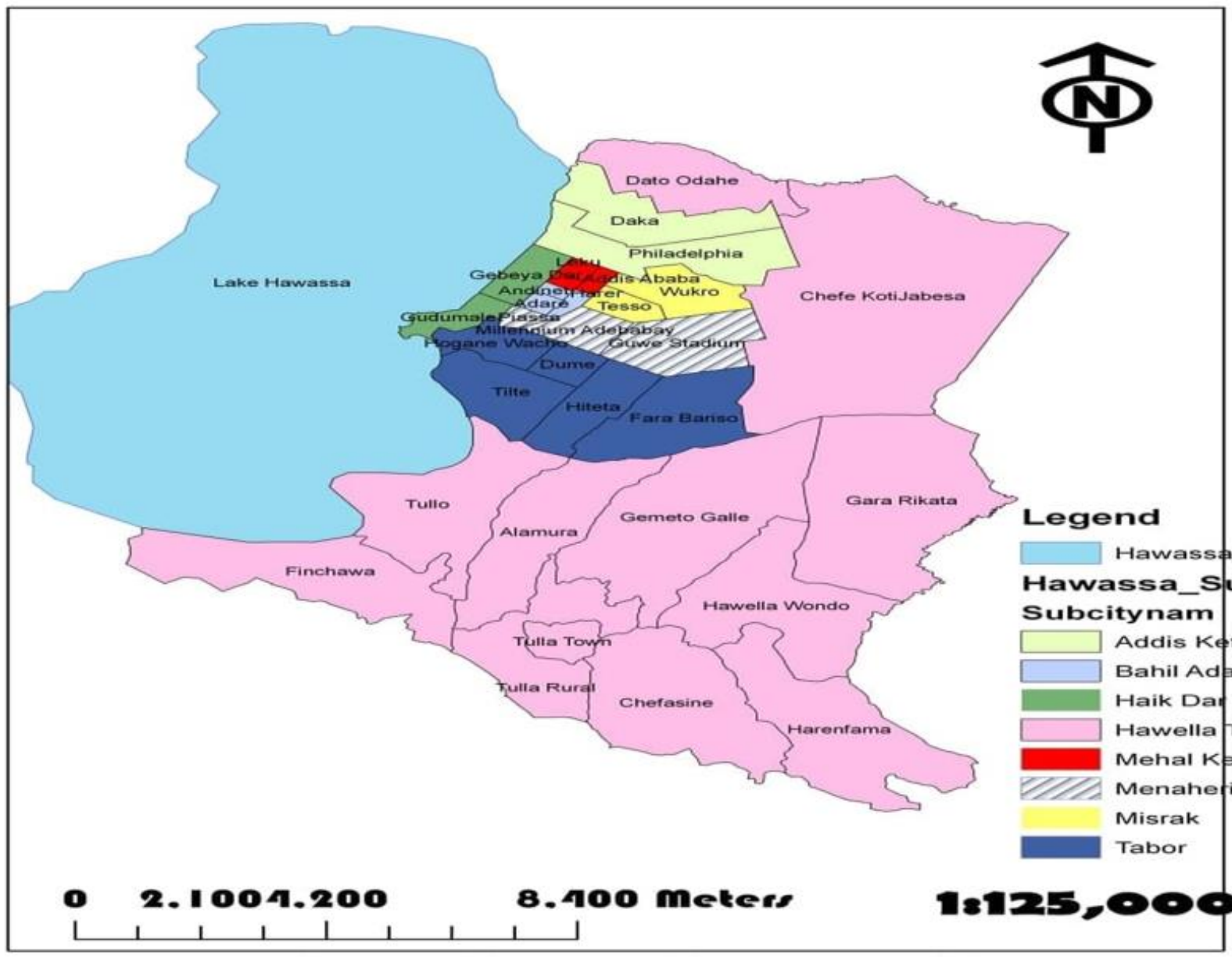

Figure 1: Map of Hawassa city, taken from Paulos et al, 2022

2.2. Study Design and sampling technique

A cross-sectional study was conducted from October 2023 to July 2024 on the fish community at Lake Hawassa to identify the prevalence of major internal and external parasites infesting fishes supported by antemortem and postmortem inspection on fish. The simple random sampling where each fish systematically selected (every specified fish from the cage) random sampling technique was used to select fishes for parasites identification. The assessment was conducted over a period of ten months, from October 2023 to July 2024The assessment was conducted in all for over 10 months.

Formatted: Font: 12 pt, Font color: Green

2.3. Study Population

The Nile tilapia (Oreochromis niloticus), African catfish (*Clarias gariepinus*), and African big barb (*Labeobarbus intermedius*) are the fish species that have been found in Lake Hawassa thus far. The black lampeye (*Aplocheilichthys antinorii*), the stone lapping minnow (G*ara quadrimaculata*), and the straight fin barb (*Barbus paludinosis*) are the other tiny fish present in the lake (Dadebo, 2000). The other three species of minnow fish are not fished because of their small size, but *O. niloticus*, *C. gariepinus*, and *L. intermedius* are the commercially significant species in the nation. About 90% of the whole production is made up of *O. niloticus*, with *C.*

gariepinus and *L. intermedius* contributing roughly 7-8% and 2-3%, respectively (Wubie et al., 2023; LFDP, 1996).

2.4. Sample Size Determination

The sample size was determined according to Thrusfield, (2005) at 95% confidence interval with a 5% marginal error. Accordingly, Zekarias and Yimer (2007) reported prevalence of (79%) of fish parasites from Lake Hawassa. We have applied collective sample size for all the fishes sampled using the formula $n = z^2 \times p\,(1-p)/d^2$, where n= sample size, z = z statistic for a level of confidence (z =1.96 at 95% CI), p = prevalence (p = 0.79), d = precision (if 5%, d= 0.05), the sample size 265 is included in the current study.

2.5. Methods of data collection

2.5.1. Description of the sampled fish

During the sampling, we have randomly picked those fishes from different sampling points. The species, sex, possible anatomical region of the parasite attachment, body weight, and total and standard lengths of individual fish were initially assessed and recorded. The sex of each fish was tentatively assessed externally and confirmed post-dissection by noting the presence of testes or ovaries ~~The sexes of each fish were determined after dissection and noting the presence of testes or ovaries~~ (Imam and Dewu, 2010). Up on arrival at the Hawassa University, the total length was measured by centimetres using measuring tape and the weight measured using digital balance. The size of fish was separately recorded for each fish species and classified into class I, class II, and class II (Tesfaye *et al*., 2020). Classifying fish species based on size and body weight during the parasite studies is crucial because this factor can influence parasite infection patterns, host vulnerability and the overall dynamics of parasite host interaction impacting both fish health and environmental ecosystem (Lima et al., 2012). The selected size breakpoints for length and weight classes reflect biologically meaningful transitions in growth and life-history stages. For example, in *C. gariepinus*, the length thresholds of 50 cm and 75 cm correspond approximately to ontogenetic stages associated with sexual maturation and trophic shifts. Individuals below 50 cm are typically juveniles or subadults with developing gonads and a diet dominated by invertebrates and small prey. Fish between 50–75 cm generally represents sexually mature adults, while individuals exceeding 75 cm are fully mature, larger-bodied predators with broader dietary niches and higher trophic positioning. These ontogenetic changes influence exposure risk to parasites through altered feeding behaviour, habitat use, and immune competence. Therefore, the selected size classes reflect biologically relevant developmental stages that may affect parasite acquisition dynamics within this ecosystem, rather than being purely arbitrary statistical categories (Keyombe et al., 2015).

Formatted: Font: (Default) Times New Roman, Font color: Green

Formatted: Justified, Line spacing: Multiple 1.15 li

Formatted: Font: 12 pt

2.5.2. Antemortem and postmortem identification of parasite

A total of 265 fish were first examined alive (skin and gill scraping and picking with forceps followed by freshly dissected samples). The randomly selected fish were immediately transported in an icebox to the Parasitology unit of Hawassa University, for parasitological examination. Fish were initially examined alive for external abnormalities immediately upon capture. They were then humanely euthanized at the sampling site and placed in an icebox containing crushed ice for transport to the laboratory (Mocho et al., 2025). ~~-~~

Each sampled fish was externally visualized, palpated, and examined for any ectoparasites on the entire fish skin, the fins, and opercular cavity including the gills. The external parasite was harvested to physiological salt solution from fish body and the fresh water (Kubitza, 2016). Scraping from fish skin were taken and smeared on to a clean microscope slide or hand lens. The sample was then examined under compound microscope at 10X, 40X and 100 X magnifications. Then postmortem examination was done using appropriate postmortem kits and standard evisceration technique (Zhokhov *et al*., 2007; Klimpel et al., 2019) and all body cavities was observed for presence of any encysted or free parasite larvae. The gill filaments, stomach, intestine, liver, heart, gall bladder, kidney and gonads were separately taken out and placed on clean Petridishes that contain 10millitre of physiological solution made of (0.9% sodium chloride w/v) (Younis et al., 2017). The head of *C. gariepinus* was dissected longitudinally, fixed in 70% ~~and~~ethanol first, and the cranial cavity was washed away into a petridish using a water dropper and checked for parasites especially *Diplostomum* species. To clear their cuticle, the parasite was placed on in the 99% lactophenol for 24 hours and identified at genus level.~~-~~

The fillet was digested in the pepsin HCl artificial digestion method is used to harvest any encysted larva in the fish muscle. Artificial digestion solution was prepared from 10gram of pepsin powder, 16mL of 25% HCl in 2 litres of tap water prewarmed at 42^0C in water bath. Briefly fish flesh was cut in to 1-2milimeter of small pieces using sharp knife. One-hundred-gram small piece of individual fish flesh was placed in 3liter beaker. The digestion solution was fully submerged the flesh; the beaker was covered with aluminium foil and heated on magnetic stirrer at 40^0C for ~~20~~ 4 hours ~~minutes~~ to fully homogenise the tissue sample. We turned off the magnetic stirrer once observing the dis-appearance of any visible insoluble tissue. The sample was strained by laboratory sieve with approximate pore size of 70-100µm. The content retained on the sieve was examined for any larvae of nematode under stereomicroscope. The collected worms were counted, recoded and preserved in 70% ethanol (Paperna, 1996; Klinger and Floyd, 1998; Roberts, 2001; Roy, 2002; Amlacher, 2005; Pouder *et al*., 2005; Fraulo et al., 2014).

The morphological characteristics of anterior and posterior parts, including boring tooth, the ratio of ventricular appendix to intestinal caecum and the morphology of their tail were referred for

Formatted: Line spacing: Multiple 1.15 li

Formatted: Font: (Default) Times New Roman

Formatted: Pattern: Clear (White)

Formatted: Normal, Space Before: Auto, After: 0 pt, Line spacing: Multiple 1.15 li

characterization. This method was specifically optimized for the recovery of infective larval nematodes (e.g., L3 stages of Anisakidae) from the musculature is relevant here to show why this muscle digestion is necessary (Mattiucci et al,2017). The taxonomic identification of parasites was limited to genus level as the fish harbors mostly larval stages of many nematodes and trematode (Poulin and Presswell, 2022; Hoffman, 2019). The helminthic parasites were identified using parasitological and morphologically approach using standard identification keys and pictorial guide (Pouder et al., 2005).

2.5.3. Prevalence and intensity of the parasite

The prevalence report is calculated considering multiple parasite infestation from single host i.e, one fish could be infected by different parasite groups or free from any parasite. The prevalence and intensity of the parasite was calculated according to the formula (El-Shahawy et al., 2017).

Accordingly,

$$\text{Prevalence}=\frac{\text{Number of individual of a host infected with a particular parasite species}}{\text{Number of hosts examined}}\text{X}100$$

$$\text{Mean intensity}=\frac{\text{Sum of individuals of parasite species in a host sample}}{\text{Total no.of infected individuals of the host in the sample}}$$

$$\text{Mean abundance}=\frac{\text{number of collected parasite}}{\text{Total number examined fishes}}$$

2.6. Data Management and Analysis

All collected raw data was entered into a Microsoft Excel database system and imported to be analysed using SPSS statistical software (version-17). Accordingly, descriptive statistics including frequencies, percentages, mean intensity and abundant intensity were presented in tubular form. The chi-square test (χ^2) was used for a possible explanation of associations between the prevalence of parasites and the selected variables (body weight, length, sex, and fish species) for the sampled fish. P-values less than 0.05 were taken as statistically significant.

3. RESULT

Prevalence of fish parasite in relation to sex, body weight, total and standard length

During the sampling period, 265 fishes belonging to *O. niloticus* (tilapia) (n=135), *B. intermedius (‘nech asa’)* also known as *Labeobarbus intermedius* (n=87), *C. gariepinus* (African catfish) (43) were sampled (Figure 1~~2~~A, 1~~2~~B, 1~~2~~C). The total length of the fish ranges from 10cm to 80cm and the total weight ranges from 100g-2Kg.

Prevalence, mean abundance and mean intensity of parasites in fish

Formatted: Font: 12 pt

Formatted: Font: (Default) Times New Roman

Formatted: Font: 12 pt

Formatted: Font color: Custom Color(RGB(27,28,29))

Formatted: Line spacing: Multiple 1.15 li

Formatted: Not Highlight

Formatted: Not Highlight

Formatted: Font: Not Italic

Of a total of 265 examined fish, 234 (88.30%) were found infested by at least one parasite group (Figure 2~~3~~-8~~9~~). The highest level of parasite infestation was reported in *C. gariepinus* (93.10%) followed by *B. intermedius* (86.05%) and *O. niloticus* (85.93%). The minimum and maximum mean intensity of 5.37 and 8.08 is recorded in *C. gariepinus* and *B. intermedius* respectively. The lower and higher mean abundance of the parasite is also recorded in *C. gariepinus* and *B. intermedius* respectively. The variations in parasite infestations by fish species were not statistically significant ($\chi2 = 2.893$; df = 2, P=~~>~~0.06~~5~~) (Table 1). *C. gariepinus* had the lowest mean intensity (MI = 5.37±2.14), and *B. intermedius* had the highest (MI = 8.08±3.21).

Formatted: Normal, Line spacing: Multiple 1.15 li, No bullets or numbering

Formatted: Not Highlight

Formatted: Font: 12 pt

Prevalence, mean abundance and mean intensity of parasites in fish

The result indicated that 56.3% of male and 43.7% of female in *O. niloticus*, 52.87% male and 47.3% female in *C. gariepinus*, and 55.81% male and 47.13% female in *B. intermedius* were affected by one or more parasites.

Table 1: Prevalence of parasitic infestation with respect to fish species studied

Species of fish studied	No. of fish affected	Number of parasites from each fish	Total parasite collected	Prevalence (%)	Mean intensity	Me abu
O. niloticus (n=135)	116	6-9	788	85.93	6.79	5.8
C. gariepinus (n=87)	81	4-11~~6~~	642	93.10	7.92	7.3
B. intermedius (n=43)	37	5-10	~~299~~318	86.05	8.59	7.4
**Overall (N=265)**	**234**	**4-10**	**1748~~48~~**	**88.30**	**7.77**	**6.8**

~~The result indicated that 56.3% of male and 43.7% of female in *O. niloticus*, 52.87% male and 47.3% female in *C. gariepinus*, and 55.81% male and 47.13% female in *B. intermedius* were affected by one or more parasites.~~ The smallest body weighted fishes are highly affected by one or more parasites (90.37% in 100-300g, 75.86% in 190-400g and 79.09% in 100-200g body weight in *O. niloticus*, *C. gariepinus*, and *B. intermedius*), respectively. This body weight class is the lower body weight in respective of fish species. Conversely, the parasite infestation increases as body length of the fish host increase. Accordingly, 70.37%, 95.40% and 93.02% of *O. niloticus*, *C. gariepinus*, and *B. intermedius* respectively are infected by fish parasites at the larger standard body length. The distribution of parasites with sex, body weight, and the total and standard length of *O. niloticus*, *C. gariepinus* and *B. intermedius* fish showed not statistically significant association (Table 2).

Formatted: Width: 8.5", Height: 11"
Formatted: Font: Bold
Formatted: Line spacing: Multiple 1.15 li
Formatted: Not Highlight
Formatted Table
Formatted: Not Highlight
Formatted: Line spacing: Multiple 1.15 li
Formatted: Not Highlight
Formatted: Line spacing: Multiple 1.15 li
Formatted: Not Highlight
Formatted: Not Highlight
Formatted: Line spacing: Multiple 1.15 li
Formatted: Not Highlight
Formatted: Font: Not Italic, Not Highlight
Formatted: Not Highlight
Formatted: Not Highlight
Formatted: Line spacing: Multiple 1.15 li
Formatted: Line spacing: Multiple 1.15 li

Table 2: Parasitic infestation in relation to sex, bodyweight, total and standard length of fishes

Species of Fish studied	Variable category		Number of examined	Number of fishes infected by parasites	Prevalence (%)	χ2	P-valu
O. niloticus (n=116)	Sex	Male	76 (56.3)	64	84.21	0.423	0.515
		Female	59 (43.7	52	88.14		
	Body Weight (gram)	100-300	122 (90.37)	105	86.07	2.382	0.304
		301-500	11 (8.15)	10	90.91		
		>500	2 (1.48)	1	50.00		
	Total Length (in centimetres~~which measurement?~~)	10-16	32 (23.70)	27	84.38	0.289	0.865
		17-25	92 (68.15)	79	85.87		
		>25	11 (8.15)	10	90.91		
	Standard Length (in centimetres)	<10	4 (2.96)	3	75.00	0.757	0.685
		10-15	36 (26.67)	30	83.33		
		>15	95 (70.37)	83	87.37		
C. gariepinus (n=81)	Sex	Male	46 (52.87)	42	91.30	0.493	0.483
		Female	41 (47.13)	39	95.12		
	Body Weight	190-400	66 (75.86)	62	93.94	0.609	0.737
		401-2000	19 (21.84)	17	89.47		
		>2000	2 (2.29)	2	100.00		
	Total Length (in centimetres)	28-50	85 (97.70)	80	94.12	5.923	0.052
		51-75	2 (2.29)	1	50.00		
		>75	0 (0.00)	0	0.00		
	Standard Length (in centimetres)	<25	4 (4.59)	3	75.00	2.140	0.343
		25-70	0 (0.00)	0	0.00		
		>70	83 (95.40)	78	93.98		
B. intermedius (37)	Sex	Male	24 (55.81)	21	87.50	0.096	0.757
		Female	19 (44.19)	16	84.20		
	Body Weight	100-200	34 (79.07)	31	91.18	4.155	0.125
		201-460	7 (16.28)	5	71.43		
		>460	2 (4.65)	1	50.00		
	Total Length (in centimetres)	15-20	3 (6.98)	2	66.67	1.027	0.598
		21-26	31 (72.09)	27	87.09		
		>26	9(20.93)	8	88.89		

Formatted: Font: Not Bold
Formatted Table
Formatted: Line spacing: Multiple 1.15 li
Formatted: Font: 12 pt
Formatted: Font: 12 pt
Formatted: Line spacing: Multiple 1.15 li
Formatted: Font: 12 pt
Formatted: Font: 12 pt
Formatted: Line spacing: Multiple 1.15 li
Formatted: Font: 12 pt

	Standard Length (in centimetres)	<10	1 (2.33)	1	100.00	2.397	0.302
		10-20	2 (4.65)	1	50.00		
		>20	40 (93.02)	35	87.50		

Prevalence of parasitic infestations with respect to anatomical locations

A total of 265 fish were examined, including *Oreochromis niloticus* (n = 135), *Clarias gariepinus* (n = 87), and *Barbus intermedius* (n = 43), with an overall infection/infestation rate of 88.3% (n = 234). In total, 1,748 parasites were collected and classified into nematodes, digenean trematodes, monogeneans, myxozoans, crustacean ectoparasites, and leeches. The most common nematodes were Contracaecum spp., particularly prevalent in *C. gariepinus* (50.57%). Digenean trematodes included Clinostomum, Euclinostomum, Diplostomum, and encysted metacercariae, with the latter reaching 68.97% in *C. gariepinus*. Monogenean infections with *Dactylogyrus* spp. occurred mainly in *O. niloticus* (28.15%). *Myxobolus* spp., leeches, Argulus, and Dolops species were also recorded across hosts. Mean parasite intensity was 8.59 in *B. intermedius*, 7.92 in *C. gariepinus*, and 6.79 in *O. niloticus*, while mean abundance was 7.40, 7.37, and 5.84, respectively.

Clinostomum species (figure ~~3~~ 2 in the annex 1) were the dominant trematodes in *O. niloticus* with a prevalence of 65.19% infecting brachial cavity, pericardial cavity, ~~pre~~peritoneal ~~mium~~ and ocular cavity, followed by *Euclinostomum* species (figure ~~4~~ 3 in the annex1) with 34.07% which infects kidney, liver and brachial cavity ~~(figure 3)~~. *Dactylogyrus* species (figure ~~5~~ 4 in the annex 1) from the gill and *Diplostomum* species (figure ~~6~~5) from the brain tissue also showed higher prevalence, accounting 28.15% and 24.44%, respectively. *Contacaecum* species (figure ~~7~~6) one of the zoonotic anisakidae infecting 26.67% of *O. niloticus* species (Table 3). *Digenean encysted metacercaria* 68.97%, *Contacaecum* species 50.57% and *Diplostomum species* 47.13% infecting musculature, mesentery and brain tissues respectively are the dominant helminths infecting *C. gariepinus* groups. ~~This prevalence report is calculated considering multiple parasite infestation from single host i.e, one fish could be infected by different parasite groups or free from any parasite~~. *Contracaecum* species (37.21%) and *Dactylogyrus* species (62.79%) are the dominant parasites affecting *B. intermedius* infecting mesentery and gills respectively. The dominant helminth in *C. gariepinus* was recorded as digenean encysted metacercaria (68.97% prevalence). Identification to genus level was not possible due to heavy encapsulation within host tissue, which obscured key morphological features required for precise taxonomic determination. Given the high intensity and overlapping morphology of the cysts, these parasites were conservatively categorized as digenean metacercariae. In contrast, *O. niloticus* harboured trematodes such as *Clinostomum* and *Euclinostomum*, which were identifiable based on well-preserved sclerotized structures. The

Formatted: Font: 12 pt
Formatted: Font: 12 pt
Formatted: Line spacing: Multiple 1.15 li
Formatted: Font: Italic
Formatted: Font: Italic
Formatted: Font: Italic
Formatted: Font: Italic
Formatted: Font: Italic
Formatted: Font: Italic
Formatted: Font: Italic
Formatted: Font: Italic
Formatted: Font: Italic
Formatted: Font: Italic
Formatted: Font: Italic
Formatted: Not Highlight

common ectoparasites infecting fish species in Lake Hawassa are Argulus species (figure 8)7 and *Dolops* species (figure 9 8 'a' and 'b' in annex 1) (Table 3).

Table 3: Prevalence with respect to the recovered parasites (n=number fishes sampled in each group).

Variables	Site of localization	Parasite species recovered	Number of infected fish	Prevalence (%)
O. niloticus (n=135)	*Clinostomum* species	Branchial cavity, pericardial cavity, between cranium and pharyngeal teeth, ocular cavity	88	65.19
	Euclinostomum species	Kidney, liver, branchial cavity	46	34.07
	Contracaecum species	Heart, pericardial Cavity	36	26.67
	Diplostomum species	Brain tissue	33	24.44
	Dactylogyrus species	Gill	38	28.15
	Myxobolus species	Intestinal contents	20	14.81
	Leeches	Skin, gills, mouth	16	11.85
	Argulus species	Skin, fins	9	6.67
	Dolops species	Gill, fins	5	3.70
C. gariepinus (n=87)	*Digenean encysted metacercaria*	Musculature	60	68.97
	Contracaecum species	Mesentery	44	50.57
	Diplostomum species	Brain tissue	41	47.13
	Unidentified parasite	Mesentery	1	1.15
	Dactylogyrus species	Gill	41	47.13
	Encysted cestode larvae	Stomach	5	5.75
	Leeches	Skin, gills, mouth	7	8.04
	Argulus species	Skin, gill, fins and oral cavity	18	20.69
	Dolops species	Skin, gill, fins,	38	43.68

Formatted: Font: Font color: Green

Formatted: Normal (Web), Line spacing: Multiple 1.15 li

Formatted Table

Formatted: Line spacing: Multiple 1.15 li

Formatted: Line spacing: Multiple 1.15 li

		Oral cavity		
B. intermedius (n=43)	*Contracaecum* species	Mesentery	16	37.21
	Unidentified parasite	Mesentery	2	4.65
	Dactylogyrus species	Gill	27	62.79
	Leeches	Skin, gills, mouth	5	11.63
	Argulus species	Skin, fins	3	6.98
	Dolops species	Skin, fins	4	9.30

Distribution, location and number of ectoparasites on the fishes sampled.

Contracaecum species is the most common parasites found on *O. niloticus with* 373 total number of parasite collected from 224 infected fishes with mean intensity of 3 parasites per fish. Different parasites have different affinity for the body of the fishes where the external parasites mostly affecting gills, skin and gills. *Leeches* are also found infecting all the three fishes studied (Table 4).

Table 4: Anatomical distribution of the different parasites

**Type of parasite**	**Parasite group**	**Anatomical location of infection**	**Number of fish infected**	**Number of parasites collected**
Clinostomum species	Trematode	Branchial cavity, pericardial cavity, between cranium and pharyngeal teeth, ocular cavity	88	255
Euclinostomum species	Trematode	Kidney, liver, branchial cavity	46	136
Contracaecum species	Nematode	Heart, pericardial cavity	124	373
Diplostomum species	Trematode	Brain tissue	74	200
Dactylogyrus species	Trematode	Gill	106	276
Myxobolus species	Protozoa	Intestinal contents	20	88
Digenean encysted metacercaria	Trematode	Musculature	60	189
Encysted cestode larvae	Cestode	Stomach	5	19
Argulus species	Crustaceans	Skin, fins	30	79
Dolops species	Crustaceans	Gill, fins	47	96

Leeches	Hurindae	Skin, gills, mouth	28	34
Unidentified internal parasite		Mesentery	1	3
**Total collected parasites**				**1748**

Formatted: Line spacing: Multiple 1.15 li

Formatted: Line spacing: Multiple 1.15 li

Formatted: Line spacing: Multiple 1.15 li

Formatted: Justified, Line spacing: Multiple 1.15 li

Formatted: Line spacing: Multiple 1.15 li

Discussions

Formatted: Font: Not Bold

The commercially important fish species: O. *niloticus, C. gariepinus,* and *B. intermedius* from the Hawassa lake are known to be infected with parasites from nematodes, cestodes, trematodes, crustaceans, protozoan families and leeches (table 1-4, figure 32-87). Some of those parasites are also pathogenic to humans and animals. Some of these parasites are known zoonotic pathogens to humans (Ziarati et al., 2022). The majority of animal parasites are found in fish and even apparently health wild fish have some parasites burdens (Petty et al., 2022).

Parasitic diseases are a major constraint to the sustainable development of aquaculture worldwide (Shafiq et al., 2023). In Ethiopia, endoparasites are commonly reported in fish from Lake Awasa, Lake Lugo, and Lake Tana (Gebremedhin et al., 2020). Globally, parasites are recognized not only as pathogens but also as ecologically significant organisms that can impair host growth and reproduction, including causing parasitic castration (de Montaudouin et al., 2012; Marcogliese, 2004; Poulin et al., 2014; Sures and Nachev, 2022). Among aquatic diseases, parasitic infections are the most prevalent, accounting for approximately 54% of cases, compared to 22% attributed to bacterial infections (Okon et al., 2024).

Formatted: Justified

Various disease and parasites are common and the major limitation to the development and sustainably of aquaculture (Shafiq et al., 2023). According to reports, fish endoparaites from Lake Awasa, Lake Lugo, and Lake Tana are common in Ethiopia (Gebremedhin et al., 2020). Prior research reports from other world indicated that parasites, which cause castrations and restrict the growth of their hosts (de Montaudouin et al., 2012), have an ecologically significant role in marine environments (Marcogliese, 2004; Poulin et al., 2014; Sures and Nachev, 2022). Parasitic disease are the common infections in aquatic environment accounting 54% followed by bacterial infections which accounts only 22% (Okon et al., 2024).

Formatted: Not Highlight

Formatted: Line spacing: Multiple 1.15 li

The current prevalence of fish parasites was found to be 88.30%, differing with fish species, sex, body weight, standard length. This result agreed with Mitiku, (2017) who indicated 83.4% prevalence of parasites from central Ethiopia from freshwater culture and capture (which lake? As it is your center of discussion). Similar prevalence report of 77.4% to 88.3% was reported (Outa et al., 2021) inVictoriain Victoria Lake (Outa et al., 2021), Kenya, 88.2%-91.2% by Mgbemena et al., (2020) from Nigeria. The current finding is found greater than earlier reports of 79% by Zekarias and Yimer, (2007) at Lake Hawassa, 69.25% by Tilahun and Behonegn, (2017) at Lake Ziway, 66.3% by Gulelat *et al*., (2013) at Koka reservoir.

Formatted: Justified

Formatted: Not Highlight

The present prevalence (88.30%) was markedly higher than previous reports from several Ethiopian and regional water bodies, including Lakes and farms Taita Taveta county of Kenya (52.3%), Lake Hashange (37.6%) (Tesfaye et al, 2024), Lake Baringo (34.8%) (Kiprono, 2017), Jimma (30.9%)(Gebreegziabher et al., 2020), Lake Chamo (21.4%)(Kaba et al., 2024), and Lake Ziway (20.8%)(Bekele and Hussein, 2015). Similarly, lower prevalence rates were reported from Taita Taveta County, Kenya (37.6%), and Kiri Reservoir, Nigeria (37.5%) (Vandi and Daniel,

Formatted: Normal (Web)

2023). These findings indicate that the current study area exhibits comparatively elevated infection levels relative to earlier investigations. ~~However, it is found significantly greater than finding by Tesfaye *et al*., (2017) who reported 37.6% at Lake Hashange, 34.8% by Kiprono, (2017) at Lake Baringo, 30.9% by Gebreegziabher *et al*., (2020) in Jimma, 21.4% by Kaba et el., (2024) in *O. niloticus* and *L. niloticus* from Chamo lake and 20.8% by Bekele and Hussien, (2015) in Lake Ziway. Lower prevalence rate as compared to the current finding of 52.3% was reported by Ageng'o et al., (2025) at farms and lake in Taita Taveta county of Kenya and 37.5% at Kiri reservoir of Nigeria (Vandi and Daniel, 2023).~~ Furthermore, higher prevalence reports of 93% was reported from Midmar reservoir, Adwa, Northen Ethiopia by Tesfay et al, (2024) in *O. niloticus*.

The difference in parasite prevalence among different studies may be attributed to ecological, methodological, and host related factors. difference in water quality, agroecological and seasonal variation can influence parasite survival, intermediate host, and transmission dynamics. Variation in fishing species composition, feeding habitats, age, sex, body structure, and the immune status may also contribute for differing susceptibility (Turner et al., 2021).

The prevalence, mean intensity and mean abundance of the parasites ~~varies~~vary with species of the fish's host. Accordingly, prevalence rate of 85.93%, 93.10% and 86.05% is registered in O. *niloticus*, *C. gariepinus*, and *B. intermedius* respectively. In the current finding, the prevalence rate is slightly higher in *C. gariepinus* as compared to O. *niloticus* and *B. intermedius*. Similar finding was reported by Tesfaye *et al*, (2020) in which the rate of parasitic infection is higher in *C. gariepinus*. Additionally, *C. gariepinus* species are among the most frequently infected fish species, according to Gulilat et al, (2013). Trute et al., (2023) also reported the presence of over 107 metazooan parasites are known to infect *C. gariepinus* in Africa with well characterized life cycle and general morphology.

Mean intensity and apparent parasite density varied among fish species, with the highest values recorded in *B. intermedius* (8.59; 7.40), followed by *C. gariepinus* (7.92; 7.37) and *O. niloticus* (6.79; 5.84). Similar findings have been reported by Oztruk and Alutnel (2006), who observed that parasite intensity and prevalence differ according to host size. These variations are influenced by abiotic factors such as water temperature and host-related factors including behaviour, age, sex, resistance, and mortality (Bakke et al., 2002; Ozer et al., 2004; Khidr et al., 2012).

~~The mean intensity and apparent density of parasites also vary with different fish species. Accordingly, 8.59, 7.92, and 6.79 and 7.40, 7.37 and 5.84 was reported in *B. intermedius, C. gariepinus* and O. *niloticus* respectively. Similarly, Oztruk and Alutnel, (2006) discovered that the parasite's mean intensity level and prevalence changed depending on the size of the host fish. Abiotic variables like water temperature and host-related variables like behaviour, sex, age, resistance, and mortality~~

Formatted: Not Highlight

Formatted: Line spacing: Multiple 1.15 li

Formatted: Font: (Default) Times New Roman

Formatted: Font color: Custom Color(RGB(27,27,27))

Formatted: Line spacing: Multiple 1.15 li, Pattern: Clear (White)

Formatted: Line spacing: Multiple 1.15 li

Formatted: Left, Indent: First line: 0", Space Before: Auto, After: Auto

Formatted: Indent: First line: 0", Line spacing: Multiple 1.15 li

~~are important determinants of the seasonal prevalence and mean intensity of parasites (Bakke et al., 2002, Ozer et al., 2004, Khidr et al., 2012).~~

The higher prevalence of internal helminths in the current study could be attributed to a number of factors, including the lack of an effective waste disposal and management system, which led to the processing of post-harvest fish and the disposal of the waste (scraps and gastrointestinal contents) into the lake and its shorelines; environmental pollution from detergents used for washing clothes on the lake shore; and some birds that could serve as intermediate or final hosts for helminths. This shows that the host-parasite interaction and changes in abiotic factors, including sewage, irrigation, sanitation, and flooding, frequently causes the distribution of fish parasites to vary from one habitat to another, and that leads to environmental stresses that impair the host's immune system (Tibebe et al, 2023). Compared to mammals, fish have a lower immune response to foreign proteins (Pradipta et al, 2012; Ardelli and Woo, 1997). Several parasites are known to alter the behaviour of their hosts, predisposing them to predation (Labaude et al, 2015). The difference in prevalence from different lakes in Ethiopia and other parts of the world may be attributed to the difference in behaviour of different fish species, the quality of water, the presence of other stressors such as pollution and climate change (Okon et al., 2024). The increase in global temperature leads to fluctuation in aquatic environment whereas the elevated water temperature could potentially compromise the fish immune system leading to fish more vulnerable to disease conditions (Grossart et al., 2019; Kaur et al., 2022).

In the present study, parasite prevalence was higher in males than females across *O. niloticus*, *C. gariepinus*, and *B. intermedius*. These values were lower than reports from Nigeria (Mgbemena et al., 2020) but higher than those from Lake Hashenge, Ethiopia (Tesfaye et al., 2017), and were comparable to findings from Nigeria and Upper Egypt. Although some studies report higher infection in females, this has been attributed to behavioural factors such as increased shoaling and prolonged parasite exposure ~~In the current study the prevalence of the parasite is relatively higher in male fishes (56.3%, 52.87%, 55.81%) than female (43.7%, 47.13%, 44.19%) in *O. niloticus, C. gariepinus* and *B. intermedius* respectively. This finding is lower than the prevalence report of 81.8% in male and 93% in female by (Mgbemena et al., 2020) from Nigeria. However, it is significantly higher to finding of Tesfaye et al., (2017) who reported 12.8% in male and 24.8% in female from Lake Hashenge, Ethiopia. Similar prevalence report of 47.73% in male and 52.27% in female was reported by (Vandi and Daniel, 2023) from Nigeria and 60% in male 40% prevalence from female in upper Egypt (Abd-ELrahman et al., 2023). It is stated in other finding that female is more likely exposed to parasitic worms due to behaviours such as higher rate of shoaling which can lead more frequent and prolonged contact with parasites~~ (Simková et al, 2005; Abd-ELrahman et al., 2023).

In the current finding the presence of parasites varies with body weight and standard length. Accordingly, the lower body weighted fishes are highly affected by one or more parasites. This finding is consistent with earlier finding that indicate there is a general trend that smaller fish or those with poor body condition may be susceptible to parasitic infections although it is not universally true. This can be due to several factors including inability to allocate enough defence

Formatted: Left, None, Indent: First line: 0", Space After: Auto

Formatted: Normal, Space Before: Auto, After: Auto

Formatted: Normal (Web), Left, None, Space Before: 0 pt

Formatted: Pattern: Clear

Formatted: Line spacing: Multiple 1.15 li

and behavioural adaptations in lower body condition fishes (Abd-ELrahman et al., 2023; Hasegawa et al., 2025). How ever several biological parameter or ecological processes determine which types of parasites are more likely to correlate closely with host size (Poulin, 2005). In other terms larger fish usually have larger fish population and wider range of parasitic species due to the availability of resources and greater capacity to harbor variety of parasites. Smaller fish however may be more vulnerable to specific larval stages of parasites potentially due to their exposure to predation (Leung, 2022). Although parasite reduces host health, parasite infection can occur due to compromised host health (Hasegawa et al., 2025). Poulin, (2014) indicated host size (and age) is generally known to be positively associated with parasite species richness and infection levels in host individuals. Nikolaev *et al*, (2006) also concluded that as age increase there is higher chance of accumulating parasites during their lifetime. In the current finding it is found difficult to harvest and investigate old age and large body weight fishes since illegal fishing along with other abiotic factors are highly practiced and may posing threat including decline in number of fish or yield of the lake Hawassa. The research team has clearly observed the illegal fishing practice and the practice of eviscerating fish along the shore of their lake which increase the risk of re-infection.

Identification of parasites with significant host and tissue specificity is made much easier by knowledge of fish hosts, while other parasites are detected due to their widespread presence and lack of host specificity (Petty et al., 2022). In line with this, *Contracaecum* was the most frequently encountered nematode parasite found in the current study. It is found 26.67% in *O. niloticus* to 50.57% in *C. gariepinus.* This finding is lower than previous reports by Zekarias and Yimer, (2007) in Lake Hawassa, who reported 39.67% in *O. niloticus*, 80.5% in *C. gariepinus*, and 36% in *B. intermedius*. ~~Moreover~~Moreover, Kaba et al., (2024) reported lower prevalence of 21.4% from *O. niloticus* and *Lates niloticus* from Chamo Lake, Arbaminch. However, it is higher as compared to Gulelat *et al.* (2013) at Koka reservoir, 24.82% in *C. gariepinus* and 16.49% in *B. intermedius*. This result suggests that the distribution of *Contracaecum's* parasites can vary from one habitat to another due to host parasite relationships and changes in water quality parameters. Recently *Contracaecum bancrofti* a unique Australian species and warming the fish tissue leads to emergence of small *Contracaecum* larvae <1 mm from intestinal tissue (Shamsi *et al*, 2018). *Contracaecum* are economically important parasites with zoonotic significance where human infection through ingestion of infected tissue is the major route (Kaba et al., 2024). Adult *Contracaecum* spp. are found in the stomach of marine mammals or piscivorous birds, and larval stages infect a wide range of invertebrates and fish species (Shamsi et al, 2017). Study from lake Chamo show that a very high proportion of people (82%) consume raw fish especially locally preferred species such as *Oreochromis niloticus* and *Lates niloticus*, and that consumer awareness of fish-borne zoonoses is generally low (Kaba et al., 2024). Several other studies have also reported human infection due to *Contracaecum* larvae following consumption of infected fish (Shamsi *et al*, 2018; Shamsi and Barton, 2023~~; Kaba et al., 2024~~). In lake Hawassa area similar pattern was practiced where humans eat fresh uncooked fish which might be infected with zoonotic parasite.

The host distribution of *Clinostomum* species was discovered in the current study to be much higher than previous studies by Gulelat *et al.* (2013), Reshid *et al.* (2015), and Tesfaye *et al.* (2017), who revealed a prevalence of 27.39%, 18.8%, and 15.2% in Lake Abaya, Koka Reservoir, and Lake Hashange, respectively. However, the prevalence of this parasite is lower than that found by Zekarias and Yimer, (2007) who reported 75.7% prevalence from Lake Hawassa. The variations may result from variations in the physiochemical characteristics of water bodies and the intermediate hosts of the parasite. In other way another digenean fluke, *Euclinostomum* species from *O. niloticus* were detected in the kidney, liver, and branchial cavity. This finding was in line to previous reports from Ethiopian lakes and water bodies.

A prevalence of 30% was reported by Zekarias and Yimer (2007) from Lake Hawassa, whereas much lower rates were documented in Koka Reservoir (2.73%) and Jimma (3.9%). In contrast, Mitiku (2017) recorded a higher prevalence of 49.35% for *Euclinostomum*. These variations are likely influenced by differences in parasite–host life cycles and environmental conditions affecting transmission dynamics.

~~Accordingly, 30% report was made by Zekarias and Yimer, (2007) from Lake Hawassa but significantly lower prevalence rate of 2.73% was reported by Gulelat *et al.* (2013) in Koka Reservoir and 3.9% by Gebreegziabher *et al.* (2020) in Jimma. However, Mitiku, (2017) recorded 49.35% of the *Euclinostomum*. This difference is mostly caused by the parasite and hosts life cycles and other environmental factors.~~ Moreover, Khan et al, 2018 reported that prevalence of 70-92% metacercarial infection belonging to *C. complanatum* was reported concluding the seasonality of the infection. He stated that the high prevalence of infection of the parasite during summer may be because of the time of emergence of cercariae in high temperature from snail vector and the environment. Digenetic trematodes, or flukes, belonging to the genera *Clinostomum* and *Euclinostomum* infect many fish species all over the world. These parasites are found in freshwater and saltwater habitats worldwide (Senapin et al., 2014). The coexistence of *Clinostomum* and *Euclinostomum* in some fish host could imply the shared intermediate hosts, environmental conditions, or ecological overlap (Choudhary et al,2022)

The digenetic trematode Clinostomum spp. commonly referred to as *yellow grubs* when in their larval form have complex and involves three hosts including freshwater snails as intermediate host, other fish or amphibians and piscivorous birds as definitive host. Humans are also accidental hosts if they consume undercooked or raw fish meal.

In fish, encysted metacercaria are not merely superficial, they often embed deeply into critical tissues such as musculature, gills and buccal cavity, and cranial and pericardial regions, cranial and pericardial regions causing necrosis, inflammation, fibrosis and oxidative response which could compromise immune function and metabolism (Salem et al., 2025).

Formatted: Not Highlight

Formatted: Justified, Space After: 0 pt, Line spacing: Multiple 1.15 li, Pattern: Clear

Formatted: Font: Times New Roman

Formatted: Justified, Line spacing: Multiple 1.15 li

Formatted: Line spacing: Multiple 1.15 li

Formatted: Justified, Line spacing: Multiple 1.15 li

Formatted: Justified, Line spacing: Multiple 1.15 li, No bullets or numbering

Formatted: Font: (Default) Times New Roman

Formatted: Justified, Line spacing: Multiple 1.15 li

Formatted: Line spacing: Multiple 1.15 li

Lower prevalence rate of *Diplostomum* species, 24.44 and 47.13% respectively was found in the brain tissue of *O. niloticus* and *C. gariepinus*. This finding is lower as compared to the previous studies 77.93% of the gill cavity of *O. niloticus* at Lake Baringo, Kenya (Kiprono, 2017)*;*83.3% from cranial cavity of *C. gariepinus* sampled at Tanzania (Mukama, 2008); 100% of the cranial cavity of *C. gariepinus* was from Lake Hawassa (Tadesse, 2009); 77.93% of the cranial cavity of *C. gariepinus* (Gulelat et al., 2013). Significantly lower prevalence rate of 1.03% *Diplostomum* species was reported from the eye of *B. intermedius* from Lake Abaya (Gulelat et al. (2013). According to the current findings, Degenian encysted metacercaria frequently infect *C. gariepinus* muscle. This finding is in line with the findings of Vidal-Martínez et al. (2012) and Galaktionov et al. (2006), who conclude that these parasites commonly affect fish in coastal marine systems, which exhibit complex life cycles that typically involve snails as first intermediate hosts, macroinvertebrates, such as bivalves and crustaceans, or vertebrates as second intermediate hosts, and fish, birds, or mammals as final hosts.

External parasites have strategic survival reasons to inhibit specific microhabitat on fish body. This specific microhabitat may provide availability of food, reproductive success, enhanced attachment or reduce the pressure of predator (Chew et al., 2024). in the current finding, the crustacean parasites *Argulus* and *Dolops* species were found infecting gills, fins, skin, and oral cavity of the three fish species. This finding is lower as compared to the previous study by Zekarias and Yimer, (2007) in Lake Hawassa, who found 5.33% and 8.33% in *O. niloticus*; 44.5% and 63.5% in *C. gariepinus,* and 6% and 17 in *B. intermedius.* Moreover, Mitiku (2017) reported 7.8% of external parasites from *O. niloticus* at Sebeta ponds. Argus species is more commonly known as fish lice, are member of large groups of branchiuran parasite that infest and cause fish disease. They are crustaceans and crabs, lobsters, and shrimp. There are over 100 species infecting freshwater fishes with *Argulus foliaceus, A. coregoni, A. japonicus* common in freshwater fishes (Stecker and Yanong, 2024). According to a study by Trutera et al, (2022) *A. africanus* and *A. rhipidiophorus* are prevalent crustacean parasites in Ethiopian fish, especially those found in Lake Hawassa. *Argulus japonicus* is an alien species that has been introduced globally and is known to damage fish such as *C. gariepinus*, while it is not specifically listed as being present in Ethiopian fishes (Zekarias and Yimer, 2008, Trutera et al, 2022).

Dactylogyrus species was find residing in the gills of fishes. The current prevalence report of 3.7-43.6% was found higher than the reported 3.96% by Mitiku *et al.* (2018) in *O. niloticus* from *Gilgel* Gibe I Dam, and 31% by Borji *et al.* (2012) in common carp in the northeast of Iran from the gill of sampled fish. *Myxobolus* species from the intestinal contents of *O. niloticus* were also found in the current study, which was not reported elsewhere in Ethiopia. How ever the prevalence rate of *Myxobolus* species in the current finding was lower than previous reports by Abdel-Baki *et al*. (2015) in the Nile River, Egypt, and Folefack *et al*. (2019) in Cameroon. Higher

prevalence rate of *Myxobolus* infection including two new species was reported from Botswana (Reed et al, 2002).

In addition to the infections caused by parasites and other pathogens, several factors are threatening the quality of Hawassa lake from which human practice along the edge of the lake is the dominant factor. Memberu et al., (2021) have observed that the quality of the Hawassa lake is inadequate for maximum production of fishes and needs mitigation measures to control eutrophication and pollution flow. Anthropogenic activities, illegal fishing practices such as the use of narrow mesh nets, a lack of community awareness regarding fishery management, and a lack of fish stocks are the main conservation issues facing Ethiopian fish and fisheries sector (Chimdo, 2022).

The presence of parasites in edible part of the fish represent a public health threat since the presence of those parasites are responsible for causing infection in humans with significant outcome (Audicana and Kennedy, 2008). Knowledge of how parasites such as *Contracaecum* species, flukes and other pathogenic parasites are transmitted, their reservoir animal, their specific reservoir animals, the specific pathogen involved, directly impacts prevention and control strategy (WHO, 2020). The focus of control methods should be on health education to promote appropriate control measures including, and safely disposing of infected (Fomda et al., 2015). As prevention and control methods for each pathogen varies, however several practices are recognised as effective for reducing the risks at the community and personal level. Safe and appropriate guidelines for animal care in the agricultural sector helps to reduce the potential for food borne zoonotic disease outbreaks through ingestion of animal food or some vegetables. Standards for clean drinking water and waste disposal as well as protection for surface water in the natural environment are important and effective control means (WHO, 2020).

Limitation of the study

This study has not stablished the characterisation of the parasite at species level, and it only focuses describing the parasite at genera level. Moreover, water the impact of water quality on the effect of parasites have also not established

4. CONCLUSION AND RECOMMENDATIONS

In the current study, internal and external infestations were the predominant cases infecting fish with 88.03%. The parasites recovered belongs to *crustaceans, monogenean, digeneans, nematode, cestode, and one protozoan and Hirudinea* having a direct effect on fish health and meat quality and, thus, a direct effect on fish production and the economy. Among these, the Dactylogyrus species was the dominant external parasite followed by Argulus and Dolops species. It is found that parasite intensity and abundance vary with the host fish species. Anatomical and physiological variables of the fish such as body size, age, body weight and gender also found determining different prevalences. Our finding also showed that the common zoonotic nematodes

such as *Contracaecum* species and flukes are commonly found in the fishes at lake Hawassa necessitating coordinated efforts of public education. We found that human practice along the shore of the lake is also one of anthropogenic factor influencing fish health and productivity.

One of the limitations of this study is that it depends on visual examination rather than digestion methods or incubation techniques in which some small or deeply encysted parasites, early developmental stages, or low-intensity infections were not detected. It highlights that the parasite diversity and prevalence in Lake Hawassa may be even higher than reported, reinforcing the the need for further, more sensitive diagnostic approaches in future research.

Formatted: Space Before: Auto, After: Auto, Line spacing: Multiple 1.15 li, Pattern: Clear (White)

Formatted: Font: Not Italic, Font color: Custom Color(RGB(34,34,34))

Formatted: Line spacing: Multiple 1.15 li

Based on the above conclusion the following recommendations were forwarded:

- To improve parasite management, it is necessary to investigate and determine how abiotic elements affect the parasite-host interaction.
- Lake Hawassa needs to be kept apart from areas utilised for fishing and pleasure purpose.
- It is necessary to investigate the diversity of parasites over a wide range of hosts and water bodies.
- The wastes products including offals of the fish themselves must be disposed of properly and kept apart from water bodies.
- Molecular detection of each fish parasite to the genetic level needs to be carried out in future as the geographic bias exists in the discovery and genetic characterization of novel parasites.

Limitation of the study

A histopathological diagnosis with greater funding and resources does not support this study. Furthermore, it is necessary to evaluate how the evaluation of water quality affects fish parasites. Another drawback is that parasites can only be identified at the genus level.

Ethical approval and consent to participate

All methods were carried out in "accordance" with relevant guidelines and regulations. Furthermore, all methods are reported in accordance with "ARRIVE" guidelines (https://arriveguidelines.org) for the reporting of animal experiments. Ethical approval was obtained from the Research Ethics Committee of the School of Veterinary Medicine, Wollega University dated 20/04/2023 with minute number SVM.RERC/0023 before starting data collection. Parent/legal guardian provided informed consent on behalf of participants of age below eighteen years whereas oral consent was received from those above eighteen-year fishermen. The Research Ethics Committee of the School of Veterinary Medicine, Wollega University approved

all aspects of this study, including the informed oral consent process. Communicative letter was written to Hawassa City municipal and fishery sector leaders explaining the objective of this study. Data was collected after explaining the aims and methodology of the study. During data collection on fish all the welfare of animal was protected. It is assured that information collected was anonymous and that it was only for research purpose.

Data Availability statement: The data that support the findings of this study are available from the corresponding author upon reasonable request.

Conflict of interest: the author declares there is no conflict of interest

Funding importation: No funding was received for this research

Author's contribution: MA and DA collected data from the field, managed and analysed it, and participated in manuscript development and revision; YT, methodology, analysis, writing the original draft and editing final paper; DA, supervision, writing the original draft and editing final paper.

Acknowledgements: the author acknowledges Wollega University, Addis Ababa University and Hawassa University

Figures of fishes and their parasites (figure 12A-9B8B)

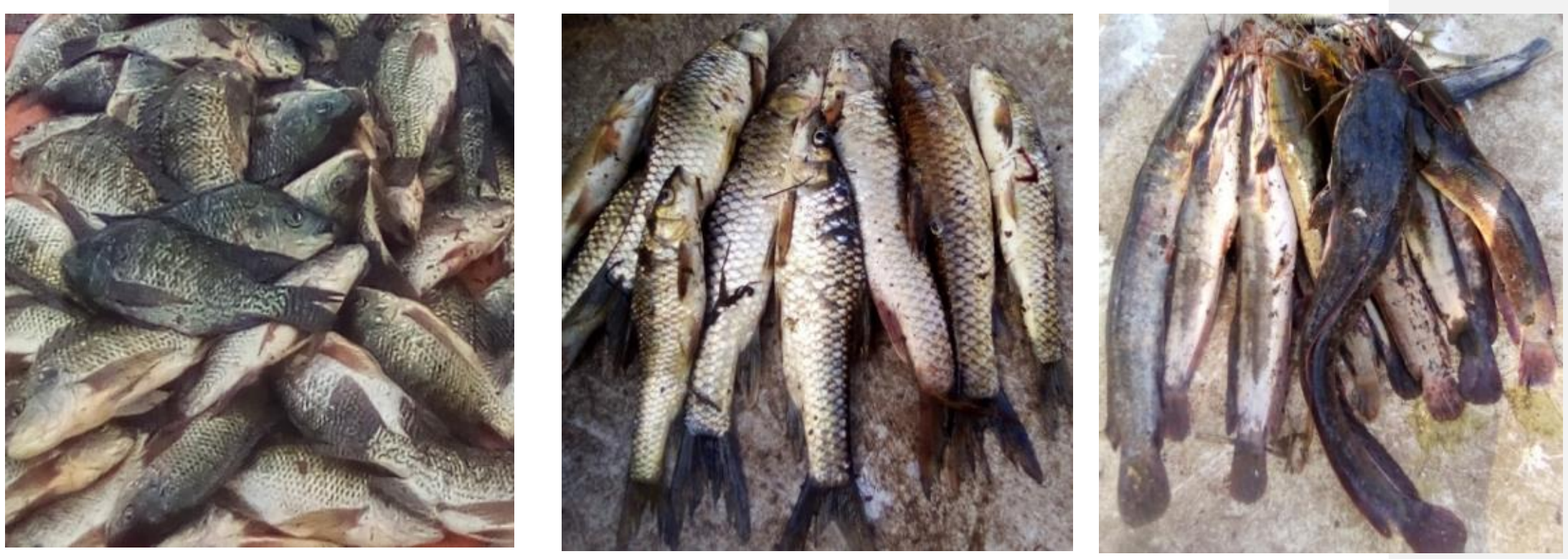

Figure 1: *O. niloticus* (Nile tilapia or Koroso) the first; *Barbus* species, the middle and *C. gariepinus* (Catfish or Ambaza), the third

Formatted: Font: Not Italic

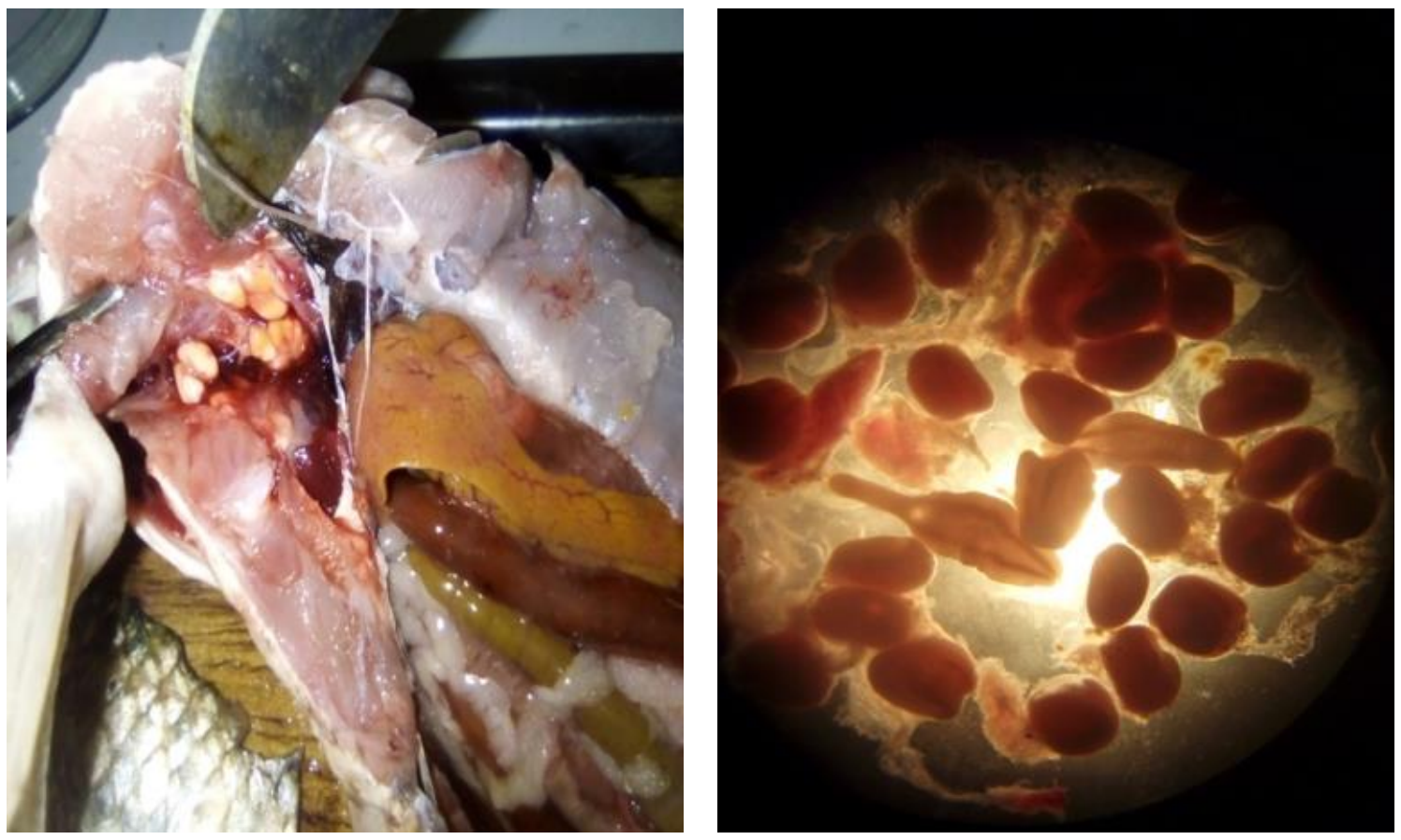

Figure ~~3~~2: Encysted (left) and Excysted (right) *Clinostomum* species

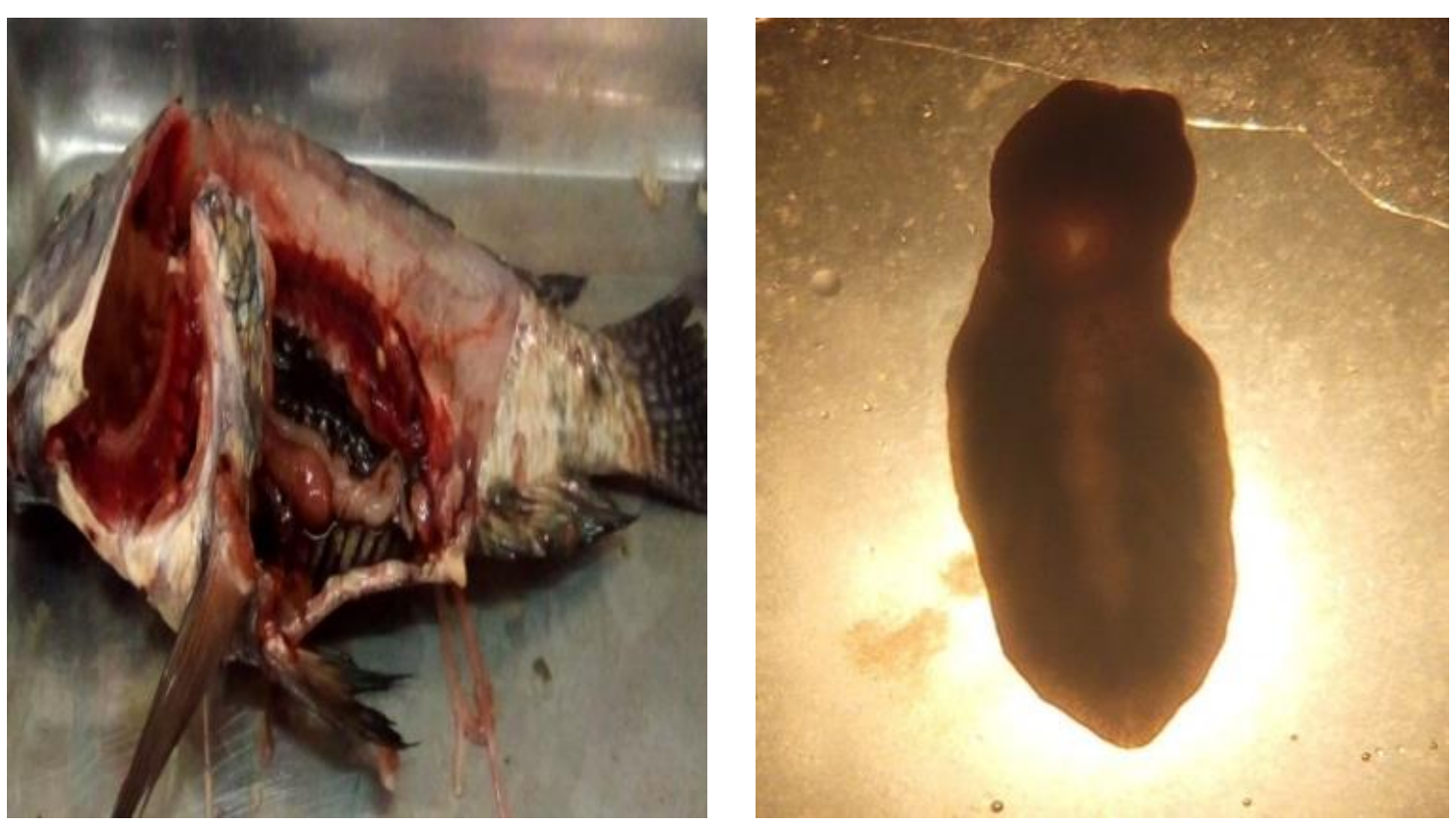

~~Figure4~~Figure3: Encysted (left) and excysted (right) *Euclinostomum* species on the kidney of *O. niloticus*

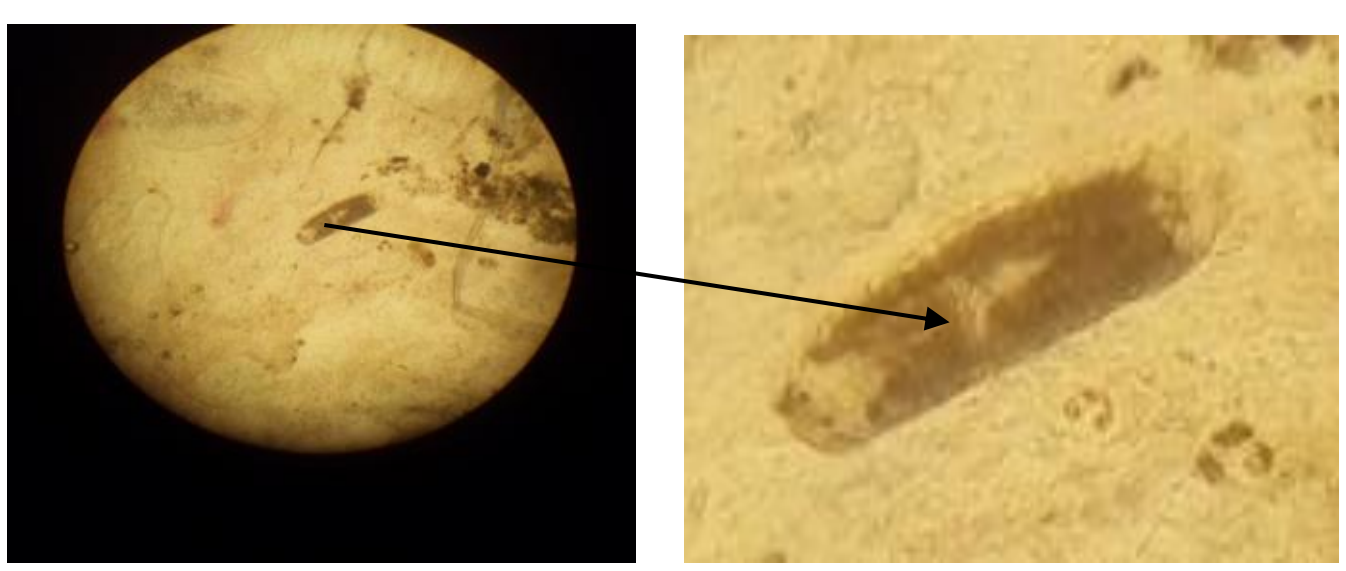

Figure ~~5~~4: *Dactylogyrus* species from the gill of Nile tilapia *(O.niloticus)*.

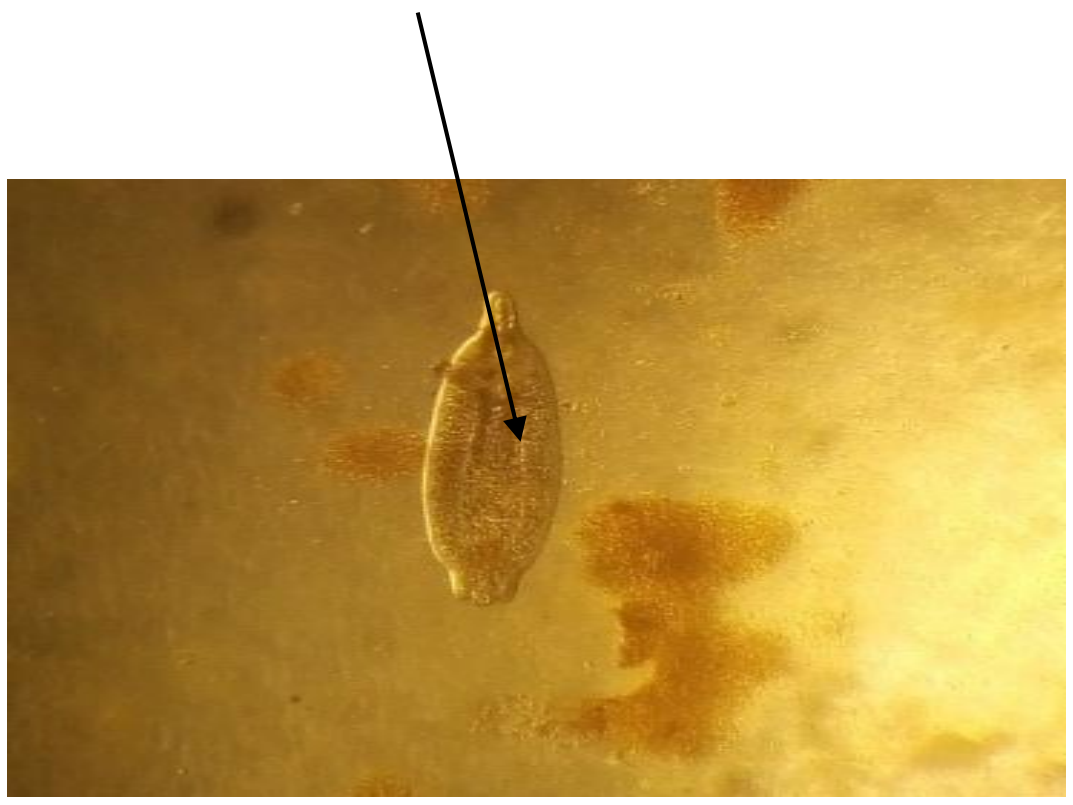

~~Figure6~~Figure5: *Diplostomum* species from the brain tissue of African catfish

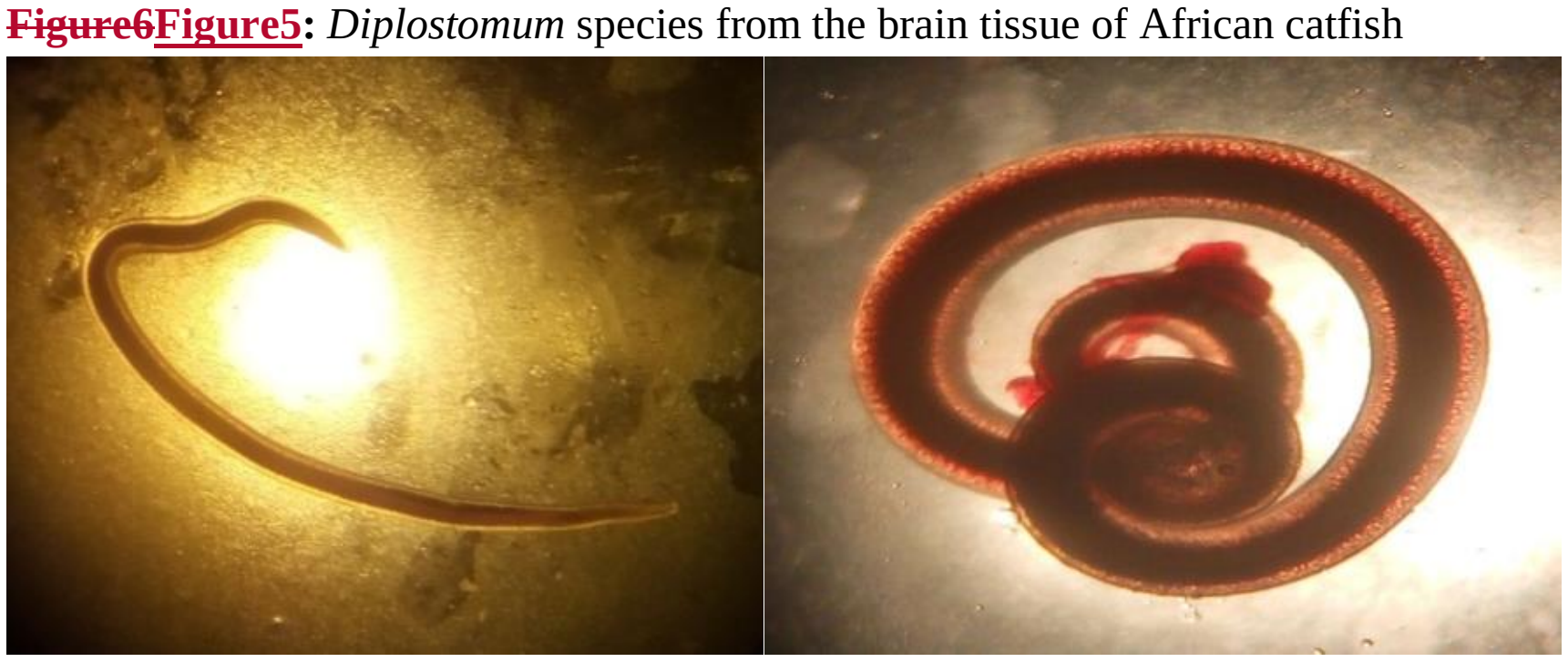

Figure ~~7a~~6a: *Contracaecum* species from the heart of Nile tilapia *(O. niloticus)*.

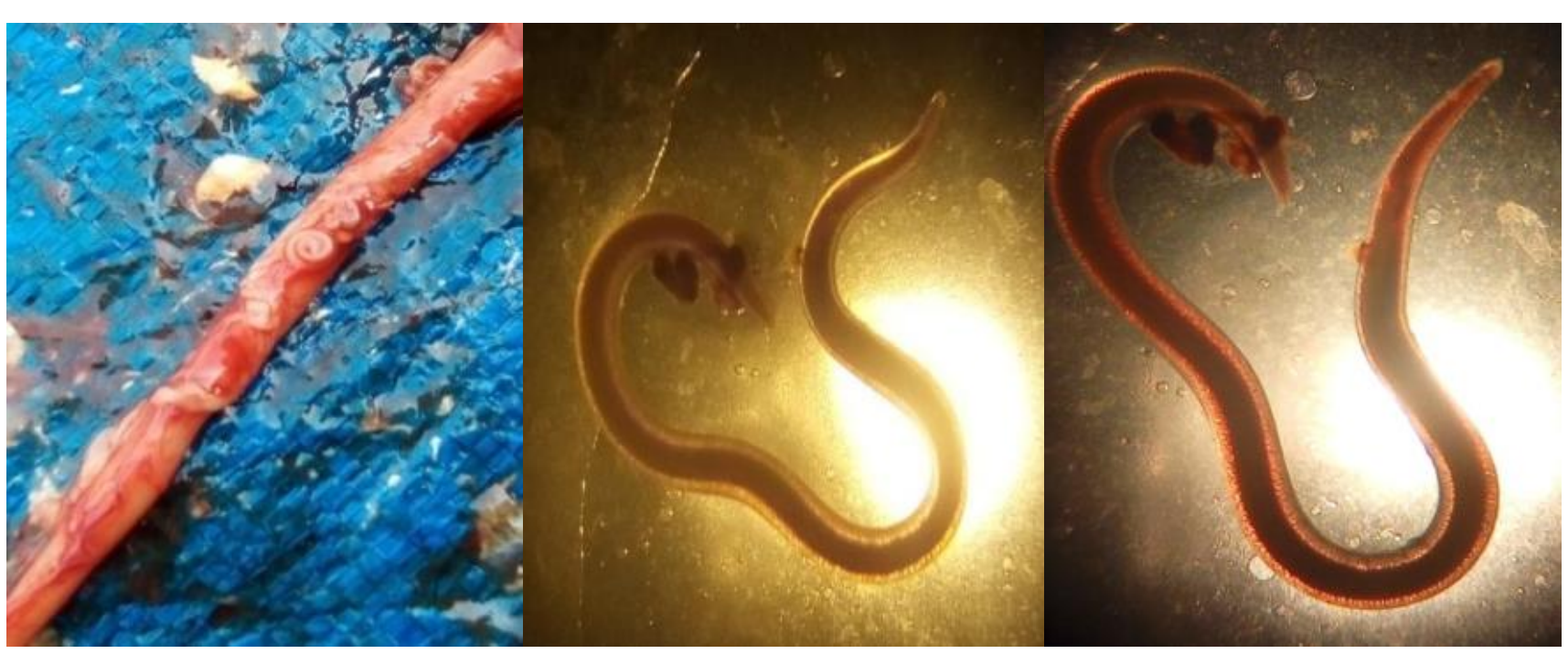

Figure ~~7b~~6b: *Contracaecum* species from mesentery of *C. gariepinus* (African catfish).

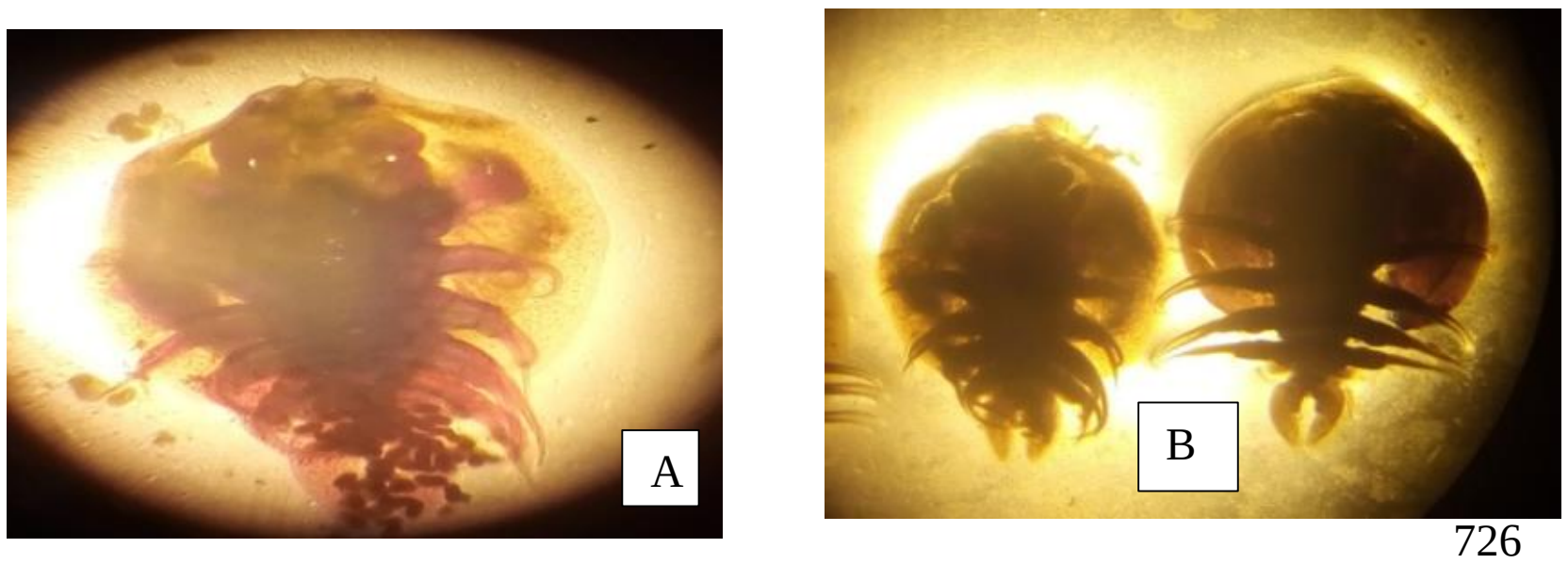

Figure 8: *Argulus* species (A: from the gill B: From fins of *C. gariepinus*)

Figure 7: *Argulus* species

(A: from the gill B: From fins of *C. gariepinus*)

Formatted: Font: 12 pt

Formatted: Justified, Line spacing: Multiple 1.15 li

Formatted: Font: 12 pt

Formatted: Line spacing: Multiple 1.15 li

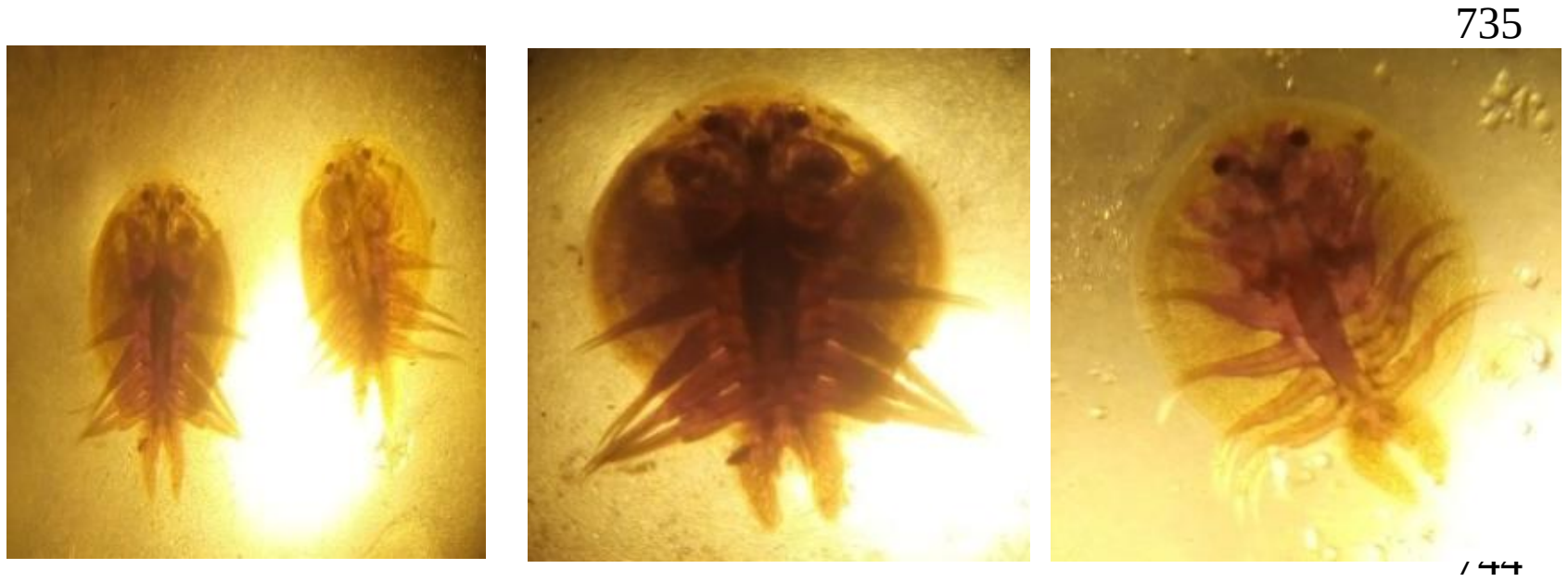

Figure ~~9a~~8a: *Dolops* species from the gill of *C. gariepinus*.

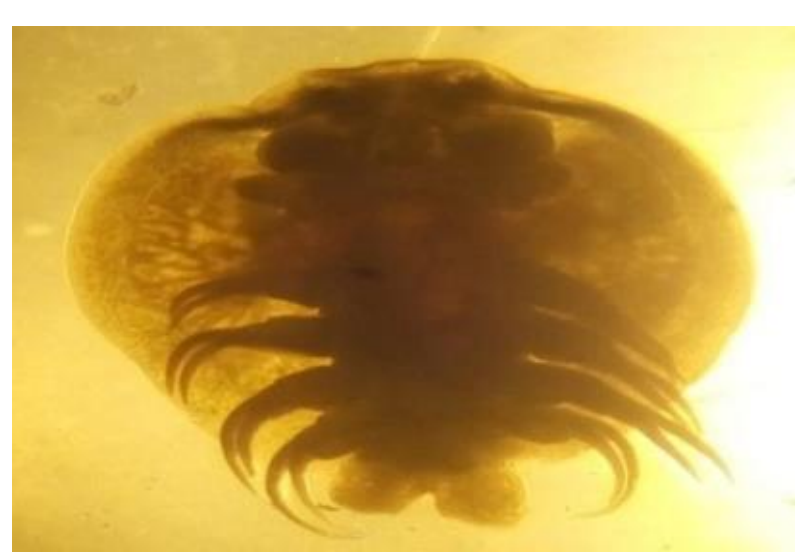

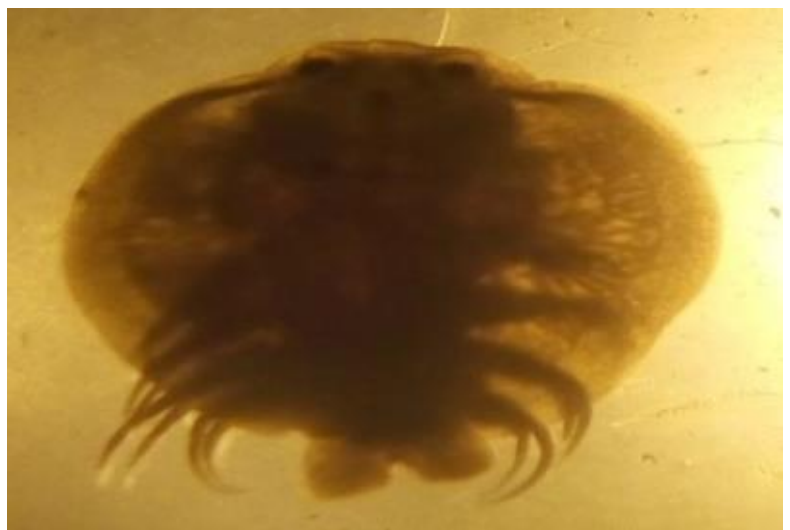

Figure ~~9b~~8b: Dorsal (left) and ventral view (right) of *Dolops* species from the skin of catfish~~.~~

REFERENCES

5.

Abd-ELrahman, S, M., Gareh, A, Mohamed, H,I.H, I., Alrashdi, B,M.B, M., Dyab, A,K.A, K., El-Khadragy, M,F., Khairy Elbarbary, N., Fouad, A,M., El-Gohary, F,A., Elmahallawy, E,K.E, K., Mohamed, S,A.,2023. Prevalence and Morphological Investigation of Parasitic Infection in Freshwater Fish (Nile Tilapia) from Upper Egypt. Animals (Basel), 18;13(6), https://doi.org/doi: 10.3390/ani13061088

Abdel-Baki, A.A.S., Abdel-Haleem, H.M., Sakran, T., Zayed, E., Ibrahim, K.E. and Al-Quraishy, S., 2015. Two Myxobolus spp. infecting the kidney of Nile tilapia (Oreochromis niloticus) in the River Nile at Beni-Suef governorate, Egypt, and the associated renal changes. *Parasitology research*, **114**(3), pp.1107-1112.

Ageng'o, F, A., Waruiru, R, M.,Wanja, D, W., Nyaga, P, N., Hamisi, M, M.,Ndegwa, J, M.,Ali, S, E., ChadagE., Chadag, M, V.,Mbuthia P, G.P, G.,2025. Parasites of Farmed and Wild Tilapine Fishes from Selected Farms and Lake Jipe in Taita Taveta County, Kenya, https://doi.org/10.1002/aff2.70042

Amlacher, E., 2005. Textbook of Fish Diseases. Narendra Publishing House, India, pp 207–292.

Ardelli, B. F. and Woo P. T. K., 1997. "Protective antibodies and anamnestic response in *Salvelinus fontinalis* to *Cryptobia salmositica* and innate resistance of *Salvelinus namaycush* to the hemoflagellate," *Journal of Parasitology*, vol. 83, no. 5, pp. 943–946, 1997.

Audicana, M, T., Kennedy, M, W.,2008. Anisakis simplex: from obscure infectious worm to inducer of immune hypersensitivity. Clin Microbiol Rev.;21(2):360-79, doi:10.1128/CMR.00012-07.

Bakke, T,A.,Harris, P,D.,Cable, J., 2002. Host specificity dynamics: observations on gyrodactylid monogeneans, Int. J. Parasitol., 32, pp. 281-308

Formatted: Font: Bold

Formatted: Line spacing: Multiple 1.15 li, Pattern: Clear (White)

Formatted: Line spacing: Multiple 1.15 li

Barber, I., Wright, H, A. 2005. Effects of Parasites on Fish Behaviour: Interactions with Host Physiology, Fish Physiology, Academic Press, 24,109-149, https://doi.org/10.1016/S1546-5098(05)24004-9

Bekele, J., Hussein, D., 2015. Prevalence of internal parasites of *Oreochromis niloticus* and *Clarius gariepinus*, journal of aquatic research and development,6(2), *http://dx.doi.org/10.4172/2155-9546.1000308*

Borji, H., Naghibi, A., Nasiri, M.R. and Ahmadi, A., 2012. Identification of Dactylogyrus spp. and other parasites of common carp in northeast of Iran. *Journal of Parasitic Diseases*, **36**(2), pp.234-238.

Boyd, C,E, McNevin AA, Davis RP. The contribution of fisheries and aquaculture to the global protein supply. Food Secur. 2022;14(3):805-827. https://doi.org/doi: 10.1007/s12571-021-01246-9.

Brian, J.I.,2023. Parasites in biodiversity conservation: Friend or foe? Trends Parasitol.; 39:618–621. https://doi.org/doi: 10.1016/j.pt.2023.05.005.

Brye, K, 2023. Aquaculture: A Sustainable Solution for Fish Cultivation and Global Food Security, Journal of Agriculture, 6(4),1-3

Chai, J.Y.; Murrell, K.D.; Lymbery, A.J.,2005. Fish-borne parasitic zoonoses: Status and issues. *Parasitol. Int.* **2005**, *35*, 1233–1254

Chew, X, Z., Cobcroft,J.Cobcroft, J., Hutson, K, S.,2024. Fish ectoparasite detection, collection and curation. Adv Parasitol, 125:105-157. https://doi.org/doi: 10.1016/bs.apar.2024.05.001

Chimdo, A. Review on potential and challenges of aquaculture practice in Ethiopia. *Appl Water Sci* **12**, 218 (2022). https://doi.org/10.1007/s13201-022-01740-1

Choudhary, K., Ray, S., Shamsi, S., Agrawal, N., *2022.* Characterization of *Clinostomum* (Digenea: Clinostomidae) spp. in India. *Parasitol Res* 121, 3083–3089 (2022). https://doi.org/10.1007/s00436-022-07644-y

CNN travel, 2023. Ethiopian food: the 15 best dishes, https://edition.cnn.com/travel/article/ethiopian-food-best-dishes-africa/index.html#:~:text=Eating%20fish%20%E2%80%93%20asa%20%E2%80%93%20in%20Ethiopia,a%20piranha%20on%20your%20plate

Dadebo, E., 2000. Reproductive biology and feeding habits of the catfish Clarias gariepinus (Burchell) (Pisces: Clariidae) in Lake Awassa, Ethiopia. *SINET: Ethiopian Journal of Science*, **23**(2), pp.231-246.

Deng, G, T.2020. Assessment of Factors Affecting Fish Production and Marketing in Gambella Region, Ethiopia. ScientificWorldJournal. 16; 2020:5260693. doi: 10.1155/2020/5260693.

Desalegn, T. and Shitaw, T., 2021.Fishery Resources, Conservation Challenges and Management Strategies in Ethiopia. *Fisheries and Aquaculture Journal*, **12**(3), pp.1a-1a.

El-Shahawy, I.S., El-Seify, M.O., Metwally, A.M., Fwaz, M.M. 2017. Survey on endoparasitic fauna of some commercially important fishes of the River Nile, southern of Egypt (Egypt) Rev. De Med. Vet.; 168:126–134.

Formatted: Indent: Left: 0", Hanging: 0.49", Space After: 0 pt, Pattern: Clear

Formatted: Font color: Auto

Formatted: Line spacing: Multiple 1.15 li

FAO, 2018. Food and Agriculture Organization of the United Nations, *The State of World Fisheries and Aquaculture 2018—Meeting the Sustainable Development Goals*; FAO: Rome, Italy, 2018.

Folefack, G.B.L., Kengne, C.M., Dongmo, B.F. and Fomena, A., 2019. Prevalence and mean intensity of Myxobolus spp. Parasitizing Orechromis niloticus in Cameroon. *International Journal of Biology*, **11**(2), pp.35-45.

Food Business, 2025. Ethiopia's Grand Renaissance Dam to increase daily fish production – Ministry of Agriculture, https://www.foodbusinessafrica.com/

Fraulo, P., Morena, C., & Costa, A. (2014). Recovery of Anisakid larvae by means of chloro-peptic digestion and proposal of the method for the official control. *Acta Parasitologica*, **59**(4), 629–634.

Galaktionov, K, V.K, V., Irwin, S, W,S., B.W, B., and Saville, D, HD, H, 2006. One of the most complex life cycles among trematodes: a description of *Parvatrema margaritense* (Ching, 1982) n. comb. (Gymnophallidae) possessing parthenogenetic metacercariae. Parasitology **132**, 733–746

Gebreegziabher, H., Degefu, H. and Tsegay, A.K., 2020. Prevalence of internal helminth parasites of fish in Gilgel-Gibe River and three selected ponds in and around Jimma Town, Southwest Ethiopia. *Turkish Journal of Fisheries and Aquatic Sciences*, **20**(9), pp.693-699.

Getahun, A., 2002. The Nile Basin: Riverine Fish and Fisheries. Dept. of Biology Addis Ababa University, Ethiopia, pp: 19.

Grossart, H, P., Van den, S., Wyngaert, M. Kagami, C. Wurzbacher, M. Cunliffe, K., 2019. Rojas-Jimenez, Nat. Rev. Microbiol., 17, pp. 339-354, 10.1038/s41579-019-0175-8

Gulelat, Y., Yimer, E., Asmare, K. and Bekele, J., 2013. Study on parasitic helminths infecting three fish species from Koka reservoir, Ethiopia. *SINET: Ethiopian Journal of Science*, **36**(2), pp.73-80.

Hailu, M., Mitiku, M, A., 2021. The Effects of Fish Parasites in Freshwater Culture and Capture Fisheries and their Treatment Mechanisms, International Journal of Zoology and Animal Biology (IZAB),4(2), 1-10, https://doi.org/10.23880/izab-16000289.

Hasegawa, R, Otsuki Y, Uemura, Y., Furusawa, C., Naka, M., Kanno, Y., Koizumi, I., 2025. Positive feedback between parasite infection and poor host body condition reduces host survival in the wild, https://doi.org/10.1111/1365-2435.14742

Hoffman GL. 2019. Parasites of North American freshwater fishes. Cornell University Press.

Hundie, K., Berisa, L., Shitaw, T., Asmamaw, B. and Fekansa, T., 2021.Evaluating Household's Willingness to Pay for Catfish Species Conservation in Lake Hawassa, Sidama National Region State, Ethiopia. *International Journal of Oceanography and Aquaculture*, **5**(2):000204.

Imam, T.S., and Dewu, R.A., 2010. Survey of piscine ecto-and intestinal parasites of clarias species sold at Galadima Road Fish Market, Kano metropolis, Nigeria. *Bioscience Research Communications*, **22**(4), pp.209-214.

Formatted: Line spacing: Multiple 1.15 li

Janda, J, M., Abbott, S, L.,2010. The genus Aeromonas: taxonomy, pathogenicity, and infection. Clin Microbiol Rev, 23(1):35-73. doi: 10.1128/CMR.00039-09.

Kaba, T, Shurbe, M, Wondimu, A. 2024. Awareness of fish-borne zoonoses and prevalence of Contracaecum in Oreochromis niloticus and Lates niloticus collected from Lake Chamo, Arba Minch, Ethiopia. Zoonoses Public Health, 71(7):790-798. doi: 10.1111/zph.13145

Kaur, K., Reddy, S., Barathe, P., Oak, U., Shriram, V., Kharat, S, S., Govarthanan, M., Kumar, V., 2022. Microplastic-associated pathogens and antimicrobial resistance in environment, Chemosphere, 291, 10.1016/j.chemosphere.2021.133005

Kaygorodova, I, A., Sorokovikova, N, V. 2014. Mass leech infestation of sculpin fish in Lake Baikal, with clarification of disease-prone species and parasite taxonomy. Parasitol Int, ;63(6):754-7. https://doi.org/doi: 10.1016/j.parint.2014.06.009.

Kelsey, D, S., 1996. Enteric Nematodes of Lower Animals Transmitted to Humans: Zoonoses in Medical Microbiology, 4th edition.

Keyombe, J,L., Waithaka, E., Obegi, B., 2015. Length–weight relationship and condition factor of Clarias gariepinus in Lake Naivasha, Kenya, International Journal of Fisheries and Aquatic Studies; 2(6): 382-385,

Khan, S., Ahmed, S., Serajuddin, M., Saifullah, M, K., 2018. Variation in seasonal prevalence and intensity of progenetic metacercariae of Clinostomum complanatum infection in Trichogaster fasciatus fish, Beni-Suef University Journal of Basic and Applied Sciences, 7(3),310-316, https://doi.org/10.1016/j.bjbas.2018.03.006.

Khidr, A, A., Said, A, E., Abu Samak, O, A., Abu Sheref,S,E., 2012. The impacts of ecological factors on prevalence, mean intensity and seasonal changes of the monogenean gill parasite, Microcotyloides sp., infesting the Terapon puta fish inhabiting coastal region of Mediterranean Sea at Damietta region, The Journal of Basic & Applied Zoology, 65(2),109-115, https://doi.org/10.1016/j.jobaz.2012.06.003.

Kiprono, S.W., 2017. Fish parasites and fisheries productivity in relation to extreme flooding of Lake Baringo, Kenya. *Published master's Thesis. Kenyatta University*.

Klimpel, S., Thomas, K., Julian, M., Dorian, D. D., Regina, K., Judith, K.,2019. *Parasites of marine fish and cephalopods - A practical guide* (1st ed.). Springer.

Klinger, R.E. and Floyd, R.F., 1998. *Introduction to freshwater fish parasites*. Gainesville: University of Florida Cooperative Extension Service, Institute of Food and Agriculture Sciences, EDIS.

Kubitza, F., 2016. Common salt a useful tool in aquaculture, part 1, Simple ingredient offers multiple benefits to freshwater fish farmers, https://www.globalseafood.org/, accessed April 2025.

Labaude S, Rigaud T, Cézilly F, 2015. Host manipulation in the face of environmental changes: Ecological consequences, International Journal for Parasitology: *Parasites and Wildlife*, **4**(3), PP. 442-451, https://doi.org/10.1016/j.ijppaw.2015.08.001

Formatted: Line spacing: Multiple 1.15 li

LEDP, 2006. Lake Fishery development project, Proceeding of the national fishery seminar. Ziway number 1995. Lake Fishery development project working paper number 21AA ministry of agriculture.

Leung, T, L, F.,2022. Economies of parasite body size. Curr Biol. 32(12): R645-R649. https://doi.org/doi: 10.1016/j.cub.2022.01.059.

LFDP, 1996. Fisheries statistical bulletin No.3. Lake Fisheries Development Project Working Paper 20. Ministry of Agriculture, Addis Ababa, Ethiopia

Lima, D, P., Giacomini, H, C., Takemoto, R, M., Agostinho, A, A., Bini, L, M.,2012. Patterns of interactions of a large fish-parasite network in a tropical floodplain. J Anim Ecol.,81(4):905-13. https://doi.org/doi: 10.1111/j.1365-2656.2012.01967. x.

Ljubojevic, D., Novakov, N., Djordjevic, V., Radosavljevic, V., Pelic, M., & Cirkovic, M. (2015a). Potential parasitic hazards for humans in fish meat. *Procedia Food Science*, **5**, 172–175.

Ljubojevic, D., Radosavljevic, V., Puvaca, N., Balos, M. Z., Đorđevic, V., Jovanovic, R., & Cirkovic, M. (2015b). Interactive effects of dietary protein level and oil source on proximate composition and fatty acid composition in common carp (*Cyprinus carpio* L.). *Journal of Food Composition and Analysis*, **37**, 44–50.

Marcogliese, D. J. 2005. Parasites of the superorganism: Are they indicators of ecosystem health? International Journal of Parasitology 35:705-716.

Mattiucci, S., Paoletti, M., Colantoni, A., Carbone, A., Gaeta, R., Proietti, A., Frattaroli, S., Fazii, P., Bruschi, F., Nascetti, G.2017. Invasive anisakiasis by the parasite Anisakis pegreffii (Nematoda: Anisakidae): diagnosis by real-time PCR hydrolysis probe system and immunoblotting assay. BMC Infect Dis,;17(1):530. doi: 10.1186/s12879-017-2633-0.

Melak, T., and Getahun, A., 2012. Diversity and relative abundance of fishes in some temporary and perennial water bodies of the Baro basin, Gambella, Ethiopia, Ethiop. J. Biol. Sci. 11(2):193-206,

Menberu, Z., Mogesse, B., Reddythota, D.,2021. Evaluation of water quality and eutrophication status of Hawassa Lake based on different water quality indices, Applied Water Science, 11:61 https://doi.org/10.1007/s13201-021-01385-6

Mengesha T, A., 2015. Fish Species Diversity in Major River Basins of Ethiopia: A Review, World Journal of Fish and Marine Sciences 7 (5): 365-374, 2015, 10.5829/idosi.wjfms.2015.7.5.95195

Mgbemena, A., Arimoro, F., Omalu, I, and Keke, U., 2020. Prevalence of helminth parasites of Clarias gariepinus and Tilapia zillii in relation to age and sex in an afrotropical stream, Egyptian Journal of Aquatic Biology & Fisheries, https://www.ejabf.journals.ekb.eg/

Mitiku, M.A., 2017. Parasite Species Richness of Fish from Fishponds and Fingerling Sources in Central Ethiopia: It's Implication on Aquaculture Development (Doctoral dissertation, Doctoral dissertation, University of Natural Resources and Life Sciences).

Mitiku, M.A., Teklu, A. and Tadesse, Z., 2018. Prevalence of parasite of Nile Tilapia *(Oreochromis niloticus)* and African Big Barb *(Labeobarbus intermidus)* fish in Gigel-

Formatted: Line spacing: Multiple 1.15 li

Gibe-I Dam, Jimma Zone, Ethiopia, American-Eurasian Journal of Scientific Research **13**(2): 18-24, 2018.

Mocho, J, P., Blasco, J, R., Lundegaard, P, R., McKimm, R., Jenčič, V., Krogh, K, V.,2025. Methods of humane killing of laboratory fish: FELASA Working Group recommendations. Lab Anim, 59(5):599-613. doi: 10.1177/00236772251351095.

Mukama, S., 2008. Variations in abundance and diversity of parasites infecting catfish, *Clarias gariepinus (Burchell 1822) and tilapia Oreochromis urolepis (Norman 1922) in the Mindu dam: Morogoro municipality, Tanzania* (Doctoral dissertation, Unesco-IHE).

Nikolaev KE, Sukhotin AA and Galaktionov KV (2006). Infection patterns in White Sea blue mussels (*Mytilus edulis* L.) of different age and size with metacercariae of *Himasthla elongata* (Echinostomatidae) and *Cercaria parvicaudata* (Renicolidae). Diseases of Aquatic Organisms **71**, 51–58

NOAA Fisheries, 2020. Aquaculture Supports a Sustainable Earth, https://www.fisheries.noaa.gov/, accessed April 2025.

Obiero, K., Meulenbroek, P., Drexler, S., Dagne, A., Akoll, P., Odong,R., Kaunda-Arara, B., Waidbacher, H.,2019. The Contribution of Fish to Food and Nutrition Security in Eastern Africa: Emerging Trends and Future Outlooks, https://doi.org/10.3390/su11061636

Ogello EO, Mlingi FT, Nyonje BM *et al*. Can integrated livestock-fish culture be a solution to East Afircan's food insecurity? A Review.*African J Food Agric Nutr Dev.13 (4),* 8058-8078 (2013).

Okon, E,M., Oyesiji, A,A., Okeleye, E,D., Kanonuhwa, M., Khalifa, N,E., Eissa, E,H., Mathew, R,T., Eissa, M,E,H., Alqahtani, M,A., S, A.lnour, S,A., 2024.The Escalating threat of climate change-driven diseases in fish: Evidence from a global perspective - A literature review. Environ Res, 263(Pt 3):120184. doi: 10.1016/j.envres.2024.120184.

Outa, J.O., Dos Santos, Q.M., Avenant-Oldewage, A. *et al.* 2021. Parasite diversity of introduced fish *Lates niloticus*, *Oreochromis niloticus* and endemic *Haplochromis* spp. of Lake Victoria, Kenya. *Parasitol Res* **120**, 1583–1592. https://doi.org/10.1007/s00436-021-07095-x

Ozer, A., Oztruk, T., Oztruk, M, O.,2002. Prevalence and intensity of *Gyrodactylus arcuatus* Bychowsky, 1933 (Monogenea) infestations on the three-spined stickleback, *Gasterosteus aculeatus* L., 1758, Turk. J. Vet. Anim. Sci, pp. 807-812

Oztruk, M, O., Alutnel, F, N.,2006. Occurrence of Dactylogyrus infection linked to seasonal changes and host fish size on four cyprinid fishes, Acta Academiae, 52 (4), pp. 407-415

Paperna, I., 1996. Parasite infection and diseases of fish in Africa: An update. Technical Paper **31**. Rome, Central Institute of Freshwater Aquaculture. *Food and Agriculture Organization, United Nations*.

Paulose, T, Nkosi, Z, Z, Endriyas, M. 2022. Prevalence of hypertension and its associated factors in Hawassa city administration, Southern Ethiopia: Community based cross-sectional study. PLoS One. 1;17(3): e0264679. doi: 10.1371/journal.pone.0264679.

Formatted: Line spacing: Multiple 1.15 li

Formatted: Line spacing: Multiple 1.15 li

Petty, B, D., Francis-Floyd, R., YanongR., Yanong, R., P,2022. Parasitic Diseases of Fish, https://www.msdvetmanual.com/, accessed, April, April 2025

Pouder, D.B., Curtis, E.W. and Yanong, R.P.E., 2005. Common freshwater parasite pictorial guide edis. *Online). http://edis. ifas. ufl. edu*.

Poulin R, 2014. Parasite biodiversity revisited: frontiers and constraints, International Journal for Parasitology, 44(9),2014, Pages 581-589, ISSN 0020-7519, https://doi.org/10.1016/j.ijpara.2014.02.003

Poulin R, Presswell B. 2022. Is parasite taxonomy really in trouble? A quantitative analysis, International Journal for Parasitology, 52, 7, 2022, 469-474, https://doi.org/10.1016/j.ijpara.2022.03.001

Poulin, R., 2005. Variation in the intraspecific relationship between fish length and intensity of parasitic infection: biological and statistical causes, https://doi.org/10.1111/j.1095-8649.2000.tb02090.x

Power, C., Shirakashi, S., Bott, N.J., Nowak, B.F. 2025. Parasites in Aquaculture. In: Smit, N.J., Sures, B. (eds) Aquatic Parasitology: Ecological and Environmental Concepts and Implications of Marine and Freshwater Parasites. Springer, Cham. https://doi.org/10.1007/978-3-031-83903-0_23

Pradipta R. Rauta, Bismita Nayak, Surajit Das, 2012. Immune system and immune responses in fish and their role in comparative immunity study: A model for higher organisms, Immunology Letters,Volume 148, Issue 1,Pages 23-33, ISSN 0165-2478, https://doi.org/10.1016/j.imlet.2012.08.003

Ramos P, Parasites in fishery products - Laboratorial and educational strategies to control, Experimental Parasitology, 211,2020, 107865, ISSN 0014-4894, https://doi.org/10.1016/j.exppara.2020.107865.

Reed, C, C., Basson, L., Van As, L, L.,2002. Myxobolus species (Myxozoa), parasites of fishes in the Okavango River and Delta, Botswana, including descriptions of two new species. Folia Parasitol (Praha);49(2):81-8. doi: 10.14411/fp.2002.016.

Renner, E.D., Duggan, I.C., 2024. Season, size, and sex: factors influencing monogenean prevalence and intensity on *Gambusia affinis* in New Zealand. *Parasitol Res* **123**, 228. https://doi.org/10.1007/s00436-024-08241-x

Reshid, M., Adugna, M., Redda, Y.T., Awol, N. and Teklu, A., 2015. A study of Clinostomum (trematode) and Contracaecum (nematode) parasites affecting Oreochromis niloticus in Small Abaya Lake, Silite Zone, Ethiopia. *J. Aquac. Res. Dev*, **6**, p.316.

Roberts, R.J., 2001. *Fish Pathology*.3rd edn. Technical Director, Landa catches Ltd. Scotland, pp. 270–300.

Roy, P.E. and Young, E., 2002. Nematode (round worm) infection in fish. *Florida, IFAS*, **91**, pp.1-10.

Salem, M.A., Mahdy, O.A., El-Saied, M.A., Kamel, M, S., Mohammed, F, F., Ramadan, *R, M.*, 2025. Molecular and pathological insights into gene expression and oxidative stress

Formatted: Line spacing: Multiple 1.15 li

Formatted: Line spacing: Multiple 1.15 li

in *Clinostomum complanatum* and *Euclinostomum heterostomum*. *Sci Rep* **15**, 37586 (2025). https://doi.org/10.1038/s41598-025-16469-5

Senapin, S., Phiwsaiya, K., Laosinchai, P., Kowasupat, C., Ruenwongsa, P., Panijpan, B., 2014. Phylogenetic analysis of parasitic trematodes of the genus Euclinostomum found in Trichopsis and Betta fish. J Parasitol.;100(3):368-71. https://doi.org/doi: 10.1645/13-285.1.

Shafiq A., Abbas F., Hafeez-Ur-Rehman M., Khan B.N., Aihetasham A., Amin I., Hmidullah, Mothana R.A., Alharbi M.S., Khan I., et al. Parasite Diversity in a Freshwater Ecosystem. Microorganisms. 2023; 11:1940.

Shamsi S, Turner A, Wassens S. 2018. Description and genetic characterization of a new Contracaecum larval type (Nematoda: Anisakidae) from Australia. J Helminthol. 2018 Mar;92(2):216-222. doi: https://doi.org/10.1017/S0022149X17000360.

Shamsi, S., Barton, D.P. 2023. A critical review of anisakidosis cases occurring globally. *Parasitol Res* **122**, 1733–1745. https://doi.org/10.1007/s00436-023-07881-9

Simková, A., Jarkovský, J., Koubková, B., Barus, V, Prokes, M.,2005. Associations between fish reproductive cycle and the dynamics of metazoan parasite infection. Parasitol Res, 95(1):65-72. https://doi.org/doi: 10.1007/s00436-004-1261-y.

Skelton, P.H., Tweddle, D., Jackson, P.B.N. 1991. Cyprinids of Africa. In: Winfield, I.J., Nelson, J.S. (eds) Cyprinid Fishes. Fish & Fisheries Series, vol 3. Springer, Dordrecht, https://doi.org/10.1007/978-94-011-3092-9_7

Steckler N., Yanong, Yanong R, PR., 2024. Argulus (Fish Louse) Infections in Fish, https://edis.ifas.ufl.edu/

Sukhdeo, M.V. 2012. Where are the parasites in food webs? *Parasites Vectors* **5**, 239, https://doi.org/10.1186/1756-3305-5-239

Sures, B., Nachev, M.,2022. Effects of multiple stressors in fish: how parasites and contaminants interact. Parasitology;149(14):1822-1828. doi: 10.1017/S0031182022001172.

Tadesse, B., 2009. Prevalence and abundance of fish parasites in Bomosa Cage Systems and lakes Babogaya and Awassa, Ethiopia.

Tesfay, S., Teferi, M., Dejenie, T., Abay T., Hiluf, G., 2024. A survey of ecto and endoparasites of Nile tilapia, Oreochromis niloticus (Linnaeus, 1758) fingerlings in Midmar reservoir, Adwa, northern Ethiopia, Journal of Applied Animal Research, 52:1, 2310158, https://doi.org/DOI: 10.1080/09712119.2024.2310158

Tesfaye, A., Teklu, A., Bekelle, T., Tkue, T., Kebede, E., Gebretsadik, T. and Berhe, N., 2017.A survey on occurrence of internal and external fish parasites and causes of fish population reduction in Lake Hashenge, Tigray, Ethiopia. *Ethiopian Veterinary Journal*, **21**(2), pp.75-91.

Tesfaye, Z., Ferede, B., Pavanasam, N. and Workagegn, K.B., 2020. Prevalence of nematode parasite, Contracaecum, in Nile tilapia, African catfish and Barbus species in Lake Hawassa, Ethiopia.Aquaculture Research, **51**(10), pp.3993-3998.

Formatted: Line spacing: Multiple 1.15 li

Formatted: Line spacing: Multiple 1.15 li

Tesgera G, and Bayou K, 2021. Assessment of Current Practices, Major Challenges and Opportunities of the Fishery of Lake Hawassa, Southern Ethiopia, DOI: http://dx.doi.org/10.22192/ijarbs.2021.08.02.004, http://www.ijarbs.com/, 8(2), 1-9

Thakkar AN, 2023, Seasonal Variation in Prevalence of Nematode Parasites In Coilia Dussumieri From Uran, West Coast of India, Journal of Survey in Fisheries Sciences, 10(1) 4007-4009

Thrusfield, M.V., 2005. Veterinary Epidemiology 2nd ed. Black Well Science LTD, UK. pp. 233-250.

Tibebe, D., Tesfaye, Y. Kassa, Y., 2023.The impact of sewage effluents on water quality of Lake Hawassa, Ethiopia. *BMC Chemistry* **17**, 42. https://doi.org/10.1186/s13065-023-00954-x.

Tilahun, G., and Bihonegn, T., 2017.Study on Helminth Parasites in *Tilapia nilotica* from Lake Zeway, Ethiopia, Int. J. Adv. Res. Biol. Sci, **4**(10): 21-25.

Truter, M., Hadfield, K, A., Smit, N, J., 2023. Review of the metazoan parasites of the economically and ecologically important African sharptooth catfish Clarias gariepinus in Africa: Current status and novel records. Adv Parasitol.;119:65-222. doi: https://doi.org/10.1016/bs.apar.2022.11.001.

Trutera, M., Hadfielda K, A., and Smit N, J., 2022. Review of the metazoan parasites of the economically and ecologically important African sharptooth catfish Clarias gariepinus in Africa: Current status and novel records, 1-158, https://doi.org/10.1016/bs.apar.2022.11.001

Turner, W, C., Kamath, P, L., van Heerden, H., Huang, Y, H., Barandongo, Z, R., Bruce, S, A., Kausrud, K.,2021 The roles of environmental variation and parasite survival in virulence-transmission relationships. R Soc Open Sci. 2021 Jun 2;8(6):210088. doi: 10.1098/rsos.210088.

Valtonen, E.T., Marcogliese, D.J. and Julkunen, M., 2010. Vertebrate diets derived from trophically transmitted fish parasites in the Bothnian Bay. *Oecologia*, **162**(1), pp.139-152.

Formatted: Line spacing: Multiple 1.15 li

Vandi, P., Daniel, J. L.2023. Prevalence of Gastrointestinal Parasites and Condition Factor of Oreochromis niloticus (L.) in Kiri Reservoir, Shelleng, Adamawa State, Nigeria, Biological and Environmental Sciences Journal for the Tropics 20(3), https://dx.doi.org/10.4314/bestj.v20i3.7, 182-188.

Vidal-Martínez, V, M., Aguirre-Macedo, M, L., McLaughlin, J, P., Hechinger, R, F., Jaramillo, A, G., Shaw, J, C., James, A, K., Kuris, A, M., Lafferty, K, D.,2012. Digenean metacercariae of fishes from the lagoon flats of Palmyra Atoll, Eastern Indo-Pacific. J Helminthol, 86(4):493-509. doi: https://doi.org/10.1017/S0022149X11000526

Wakjira,M., Tolemariam,T., Kim, D, J., Kim, K, R., 2013. Aquaculture development in Ethiopia: review on potential and strategy, journal of agriculture, life and environmental sciences, 25(3), 20-24

WHO, 2020. Zoonoses, https://www.who.int/, assessed April 18, 2025.

Wubie, A., Teshome, K., Tesfaye, G., 2023. Potential fish yield and biology of some fish in Lake Hertale, Afar regional state, Ethiopia. Heliyon, 9(8):e18661. doi: 10.1016/j.heliyon. 2023.e18661.

Zekarias, T. and Yimer, E., 2007. Study on parasites of fish at Lake Awassa, Ethiopia. *Bulletin of Animal Health and Production in Africa*, **55**(3), pp.149-155.

Zhokhov, A.E., Mironovsky, A.N. and Miretskaya, D.A., 2007. Methods of the complete Parasitological dissection of fish. *Freshwater biology group, JERBE, Moscow–Addis-Ababa*, pp.1-12.

Ziarati, M., Zorriehzahra, M, J., Hassantabar, F., Mehrabi, Z., Dhawan, M., Sharun, K., Emran, T, B., Dhama, K., Chaicumpa, W., Shamsi, S.,2022. Zoonotic diseases of fish and their prevention and control. Vet Q.;42(1):95-118. https://doi.org/ doi: 10.1080/01652176.2022.2080298.

~~Abd-ELrahman, S, M., Gareh, A, Mohamed, H,I.H, I., Alrashdi, B,M.B, M., Dyab, A,K.A, K., El-Khadragy, M,F., Khairy Elbarbary, N., Fouad, A,M., El-Gohary, F,A., Elmahallawy, E,K.E, K., Mohamed, S,A.,2023. Prevalence and Morphological Investigation of Parasitic Infection in Freshwater Fish (Nile Tilapia) from Upper Egypt. Animals (Basel), 18;13(6), https://doi.org/doi: 10.3390/ani13061088~~

~~Abdel-Baki, A.A.S., Abdel-Haleem, H.M., Sakran, T., Zayed, E., Ibrahim, K.E. and Al-Quraishy, S., 2015. Two Myxobolus spp. infecting the kidney of Nile tilapia (Oreochromis niloticus) in the River Nile at Beni Suef governorate, Egypt, and the associated renal changes. *Parasitology research*, **114**(3), pp.1107-1112.~~

~~Ageng'o, F, A., Waruiru, R, M.,Wanja, D, W., Nyaga, P, N., Hamisi, M, M.,Ndegwa, J, M.,Ali, S, E., ChadagE., Chadag, M, V.,Mbuthia P, G.P, G.,2025. Parasites of Farmed and Wild Tilapine Fishes from Selected Farms and Lake Jipe in Taita Taveta County, Kenya, https://doi.org/10.1002/aff2.70042~~

~~Amlacher, E., 2005. Textbook of Fish Diseases. Narendra Publishing House, India, pp 207–292.~~

~~Ardelli, B. F. and Woo P. T. K., 1997. "Protective antibodies and anamnestic response in *Salvelinus fontinalis* to *Cryptobia salmositica* and innate resistance of *Salvelinus namaycush* to the hemoflagellate," *Journal of Parasitology*, vol. 83, no. 5, pp. 943–946, 1997.~~

~~Audicana, M, T., Kennedy, M, W.,2008. Anisakis simplex: from obscure infectious worm to inducer of immune hypersensitivity. Clin Microbiol Rev.;21(2):360-79, doi:10.1128/CMR.00012-07.~~

~~Bakke, T,A.,Harris, P,D.,Cable, J., 2002. Host specificity dynamics: observations on gyrodactylid monogeneans, Int. J. Parasitol., 32, pp. 281-308~~

~~Barber, I., Wright, H, A. 2005. Effects of Parasites on Fish Behaviour: Interactions with Host Physiology, Fish Physiology, Academic Press, 24,109-149, https://doi.org/10.1016/S1546-5098(05)24004-9~~

~~Bekele, J., Hussein, D., 2015. Prevalence of internal parasites of *Oreochromis niloticus* and *Clarius gariepinus*, journal of aquatic research and development,6(2), *http://dx.doi.org/10.4172/2155-9546.1000308*~~

Formatted: Line spacing: Multiple 1.15 li

Borji, H., Naghibi, A., Nasiri, M.R. and Ahmadi, A., 2012. Identification of Dactylogyrus spp. and other parasites of common carp in northeast of Iran. *Journal of Parasitic Diseases*, **36**(2), pp.234-238.

Boyd CE, McNevin AA, Davis RP. The contribution of fisheries and aquaculture to the global protein supply. Food Secur. 2022;14(3):805-827. https://doi.org/doi: 10.1007/s12571-021-01246-9.

Brian, J.I.,2023. Parasites in biodiversity conservation: Friend or foe? Trends Parasitol.; 39:618–621. https://doi.org/doi: 10.1016/j.pt.2023.05.005.

Brye K, 2023. Aquaculture: A Sustainable Solution for Fish Cultivation and Global Food Security, Journal of Agriculture, 6(4),1-3

Chai, J.Y.; Murrell, K.D.; Lymbery, A.J.,2005. Fish-borne parasitic zoonoses: Status and issues. *Parasitol. Int.* **2005**, *35*, 1233–1254

Chew, X, Z., Cobcroft,J.Cobcroft, J., Hutson, K, S.,2024. Fish ectoparasite detection, collection and curation. Adv Parasitol, 125:105-157. https://doi.org/doi: 10.1016/bs.apar.2024.05.001

Chimdo, A. Review on potential and challenges of aquaculture practice in Ethiopia. *Appl Water Sci* **12**, 218 (2022). https://doi.org/10.1007/s13201-022-01740-1

CNN travel, 2023. Ethiopian food: the 15 best dishes, https://edition.cnn.com/travel/article/ethiopian-food-best-dishes-africa/index.html#:~:text=Eating%20fish%20%E2%80%93%20asa%20%E2%80%93%20in%20Ethiopia,a%20piranha%20on%20your%20plate

Dadebo, E., 2000. Reproductive biology and feeding habits of the catfish Clarias gariepinus (Burchell) (Pisces: Clariidae) in Lake Awassa, Ethiopia. *SINET: Ethiopian Journal of Science*, **23**(2), pp.231-246.

Deng, G, T.2020. Assessment of Factors Affecting Fish Production and Marketing in Gambella Region, Ethiopia. ScientificWorldJournal. 16; 2020:5260693. doi: 10.1155/2020/5260693.

Desalegn, T. and Shitaw, T., 2021.Fishery Resources, Conservation Challenges and Management Strategies in Ethiopia. *Fisheries and Aquaculture Journal*, **12**(3), pp.1a-1a.

El-Shahawy, I.S., El-Seify, M.O., Metwally, A.M., Fwaz, M.M. 2017. Survey on endoparasitic fauna of some commercially important fishes of the River Nile, southern of Egypt (Egypt) Rev. De Med. Vet.; 168:126–134.

Folefack, G.B.L., Kengne, C.M., Dongmo, B.F. and Fomena, A., 2019. Prevalence and mean intensity of Myxobolus spp. Parasitizing Orechromis niloticus in Cameroon. *International Journal of Biology*, **11**(2), pp.35-45.

Food Business, 2025. Ethiopia's Grand Renaissance Dam to increase daily fish production – Ministry of Agriculture, https://www.foodbusinessafrica.com/

Fraulo, P., Morena, C., & Costa, A. (2014). Recovery of Anisakid larvae by means of chloro-peptic digestion and proposal of the method for the official control. *Acta Parasitologica*, **59**(4), 629–634.

Galaktionov, K, V.K, V., Irwin, S, W,S., B.W, B., and Saville, D, HD, H, 2006. One of the most complex life cycles among trematodes: a description of *Parvatrema margaritense* (Ching,

1982) n. comb. (Gymnophallidae) possessing parthenogenetic metacercariae. Parasitology **132**, 733–746

Gebreegziabher, H., Degefu, H. and Tsegay, A.K., 2020. Prevalence of internal helminth parasites of fish in Gilgel Gibe River and three selected ponds in and around Jimma Town, Southwest Ethiopia. *Turkish Journal of Fisheries and Aquatic Sciences*, **20**(9), pp.693-699.

Getahun, A., 2002. The Nile Basin: Riverine Fish and Fisheries. Dept. of Biology Addis Ababa University, Ethiopia, pp: 19.

Grossart, H, P., Van den, S., Wyngaert, M. Kagami, C. Wurzbacher, M. Cunliffe, K., 2019. Rojas-Jimenez, Nat. Rev. Microbiol., 17, pp. 339-354, 10.1038/s41579-019-0175-8

Gulelat, Y., Yimer, E., Asmare, K. and Bekele, J., 2013. Study on parasitic helminths infecting three fish species from Koka reservoir, Ethiopia. *SINET: Ethiopian Journal of Science*, **36**(2), pp.73-80.

Hailu, M., Mitiku, M, A., 2021. The Effects of Fish Parasites in Freshwater Culture and Capture Fisheries and their Treatment Mechanisms, International Journal of Zoology and Animal Biology (IZAB),4(2), 1-10, https://doi.org/10.23880/izab-16000289.

Hasegawa, R, Otsuki Y, Uemura, Y., Furusawa, C., Naka, M., Kanno, Y., Koizumi, I., 2025. Positive feedback between parasite infection and poor host body condition reduces host survival in the wild, https://doi.org/10.1111/1365-2435.14742

Hoffman GL. 2019. Parasites of North American freshwater fishes. Cornell University Press.

Hundie, K., Berisa, L., Shitaw, T., Asmamaw, B. and Fekansa, T., 2021.Evaluating Household's Willingness to Pay for Catfish Species Conservation in Lake Hawassa, Sidama National Region State, Ethiopia. *International Journal of Oceanography and Aquaculture*, **5**(2):000204.

Imam, T.S., and Dewu, R.A., 2010. Survey of piscine ecto-and intestinal parasites of clarias species sold at Galadima Road Fish Market, Kano metropolis, Nigeria. *Bioscience Research Communications*, **22**(4), pp.209-214.

Janda, J, M., Abbott, S, L.,2010. The genus Aeromonas: taxonomy, pathogenicity, and infection. Clin Microbiol Rev, 23(1):35-73. doi: 10.1128/CMR.00039-09.

Kaba, T, Shurbe, M, Wondimu, A. 2024. Awareness of fish-borne zoonoses and prevalence of Contracaecum in Oreochromis niloticus and Lates niloticus collected from Lake Chamo, Arba Minch, Ethiopia. Zoonoses Public Health, 71(7):790-798. doi: 10.1111/zph.13145

Kaur, K., Reddy, S., Barathe, P., Oak, U., Shriram, V., Kharat, S, S., Govarthanan, M., Kumar, V., 2022. Microplastic-associated pathogens and antimicrobial resistance in environment, Chemosphere, 291, 10.1016/j.chemosphere.2021.133005

Kaygorodova, I, A., Sorokovikova, N, V. 2014. Mass leech infestation of sculpin fish in Lake Baikal, with clarification of disease-prone species and parasite taxonomy. Parasitol Int, ;63(6):754-7. https://doi.org/doi: 10.1016/j.parint.2014.06.009.

Kelsey, D, S., 1996. Enteric Nematodes of Lower Animals Transmitted to Humans: Zoonoses in Medical Microbiology, 4th edition.

Khan, S., Ahmed, S., Serajuddin, M., Saifullah, M, K., 2018. Variation in seasonal prevalence and intensity of progenetic metacercariae of Clinostomum complanatum infection in Trichogaster fasciatus fish, Beni-Suef University Journal of Basic and Applied Sciences, 7(3),310-316, https://doi.org/10.1016/j.bjbas.2018.03.006.

Khidr, A, A., Said, A, E., Abu Samak, O, A., Abu Sheref,S,E., 2012. The impacts of ecological factors on prevalence, mean intensity and seasonal changes of the monogenean gill parasite, Microcotyloides sp., infesting the Terapon puta fish inhabiting coastal region of Mediterranean Sea at Damietta region, The Journal of Basic & Applied Zoology, 65(2),109-115, https://doi.org/10.1016/j.jobaz.2012.06.003.

Kiprono, S.W., 2017. Fish parasites and fisheries productivity in relation to extreme flooding of Lake Baringo, Kenya. *Published master's Thesis. Kenyatta University*.

Klimpel, S., Thomas, K., Julian, M., Dorian, D. D., Regina, K., Judith, K.,2019. *Parasites of marine fish and cephalopods - A practical guide* (1st ed.). Springer.

Klinger, R.E. and Floyd, R.F., 1998. *Introduction to freshwater fish parasites*. Gainesville: University of Florida Cooperative Extension Service, Institute of Food and Agriculture Sciences, EDIS.

Kubitza, F., 2016. Common salt a useful tool in aquaculture, part 1, Simple ingredient offers multiple benefits to freshwater fish farmers, https://www.globalseafood.org/, accessed April 2025.

Labaude S, Rigaud T, Cézilly F, 2015. Host manipulation in the face of environmental changes: Ecological consequences, International Journal for Parasitology: *Parasites and Wildlife*, **4**(3), PP. 442-451, https://doi.org/10.1016/j.ijppaw.2015.08.001

LEDP, 2006. Lake Fishery development project, Proceeding of the national fishery seminar. Ziway number 1995. Lake Fishery development project working paper number 21AA ministry of agriculture.

Leung, T, L, F.,2022. Economies of parasite body size. Curr Biol. 32(12): R645-R649. https://doi.org/doi: 10.1016/j.cub.2022.01.059.

LFDP, 1996. Fisheries statistical bulletin No.3. Lake Fisheries Development Project Working Paper 20. Ministry of Agriculture, Addis Ababa, Ethiopia

Lima, D, P., Giacomini, H, C., Takemoto, R, M., Agostinho, A, A., Bini, L, M.,2012. Patterns of interactions of a large fish-parasite network in a tropical floodplain. J Anim Ecol.,81(4):905-13. https://doi.org/doi: 10.1111/j.1365-2656.2012.01967. x.

Ljubojevic, D., Novakov, N., Djordjevic, V., Radosavljevic, V., Pelic, M., & Cirkovic, M. (2015a). Potential parasitic hazards for humans in fish meat. *Procedia Food Science*, **5**, 172–175.

Ljubojevic, D., Radosavljevic, V., Puvaca, N., Balos, M. Z., Đorđevic, V., Jovanovic, R., & Cirkovic, M. (2015b). Interactive effects of dietary protein level and oil source on proximate composition and fatty acid composition in common carp (*Cyprinus carpio* L.). *Journal of Food Composition and Analysis*, **37**, 44–50.

Marcogliese, D. J. 2005. Parasites of the superorganism: Are they indicators of ecosystem health? International Journal of Parasitology 35:705-716.

Melak, T., and Getahun, A., 2012. Diversity and relative abundance of fishes in some temporary and perennial water bodies of the Baro basin, Gambella, Ethiopia, Ethiop. J. Biol. Sci. 11(2):193-206,

Menberu, Z., Mogesse, B., Reddythota, D.,2021. Evaluation of water quality and eutrophication status of Hawassa Lake based on different water quality indices, Applied Water Science, 11:61 https://doi.org/10.1007/s13201-021-01385-6

Mengesha T, A., 2015. Fish Species Diversity in Major River Basins of Ethiopia: A Review, World Journal of Fish and Marine Sciences 7 (5): 365-374, 2015, 10.5829/idosi.wjfms.2015.7.5.95195

Mgbemena, A., Arimoro, F., Omalu, I, and Keke, U., 2020. Prevalence of helminth parasites of Clarias gariepinus and Tilapia zillii in relation to age and sex in an afrotropical stream, Egyptian Journal of Aquatic Biology & Fisheries, https://www.ejabf.journals.ekb.eg/

Mitiku, M.A., 2017. Parasite Species Richness of Fish from Fishponds and Fingerling Sources in Central Ethiopia: It's Implication on Aquaculture Development (Doctoral dissertation, Doctoral dissertation, University of Natural Resources and Life Sciences).

Mitiku, M.A., Teklu, A. and Tadesse, Z., 2018. Prevalence of parasite of Nile Tilapia *(Oreochromis niloticus)* and African Big Barb *(Labeobarbus intermidus)* fish in Gigel-Gibe I Dam, Jimma Zone, Ethiopia, American-Eurasian Journal of Scientific Research **13**(2): 18-24, 2018.

Mukama, S., 2008. Variations in abundance and diversity of parasites infecting catfish, *Clarias gariepinus (Burchell 1822) and tilapia Oreochromis urolepis (Norman 1922) in the Mindu dam: Morogoro municipality, Tanzania* (Doctoral dissertation, Unesco-IHE).

Nikolaev KE, Sukhotin AA and Galaktionov KV (2006). Infection patterns in White Sea blue mussels (*Mytilus edulis* L.) of different age and size with metacercariae of *Himasthla elongata* (Echinostomatidae) and *Cercaria parvicaudata* (Renicolidae). Diseases of Aquatic Organisms **71**, 51–58

NOAA Fisheries, 2020. Aquaculture Supports a Sustainable Earth, https://www.fisheries.noaa.gov/, accessed April 2025.

Ogello EO, Mlingi FT, Nyonje BM *et al*. Can integrated livestock-fish culture be a solution to East Afircan's food insecurity? A Review.*African J Food Agric Nutr Dev.13 (4)*, 8058-8078 (2013).

Okon, E,M., Oyesiji, A,A., Okeleye, E,D., Kanonuhwa, M., Khalifa, N,E., Eissa, E,H., Mathew, R,T., Eissa, M,E,H., Alqahtani, M,A., S, A.lnour, S,A., 2024.The Escalating threat of climate change-driven diseases in fish: Evidence from a global perspective - A literature review. Environ Res, 263(Pt 3):120184. doi: 10.1016/j.envres.2024.120184.

Outa, J.O., Dos Santos, Q.M., Avenant-Oldewage, A. *et al.* 2021. Parasite diversity of introduced fish *Lates niloticus, Oreochromis niloticus* and endemic *Haplochromis* spp. of Lake

Victoria, Kenya. *Parasitol Res* **120**, 1583-1592. https://doi.org/10.1007/s00436-021-07095-x

Ozer, A., Oztruk, T., Oztruk, M, O.,2002. Prevalence and intensity of *Gyrodactylus arcuatus* Bychowsky, 1933 (Monogenea) infestations on the three-spined stickleback, *Gasterosteus aculeatus* L., 1758, Turk. J. Vet. Anim. Sci, pp. 807-812

Oztruk, M, O., Alutnel, F, N.,2006. Occurrence of Dactylogyrus infection linked to seasonal changes and host fish size on four cyprinid fishes, Acta Academiae, 52 (4), pp. 407-415

Paperna, I., 1996. Parasite infection and diseases of fish in Africa: An update. Technical Paper **31**. Rome, Central Institute of Freshwater Aquaculture. *Food and Agriculture Organization, United Nations*.

Paulose, T, Nkosi, Z, Z, Endriyas, M. 2022. Prevalence of hypertension and its associated factors in Hawassa city administration, Southern Ethiopia: Community based cross-sectional study. PLoS One. 1;17(3): e0264679. doi: 10.1371/journal.pone.0264679.

Petty, B, D., Francis-Floyd, R., YanongR., Yanong, R., P,2022. Parasitic Diseases of Fish, https://www.msdvetmanual.com/, accessed, April, April 2025

Pouder, D.B., Curtis, E.W. and Yanong, R.P.E., 2005. Common freshwater parasite pictorial guide edis. *Online). http://edis. ifas. ufl. edu*.

Poulin R, 2014. Parasite biodiversity revisited: frontiers and constraints, International Journal for Parasitology, 44(9),2014, Pages 581-589, ISSN 0020-7519, https://doi.org/10.1016/j.ijpara.2014.02.003

Poulin R, Presswell B. 2022. Is parasite taxonomy really in trouble? A quantitative analysis, International Journal for Parasitology, 52, 7, 2022, 469-474, https://doi.org/10.1016/j.ijpara.2022.03.001

Poulin, R., 2005. Variation in the intraspecific relationship between fish length and intensity of parasitic infection: biological and statistical causes, https://doi.org/10.1111/j.1095-8649.2000.tb02090.x

Pradipta R. Rauta, Bismita Nayak, Surajit Das, 2012. Immune system and immune responses in fish and their role in comparative immunity study: A model for higher organisms, Immunology Letters,Volume 148, Issue 1,Pages 23-33, ISSN 0165-2478, https://doi.org/10.1016/j.imlet.2012.08.003

Ramos P, Parasites in fishery products – Laboratorial and educational strategies to control, Experimental Parasitology, 211,2020, 107865, ISSN 0014-4894, https://doi.org/10.1016/j.exppara.2020.107865.

Reed, C, C., Basson, L., Van As, L, L.,2002. Myxobolus species (Myxozoa), parasites of fishes in the Okavango River and Delta, Botswana, including descriptions of two new species. Folia Parasitol (Praha);49(2):81-8. doi: 10.14411/fp.2002.016.

Reshid, M., Adugna, M., Redda, Y.T., Awol, N. and Teklu, A., 2015. A study of Clinostomum (trematode) and Contracaecum (nematode) parasites affecting Oreochromis niloticus in Small Abaya Lake, Silite Zone, Ethiopia. *J. Aquac. Res. Dev*, **6**, p.316.

Roberts, R.J., 2001. *Fish Pathology*.3rd edn. Technical Director, Landa catches Ltd. Scotland, pp. 270–300.

Roy, P.E. and Young, E., 2002. Nematode (round worm) infection in fish. *Florida, IFAS*, **91**, pp.1-10.

Senapin, S., Phiwsaiya, K., Laosinchai, P., Kowasupat, C., Ruenwongsa, P., Panijpan, B., 2014. Phylogenetic analysis of parasitic trematodes of the genus Euclinostomum found in Trichopsis and Betta fish. J Parasitol.;100(3):368-71. https://doi.org/doi: 10.1645/13-285.1.

Shafiq A., Abbas F., Hafeez-Ur-Rehman M., Khan B.N., Aihetasham A., Amin I., Hmidullah, Mothana R.A., Alharbi M.S., Khan I., et al. Parasite Diversity in a Freshwater Ecosystem. Microorganisms. 2023; 11:1940.

Shamsi S, Turner A, Wassens S. 2018. Description and genetic characterization of a new Contracaecum larval type (Nematoda: Anisakidae) from Australia. J Helminthol. 2018 Mar;92(2):216-222. doi: https://doi.org/10.1017/S0022149X17000360.

Shamsi, S., Barton, D.P. 2023. A critical review of anisakidosis cases occurring globally. *Parasitol Res* **122**, 1733–1745. https://doi.org/10.1007/s00436-023-07881-9

Simková, A., Jarkovský, J., Koubková, B., Barus, V, Prokes, M.,2005. Associations between fish reproductive cycle and the dynamics of metazoan parasite infection. Parasitol Res, 95(1):65-72. https://doi.org/doi: 10.1007/s00436-004-1261-y.

Skelton, P.H., Tweddle, D., Jackson, P.B.N. 1991. Cyprinids of Africa. In: Winfield, I.J., Nelson, J.S. (eds) Cyprinid Fishes. Fish & Fisheries Series, vol 3. Springer, Dordrecht, https://doi.org/10.1007/978-94-011-3092-9_7

Steckler N., Yanong, Yanong R, PR., 2024. Argulus (Fish Louse) Infections in Fish, https://edis.ifas.ufl.edu/

Sures, B., Nachev, M.,2022. Effects of multiple stressors in fish: how parasites and contaminants interact. Parasitology;149(14):1822-1828. doi: 10.1017/S0031182022001172.

Tadesse, B., 2009. Prevalence and abundance of fish parasites in Bomosa Cage Systems and lakes Babogaya and Awassa, Ethiopia.

Tesgera G, and Bayou K, 2021. Assessment of Current Practices, Major Challenges and Opportunities of the Fishery of Lake Hawassa, Southern Ethiopia, DOI: http://dx.doi.org/10.22192/ijarbs.2021.08.02.004, http://www.ijarbs.com/, 8(2), 1-9

Tesfay, S., Teferi, M., Dejenie, T., Abay T., Hiluf, G., 2024. A survey of ecto and endoparasites of Nile tilapia, Oreochromis niloticus (Linnaeus, 1758) fingerlings in Midmar reservoir, Adwa, northern Ethiopia, Journal of Applied Animal Research, 52:1, 2310158, https://doi.org/DOI: 10.1080/09712119.2024.2310158

Tesfaye, A., Teklu, A., Bekelle, T., Tkue, T., Kebede, E., Gebretsadik, T. and Berhe, N., 2017.A survey on occurrence of internal and external fish parasites and causes of fish population reduction in Lake Hashenge, Tigray, Ethiopia. *Ethiopian Veterinary Journal*, **21**(2), pp.75-91.

Tesfaye, Z., Ferede, B., Pavanasam, N. and Workagegn, K.B., 2020. Prevalence of nematode parasite, Contracaecum, in Nile tilapia, African catfish and Barbus species in Lake Hawassa, Ethiopia.Aquaculture Research, **51**(10), pp.3993-3998.

Thakkar AN, 2023, Seasonal Variation in Prevalence of Nematode Parasites In Coilia Dussumieri From Uran, West Coast of India, Journal of Survey in Fisheries Sciences, 10(1) 4007-4009

Thrusfield, M.V., 2005. Veterinary Epidemiology 2nd ed. Black Well Science LTD, UK. pp. 233-250.

Tibebe, D., Tesfaye, Y. Kassa, Y., 2023.The impact of sewage effluents on water quality of Lake Hawassa, Ethiopia. *BMC Chemistry* **17**, 42. https://doi.org/10.1186/s13065-023-00954-x.

Tilahun, G., and Bihonegn, T., 2017.Study on Helminth Parasites in *Tilapia nilotica* from Lake Zeway, Ethiopia, Int. J. Adv. Res. Biol. Sci, **4**(10): 21-25.

Truter, M., Hadfield, K, A., Smit, N, J., 2023. Review of the metazoan parasites of the economically and ecologically important African sharptooth catfish Clarias gariepinus in Africa: Current status and novel records. Adv Parasitol.;119:65-222. doi: https://doi.org/10.1016/bs.apar.2022.11.001.

Trutera, M., Hadfielda K, A., and Smit N, J., 2022. Review of the metazoan parasites of the economically and ecologically important African sharptooth catfish Clarias gariepinus in Africa: Current status and novel records, 1-158, https://doi.org/10.1016/bs.apar.2022.11.001

Valtonen, E.T., Marcogliese, D.J. and Julkunen, M., 2010. Vertebrate diets derived from trophically transmitted fish parasites in the Bothnian Bay. *Oecologia*, **162**(1), pp.139-152.

Vandi, P., Daniel, J. L.2023. Prevalence of Gastrointestinal Parasites and Condition Factor of Oreochromis niloticus (L.) in Kiri Reservoir, Shelleng, Adamawa State, Nigeria, Biological and Environmental Sciences Journal for the Tropics 20(3), https://dx.doi.org/10.4314/bestj.v20i3.7, 182-188.

Vidal-Martínez, V, M., Aguirre-Macedo, M, L., McLaughlin, J, P., Hechinger, R, F., Jaramillo, A, G., Shaw, J, C., James, A, K., Kuris, A, M., Lafferty, K, D.,2012. Digenean metacercariae of fishes from the lagoon flats of Palmyra Atoll, Eastern Indo-Pacific. J Helminthol, 86(4):493-509. doi: https://doi.org/10.1017/S0022149X11000526

Wakjira,M., Tolemariam,T., Kim, D, J., Kim, K, R., 2013. Aquaculture development in Ethiopia: review on potential and strategy, journal of agriculture, life and environmental sciences, 25(3), 20-24

WHO, 2020. Zoonoses, https://www.who.int/, assessed April 18, 2025.

Zekarias, T. and Yimer, E., 2007. Study on parasites of fish at Lake Awassa, Ethiopia. *Bulletin of Animal Health and Production in Africa*, **55**(3), pp.149-155.

Zhokhov, A.E., Mironovsky, A.N. and Miretskaya, D.A., 2007. Methods of the complete Parasitological dissection of fish. *Freshwater biology group, JERBE, Moscow Addis-Ababa*, pp.1-12.

Ziarati, M., Zorriehzahra, M, J., Hassantabar, F., Mehrabi, Z., Dhawan, M., Sharun, K., Emran, T, B., Dhama, K., Chaicumpa, W., Shamsi, S.,2022. Zoonotic diseases of fish and their

prevention and control. Vet Q.;42(1):95-118. https://doi.org/ doi: 10.1080/01652176.2022.2080298.

Renner, E.D., Duggan, I.C., 2024. Season, size, and sex: factors influencing monogenean prevalence and intensity on *Gambusia affinis* in New Zealand. *Parasitol Res* **123**, 228. https://doi.org/10.1007/s00436-024-08241-x

Rebuttal letter (Response to reviewers and editors)

Manuscript Title: Prevalence of fish parasites from Lake Hawassa, Ethiopia

Manuscript ID: PONE-D-25-59453

Dear Editor and Reviewers,

We would like to sincerely thank the editor and reviewers for the careful evaluation of our manuscript and for the constructive comments and suggestions. These comments have greatly helped us improve the clarity, quality, and scientific rigor of the manuscript.

We have carefully revised the manuscript according to all comments. All changes are highlighted in the revised version, and detailed responses to each comment are provided below.

1. Please upload a Response to Reviewers letter which should include a point-by-point response to each of the points made by the Editor and / or Reviewers. (This should be uploaded as a 'Response to Reviewers' file type.) Please follow this link for more information: http://blogs.PLOS.org/everyone/2011/05/10/how-to-submit-your-revised-manuscript/
   Authors response: Thank you for bringing this to our attention. We have thoroughly checked the manuscript and supporting files to ensure that they meet PLOS ONE formatting and style requirements, including the appropriate file naming convections. All necessary corrections have been made in the revised submission.
2. To comply with PLOS One submissions requirements, in your Methods section, please provide additional information regarding the experiments involving animals and ensure you have included details on (1) methods of sacrifice, (2) methods of anesthesia and/or analgesia, and (3) efforts to alleviate suffering.

   Author response: Thank you for this important comment. We have revised the Methods section to include additional details regarding the animal-related procedures, including the methods of sacrifice, anesthesia and/or analgesia, and the measures taken to minimize animal suffering, in accordance with the PLOS ONE submission requirements.

3. "We note that your Data Availability Statement is currently as follows: All relevant data are within the manuscript and its Supporting Information files

   Please confirm at this time whether or not your submission contains all raw data required to replicate the results of your study. Authors must share the “minimal data set” for their submission. PLOS defines the minimal data set to consist of the data required to replicate all study findings reported in the article, as well as related metadata and methods (https://journals.plos.org/plosone/s/data-availability#loc-minimal-data-set-definition).

   For example, authors should submit the following data:
   - The values behind the means, standard deviations and other measures reported;
   - The values used to build graphs;
   - The points extracted from images for analysis.

Authors do not need to submit their entire data set if only a portion of the data was used in the reported study.

If your submission does not contain these data, please either upload them as Supporting Information files or deposit them to a stable, public repository and provide us with the relevant URLs, DOIs, or accession numbers. For a list of recommended repositories, please see https://journals.plos.org/plosone/s/recommended-repositories.

If there are ethical or legal restrictions on sharing a de-identified data set, please explain them in detail (e.g., data contain potentially sensitive information, data are owned by a third-party organization, etc.) and who has imposed them (e.g., an ethics committee). Please also provide contact information for a data access committee, ethics committee, or other institutional body to which data requests may be sent. If data are owned by a third party, please indicate how others may request data access."

Authors response: Dear editor in chief, Thank you for your comment. We confirm that the submission contains all the necessary raw data required to replicate the results of our study. The data used to create those tables are submitted as additional table S1.

4. We note that Figure 1 in your submission contain [map/satellite] images which may be copyrighted. All PLOS content is published under the Creative Commons Attribution License (CC BY 4.0), which means that the manuscript, images, and Supporting Information files will be freely available online, and any third party is permitted to access, download, copy, distribute, and use these materials in any way, even commercially, with proper attribution. For these reasons, we cannot publish previously copyrighted maps or satellite images created using proprietary data, such as Google software (Google Maps, Street View, and Earth). For more information, see our copyright guidelines: http://journals.plos.org/plosone/s/licenses-and-copyright.

   We require you to either (1) present written permission from the copyright holder to publish these figures specifically under the CC BY 4.0 license, or (2) remove the figures from your submission:

   1. You may seek permission from the original copyright holder of Figure 1 to publish the content specifically under the CC BY 4.0 license.

   We recommend that you contact the original copyright holder with the Content Permission Form (http://journals.plos.org/plosone/s/file?id=7c09/content-permission-form.pdf) and the following text:

   “I request permission for the open-access journal PLOS ONE to publish XXX under the Creative Commons Attribution License (CCAL) CC BY 4.0 (http://creativecommons.org/licenses/by/4.0/). Please be aware that this license allows unrestricted use and distribution, even commercially, by third parties. Please reply and provide explicit written permission to publish XXX under a CC BY license and complete the attached form.”

   Please upload the completed Content Permission Form or other proof of granted permissions as an "Other" file with your submission.

   In the figure caption of the copyrighted figure, please include the following text:

“Reprinted from [ref] under a CC BY license, with permission from [name of publisher], original copyright [original copyright year].”

2. If you are unable to obtain permission from the original copyright holder to publish these figures under the CC BY 4.0 license or if the copyright holder’s requirements are incompatible with the CC BY 4.0 license, please either i) remove the figure or ii) supply a replacement figure that complies with the CC BY 4.0 license. Please check copyright information on all replacement figures and update the figure caption with source information. If applicable, please specify in the figure caption text when a figure is similar but not identical to the original image and is therefore for illustrative purposes only.

The following resources for replacing copyrighted map figures may be helpful:

USGS National Map Viewer (public domain): http://viewer.nationalmap.gov/viewer/

The Gateway to Astronaut Photography of Earth (public domain): http://eol.jsc.nasa.gov/sseop/clickmap/

Maps at the CIA (public domain): https://www.cia.gov/library/publications/the-world-factbook/index.html and https://www.cia.gov/library/publications/cia-maps-publications/index.html

NASA Earth Observatory (public domain): http://earthobservatory.nasa.gov/

Landsat: http://landsat.visibleearth.nasa.gov/

USGS EROS (Earth Resources Observatory and Science (EROS) Center) (public domain): http://eros.usgs.gov/#

Natural Earth (public domain): http://www.naturalearthdata.com/

Authors response: Thank you for bringing this to our attention. We have thoroughly checked the manuscript and supporting files to ensure that they meet PLOS ONE formatting and style requirements, including the appropriate file naming convections. All necessary corrections have been made in the revised submission.

5. We note that Figure 2 in your submission contain copyrighted images. All PLOS content is published under the Creative Commons Attribution License (CC BY 4.0), which means that the manuscript, images, and Supporting Information files will be freely available online, and any third party is permitted to access, download, copy, distribute, and use these materials in any way, even commercially, with proper attribution. For more information, see our copyright guidelines: http://journals.plos.org/plosone/s/licenses-and-copyright.

We require you to either (1) present written permission from the copyright holder to publish these figures specifically under the CC BY 4.0 license, or (2) remove the figures from your submission:

1. You may seek permission from the original copyright holder of Figure 2 to publish

the content specifically under the CC BY 4.0 license.

We recommend that you contact the original copyright holder with the Content Permission Form (http://journals.plos.org/plosone/s/file?id=7c09/content-permission-form.pdf) and the following text:

“I request permission for the open-access journal PLOS ONE to publish XXX under the Creative Commons Attribution License (CCAL) CC BY 4.0 (http://creativecommons.org/licenses/by/4.0/). Please be aware that this license allows unrestricted use and distribution, even commercially, by third parties. Please reply and provide explicit written permission to publish XXX under a CC BY license and complete the attached form.”

Please upload the completed Content Permission Form or other proof of granted permissions as an "Other" file with your submission.

In the figure caption of the copyrighted figure, please include the following text: “Reprinted from [ref] under a CC BY license, with permission from [name of publisher], original copyright [original copyright year].”

2. If you are unable to obtain permission from the original copyright holder to publish these figures under the CC BY 4.0 license or if the copyright holder’s requirements are incompatible with the CC BY 4.0 license, please either i) remove the figure or ii) supply a replacement figure that complies with the CC BY 4.0 license. Please check copyright information on all replacement figures and update the figure caption with source information. If applicable, please specify in the figure caption text when a figure is similar but not identical to the original image and is therefore for illustrative purposes only.

Author response: Dear editor in chief, thank you for bringing this issue to our attention. We understand the copyright restrictions related to maps and satellite images created using proprietary data. Although we initially requested permission from the original copyright holder, we have decided to remove the map from Figure 1, as it only represented a very narrow area. Instead, we have revised the manuscript to describe the agroecological characteristics of the Lake Hawassa area in the text.

If the reviewer comments include a recommendation to cite specific previously published works, please review and evaluate these publications to determine whether they are relevant and should be cited. There is no requirement to cite these works unless the editor has indicated otherwise.

Authors response: Thank you for this comment. We have carefully evaluated the publications suggested by the reviewers and have included them as references where they were relevant to the study.

From the earlier editorial and reviewers' comments

1. Please ensure that your manuscript meets PLOS ONE's style requirements, including those for file naming. The PLOS ONE style templates can be found at

https://journals.plos.org/plosone/s/file?id=wjVg/PLOSOne_formatting_sample_main_body.pdf and

https://journals.plos.org/plosone/s/file?id=ba62/PLOSOne_formatting_sample_title_authors_affiliations.pdf

Authors response: Thank you for bringing this to our attention. We have thoroughly checked the manuscript and supporting files to ensure that they meet PLOS ONE formatting and style requirements, including the appropriate file naming convections. All necessary corrections have been made in the revised submission.

2. In your Methods section, please provide additional information regarding the permits you obtained for the work. Please ensure you have included the full name of the authority that approved the field site access and, if no permits were required, a brief statement explaining why.

Author response: We have communicated city municipality via letter from Wollega University dated 24/04/2023 immediately after obtaining ethical clearance. The Hawassa city administration accepts our letter and permit as for the research to be conducted.

3. To comply with PLOS One submissions requirements, in your Methods section, please provide additional information regarding the experiments involving animals and ensure you have included details on (1) methods of sacrifice, (2) methods of anesthesia and/or analgesia, and (3) efforts to alleviate suffering.

Author response: All the fishes we have used have been captured for commercial reason. We immediately captured fresh fishes with cooperation from fishery person who are professional in capturing fishes and have fish cages. No anaesthesia was used but we process the sample after they stop breathing which is in minutes after they are captured. No suffering exists during sample processing.

4. When completing the data availability statement of the submission form, you indicated that you would make your data available on acceptance. We strongly recommend all authors decide on a data sharing plan before acceptance, as the process can be lengthy and hold up publication timelines. Please note that, though access restrictions are acceptable now, your entire data will need to be made freely accessible if your manuscript is accepted for publication. This policy applies to all data except where public deposition would breach compliance with the protocol approved by your research ethics board. If you are unable to adhere to our open data policy,

please kindly revise your statement to explain your reasoning and we will seek the editor's input on an exemption. Please be assured that, once you have provided your new statement, the assessment of your exemption will not hold up the peer review process.

Author response: I agree with journal data availability policy that our data will fully be available without restriction.

5. We note that Figure 1 in your submission contain [map/satellite] images which may be copyrighted. All PLOS content is published under the Creative Commons Attribution License (CC BY 4.0), which means that the manuscript, images, and Supporting Information files will be freely available online, and any third party is permitted to access, download, copy, distribute, and use these materials in any way, even commercially, with proper attribution. For these reasons, we cannot publish previously copyrighted maps or satellite images created using proprietary data, such as Google software (Google Maps, Street View, and Earth). For more information, see our copyright guidelines: http://journals.plos.org/plosone/s/licenses-and-copyright.

We require you to either (1) present written permission from the copyright holder to publish these figures specifically under the CC BY 4.0 license, or (2) remove the figures from your submission:

1. You may seek permission from the original copyright holder of Figure 1 to publish the content specifically under the CC BY 4.0 license.

We recommend that you contact the original copyright holder with the Content Permission Form (http://journals.plos.org/plosone/s/file?id=7c09/content-permission-form.pdf) and the following text:

“I request permission for the open-access journal PLOS ONE to publish XXX under the Creative Commons Attribution License (CCAL) CC BY 4.0 (http://creativecommons.org/licenses/by/4.0/). Please be aware that this license allows unrestricted use and distribution, even commercially, by third parties. Please reply and provide explicit written permission to publish XXX under a CC BY license and complete the attached form.”

Please upload the completed Content Permission Form or other proof of granted permissions as an "Other" file with your submission.

Author response: Dear editor in chief, thank you for bringing this issue to our attention. We understand the copyright restrictions related to maps and satellite images created using proprietary data. Although we initially requested permission from the original copyright holder, we have decided to remove the map from Figure 1, as it only represented a very narrow area. Instead, we have revised the manuscript to describe the agroecological characteristics of the Lake Hawassa area in the text.

In the figure caption of the copyrighted figure, please include the following text: “Reprinted from [ref] under a CC BY license, with permission from [name of publisher], original copyright [original copyright year].”

2. If you are unable to obtain permission from the original copyright holder to publish these figures under the CC BY 4.0 license or if the copyright holder’s requirements are incompatible with the CC BY 4.0 license, please either i) remove the figure or ii) supply a replacement figure that complies with the CC BY 4.0 license. Please check copyright information on all replacement figures and update the figure caption with source information. If applicable, please specify in the figure caption text when a figure is similar but not identical to the original image and is therefore for illustrative purposes only.

The following resources for replacing copyrighted map figures may be helpful:

USGS National Map Viewer (public domain): http://viewer.nationalmap.gov/viewer/

The Gateway to Astronaut Photography of Earth (public domain): http://eol.jsc.nasa.gov/sseop/clickmap/

Maps at the CIA (public domain): https://www.cia.gov/library/publications/the-world-factbook/index.html and https://www.cia.gov/library/publications/cia-maps-publications/index.html

NASA Earth Observatory (public domain): http://earthobservatory.nasa.gov/

Landsat: http://landsat.visibleearth.nasa.gov/

USGS EROS (Earth Resources Observatory and Science (EROS) Center) (public domain): http://eros.usgs.gov/#

Natural Earth (public domain): http://www.naturalearthdata.com/

Author response: Dear editor in chief, thank you for bringing this issue to our attention. We understand the copyright restrictions related to maps and satellite images created using proprietary data. Although we initially requested permission from the original copyright holder, we have decided to remove the map from Figure 1, as it only represented a very narrow area. Instead, we have revised the manuscript to describe the agroecological characteristics of the Lake Hawassa area in the text.

6. We note that Figure 2 in your submission contain copyrighted images. All PLOS content is published under the Creative Commons Attribution License (CC BY 4.0), which means that the manuscript, images, and Supporting Information files will be freely available online, and any third party is permitted to access, download, copy, distribute, and use these materials in any way, even commercially, with proper

attribution. For more information, see our copyright guidelines: http://journals.plos.org/plosone/s/licenses-and-copyright.

We require you to either (1) present written permission from the copyright holder to publish these figures specifically under the CC BY 4.0 license, or (2) remove the figures from your submission:

1. You may seek permission from the original copyright holder of Figure 2 to publish the content specifically under the CC BY 4.0 license.

We recommend that you contact the original copyright holder with the Content Permission Form (http://journals.plos.org/plosone/s/file?id=7c09/content-permission-form.pdf) and the following text:

"I request permission for the open-access journal PLOS ONE to publish XXX under the Creative Commons Attribution License (CCAL) CC BY 4.0 (http://creativecommons.org/licenses/by/4.0/). Please be aware that this license allows unrestricted use and distribution, even commercially, by third parties. Please reply and provide explicit written permission to publish XXX under a CC BY license and complete the attached form."

Please upload the completed Content Permission Form or other proof of granted permissions as an "Other" file with your submission.

In the figure caption of the copyrighted figure, please include the following text: "Reprinted from [ref] under a CC BY license, with permission from [name of publisher], original copyright [original copyright year]."

Author response: Dear Editor-in-Chief, thank you for your comment regarding Figure 2 in the earlier submission (now labelled as Figure 1 after revision). We would like to clarify that all images included in this manuscript including Figure 1 were captured by the research team during the study. Therefore, no third-party permission is required for their publication or use for scientific purposes.

2. If you are unable to obtain permission from the original copyright holder to publish these figures under the CC BY 4.0 license or if the copyright holder's requirements are incompatible with the CC BY 4.0 license, please either i) remove the figure or ii) supply a replacement figure that complies with the CC BY 4.0 license. Please check copyright information on all replacement figures and update the figure caption with source information. If applicable, please specify in the figure caption text when a figure is similar but not identical to the original image and is therefore for illustrative purposes only.

If the reviewer comments include a recommendation to cite specific previously published works, please review and evaluate these publications to determine whether

they are relevant and should be cited. There is no requirement to cite these works unless the editor has indicated otherwise.

[Note: HTML markup is below. Please do not edit.]

Reviewers' comments: Reviewer's Responses to Questions

Comments to the Author

1. Is the manuscript technically sound, and do the data support the conclusions?

The manuscript must describe a technically sound piece of scientific research with data that supports the conclusions. Experiments must have been conducted rigorously, with appropriate controls, replication, and sample sizes. The conclusions must be drawn appropriately based on the data presented.

Reviewer #1: Yes

Author response: Thank you, we appreciate your guidance and have carefully considered these aspects in revising our manuscript.

Reviewer #2: Yes

Author response: Thank you, we appreciate your guidance and have carefully considered these aspects in revising our manuscript.

Reviewer #3: No

Author response: we have improved the manuscript as per the reviewers and editor in chief comments.

Reviewer #4: Partly

Author response: Thank you, we have also thoroughly improved the manuscript

---

2. Has the statistical analysis been performed appropriately and rigorously?

Reviewer #1: Yes

Author response: Thank you

Reviewer #2: Yes

Author response: Thank you

Reviewer #3: No

Author response: we have improved the methodological and statical section of the manuscript

Reviewer #4: Yes

Author response: Thank you

---

3. Have the authors made all data underlying the findings in their manuscript fully available?

The PLOS Data policy requires authors to make all data underlying the findings described in their manuscript fully available without restriction, with rare exception (please refer to the Data Availability Statement in the manuscript PDF file). The data should be provided as part of the manuscript or its supporting information or deposited to a public repository. For example, in addition to summary statistics, the data points behind means, medians and variance measures should be available. If there are restrictions on publicly sharing data—e.g. participant privacy or use of data from a third party—those must be specified.

Reviewer #1: Yes

Author response: Thank you, thank you, we have also thoroughly improved the manuscript

Reviewer #2: Yes

Author response: Thank you, we have also thoroughly improved the manuscript

Reviewer #3: No

Author response: We declare that all data are fully available without restriction

Reviewer #4: Yes

Author response: Thank you, we have also thoroughly improved the manuscript

---

4. Is the manuscript presented in an intelligible fashion and written in standard English?

PLOS ONE does not copyedit accepted manuscripts, so the language in submitted articles must be clear, correct, and unambiguous. Any typographical or grammatical errors should be corrected at revision, so please note any specific errors here.

Reviewer #1: Yes

Reviewer #2: Yes

Reviewer #3: No

Author response: We have thoroughly edited the manuscript during our current revision.

Reviewer #4: No

Author response: We have thoroughly edited the manuscript during our current revision.

---

5. Review Comments to the Author

Please use the space provided to explain your answers to the questions above. You may also include additional comments for the author, including concerns about dual publication, research ethics, or publication ethics. (Please upload your review as an attachment if it exceeds 20,000 characters)

Reviewer #1: Dear Author
I sincerely appreciate the opportunity to review this article, which is titled: Prevalence of fish parasites from Lake Hawassa, Ethiopia
Manuscript Number: PONE-D-25-59453
That is a good, clear title, but it could be significantly improved by adding more specificity about the hosts and the study's key finding, which increases its impact.

Author response: Accordingly, we have significantly improved the manuscript by carefully addressing the suggestions and comments raised by both the reviewers.

This is a great starting point for a manuscript, but there are several points that require clarification or correction to meet high scientific standards.
Here is a point-by-point scientific review:
The manuscript is efficient, but the lack of citations for key statistics and claims is a serious issue that must be addressed before submission.

Author response: we have addressed those suggestions provided by respected reviewer.

You also need to correct the simple grammar and spelling errors noted in Ms.

Author response: ok., thanks

Strengthening the opening statements, maintaining scientific accuracy, and attending to the requirement for citations are the main objectives of this review.

Kindly find the attached file

Author response: we have addressed those suggestions provided by respected reviewer.

Reviewer #2: This manuscript presents a cross-sectional parasitological survey of three commercially important fish species from Lake Hawassa, Ethiopia. While the topic is relevant and the sample size is adequate, the manuscript requires substantial revision before publication.

Author response: we have substantially reviewed, used new and existing references to improve the manuscript

The study provides valuable baseline data on fish parasite prevalence but suffers from some gaps, incomplete taxonomic identification, and inadequate contextualization of findings.

Author response: we have improved the taxonomic identification and hence conceptualisation of the key findings in the result and discussion.

1- Taxonomic Identification Limitations

The authors acknowledge limiting identification to genus level, citing that "fish harbors mostly larval stages" (lines 242-243). However, this is scientifically problematic:

-Species-level identification is feasible: Modern molecular techniques (PCR, DNA barcoding) can identify larval stages to species level. The authors provide no justification for not attempting molecular identification.

Author response: We have indicated the limitation of the current study in the limitation section. PCR and DNA barcoding needs high-tech molecular diagnostic machines which is limited in my institution. This work is purely for academic purpose

-Public health implications: For zoonotic parasites like Contracaecum spp., species-level identification is crucial as different species have different zoonotic potential.

-Ecological interpretation: Genus-level identification limits ecological and epidemiological interpretations.

Question 1: Why were molecular identification methods not employed, especially given the zoonotic importance of several parasites identified? Were resources unavailable or was this a deliberate methodological choice?
Author response: we are unable to extract DNA and perform molecular identification due to limitation of resources and test kits. This research is conducted with the university lecturers without any local or international fund. Here we have performed base line data on epidemiology and assure you to perform molecular test in future projects with collaborated effort
2- water quality parameters:
The manuscript mentions water quality issues (lines 362-377, 488-491, 708-710) but provides NO water quality data. The authors acknowledge environmental stressors but fail to measure them. Multiple references cited (Memberu et al., 2021; Tibebe et al., 2023) demonstrate poor water quality in Lake Hawassa

Author response: we have conducted some questionnaire survey including local leaders, environmental protection officers affiliated to Hawassa Municipal abattoir who have witnessed the availability of water contamination.
Question 2: Were any water quality parameters measured during this study? If not, why was this critical environmental component excluded from a parasitological survey?
Author response: water quality analysis is bit expensive project in Ethiopian context. But the research team clearly observed the contamination of water by offals from the fish, car washing to the lake, discarding of plastics to the lake and the high availability of free roaming birds which could add stress to the fish and create good ground for fish parasites. We can provide some pictures captured by research team. We have questionnaire survey report that indicates human practice around the lake.
3-Temporal Sampling Design
"October 2023 to July 2024" and "conducted in all for over 10 months" (lines 169-173)
-Were fish collected continuously or at intervals?
-What was the sampling frequency?
-Were seasonal variations accounted for?
-Multiple citations (Bakke et al., 2002; Ozer et al., 2004; Khidr et al., 2012; Khan et al., 2018) emphasize seasonal effects on parasite prevalence
-Provide a clear sampling timeline and sample sizes per collection period. Analyze seasonal effects if data permit.

Authors response: we have randomly selected the fish of our target until we reach the desired sample size(n=265). The period mentioned here is including the time spent in preparation to sample processing. During data collection we did not actually consider seasonal effects.

4-Sample Selection
"Systematic random sampling" (line 171): This term is contradictory. Was sampling:

-Systematic? or Random (using random number generators)? or Stratified by species?
-Source of fish unclear: Were fish collected:
=Directly from the lake (by researchers)?
=From fishermen (introducing selectivity bias)?
=From fish markets (introducing preservation/handling artifacts)?
Authors response: the sample was directly collected by the researchers at the lake Hawassa. Systematic random sampling means to indicate that we are randomly selecting the fishes at specific interval; from every 10 fishes at random but systematically to decide which number will be selected during the research day. A
-Sampling locations: No description of geographic sampling within Lake Hawassa
Authors response: we have provided the geographic description of Lake Hawassa and Hawasa city in the updated manuscript.
5- some parasitological methods lack details as magnification power, fixation details...etc
Authors response:
6-Results:
-Table 1 vs. Text discrepancy:
-Table 1 reports 788 parasites from O. niloticus (line 277)
-Text reports 787 parasites (lines 30-31, abstract)
Which is correct?
Authors response: The typo error in abstract is corrected. The correct parasite number from *O. niloticus* is 788.
7-Fish morphometrics:
-No mean ± SD for fish weights and lengths per species
-Weight and length ranges provided (lines 199-206) but no central tendencies
-Impossible to assess if samples represent typical population demographics
Authors response:
8- Parasite intensity data:
-Table 1 shows "Number of parasites from each fish: 6-9, 4-6, 5-10"
-These ranges are unclear: Are these per infected fish? Per parasite species?

Authors response: this number indicates the number of parasites collected from infected fish.

Please also refer the formula for;

$$\text{Mean intensity}=\frac{\text{Sum of individuals of parasite species in a host sample}}{\text{Total no.of infected individuals of the host in the sample}}$$

-Mean intensities (6.79, 7.92, 8.59) don't align logically with these ranges
Authors response: mean intensity is calculated as the number of parasites collected/the number of fish affected. For example, 116 fishes are infected from O. niloticus, and 788 different parasites are collected. By dividing 788 to 116 we get 6.79. for other fish species we have computed similarly (*El-Shahawy et al., 2017).*

9- Discussion:
-Comparison-heavy, interpretation-light: The discussion is dominated by comparing prevalence values to other studies (lines 329-343) without explaining WHY differences exist
Author response: We have explained the difference in prevalence in relation to parasite ecological, host related factors, and fishing composition (Turner et al., 2021).

Ecological context missing:
-No discussion of Lake Hawassa's specific ecological characteristics
-How do lake depth, temperature, presence of intermediate hosts affect parasite communities?
-What snail species are present (necessary for digenean life cycles)?

Authors response: the ecological features of the lake was updated in the manuscript. ‘Lake Hawassa have temperature range of 21.3^{0}C. The surface are ranges from 8500-9000 hectare and the maximum central depth of 18-22m with shoreline ranging from 50-65km in length. While lake Hawassa is the smallest of the rift valley lake, it is rich in phytoplankton and zooplankton that supports the six population of the fish resources’
Zoonotic risk inadequately addressed:

-Contracaecum spp. are identified as zoonotic (lines 421-427) but no risk assessment is provided
-What are fish consumption practices in the region?
-Are fish cooked adequately?
-No connection to the cultural practices mentioned (lines 84-89)
Authors response: there is a tradition of consuming raw or undercooked fresh fish which is the risk factor for anisakiasis like infection.
Typographical errors:
-Line 16: "pastes" → "parasites"
-Line 38-39: "mean intensity...is found to be" → awkward phrasing; use "was"
-Line 64: "Some of the lakes" → "Several lakes" (more formal)
-Line 73: "accounts 90%" → "accounts for 90%"

enhance your discussion with following:

Author response: the discussion was enhanced by using the suggested and other resources

Khormi, M.A., Emeish, W.F.A., Alghamdi, A.A.A., Fawaz, M,M.,Al-Amgad, Z., Abdelsalam, M., Al-Megrin, W, A.,Bakry, K, A., 2025. Pathophysiological impact of *Euclinostomum heterostomum* infection in Nile tilapia (*Oreochromis niloticus*): a multidisciplinary investigation revealing immune-oxidative stress interactions. *Aquacult Int* **33**, 524 (2025). https://doi.org/10.1007/s10499-025-02205-3
Atwah, M.A., Khormi, M.A., Abdelbaky, A.A., Abd El Megeed, O, H., Aborasain, A, M., Salem, M,A.,Soliman, M.A., Khalefa, H, S., Abd-El Hafeez, H, H., Abdelsalam, M.,

2025. Multifaceted analysis of cymothoids parasitism, environmental stressors and heavy metal bioaccumulation dynamics in mediterranean farmed fish in Egypt. *Aquacult Int* **33**, 354, https://doi.org/10.1007/s10499-025-02037-1
Attia, M.M., Abdelsalam, M., Mohamed, H.I., Shadidizaji, A., Soliman, A., W., Warda, W., 2025. *Punica granatum* extract demonstrates antiparasitic effects against *Caligus clemensi* through in silico and in vitro studies. *Sci Rep* **15**, 35005, https://doi.org/10.1038/s41598-025-19529-y

Reviewer #3: The authors have important information. However, the way the article is presented does not meet minimum quality standards. I include many observations in the document. The authors should review the document thoroughly.

Reviewer #4: Thank you for the opportunity to review this manuscript. The topic is important and highly relevant, particularly given the need for more research from regions such as Ethiopia, where local data can substantially improve global understanding. The effort invested in this study is evident, and with further refinement the manuscript has the potential to make a valuable contribution.

Authors response:
Below I outline several areas where improvements would strengthen the clarity, scientific quality, and overall impact of the work. The manuscript would benefit from substantial improvement in both English expression and scientific writing. At present, the narrative is difficult to follow, and key messages become obscured by issues of organisation and clarity.
A more structured, focused, and concise writing style is strongly recommended.
Lines 13–14:
Please clarify whether you are referring to 200 species of marine fish, freshwater fish, or both. This distinction is important for understanding the ecological context of the study and the relevance of the lake system.

Authors response: Most of the fish species in Ethiopia are freshwater fish but their also marine fishes. It is updated in the manuscript
Lines 12–17: It would strengthen the introduction to briefly outline the significance of the Lake. A concise sentence or two on its ecological value, socio-economic importance, or role in local fisheries would help readers unfamiliar with the region appreciate why this research matters.

Authors response: the socioeconomic importance of the lake Hawassa was included in the manuscript.
Lines 18–25: Please specify the number of fish examined and list the species included in the study. Clear reporting of sample size and species composition is essential for evaluating the validity and representativeness of the findings.

Authors response: the overall sample size is, 265;

1. *O. niloticus* (tilapia), n=135,
2. *B. intermedius ('nech asa')* also known as *Labeobarbus intermedius*, n=87,
3. *C. gariepinus* (African catfish), n=43.

Lines 27–28: The information provided here relates to study procedures and should be relocated to the Methodology section. This will improve the organisation and scientific flow of the manuscript.

Authors response: ok. The manuscript updated accordingly

Lines 27–39: The reporting in this section requires substantial reorganisation for clarity and scientific accuracy.

-Dactylogyrus is not an internal parasite. Please correct its placement. Dactylogyrus spp. are monogenean ectoparasites located on gills and should not be grouped with internal helminths.

-For scientific clarity, this whole section would be much stronger if reorganised by major taxonomic groups, rather than by host species. Grouping the results this way will make the ecological patterns easier to follow and will align with standard parasitological reporting practices.

A suggested structure is:

o Nematodes (e.g., Contracaecum)

o Digenean trematodes (e.g., Clinostomum, Euclinostomum, Diplostomum, encysted metacercariae)

o Monogeneans (Dactylogyrus)

o Myxozoans (Myxobolus spp.)

o Crustacean ectoparasites (Argulus, Dolops)

o Hirudinea (leeches)

Authors: The suggestions were included in the updated manuscript.

-The sentence listing sample sizes (Oreochromis niloticus n=135, Clarias gariepinus n=87, Barbus intermedius n=43) should appear at the beginning of the Results section or in the Methods, not within the middle of the parasite description.

Author response: the updated manuscript is provided with correct structure.

-The overall infection rate (88.30%, n=234) is useful, but please clarify:

o Infection versus infestation terminology

o Whether the 1748 parasites represent total count or mean counts per species

Authors response: from 265 fish sampled 235 of them were affected with one or more internal or external parasites. The total 1748 parasites are collected ad counted from all positive fishes

o Whether prevalence and intensity values are calculated per parasite taxon or per host species

Maybe you could write it something like this, please checj for accuracy: A total of 265 fish were examined, including Oreochromis niloticus (n = 135), Clarias gariepinus (n = 87), and Barbus intermedius (n = 43), with an overall infection/infestation rate of 88.3% (n = 234). In total, 1,748 parasites were collected and classified into nematodes, digenean trematodes, monogeneans, myxozoans, crustacean ectoparasites, and leeches. The most common nematodes were Contracaecum spp., particularly prevalent in C. gariepinus (50.57%). Digenean trematodes included Clinostomum, Euclinostomum, Diplostomum, and encysted metacercariae, with the latter reaching 68.97% in C. gariepinus. Monogenean infections with Dactylogyrus spp. occurred mainly in O. niloticus (28.15%). Myxobolus spp., leeches, Argulus, and Dolops species were also recorded across hosts. Mean parasite intensity was 8.59 in B. intermedius, 7.92 in C. gariepinus, and 6.79 in O. niloticus, while mean abundance was 7.40, 7.37, and 5.84, respectively.

Authors response: The suggestion accepted in good look, Thank you

Lines 99–116 and elsewhere:
Please ensure that “spp.” is not italicised throughout the manuscript. Only the genus and species names should appear in italics (e.g., Contracaecum spp.), while the abbreviation “spp.” always remains in regular font.

Authors response: The taxonomy of the parasite corrected in the manuscript

Lines 117 to 118: please provide a reference.

Authors response: ok, we have added relevant reference

Lines 129–130: Please clarify whether this statement refers specifically to the situation in Ethiopia. If it does not relate directly to Ethiopia or is not supported by local data, I recommend deleting the sentence to maintain accuracy and avoid overgeneralisation.

Authors response: ok. We have corrected the wording

Lines 129–142: You may also wish to consider the impacts of parasites on fish behaviour, as this is an increasingly recognised aspect of fish health and ecology. Behavioural alterations caused by parasitic infections can influence feeding, predator avoidance, reproduction, and overall survival, thereby affecting population dynamics and fisheries productivity. Including a brief acknowledgement of this dimension would enrich the Discussion and provide a more holistic interpretation of your findings. If relevant, you may consult recent literature on parasite-induced behavioural changes in fish for additional context. For example:
Author response: OK, thanks, we have used the suggested references to enhance our discussion

Animals 2024 Vol. 14 Issue 9 Pages 1315
Applied Animal Behaviour Science 2022 Pages 105722
Australian Freshwater Sciences Society. Waurn Ponds, 1-4 December. 2019
Behaviour 2012 Vol. 149 Issue 6 Pages 601-622
Int J Parasitol 1995 Vol. 25 Issue 12 Pages 1371-1383
J Exp Biol 2013 Vol. 216 Issue 1 Pages 18-26
L. Rogers, E. Sales, S. Shamsi, R. K. Kopf and R. Freire
Parasitol Res 2021 Vol. 120 Issue 2 Pages 515-523

Line 143: I recommend replacing the term "developing country" with "low-income country" when referring to Ethiopia. The phrase "developing country" can be overly broad and does not reflect the many areas in which Ethiopia is already well-developed culturally, scientifically, and economically. Using "low-income country" provides clearer, more precise context and aligns with current international terminology.

Authors response: ok. We have replaced the term 'developing country' with 'low and middle-income country'.

Tables:
Please add horizontal lines and clearer structural formatting to the tables to improve readability. At present, the data are difficult to follow, and adding lines (for example, separating header rows from data rows and delineating major sections) will make the tables much easier for readers to interpret. Consistent alignment of columns and spacing will further enhance clarity.

Authors response: the tables are clearly separated by lines.

Discussion
Please expand the Discussion to address the limitations of using visual inspection alone for parasite detection. While visual examination is acceptable and widely used in field-based parasitology studies, it has well-recognised limitations. Without applying digestion methods (see Canadian Journal of Fisheries and Aquatic Sciences 1986 Vol. 43 Issue 7 Pages 1312-1317) or incubation techniques (see International Journal of Food Microbiology 2016 Vol. 227 Pages 13-16), it is likely that some small or deeply encysted parasites, early developmental stages, or low-intensity infections were not detected. Acknowledging this limitation is important for transparency and will actually strengthen your study. It highlights that the parasite diversity and prevalence in Lake Hawassa may be even higher than reported, reinforcing the significance of your findings and the need for further, more sensitive diagnostic approaches in future research.

Authors response: the limitation of the current finding is clearly stated

Discussion (lines 315-317, 329 to 331 and more): Some parts of the Discussion section repeat information already presented in the Results. These repeated descriptions should be removed to avoid redundancy. The Discussion should focus on interpreting the findings, comparing them with previous studies, exploring implications, and acknowledging limitations. Please revise by deleting the sections that duplicate the Results, including lines XXX, and ensure the Discussion provides insight rather than repetition.

Authors response: Thank you. We have addressed the issues according to the reviewers' comments.

Lines 428–431:
Clinostomum is a particularly important parasite, and the Discussion would benefit from addressing aspects beyond simple prevalence comparisons. I encourage the authors to expand this section by briefly discussing:
• Key features of the life cycle (including the role of snails, fish, and piscivorous birds),
• Its pathogenic significance, especially its occurrence in the skull, musculature, and other critical tissues,
• Implications for fish health, fish welfare, and potential zoonotic considerations, and
• Any ecological or economic consequences relevant to Lake Hawassa or similar systems.
Including a short synthesis similar to discussions found in recent studies on Clinostomum—for example, research addressing tissue localisation, pathological effects, and host–parasite interactions—would enrich the manuscript and provide readers with a more comprehensive understanding of the importance of this genus.
Some articles that authors may find useful to read are suggested below:
Aquaculture 2021 Vol. 539 Pages 736584
DOI: https://doi.org/10.1016/j.aquaculture.2021.736584
Journal of helminthology 2020 Vol. 94
Korean J Parasitol 2019 Vol. 57 Issue 2 Pages 175-177
Parasitology Research 2021 Vol. 120 Pages 2793-2803
Revue de Médecine Vétérinaire 2018 Vol. 7 Pages 147-151
Syst Parasitol 2018 Vol. 95 Issue 6 Pages 543-566
Line 445:
The co-occurrence of Clinostomum and Euclinostomum is an interesting and noteworthy finding. You may wish to highlight this more clearly, as such combinations can offer insights into shared intermediate hosts, environmental conditions, or ecological overlap. The authors may also find relevant comparisons in studies from India (Parasitology Research 2022 Vol. 121 Issue 11 Pages 3083-3089) where similar patterns have been reported. Including a brief mention of that work could help contextualise your findings and strengthen the Discussion.

Figures:
The figures require substantial improvement in multiple aspects. I strongly recommend that the authors revise them with guidance from a senior researcher experienced in parasitological imaging and figure preparation. At present, several issues affect clarity and scientific value:
• In some images, it is unclear what the arrows are pointing to. Please ensure arrows clearly indicate the diagnostic structures or lesions of interest.
• Some figures contain multiple images but lack labels (e.g., A, B, C) or accompanying explanations, making it impossible for the reader to understand what each panel represents.
• Scale bars and magnification details are missing or inconsistent.
• Image quality varies considerably, and some photographs are too dark, blurry, or low resolution for reliable interpretation.
Improving these elements will significantly enhance the scientific credibility of the manuscript. Clear, well-labelled, high-quality figures are essential for parasitology work, particularly when morphological identification is involved.

---

6. PLOS authors have the option to publish the peer review history of their article (what does this mean?). If published, this will include your full peer review and any attached files.

If you choose "no", your identity will remain anonymous but your review may still be made public.

Do you want your identity to be public for this peer review? For information about this choice, including consent withdrawal, please see our Privacy Policy.

Reviewer #1: No

Reviewer #2: No

Reviewer #3: **Yes:** José Alberto Iannacone Oliver, Ph.D.

Reviewer #4: No

Dear Author

I sincerely appreciate the opportunity to review this article, which is titled:

Prevalence of fish parasites from Lake Hawassa, Ethiopia

Manuscript Number: PONE-D-25-59453

That is a good, clear title, but it could be significantly improved by adding more specificity about the hosts and the study's key finding, which increases its impact. This is a great starting point for a manuscript, but there are several points that require clarification or correction to meet high scientific standards.

Here is a point-by-point scientific review:

The manuscript is efficient, but the lack of citations for key statistics and claims is a serious issue that must be addressed before submission. You also need to correct the simple grammar and spelling errors noted in Ms. Strengthening the opening statements, maintaining scientific accuracy, and attending to the requirement for citations are the main objectives of this review.

1- Phrasing and Grammar (Line 13):

for scientific writing, the phrase "Fish provides... to human being" is a little too casual.
Author response: Ok, It is revised to be scientifically sound

2- Use common plural or collective terms. "Fish provides quality protein, various vitamins and minerals essential for human consumption (or humans)," is the recommendation.
Author response: Ok, the suggestion is well appreciated and sincerely accepted.

1. Errors in Grammar and Typography (Lines 15–17): Required: Fix both mistakes.

   There are two spelling and grammar mistakes:

   A-"The parasite fish of Lake Hawassa are..." (L15)

   B-"...limited reports on the type of pastes..." (L17):

Author response: we have corrected both mistakes

1. The statistic "More than17 % of animal protein for global population is obtained from fish" is accurate and needs to be backed up by a recent, trustworthy source (such as an FAO report). Required: add this assertion, include a citation.
   Author response: We have referenced FAO and regional study,

1. FAO, 2018. Food and Agriculture Organization of the United Nations, *The State of World Fisheries and Aquaculture 2018—Meeting the Sustainable Development Goals*; FAO: Rome, Italy, 2018.
2. Obiero, K., Meulenbroek, P., Drexler, S., Dagne, A., Akoll, P., Odong,R., Kaunda-Arara, B., Waidbacher, H.,2019. The Contribution of Fish to Food and Nutrition Security in Eastern Africa: Emerging Trends and Future Outlooks, https://doi.org/10.3390/su11061636

2. Given that your study only looks at one lake (Hawassa), the assertion that fish parasites are the "main cause of the significant threat to the fish biodiversity of Ethiopia's freshwater islands" is very strong and needs immediate, specific evidence or citation. Another non-standard term that needs to be defined is "freshwater islands" (e.g., freshwater ecosystems or specific lakes). Needed Action: Modify the assertion or give an immediate explanation. Concentrate the claim on commercial species or aquaculture if those are the main threats.

Author response: While we have explained the statement with recent reference our intention was to provide global scenario in the background instead of only Ethiopian context.

We have also cited

Power, C., Shirakashi, S., Bott, N.J., Nowak, B.F. (2025). Parasites in Aquaculture. In: Smit, N.J., Sures, B. (eds) Aquatic Parasitology: Ecological and Environmental Concepts and Implications of Marine and Freshwater Parasites. Springer, Cham. https://doi.org/10.1007/978-3-031-83903-0_23 , to explain the issue more

3. Methodes;
 Clarify these points
4. Sampling Location & Method: Where and how were the fish collected?

5. Sample Size (N):
6. What was the total number of fish examined?
7. Taxonomic Keys: Name the primary identification guides used.
8. Statistical Detail: Specify the significance level (e.g., $P < 0.05$) and the software used.

Author response: in methodology we have updated that the samples were collected from different fishing centres/corners of the lake during repeated visiting and the researcher was one responsible for all the data collected and analysed here. Fishermen's have supported us as they have different meshes.

9. **Results:**

10. The numbered of fish samples (N) n=135 (*Oreochromis niloticus*), n=87 (*Clarias gariepinus*), and n=43 (*Barbus intermedius*). **Total N=265**.**Correction:** The text states the overall infection rate is 88.30% (n=234). This indicates that **N=265**

fish were examined (135+87+43=265), and 234 were infected. This number (265) should be explicitly stated as the total Sample Size for clarity.

Author response: 265 is the total sample size, Ok. We have explained it in the updated manuscript

-The design of the Study and Sampling Method (Lines 169–173);
- Contradictory Sampling Terminology (Line 171): Critique: In conventional scientific methodology, the term "systematic random sampling technique" is usually ambiguous. Simple random sampling, in which each fish has an equal chance of being selected, and systematic sampling, such as every numbered of fish, are two different approaches.
- Could be rewriting the precise process that is employed in the field.

Author response: we have defined the sampling technique and methodology in the updated manuscript

11. Have you taken random subsamples from the entire catch? Use either "Simple Random Sampling" or "Random Subsampling." Employ "Systematic Sampling." I suggest precisely define the procedure.

Author response: Systematic Sampling was employed only to select the fish from the net, for example every 10th from the cage/net

1. Duration Clarity (Line 173):

2. Remark: The sentence "The assessment was conducted in all for over 10 months" is grammatically incorrect. Correct to: Make the wording simpler." The assessment was conducted over a period of ten months, from October 2023 to July 2024," is the recommendation.

Author response: ok, we have applied in the updated manuscript

3. Justification of the Study Design (Lines 169–170):

The 10-month period may suggest seasonal comparisons (longitudinal element), which are frequently required in parasitology, even though a "cross-sectional study" is suitable for prevalence at a specific moment in time. I suggest rewriting the sentence to account for seasonal variations in prevalence. You should indicate that the cross-sectional data collection was repeated monthly if you are analyzing data by season or month. Reiterate that the goal is to provide a thorough overall analysis if all 10 months of data are combined. Months of data are pooled, reaffirm that the purpose is to provide a comprehensive overall prevalence estimate for the study period Population under Study (Lines 174–183).

Author response: the ten months data collection period is normal data collection period for simple random sampling, duration of almost one year. Our intention was not to include seasonal variation, but we have focused on parasite epidemiology.

- (Lines 181–183): The exact percentages of total production that the primary fish species (*O. niloticus*, *C. gariepinus,* &L. intermedius) contributed are based on a 1996

reference (LFDP, 1996). The ecosystem of Lake Hawassa has been severely impacted, and it is very likely that community structure has changed over the past thirty years. Updated recent reference to confirm or update these proportions (within the last ten years). Add recent references.

Authors response: Ok, https://doi.org/10.1016/j.heliyon.2023.e18661 is added to describe the regional importance of those fishes
Wubie, A., Teshome, K., Tesfaye, G., 2023. Potential fish yield and biology of some fish in Lake Hertale, Afar regional state, Ethiopia. Heliyon, 9(8):e18661. doi: 10.1016/j.heliyon. 2023.e18661.

-Convention for Scientific Naming (Lines 177–179): The three large fish species and the three small fish species (*Barbus paludinosis, Aplocheilichthys antinorii*, and *Gara quadrimaculata*) are identified by their scientific names. The genus and species name must be written out completely when referring to a species for the first time (e.g., *Oreochromis niloticus*). The genus abbreviation (*O. niloticus*) may be used in later applications. Required make sure the names of all other species (including the minor species mentioned here) adhere to this rule throughout the manuscript but use the abbreviation correctly for *O. niloticus* and *C. gariepinus* later in line (179).
Authors response: the full species name was initially defined in abstract, line 19-20, we have updated it accordingly.

3. Lines 194-209; Categorization and Sampling
   1. Vague Sampling Methodology (Line 194): It is not enough to say that "randomly picked those fishes from different sampling points."
   - Required: Describe the precise capture technique (such as electrofishing, cast nets, or gill nets) and how bias was prevented at each sampling location by ensuring true random selection (such as systematic selection of size classes).
   Authors response: We have used simple random sampling but systematically to every day to achieve the calculated sample size, as you have suggested. We have updated in the methodology
   2. Lines 195-199: Measurement Consistency Only text is mentioned for the final measurement and categorization (L198-199), despite the fact that both Total Length, and Standard Length were initially measured (L195-196).
   - Action Needed: State clearly which length measurement (Total Length or Standard Length) was used for all analysis and categorization, and why, if both were initially recorded.

Authors response: Although both Total Length (TL) and Standard Length (SL) were initially recorded, standard length (SL), was used for all statical analysis and size categorization in this study. SL was selected because it provides a more consistent and reliable measure of body size, excluding the caudal fin, which can be subject to damage, regeneration, or measurement variability. Using SL therefore minimizes measurement bias and improves comparability

among individuals. Total Length was recorded for descriptive purposes and comparison with previous studies.

4. **Line 197: Clarity on Sex Determination Timing** The text implies initial assessment of sex, but confirmation is done "after dissection. **Action Needed:** Rephrase for clarity, e.g., "The sex of each fish was tentatively assessed externally and confirmed post-dissection by noting the presence of testes or ovaries (Imam and Dewu, 2010)."
5. **Authors response:** ok, we have updated accordingly
6. **Lines 200-206: Lack of Justification for Categorical Breakpoints** While classifying fish by size is crucial (L207-209), the specific breakpoints for Length (Class I, II, III) and Weight (Class I, II, III) for the three species are only referenced to an external paper (Tesfaye et al., 2020). **Could be** Provide a brief biological justification for why these specific size ranges (e.g., 50 cm and 75 cm for *C. gariepinus* length were chosen as breakpoints, beyond citing the reference. Are they linked to sexual maturity or dietary shifts in this specific ecosystem?

Authors response: The selected size breakpoints for length and weight classes were not arbitrarily adopted from Tesfaye et al. (2020) but reflect biologically meaningful transitions in growth and life-history stages. For example, in Clarias gariepinus, the length thresholds of 50 cm and 75 cm correspond approximately to ontogenetic stages associated with sexual maturation and trophic shifts. Individuals below 50 cm are typically juveniles or subadults with developing gonads and a diet dominated by invertebrates and small prey. Fish between 50–75 cm generally represents sexually mature adults, while individuals exceeding 75 cm are fully mature, larger-bodied predators with broader dietary niches and higher trophic positioning. These ontogenetic changes influence exposure risk to parasites through altered feeding behaviour, habitat use, and immune competence. Therefore, the selected size classes reflect biologically relevant developmental stages that may affect parasite acquisition dynamics within this ecosystem, rather than being purely arbitrary statistical categories.

7. Lines 200–206: The categorical breakpoints are not justified: The precise breakpoints for Length (Class I, II, III) and Weight (Class I, II, III) for the three species are only cited in an external paper (Tesfaye et al., 2020), despite the fact that size classification of fish is essential (L207-209).
8. Needed Action: Beyond simply mentioning the source, give a succinct biological explanation for the selection of these particular size ranges as breakpoints (e.g., 50 cm and75 cm for *C. gariepinus* length). Are they associated with changes in diet or sexual maturity in this particular ecosystem?
9. **Authors response:** The selected size breakpoints for length and weight classes were not arbitrarily adopted from Tesfaye et al. (2020) but reflect biologically meaningful transitions in growth and life-history stages. For example, in Clarias gariepinus, the length thresholds of 50 cm and 75 cm correspond approximately to ontogenetic stages associated with sexual maturation and trophic shifts. Individuals below 50 cm are

typically juveniles or subadults with developing gonads and a diet dominated by invertebrates and small prey. Fish between 50–75 cm generally represents sexually mature adults, while individuals exceeding 75 cm are fully mature, larger-bodied predators with broader dietary niches and higher trophic positioning. These ontogenetic changes influence exposure risk to parasites through altered feeding behaviour, habitat use, and immune competence. Therefore, the selected size classes reflect biologically relevant developmental stages that may affect parasite acquisition dynamics within this ecosystem, rather than being purely arbitrary statistical categories.

- Lines 210-242: Antemortem and Postmortem Identification of Parasite

- Clarify the method in Transport/Euthanasia (Lines 212-213): The fish were "examined alive" (L211) and then transported "in an icebox" (L212). The icebox suggests chilling or freezing, which would kill the fish or severely inhibit parasite motility. **Action Needed**: Specify the exact conditions: Were the fish euthanized immediately after the initial examination and before transport, or were they transported alive in a cooled, aerated environment?

10. If an icebox was used, state that the fish were immediately sacrificed (euthanized) upon collection to prevent autolysis and preserve parasites.
11. Authors response: Fish were initially examined alive for external abnormalities immediately upon capture. They were then humanely euthanized at the sampling site and placed in an icebox containing crushed ice for transport to the laboratory. The use of an icebox was solely to retard autolysis and preserve parasite integrity for accurate postmortem examination. Fish were not transported alive.
12. Line 216: Ectoparasite Harvesting Solution The text mentions harvesting external parasites into "physiological salt solution" and "fresh water."
13. **Action Needed:** Standardize the solution. Fresh water is highly likely to cause osmotic shock and rupture delicate parasites (like protozoans or monogeneans), making identification difficult. Specify the *concentration* of the physiological saline

Author response: Ectoparasites collected from the skin, fins, and gills were immediately transferred into 0.9% physiological saline solution (NaCl) to maintain osmotic balance and preserve parasite morphology during examination. The use of isotonic saline prevented osmotic shock that could distort or rupture delicate protozoans and monogeneans. No fresh water was used during parasite harvesting or processing.

14. **Line 227: Parasite Clearing Concentration (**Using 99% lactophenol for 24 hours to clear the parasite cuticle for identification may be too aggressive and could destroy internal structures. **Correct:** Justify why use of 99% lactophenol concentration. Often, a 70% alcohol fixation followed by a temporary mount in

a standard clearing agent is preferred, or a gentler, more diluted concentration is used for prolonged periods.

Authors response: Parasite specimens were first fixed in 70% ethanol to preserve internal morphology. For identification, selected specimens were temporarily mounted and cleared in lactophenol for approximately 24 hours to enhance visualization of sclerotized and internal structures.

Furthermore, I recommend you focus on revising the transport conditions and correcting the severe temperature error in the digestion protocol

-**Lines: 230 and 233;** Major **Typographical Error in Digestion Temperature;** the artificial digestion temperatures of 420C and 400C are physically and biologically impossible. **Correction:** These should be corrected to biological temperatures, likely 42C and 40C, respectively. This is a critical error in the methodology that must be fixed.

Authors response: it is typo error, we have corrected in the updated manuscript, the maximum digestion temperature is 42^0C.

-**Line 234: Insufficient Digestion Time;** Digesting 100g of fish flesh at 40C for only 20 minutes is highly unlikely to achieve "fully homogenise the tissue sample."

- **Correction:** Justify the 20-minute duration. Typical tissue digestion protocols for larval parasite recovery often require 2 to 6 hours, sometimes with intermediate steps or agitation, to ensure maximum yield. If a very short time was used, the recovery efficiency should be discussed or validated.

Authors response: Ok. We have updated the digestion protocol

-**Lines 228, 236: Target of Digestion**

-The digestion method is used to harvest "any encysted larva in the fish muscle" (L228), and the straining retained material "was examined for any larvae of nematode" (L236).

Ok. Thanks

15. **Could be corrected:** The subsequent morphological checks (L240-242) clearly point to Anisakid nematodes (like *Anisakis* or *Contracaecum*). State that the method was specifically optimized for the recovery of infective larval nematodes (e.g., L3 stages of Anisakidae) from the musculature is relevant here to show why this muscle digestion is necessary.

Authors response: we have used the suggestion and updated accordingly

16. I recommend: How common, how many and how strong (Lines 267–274) - Ecological metrics don't have any measures of dispersion (Lines 270–272): Critique: This is the biggest problem. Mean Intensity (MI) and Mean Abundance (MA) are ecological statistics that change a lot. They are given as point estimates, with a minimum of 5.37 and a maximum of 8.08. Without a measure of variability, these numbers don't mean anything statistically or can be compared.

17. Action Needed: You need to report both Mean Intensity and Mean Abundance with a measure of dispersion, which is usually the Standard Deviation (SD) or the Standard Error (SE).
- Suggestion: Show the data for each of the three fish species as MI ± SD and MA ± SD.

18. Authors response: we have updated, Thank you

19. Unclear Minimum/Maximum Reporting (Lines 270–272):
Critique: The minimum and maximum values for MI and MA are presented across species without comprehensive context. It is clear that *C. gariepinus* had the lowest MI (5.37) and *B. intermedius* had the highest (8.08), but this should be made clear and checked against the reported values.
What needs to be done: Clearly state: "*C. gariepinus* had the lowest mean intensity (MI = 5.37 pm SD), and *B. intermedius* had the highest (MI = 8.08 pm SD)."
authors response: Thank you, we have included the suggestion
20. Lines 270–272: Ignoring Dispersion Measures for Ecological Metrics
-This is the biggest error. Ecological statistics that vary greatly include Mean Intensity (MI) and Mean Abundance (MA). They are given as point estimates, with a minimum of 5.37 and a maximum of 8.08. Without a measure of variability, these metrics lack statistical significance and comparability.
-Action Required: A measure of dispersion, usually the Standard Deviation (SD) or the Standard Error (SE), must be included with both Mean Intensity and Mean Abundance. I Suggested: for each of the three fish species, display the data as MI -SD and MA -SD.
authors response: Thank you, we have included the suggestion

21. Lines: 273–274: Reference to the Critical Table. Could be corrected the text in (Table 1) specifically cites for the statistical data and makes reference to the Chi-square test result.
- The raw counts for prevalence (infected fish / total fish) for each species, which were used to create the chi 2 statistic, must be included in Table 1. The sentence that introduces the statistic must come before or right after the table.
22. Ok. We have updated the manucrip
23. Line (274): Reporting P-Values, while it is acceptable to report P>0.05, it is preferable to report the precise P-value for non-significant results, unless it is extremely large (e.g., P = 0.123).
- Required correction: Substitute the actual P-value for P>0.05, if it is available.
Authors response: we have updated accordingly
24. Line 289: Typographical Error in Anatomical Location

25. Remark: The text incorrectly identifies the *Clinostomum* infection site as "branchial cavity, pericardial cavity, premium and ocular cavity." Most likely, the word "premium" is misspelled.
-Required correction: To match the exact description later given in Table 3, this should be changed to a standard anatomical term, most likely "peritoneum," "pharyngeal muscles," or "cranium and pharyngeal teeth"
Authors response: we have updated the manuscript accordingly, thanks
26. Line (308). Generally
27. All figured contain photos are undistinct and very low quality, replace the Fig. by more distinct photos
28. All figures could be distinct morphological characters to confirmed the key of parasites identifications

Authors response: we have improved the image quality and the indicators.

Internal Figure Reference Error in Line 290: Remark: The text notes that *Euclinostomum* is common and that it affects different organs (figure 3). There was confusion because Figure 3 was already cited for *Clinostomum* (Line 288). Required correction: to guarantee that figures are uniquely cited in the text, confirm and update the figure reference for *Euclinostomum* (for example, if Figure 4 depicts *Euclinostomum,* then refer to Figure 4 here).

Authors response: figure 3 depicts *Clinostomum* while figure 4 indicates *Euclinostomum*

-Misspelling in Parasite Name (Lines 293 and 299): Remark: The parasite is frequently spelled incorrectly as "Contracecum" (Line 299) and *"Contacaecum"* (e.g., Line 293), throughout the manuscript, the spelling "*Contracaecum"* needs to be corrected.

Authors response: We have corrected the parasite name in the manuscript

29. Line 295: Inaccurate dominant helminth identification: "Digenean encysted metacercaria" (68.97% prevalence) is the term used in the text to describe the dominant parasite in *C. gariepinus*. Since *O. niloticus* was found to contain certain trematodes (*Clinostomum, Euclinostomum*), the inability to identify the dominant digenean in *C. gariepinus* at least to genus
30. Level indicates challenging morphology or insufficient analysis.
- Could be explaining in brief why this dominant parasite was unable to be identified down to the genus level (for example, "due to heavy encapsulation preventing morphological observation," or "given the high intensity and difficulty of differentiation, these were categorized generally as Digenean metacercariae").

Authors response: The dominant helminth in Clarias gariepinus was recorded as digenean encysted metacercaria (68.97% prevalence). Identification to genus level was not possible due to heavy encapsulation within host tissue, which obscured key morphological features required

for precise taxonomic determination. Given the high intensity and overlapping morphology of the cysts, these parasites were conservatively categorized as digenean metacercariae. In contrast, O. niloticus harbored trematodes such as Clinostomum and Euclinostomum, which were identifiable based on well-preserved sclerotized structures.

-Lines 297–298: Methodology Placement: Your calculation method is clarified by the statement that "prevalence report is calculated considering multiple parasite infestation from single host..."
-Required correction: Transfer this explanation to the Methods section, where you specify how prevalence, mean intensity, and mean abundance are calculated (more precisely, in the 2.5.3 Data Analysis sub-section).

Author response: Thank you for the suggestion. We have moved the suggested text to the Methods section accordingly.
