# Supplementary material for: Prevalence of fish parasites from Lake Hawassa, Ethiopia
Source: PLoS One. 2026 Sep 11;21(9):e0357816. doi: 10.1371/journal.pone.0357816 (PMC13568496; doi:10.1371/journal.pone.0357816)
Supplement: Supplementary file 4 [file pone.0357816.s002.docx]

Dear Author

I sincerely appreciate the opportunity to review this article, which is titled:

Prevalence of fish parasites from Lake Hawassa, Ethiopia

Manuscript Number: PONE-D-25-59453

That is a good, clear title, but it could be significantly improved by adding more specificity about the hosts and the study's key finding, which increases its impact. This is a great starting point for a manuscript, but there are several points that require clarification or correction to meet high scientific standards.

Here is a point-by-point scientific review:

The manuscript is efficient, but the lack of citations for key statistics and claims is a serious issue that must be addressed before submission. You also need to correct the simple grammar and spelling errors noted in Ms. Strengthening the opening statements, maintaining scientific accuracy, and attending to the requirement for citations are the main objectives of this review.

1- Phrasing and Grammar (Line 13):

for scientific writing, the phrase "Fish provides... to human being" is a little too casual.

2- Use common plural or collective terms. "Fish provides quality protein, various vitamins and minerals essential for human consumption (or humans)," is the recommendation.

- Errors in Grammar and Typography (Lines 15–17): Required: Fix both mistakes.

  There are two spelling and grammar mistakes:

  A-"The parasite fish of Lake Hawassa are..." (L15)

  B-"...limited reports on the type of pastes..." (L17)
- The statistic "More than17 % of animal protein for global population is obtained from fish" is accurate and needs to be backed up by a recent, trustworthy source (such as an FAO report). Required: add this assertion, include a citation.
- Given that your study only looks at one lake (Hawassa), the assertion that fish parasites are the "main cause of the significant threat to the fish biodiversity of Ethiopia's freshwater islands" is very strong and needs immediate, specific evidence or citation. Another non-standard term that needs to be defined is "freshwater islands" (e.g., freshwater ecosystems or specific lakes). Needed Action: Modify the assertion or give an immediate explanation. Concentrate the claim on commercial species or aquaculture if those are the main threats.
- Methodes;

Clarify these points

- Sampling Location & Method: Where and how were the fish collected?
- Sample Size (N): What was the total number of fish examined?
- Taxonomic Keys: Name the primary identification guides used.
- Statistical Detail: Specify the significance level (e.g., P < 0.05) and the software used.
- **Results:**
- The numbered of fish samples (N) n=135 (*Oreochromis niloticus*), n=87 (*Clarias gariepinus*), and n=43 (*Barbus intermedius*). **Total N=265**.**Correction:** The text states the overall infection rate is 88.30% (n=234). This indicates that **N=265** fish were examined (135+87+43=265), and 234 were infected. This number (265) should be explicitly stated as the total Sample Size for clarity.

-The design of the Study and Sampling Method (Lines 169–173);
- Contradictory Sampling Terminology (Line 171): Critique: In conventional scientific methodology, the term "systematic random sampling technique" is usually ambiguous. Simple random sampling, in which each fish has an equal chance of being selected, and systematic sampling, such as every numbered of fish, are two different approaches.
- Could be rewriting the precise process that is employed in the field.

- Have you taken random subsamples from the entire catch? Use either "Simple Random Sampling" or "Random Subsampling." Employ "Systematic Sampling." I suggest: precisely define the procedure.

2- Duration Clarity (Line 173):

- Remark: The sentence "The assessment was conducted in all for over 10 months" is grammatically incorrect. Correct to: Make the wording simpler." The assessment was conducted over a period of ten months, from October 2023 to July 2024," is the recommendation.

3. Justification of the Study Design (Lines 169–170):

The 10-month period may suggest seasonal comparisons (longitudinal element), which are frequently required in parasitology, even though a "cross-sectional study" is suitable for prevalence at a specific moment in time. I suggest rewriting the sentence to account for seasonal variations in prevalence. You should indicate that the cross-sectional data collection was repeated monthly if you are analyzing data by season or month. Reiterate that the goal is to provide a thorough overall analysis if all 10 months of data are combined. Months of data are pooled, reaffirm that the purpose is to provide a comprehensive overall prevalence estimate for the study period Population under Study (Lines 174–183).

- (Lines 181–183): The exact percentages of total production that the primary fish species (*O. niloticus*, *C. gariepinus,* &L. intermedius) contributed are based on a 1996 reference (LFDP, 1996). The ecosystem of Lake Hawassa has been severely impacted, and it is very likely that community structure has changed over the past thirty years. Updated recent reference to confirm or update these proportions (within the last ten years). Add recent references.

-Convention for Scientific Naming (Lines 177–179): The three large fish species and the three small fish species (*Barbus paludinosis, Aplocheilichthys antinorii*, and *Gara quadrimaculata*) are identified by their scientific names. The genus and species name must be written out completely when referring to a species for the first time (e.g., *Oreochromis niloticus*). The genus abbreviation (*O. niloticus*) may be used in later applications. Required make sure the names of all other species (including the minor species mentioned here) adhere to this rule throughout the manuscript, but use the abbreviation correctly for *O. niloticus* and *C. gariepinus* later in line (179).

- Lines 194-209; Categorization and Sampling
  1. Vague Sampling Methodology (Line 194): It is not enough to say that "randomly picked those fishes from different sampling points."
  - Required: Describe the precise capture technique (such as electrofishing, cast nets, or gill nets) and how bias was prevented at each sampling location by ensuring true random selection (such as systematic selection of size classes).
  2. Lines 195-199: Measurement Consistency Only text is mentioned for the final measurement and categorization (L198-199), despite the fact that both Total Length, and Standard Length were initially measured (L195-196).
  **- Action Needed:** State clearly which length measurement (Total Length or Standard Length) was used for all analysis and categorization, and why, if both were initially recorded.
- **Line 197:** **Clarity on Sex Determination Timing** The text implies initial assessment of sex, but confirmation is done "after dissection. **Action Needed:** Rephrase for clarity, e.g., "The sex of each fish was tentatively assessed externally and confirmed post-dissection by noting the presence of testes or ovaries (Imam and Dewu, 2010)."
- **Lines 200-206:** **Lack of Justification for Categorical Breakpoints** While classifying fish by size is crucial (L207-209), the specific breakpoints for Length (Class I, II, III) and Weight (Class I, II, III) for the three species are only referenced to an external paper (Tesfaye et al., 2020). **Could be** Provide a brief biological justification for why these specific size ranges (e.g., 50 cm and 75 cm for *C. gariepinus* length were chosen as breakpoints, beyond citing the reference. Are they linked to sexual maturity or dietary shifts in this specific ecosystem?
- Lines 200–206: The categorical breakpoints are not justified: The precise breakpoints for Length (Class I, II, III) and Weight (Class I, II, III) for the three species are only cited in an external paper (Tesfaye et al., 2020), despite the fact that size classification of fish is essential (L207-209).
- Needed Action: Beyond simply mentioning the source, give a succinct biological explanation for the selection of these particular size ranges as breakpoints (e.g., 50 cm and75 cm for *C. gariepinus* length). Are they associated with changes in diet or sexual maturity in this particular ecosystem?

- Lines 210-242: Antemortem and Postmortem Identification of Parasite

- Clarify the method in Transport/Euthanasia (Lines 212-213): The fish were "examined alive" (L211) and then transported "in an icebox" (L212). The icebox suggests chilling or freezing, which would kill the fish or severely inhibit parasite motility. **Action Needed**: Specify the exact conditions: Were the fish euthanized immediately after the initial examination and before transport, or were they transported alive in a cooled, aerated environment?

- If an icebox was used, state that the fish were immediately sacrificed (euthanized) upon collection to prevent autolysis and preserve parasites.
- Line 216: Ectoparasite Harvesting Solution The text mentions harvesting external parasites into "physiological salt solution" and "fresh water."
- **Action Needed:** Standardize the solution. Fresh water is highly likely to cause osmotic shock and rupture delicate parasites (like protozoans or monogeneans), making identification difficult. Specify the *concentration* of the physiological saline
- **Line 227:** **Parasite Clearing Concentration (**Using 99% lactophenol for 24 hours to clear the parasite cuticle for identification may be too aggressive and could destroy internal structures. **Correct:** Justify why use of 99% lactophenol concentration. Often, a 70% alcohol fixation followed by a temporary mount in a standard clearing agent is preferred, or a gentler, more diluted concentration is used for prolonged periods.

Furthermore, I recommend you focus on revising the transport conditions and correcting the severe temperature error in the digestion protocol

-**Lines: 230 and 233;** Major **Typographical Error in Digestion Temperature;** the artificial digestion temperatures of 420C and 400C are physically and biologically impossible. **Correction:** These should be corrected to biological temperatures, likely 42C and 40C, respectively. This is a critical error in the methodology that must be fixed.

-**Line 234:** **Insufficient Digestion Time;** Digesting 100g of fish flesh at 40C for only 20 minutes is highly unlikely to achieve "fully homogenise the tissue sample."

- **Correction:** Justify the 20 minute duration. Typical tissue digestion protocols for larval parasite recovery often require 2 to 6 hours, sometimes with intermediate steps or agitation, to ensure maximum yield. If a very short time was used, the recovery efficiency should be discussed or validated.

-**Lines 228, 236:** **Target of Digestion**

**-**The digestion method is used to harvest "any encysted larva in the fish muscle" (L228), and the straining retained material "was examined for any larvae of nematode" (L236).

- **Could be corrected:** The subsequent morphological checks (L240-242) clearly point to Anisakid nematodes (like *Anisakis* or *Contracaecum*). State that the method was specifically optimized for the recovery of infective larval nematodes (e.g., L3 stages of Anisakidae) from the musculature is relevant here to show why this muscle digestion is necessary.
- I recommend: How common, how many and how strong (Lines 267–274)
  - Ecological metrics don't have any measures of dispersion (Lines 270–272): Critique: This is the biggest problem. Mean Intensity (MI) and Mean Abundance (MA) are ecological statistics that change a lot. They are given as point estimates, with a minimum of 5.37 and a maximum of 8.08. Without a measure of variability, these numbers don't mean anything statistically or can be compared.
- Action Needed: You need to report both Mean Intensity and Mean Abundance with a measure of dispersion, which is usually the Standard Deviation (SD) or the Standard Error (SE).
  - Suggestion: Show the data for each of the three fish species as MI ± SD and MA ± SD.
- Unclear Minimum/Maximum Reporting (Lines 270–272):
  Critique: The minimum and maximum values for MI and MA are presented across species without comprehensive context. It is clear that *C. gariepinus* had the lowest MI (5.37) and *B. intermedius* had the highest (8.08), but this should be made clear and checked against the reported values.
  What needs to be done: Clearly state: "*C. gariepinus* had the lowest mean intensity (MI = 5.37 pm SD), and *B. intermedius* had the highest (MI = 8.08 pm SD)."
- Lines 270–272: Ignoring Dispersion Measures for Ecological Metrics

-This is the biggest error. Ecological statistics that vary greatly include Mean Intensity (MI) and Mean Abundance (MA). They are given as point estimates, with a minimum of 5.37 and a maximum of 8.08. Without a measure of variability, these metrics lack statistical significance and comparability.
-Action Required: A measure of dispersion, usually the Standard Deviation (SD) or the Standard Error (SE), must be included with both Mean Intensity and Mean Abundance. I Suggested: for each of the three fish species, display the data as MI -SD and MA -SD.

- Lines: 273–274: Reference to the Critical Table. Could be corrected the text in (Table 1) specifically cites for the statistical data and makes reference to the Chi-square test result.
  - The raw counts for prevalence (infected fish / total fish) for each species, which were used to create the chi 2 statistic, must be included in Table 1. The sentence that introduces the statistic must come before or right after the table.
- Line (274): Reporting P-Values, while it is acceptable to report P>0.05, it is preferable to report the precise $P$-value for non-significant results, unless it is extremely large (e.g., P = 0.123).
  - Required correction: Substitute the actual P-value for P>0.05, if it is available.
- Line 289: Typographical Error in Anatomical Location
- Remark: The text incorrectly identifies the *Clinostomum* infection site as "branchial cavity, pericardial cavity, premium and ocular cavity." Most likely, the word "premium" is misspelled.
  -Required correction: To match the exact description later given in Table 3, this should be changed to a standard anatomical term, most likely "peritoneum," "pharyngeal muscles," or "cranium and pharyngeal teeth" Line (308). Generally
- All figured contain photos are undistinct and very low quality, replace the Fig. by more distinct photos
- All figures could be distinct morphological characters to confirmed the key of parasites identifications
  - Internal Figure Reference Error in Line 290: Remark: The text notes that *Euclinostomum* is common and that it affects different organs (figure 3). There was confusion because Figure 3 was already cited for *Clinostomum* (Line 288). Required correction: to guarantee that figures are uniquely cited in the text, confirm and update the figure reference for *Euclinostomum* (for example, if Figure 4 depicts *Euclinostomum,* then refer to Figure 4 here).
  -Misspelling in Parasite Name (Lines 293 and 299):
  Remark: The parasite is frequently spelled incorrectly as "Contracecum" (Line 299) and *"Contacaecum"* (e.g., Line 293), throughout the manuscript, the spelling "*Contracaecum"* needs to be corrected.
- Line 295: Inaccurate dominant helminth identification: "Digenean encysted metacercaria" (68.97% prevalence) is the term used in the text to describe the dominant parasite in *C. gariepinus*. Since *O. niloticus* was found to contain certain trematodes (*Clinostomum, Euclinostomum*), the inability to identify the dominant digenean in *C. gariepinus* at least to genus
- Level indicates challenging morphology or insufficient analysis.
  - Could be explaining in brief why this dominant parasite was unable to be identified down to the genus level (for example, "due to heavy encapsulation preventing morphological observation," or "given the high intensity and difficulty of differentiation, these were categorized generally as Digenean metacercariae").
  -Lines 297–298: Methodology Placement: Your calculation method is clarified by the statement that "prevalence report is calculated considering multiple parasite infestation from single host..."
  -Required correction: Transfer this explanation to the Methods section, where you specify how prevalence, mean intensity, and mean abundance are calculated (more precisely, in the 2.5.3 Data Analysis sub-section). Only the results,
