# Supplementary material for: Prevalence of fish parasites from Lake Hawassa, Ethiopia
Source: PLoS One. 2026 Sep 11;21(9):e0357816. doi: 10.1371/journal.pone.0357816 (PMC13568496; doi:10.1371/journal.pone.0357816)
Supplement: Supplementary file 5 [file pone.0357816.s006.docx]

Rebuttal letter (Response to reviewers and editors)

**Manuscript Title:** Prevalence of fish parasites from Lake Hawassa, Ethiopia

**Manuscript ID:** PONE-D-25-59453

Dear Editor and Reviewers,

We would like to sincerely thank the editor and reviewers for the careful evaluation of our manuscript and for the constructive comments and suggestions. These comments have greatly helped us improve the clarity, quality, and scientific rigor of the manuscript.

We have carefully revised the manuscript according to all comments. All changes are highlighted in the revised version, and detailed responses to each comment are provided below.

1. Please upload a Response to Reviewers letter which should include a point-by-point response to each of the points made by the Editor and / or Reviewers. (This should be uploaded as a 'Response to Reviewers' file type.) Please follow this link for more information: [http://blogs.PLOS.org/everyone/2011/05/10/how-to-submit-your-revised-manuscript/](http://blogs.plos.org/everyone/2011/05/10/how-to-submit-your-revised-manuscript/)
   Authors response: Thank you for bringing this to our attention. We have thoroughly checked the manuscript and supporting files to ensure that they meet PLOS ONE formatting and style requirements, including the appropriate file naming convections. All necessary corrections have been made in the revised submission.
2. To comply with PLOS One submissions requirements, in your Methods section, please provide additional information regarding the experiments involving animals and ensure you have included details on (1) methods of sacrifice, (2) methods of anesthesia and/or analgesia, and (3) efforts to alleviate suffering.

Author response: Thank you for this important comment. We have revised the Methods section to include additional details regarding the animal-related procedures, including the methods of sacrifice, anesthesia and/or analgesia, and the measures taken to minimize animal suffering, in accordance with the PLOS ONE submission requirements.

3. "We note that your Data Availability Statement is currently as follows: All relevant data are within the manuscript and its Supporting Information files

   Please confirm at this time whether or not your submission contains all raw data required to replicate the results of your study. Authors must share the “minimal data set” for their submission. PLOS defines the minimal data set to consist of the data required to replicate all study findings reported in the article, as well as related metadata and methods (<https://journals.plos.org/plosone/s/data-availability#loc-minimal-data-set-definition>).

   For example, authors should submit the following data:
   - The values behind the means, standard deviations and other measures reported;
   - The values used to build graphs;
   - The points extracted from images for analysis.

   Authors do not need to submit their entire data set if only a portion of the data was used in the reported study.

   If your submission does not contain these data, please either upload them as Supporting Information files or deposit them to a stable, public repository and provide us with the relevant URLs, DOIs, or accession numbers. For a list of recommended repositories, please see <https://journals.plos.org/plosone/s/recommended-repositories>.

   If there are ethical or legal restrictions on sharing a de-identified data set, please explain them in detail (e.g., data contain potentially sensitive information, data are owned by a third-party organization, etc.) and who has imposed them (e.g., an ethics committee). Please also provide contact information for a data access committee, ethics committee, or other institutional body to which data requests may be sent. If data are owned by a third party, please indicate how others may request data access."

   Authors response: Dear editor in chief, Thank you for your comment. We confirm that the submission contains all the necessary raw data required to replicate the results of our study. The data used to create those tables are submitted as additional table S1.
4. We note that Figure 1 in your submission contain [map/satellite] images which may be copyrighted. All PLOS content is published under the Creative Commons Attribution License (CC BY 4.0), which means that the manuscript, images, and Supporting Information files will be freely available online, and any third party is permitted to access, download, copy, distribute, and use these materials in any way, even commercially, with proper attribution. For these reasons, we cannot publish previously copyrighted maps or satellite images created using proprietary data, such as Google software (Google Maps, Street View, and Earth). For more information, see our copyright guidelines: <http://journals.plos.org/plosone/s/licenses-and-copyright>.

   We require you to either (1) present written permission from the copyright holder to publish these figures specifically under the CC BY 4.0 license, or (2) remove the figures from your submission:

   1. You may seek permission from the original copyright holder of Figure 1 to publish the content specifically under the CC BY 4.0 license.

   We recommend that you contact the original copyright holder with the Content Permission Form (<http://journals.plos.org/plosone/s/file?id=7c09/content-permission-form.pdf>) and the following text:

   “I request permission for the open-access journal PLOS ONE to publish XXX under the Creative Commons Attribution License (CCAL) CC BY 4.0 (<http://creativecommons.org/licenses/by/4.0/>). Please be aware that this license allows unrestricted use and distribution, even commercially, by third parties. Please reply and provide explicit written permission to publish XXX under a CC BY license and complete the attached form.”

   Please upload the completed Content Permission Form or other proof of granted permissions as an "Other" file with your submission.

   In the figure caption of the copyrighted figure, please include the following text: “Reprinted from [ref] under a CC BY license, with permission from [name of publisher], original copyright [original copyright year].”

   2. If you are unable to obtain permission from the original copyright holder to publish these figures under the CC BY 4.0 license or if the copyright holder’s requirements are incompatible with the CC BY 4.0 license, please either i) remove the figure or ii) supply a replacement figure that complies with the CC BY 4.0 license. Please check copyright information on all replacement figures and update the figure caption with source information. If applicable, please specify in the figure caption text when a figure is similar but not identical to the original image and is therefore for illustrative purposes only.

   The following resources for replacing copyrighted map figures may be helpful:

   USGS National Map Viewer (public domain): <http://viewer.nationalmap.gov/viewer/>

   The Gateway to Astronaut Photography of Earth (public domain): <http://eol.jsc.nasa.gov/sseop/clickmap/>

   Maps at the CIA (public domain): <https://www.cia.gov/library/publications/the-world-factbook/index.html> and <https://www.cia.gov/library/publications/cia-maps-publications/index.html>

   NASA Earth Observatory (public domain): <http://earthobservatory.nasa.gov/>

   Landsat: <http://landsat.visibleearth.nasa.gov/>

   USGS EROS (Earth Resources Observatory and Science (EROS) Center) (public domain): [http://eros.usgs.gov/#](http://eros.usgs.gov/)

   Natural Earth (public domain): <http://www.naturalearthdata.com/>

   Authors response: Thank you for bringing this to our attention. We have thoroughly checked the manuscript and supporting files to ensure that they meet PLOS ONE formatting and style requirements, including the appropriate file naming convections. All necessary corrections have been made in the revised submission.

   5. We note that Figure 2 in your submission contain copyrighted images. All PLOS content is published under the Creative Commons Attribution License (CC BY 4.0), which means that the manuscript, images, and Supporting Information files will be freely available online, and any third party is permitted to access, download, copy, distribute, and use these materials in any way, even commercially, with proper attribution. For more information, see our copyright guidelines: <http://journals.plos.org/plosone/s/licenses-and-copyright>.

   We require you to either (1) present written permission from the copyright holder to publish these figures specifically under the CC BY 4.0 license, or (2) remove the figures from your submission:

   1. You may seek permission from the original copyright holder of Figure 2 to publish the content specifically under the CC BY 4.0 license.

   We recommend that you contact the original copyright holder with the Content Permission Form (<http://journals.plos.org/plosone/s/file?id=7c09/content-permission-form.pdf>) and the following text:

   “I request permission for the open-access journal PLOS ONE to publish XXX under the Creative Commons Attribution License (CCAL) CC BY 4.0 (<http://creativecommons.org/licenses/by/4.0/>). Please be aware that this license allows unrestricted use and distribution, even commercially, by third parties. Please reply and provide explicit written permission to publish XXX under a CC BY license and complete the attached form.”

   Please upload the completed Content Permission Form or other proof of granted permissions as an "Other" file with your submission.

   In the figure caption of the copyrighted figure, please include the following text: “Reprinted from [ref] under a CC BY license, with permission from [name of publisher], original copyright [original copyright year].”

   2. If you are unable to obtain permission from the original copyright holder to publish these figures under the CC BY 4.0 license or if the copyright holder’s requirements are incompatible with the CC BY 4.0 license, please either i) remove the figure or ii) supply a replacement figure that complies with the CC BY 4.0 license. Please check copyright information on all replacement figures and update the figure caption with source information. If applicable, please specify in the figure caption text when a figure is similar but not identical to the original image and is therefore for illustrative purposes only.

Author response: Dear editor in chief, thank you for bringing this issue to our attention. We understand the copyright restrictions related to maps and satellite images created using proprietary data. Although we initially requested permission from the original copyright holder, we have decided to remove the map from Figure 1, as it only represented a very narrow area. Instead, we have revised the manuscript to describe the agroecological characteristics of the Lake Hawassa area in the text.

If the reviewer comments include a recommendation to cite specific previously published works, please review and evaluate these publications to determine whether they are relevant and should be cited. There is no requirement to cite these works unless the editor has indicated otherwise.

Authors response: Thank you for this comment. We have carefully evaluated the publications suggested by the reviewers and have included them as references where they were relevant to the study.

**From the earlier editorial and reviewers’ comments**

1. Please ensure that your manuscript meets PLOS ONE's style requirements, including those for file naming. The PLOS ONE style templates can be found at

<https://journals.plos.org/plosone/s/file?id=wjVg/PLOSOne_formatting_sample_main_body.pdf> and

<https://journals.plos.org/plosone/s/file?id=ba62/PLOSOne_formatting_sample_title_authors_affiliations.pdf>

Authors response: Thank you for bringing this to our attention. We have thoroughly checked the manuscript and supporting files to ensure that they meet PLOS ONE formatting and style requirements, including the appropriate file naming convections. All necessary corrections have been made in the revised submission.

2. In your Methods section, please provide additional information regarding the permits you obtained for the work. Please ensure you have included the full name of the authority that approved the field site access and, if no permits were required, a brief statement explaining why.

Author response: We have communicated city municipality via letter from Wollega University dated 24/04/2023 immediately after obtaining ethical clearance. The Hawassa city administration accepts our letter and permit as for the research to be conducted.

3. To comply with PLOS One submissions requirements, in your Methods section, please provide additional information regarding the experiments involving animals and ensure you have included details on (1) methods of sacrifice, (2) methods of anesthesia and/or analgesia, and (3) efforts to alleviate suffering.

Author response: All the fishes we have used have been captured for commercial reason. We immediately captured fresh fishes with cooperation from fishery person who are professional in capturing fishes and have fish cages. No anaesthesia was used but we process the sample after they stop breathing which is in minutes after they are captured. No suffering exists during sample processing.

4. When completing the data availability statement of the submission form, you indicated that you would make your data available on acceptance. We strongly recommend all authors decide on a data sharing plan before acceptance, as the process can be lengthy and hold up publication timelines. Please note that, though access restrictions are acceptable now, your entire data will need to be made freely accessible if your manuscript is accepted for publication. This policy applies to all data except where public deposition would breach compliance with the protocol approved by your research ethics board. If you are unable to adhere to our open data policy, please kindly revise your statement to explain your reasoning and we will seek the editor's input on an exemption. Please be assured that, once you have provided your new statement, the assessment of your exemption will not hold up the peer review process.

Author response: I agree with journal data availability policy that our data will fully be available without restriction.

5. We note that Figure 1 in your submission contain [map/satellite] images which may be copyrighted. All PLOS content is published under the Creative Commons Attribution License (CC BY 4.0), which means that the manuscript, images, and Supporting Information files will be freely available online, and any third party is permitted to access, download, copy, distribute, and use these materials in any way, even commercially, with proper attribution. For these reasons, we cannot publish previously copyrighted maps or satellite images created using proprietary data, such as Google software (Google Maps, Street View, and Earth). For more information, see our copyright guidelines: <http://journals.plos.org/plosone/s/licenses-and-copyright>.

We require you to either (1) present written permission from the copyright holder to publish these figures specifically under the CC BY 4.0 license, or (2) remove the figures from your submission:

1. You may seek permission from the original copyright holder of Figure 1 to publish the content specifically under the CC BY 4.0 license.

We recommend that you contact the original copyright holder with the Content Permission Form (<http://journals.plos.org/plosone/s/file?id=7c09/content-permission-form.pdf>) and the following text:

“I request permission for the open-access journal PLOS ONE to publish XXX under the Creative Commons Attribution License (CCAL) CC BY 4.0 (<http://creativecommons.org/licenses/by/4.0/>). Please be aware that this license allows unrestricted use and distribution, even commercially, by third parties. Please reply and provide explicit written permission to publish XXX under a CC BY license and complete the attached form.”

Please upload the completed Content Permission Form or other proof of granted permissions as an "Other" file with your submission.

Author response: Dear editor in chief, thank you for bringing this issue to our attention. We understand the copyright restrictions related to maps and satellite images created using proprietary data. Although we initially requested permission from the original copyright holder, we have decided to remove the map from Figure 1, as it only represented a very narrow area. Instead, we have revised the manuscript to describe the agroecological characteristics of the Lake Hawassa area in the text.

In the figure caption of the copyrighted figure, please include the following text: “Reprinted from [ref] under a CC BY license, with permission from [name of publisher], original copyright [original copyright year].”

2. If you are unable to obtain permission from the original copyright holder to publish these figures under the CC BY 4.0 license or if the copyright holder’s requirements are incompatible with the CC BY 4.0 license, please either i) remove the figure or ii) supply a replacement figure that complies with the CC BY 4.0 license. Please check copyright information on all replacement figures and update the figure caption with source information. If applicable, please specify in the figure caption text when a figure is similar but not identical to the original image and is therefore for illustrative purposes only.

The following resources for replacing copyrighted map figures may be helpful:

USGS National Map Viewer (public domain): <http://viewer.nationalmap.gov/viewer/>

The Gateway to Astronaut Photography of Earth (public domain): <http://eol.jsc.nasa.gov/sseop/clickmap/>

Maps at the CIA (public domain): <https://www.cia.gov/library/publications/the-world-factbook/index.html> and <https://www.cia.gov/library/publications/cia-maps-publications/index.html>

NASA Earth Observatory (public domain): <http://earthobservatory.nasa.gov/>

Landsat: <http://landsat.visibleearth.nasa.gov/>

USGS EROS (Earth Resources Observatory and Science (EROS) Center) (public domain): [http://eros.usgs.gov/#](http://eros.usgs.gov/)

Natural Earth (public domain): <http://www.naturalearthdata.com/>

Author response: Dear editor in chief, thank you for bringing this issue to our attention. We understand the copyright restrictions related to maps and satellite images created using proprietary data. Although we initially requested permission from the original copyright holder, we have decided to remove the map from Figure 1, as it only represented a very narrow area. Instead, we have revised the manuscript to describe the agroecological characteristics of the Lake Hawassa area in the text.

6. We note that Figure 2 in your submission contain copyrighted images. All PLOS content is published under the Creative Commons Attribution License (CC BY 4.0), which means that the manuscript, images, and Supporting Information files will be freely available online, and any third party is permitted to access, download, copy, distribute, and use these materials in any way, even commercially, with proper attribution. For more information, see our copyright guidelines: <http://journals.plos.org/plosone/s/licenses-and-copyright>.

We require you to either (1) present written permission from the copyright holder to publish these figures specifically under the CC BY 4.0 license, or (2) remove the figures from your submission:

1. You may seek permission from the original copyright holder of Figure 2 to publish the content specifically under the CC BY 4.0 license.

We recommend that you contact the original copyright holder with the Content Permission Form (<http://journals.plos.org/plosone/s/file?id=7c09/content-permission-form.pdf>) and the following text:

“I request permission for the open-access journal PLOS ONE to publish XXX under the Creative Commons Attribution License (CCAL) CC BY 4.0 (<http://creativecommons.org/licenses/by/4.0/>). Please be aware that this license allows unrestricted use and distribution, even commercially, by third parties. Please reply and provide explicit written permission to publish XXX under a CC BY license and complete the attached form.”

Please upload the completed Content Permission Form or other proof of granted permissions as an "Other" file with your submission.

In the figure caption of the copyrighted figure, please include the following text: “Reprinted from [ref] under a CC BY license, with permission from [name of publisher], original copyright [original copyright year].”

Author response: Dear Editor-in-Chief, thank you for your comment regarding Figure 2 in the earlier submission (now labelled as Figure 1 after revision). We would like to clarify that all images included in this manuscript including Figure 1 were captured by the research team during the study. Therefore, no third-party permission is required for their publication or use for scientific purposes.

2. If you are unable to obtain permission from the original copyright holder to publish these figures under the CC BY 4.0 license or if the copyright holder’s requirements are incompatible with the CC BY 4.0 license, please either i) remove the figure or ii) supply a replacement figure that complies with the CC BY 4.0 license. Please check copyright information on all replacement figures and update the figure caption with source information. If applicable, please specify in the figure caption text when a figure is similar but not identical to the original image and is therefore for illustrative purposes only.

If the reviewer comments include a recommendation to cite specific previously published works, please review and evaluate these publications to determine whether they are relevant and should be cited. There is no requirement to cite these works unless the editor has indicated otherwise. 

[Note: HTML markup is below. Please do not edit.]

Reviewers' comments: Reviewer's Responses to Questions

**Comments to the Author**

1. Is the manuscript technically sound, and do the data support the conclusions?

The manuscript must describe a technically sound piece of scientific research with data that supports the conclusions. Experiments must have been conducted rigorously, with appropriate controls, replication, and sample sizes. The conclusions must be drawn appropriately based on the data presented.

Reviewer #1: Yes

Author response: Thank you, we appreciate your guidance and have carefully considered these aspects in revising our manuscript.

Reviewer #2: Yes

Author response: Thank you, we appreciate your guidance and have carefully considered these aspects in revising our manuscript.

Reviewer #3: No

Author response: we have improved the manuscript as per the reviewers and editor in chief comments.

Reviewer #4: Partly

Author response: Thank you, we have also thoroughly improved the manuscript

2. Has the statistical analysis been performed appropriately and rigorously?

Reviewer #1: Yes

Author response: Thank you

Reviewer #2: Yes

Author response: Thank you

Reviewer #3: No

Author response: we have improved the methodological and statical section of the manuscript

Reviewer #4: Yes

Author response: Thank you

3. Have the authors made all data underlying the findings in their manuscript fully available?

The [PLOS Data policy](http://track.editorialmanager.com/CL0/http:%2F%2Fwww.plosone.org%2Fstatic%2Fpolicies.action%23sharing/1/010f019abede830e-7a74245e-0f28-4b00-a4c0-64d8ce610dbb-000000/ZQCpyoGnjEZu9yO46-wEV7gsQzd3oRiXilsoDtNtDS4=238) requires authors to make all data underlying the findings described in their manuscript fully available without restriction, with rare exception (please refer to the Data Availability Statement in the manuscript PDF file). The data should be provided as part of the manuscript or its supporting information or deposited to a public repository. For example, in addition to summary statistics, the data points behind means, medians and variance measures should be available. If there are restrictions on publicly sharing data—e.g. participant privacy or use of data from a third party—those must be specified.

Reviewer #1: Yes

Author response: Thank you, thank you, we have also thoroughly improved the manuscript

Reviewer #2: Yes

Author response: Thank you, we have also thoroughly improved the manuscript

Reviewer #3: No

Author response: We declare that all data are fully available without restriction

Reviewer #4: Yes

Author response: Thank you, we have also thoroughly improved the manuscript

4. Is the manuscript presented in an intelligible fashion and written in standard English?

PLOS ONE does not copyedit accepted manuscripts, so the language in submitted articles must be clear, correct, and unambiguous. Any typographical or grammatical errors should be corrected at revision, so please note any specific errors here.

Reviewer #1: Yes

Reviewer #2: Yes

Reviewer #3: No

Author response: We have thoroughly edited the manuscript during our current revision.

Reviewer #4: No

Author response: We have thoroughly edited the manuscript during our current revision.

5. Review Comments to the Author

Please use the space provided to explain your answers to the questions above. You may also include additional comments for the author, including concerns about dual publication, research ethics, or publication ethics. (Please upload your review as an attachment if it exceeds 20,000 characters)

Reviewer #1: Dear Author
I sincerely appreciate the opportunity to review this article, which is titled:
Prevalence of fish parasites from Lake Hawassa, Ethiopia
Manuscript Number: PONE-D-25-59453
That is a good, clear title, but it could be significantly improved by adding more specificity about the hosts and the study's key finding, which increases its impact.

Author response: Accordingly, we have significantly improved the manuscript by carefully addressing the suggestions and comments raised by both the reviewers.

This is a great starting point for a manuscript, but there are several points that require clarification or correction to meet high scientific standards.
Here is a point-by-point scientific review:
The manuscript is efficient, but the lack of citations for key statistics and claims is a serious issue that must be addressed before submission.

Author response: we have addressed those suggestions provided by respected reviewer.

You also need to correct the simple grammar and spelling errors noted in Ms.

Author response: ok., thanks

Strengthening the opening statements, maintaining scientific accuracy, and attending to the requirement for citations are the main objectives of this review.

Kindly find the attached file

Author response: we have addressed those suggestions provided by respected reviewer.

Reviewer #2: This manuscript presents a cross-sectional parasitological survey of three commercially important fish species from Lake Hawassa, Ethiopia. While the topic is relevant and the sample size is adequate, the manuscript requires substantial revision before publication.

Author response: we have substantially reviewed, used new and existing references to improve the manuscript

The study provides valuable baseline data on fish parasite prevalence but suffers from some gaps, incomplete taxonomic identification, and inadequate contextualization of findings.

Author response: we have improved the taxonomic identification and hence conceptualisation of the key findings in the result and discussion.

1- Taxonomic Identification Limitations
The authors acknowledge limiting identification to genus level, citing that "fish harbors mostly larval stages" (lines 242-243). However, this is scientifically problematic:
-Species-level identification is feasible: Modern molecular techniques (PCR, DNA barcoding) can identify larval stages to species level. The authors provide no justification for not attempting molecular identification.

Author response: We have indicated the limitation of the current study in the limitation section. PCR and DNA barcoding needs high-tech molecular diagnostic machines which is limited in my institution. This work is purely for academic purpose
-Public health implications: For zoonotic parasites like Contracaecum spp., species-level identification is crucial as different species have different zoonotic potential.

-Ecological interpretation: Genus-level identification limits ecological and epidemiological interpretations.

Question 1: Why were molecular identification methods not employed, especially given the zoonotic importance of several parasites identified? Were resources unavailable or was this a deliberate methodological choice?
Author response: we are unable to extract DNA and perform molecular identification due to limitation of resources and test kits. This research is conducted with the university lecturers without any local or international fund. Here we have performed base line data on epidemiology and assure you to perform molecular test in future projects with collaborated effort
2- water quality parameters:
The manuscript mentions water quality issues (lines 362-377, 488-491, 708-710) but provides NO water quality data. The authors acknowledge environmental stressors but fail to measure them. Multiple references cited (Memberu et al., 2021; Tibebe et al., 2023) demonstrate poor water quality in Lake Hawassa

Author response: we have conducted some questionnaire survey including local leaders, environmental protection officers affiliated to Hawassa Municipal abattoir who have witnessed the availability of water contamination.
Question 2: Were any water quality parameters measured during this study? If not, why was this critical environmental component excluded from a parasitological survey?
Author response: water quality analysis is bit expensive project in Ethiopian context. But the research team clearly observed the contamination of water by offals from the fish, car washing to the lake, discarding of plastics to the lake and the high availability of free roaming birds which could add stress to the fish and create good ground for fish parasites. We can provide some pictures captured by research team. We have questionnaire survey report that indicates human practice around the lake.
3-Temporal Sampling Design
"October 2023 to July 2024" and "conducted in all for over 10 months" (lines 169-173)
-Were fish collected continuously or at intervals?
-What was the sampling frequency?
-Were seasonal variations accounted for?
-Multiple citations (Bakke et al., 2002; Ozer et al., 2004; Khidr et al., 2012; Khan et al., 2018) emphasize seasonal effects on parasite prevalence
-Provide a clear sampling timeline and sample sizes per collection period. Analyze seasonal effects if data permit.

Authors response: we have randomly selected the fish of our target until we reach the desired sample size(n=265). The period mentioned here is including the time spent in preparation to sample processing. During data collection we did not actually consider seasonal effects.

4-Sample Selection
"Systematic random sampling" (line 171): This term is contradictory. Was sampling:
-Systematic? or Random (using random number generators)? or Stratified by species?
-Source of fish unclear: Were fish collected:
=Directly from the lake (by researchers)?
=From fishermen (introducing selectivity bias)?
=From fish markets (introducing preservation/handling artifacts)?
Authors response: the sample was directly collected by the researchers at the lake Hawassa. Systematic random sampling means to indicate that we are randomly selecting the fishes at specific interval; from every 10 fishes at random but systematically to decide which number will be selected during the research day. A
-Sampling locations: No description of geographic sampling within Lake Hawassa
Authors response: we have provided the geographic description of Lake Hawassa and Hawasa city in the updated manuscript.
5- some parasitological methods lack details as magnification power, fixation details...etc
Authors response:
6-Results:
-Table 1 vs. Text discrepancy:
-Table 1 reports 788 parasites from O. niloticus (line 277)
-Text reports 787 parasites (lines 30-31, abstract)
Which is correct?
Authors response: The typo error in abstract is corrected. The correct parasite number from *O. niloticus* is 788.
7-Fish morphometrics:
-No mean ± SD for fish weights and lengths per species
-Weight and length ranges provided (lines 199-206) but no central tendencies
-Impossible to assess if samples represent typical population demographics
Authors response:
8- Parasite intensity data:
-Table 1 shows "Number of parasites from each fish: 6-9, 4-6, 5-10"
-These ranges are unclear: Are these per infected fish? Per parasite species?

Authors response: this number indicates the number of parasites collected from infected fish.

Please also refer the formula for;

Mean intensity=$\frac{Sum of individuals of parasite species in a host sample}{Total no. of infected individuals of the host in the sample}$

-Mean intensities (6.79, 7.92, 8.59) don't align logically with these ranges
Authors response: mean intensity is calculated as the number of parasites collected/the number of fish affected. For example, 116 fishes are infected from O. niloticus, and 788 different parasites are collected. By dividing 788 to 116 we get 6.79. for other fish species we have computed similarly (*El-Shahawy et al., 2017).*
9- Discussion:
-Comparison-heavy, interpretation-light: The discussion is dominated by comparing prevalence values to other studies (lines 329-343) without explaining WHY differences exist
Author response: We have explained the difference in prevalence in relation to parasite ecological, host related factors, and fishing composition (Turner et al., 2021).

Ecological context missing:
-No discussion of Lake Hawassa's specific ecological characteristics
-How do lake depth, temperature, presence of intermediate hosts affect parasite communities?
-What snail species are present (necessary for digenean life cycles)?

Authors response: the ecological features of the lake was updated in the manuscript. ‘Lake Hawassa have temperature range of 21.3^0^C. The surface are ranges from 8500-9000 hectare and the maximum central depth of 18-22m with shoreline ranging from 50-65km in length. While lake Hawassa is the smallest of the rift valley lake, it is rich in phytoplankton and zooplankton that supports the six population of the fish resources’
Zoonotic risk inadequately addressed:

-Contracaecum spp. are identified as zoonotic (lines 421-427) but no risk assessment is provided
-What are fish consumption practices in the region?
-Are fish cooked adequately?
-No connection to the cultural practices mentioned (lines 84-89)
Authors response: there is a tradition of consuming raw or undercooked fresh fish which is the risk factor for anisakiasis like infection.
Typographical errors:
-Line 16: "pastes" → "parasites"
-Line 38-39: "mean intensity...is found to be" → awkward phrasing; use "was"
-Line 64: "Some of the lakes" → "Several lakes" (more formal)
-Line 73: "accounts 90%" → "accounts for 90%"

enhance your discussion with following:

Author response: the discussion was enhanced by using the suggested and other resources

Khormi, M.A., Emeish, W.F.A., Alghamdi, A.A.A., Fawaz, M,M.,Al-Amgad, Z., Abdelsalam, M., Al-Megrin, W, A.,Bakry, K, A., 2025. Pathophysiological impact of *Euclinostomum heterostomum* infection in Nile tilapia (*Oreochromis niloticus*): a multidisciplinary investigation revealing immune-oxidative stress interactions. *Aquacult Int* **33**, 524 (2025). <https://doi.org/10.1007/s10499-025-02205-3>

Atwah, M.A., Khormi, M.A., Abdelbaky, A.A., Abd El Megeed, O, H., Aborasain, A, M., Salem, M,A.,Soliman, M.A., Khalefa, H, S., Abd-El Hafeez, H, H., Abdelsalam, M., *2025.* Multifaceted analysis of cymothoids parasitism, environmental stressors and heavy metal bioaccumulation dynamics in mediterranean farmed fish in Egypt. *Aquacult Int* **33**, 354, https://doi.org/10.1007/s10499-025-02037-1

Attia, M.M., Abdelsalam, M., Mohamed, H.I., Shadidizaji, A., Soliman, A., W., Warda, W., 2025. *Punica granatum* extract demonstrates antiparasitic effects against *Caligus clemensi* through in silico and in vitro studies. *Sci Rep* **15**, 35005, https://doi.org/10.1038/s41598-025-19529-y

Reviewer #3: The authors have important information. However, the way the article is presented does not meet minimum quality standards. I include many observations in the document. The authors should review the document thoroughly.

Reviewer #4: Thank you for the opportunity to review this manuscript. The topic is important and highly relevant, particularly given the need for more research from regions such as Ethiopia, where local data can substantially improve global understanding. The effort invested in this study is evident, and with further refinement the manuscript has the potential to make a valuable contribution.

Authors response:
Below I outline several areas where improvements would strengthen the clarity, scientific quality, and overall impact of the work. The manuscript would benefit from substantial improvement in both English expression and scientific writing. At present, the narrative is difficult to follow, and key messages become obscured by issues of organisation and clarity.
A more structured, focused, and concise writing style is strongly recommended.
Lines 13–14:
Please clarify whether you are referring to 200 species of marine fish, freshwater fish, or both. This distinction is important for understanding the ecological context of the study and the relevance of the lake system.

Authors response: Most of the fish species in Ethiopia are freshwater fish but their also marine fishes. It is updated in the manuscript
Lines 12–17: It would strengthen the introduction to briefly outline the significance of the Lake. A concise sentence or two on its ecological value, socio-economic importance, or role in local fisheries would help readers unfamiliar with the region appreciate why this research matters.

Authors response: the socioeconomic importance of the lake Hawassa was included in the manuscript.
Lines 18–25: Please specify the number of fish examined and list the species included in the study. Clear reporting of sample size and species composition is essential for evaluating the validity and representativeness of the findings.

Authors response: the overall sample size is, 265;

1. *O. niloticus* (tilapia), n=135,
2. *B. intermedius* *(‘nech asa’*) also known as *Labeobarbus intermedius*, n=87,
3. *C. gariepinus* (African catfish), n=43.

Lines 27–28: The information provided here relates to study procedures and should be relocated to the Methodology section. This will improve the organisation and scientific flow of the manuscript.

Authors response: ok. The manuscript updated accordingly
Lines 27–39: The reporting in this section requires substantial reorganisation for clarity and scientific accuracy.
-Dactylogyrus is not an internal parasite. Please correct its placement. Dactylogyrus spp. are monogenean ectoparasites located on gills and should not be grouped with internal helminths.
-For scientific clarity, this whole section would be much stronger if reorganised by major taxonomic groups, rather than by host species. Grouping the results this way will make the ecological patterns easier to follow and will align with standard parasitological reporting practices.

A suggested structure is:
o Nematodes (e.g., Contracaecum)
o Digenean trematodes (e.g., Clinostomum, Euclinostomum, Diplostomum, encysted metacercariae)
o Monogeneans (Dactylogyrus)
o Myxozoans (Myxobolus spp.)
o Crustacean ectoparasites (Argulus, Dolops)
o Hirudinea (leeches)

Authors: The suggestions were included in the updated manuscript.
-The sentence listing sample sizes (Oreochromis niloticus n=135, Clarias gariepinus n=87, Barbus intermedius n=43) should appear at the beginning of the Results section or in the Methods, not within the middle of the parasite description.

Author response: the updated manuscript is provided with correct structure.
-The overall infection rate (88.30%, n=234) is useful, but please clarify:
o Infection versus infestation terminology
o Whether the 1748 parasites represent total count or mean counts per species

Authors response: from 265 fish sampled 235 of them were affected with one or more internal or external parasites. The total 1748 parasites are collected ad counted from all positive fishes

o Whether prevalence and intensity values are calculated per parasite taxon or per host species
Maybe you could write it something like this, please checj for accuracy: A total of 265 fish were examined, including Oreochromis niloticus (n = 135), Clarias gariepinus (n = 87), and Barbus intermedius (n = 43), with an overall infection/infestation rate of 88.3% (n = 234). In total, 1,748 parasites were collected and classified into nematodes, digenean trematodes, monogeneans, myxozoans, crustacean ectoparasites, and leeches. The most common nematodes were Contracaecum spp., particularly prevalent in C. gariepinus (50.57%). Digenean trematodes included Clinostomum, Euclinostomum, Diplostomum, and encysted metacercariae, with the latter reaching 68.97% in C. gariepinus. Monogenean infections with Dactylogyrus spp. occurred mainly in O. niloticus (28.15%). Myxobolus spp., leeches, Argulus, and Dolops species were also recorded across hosts. Mean parasite intensity was 8.59 in B. intermedius, 7.92 in C. gariepinus, and 6.79 in O. niloticus, while mean abundance was 7.40, 7.37, and 5.84, respectively.

Authors response: The suggestion accepted in good look, Thank you

Lines 99–116 and elsewhere:
Please ensure that “spp.” is not italicised throughout the manuscript. Only the genus and species names should appear in italics (e.g., Contracaecum spp.), while the abbreviation “spp.” always remains in regular font.

Authors response: The taxonomy of the parasite corrected in the manuscript

Lines 117 to 118: please provide a reference.

Authors response: ok, we have added relevant reference

Lines 129–130: Please clarify whether this statement refers specifically to the situation in Ethiopia. If it does not relate directly to Ethiopia or is not supported by local data, I recommend deleting the sentence to maintain accuracy and avoid overgeneralisation.

Authors response: ok. We have corrected the wording

Lines 129–142: You may also wish to consider the impacts of parasites on fish behaviour, as this is an increasingly recognised aspect of fish health and ecology. Behavioural alterations caused by parasitic infections can influence feeding, predator avoidance, reproduction, and overall survival, thereby affecting population dynamics and fisheries productivity. Including a brief acknowledgement of this dimension would enrich the Discussion and provide a more holistic interpretation of your findings. If relevant, you may consult recent literature on parasite-induced behavioural changes in fish for additional context. For example:
Author response: OK, thanks, we have used the suggested references to enhance our discussion

Animals 2024 Vol. 14 Issue 9 Pages 1315
Applied Animal Behaviour Science 2022 Pages 105722
Australian Freshwater Sciences Society. Waurn Ponds, 1-4 December. 2019
Behaviour 2012 Vol. 149 Issue 6 Pages 601-622
Int J Parasitol 1995 Vol. 25 Issue 12 Pages 1371-1383
J Exp Biol 2013 Vol. 216 Issue 1 Pages 18-26
L. Rogers, E. Sales, S. Shamsi, R. K. Kopf and R. Freire
Parasitol Res 2021 Vol. 120 Issue 2 Pages 515-523

Line 143: I recommend replacing the term “developing country” with “low-income country” when referring to Ethiopia. The phrase “developing country” can be overly broad and does not reflect the many areas in which Ethiopia is already well-developed culturally, scientifically, and economically. Using “low-income country” provides clearer, more precise context and aligns with current international terminology.

Authors response: ok. We have replaced the term ‘developing country’ with ‘low and middle-income country’.

Tables:
Please add horizontal lines and clearer structural formatting to the tables to improve readability. At present, the data are difficult to follow, and adding lines (for example, separating header rows from data rows and delineating major sections) will make the tables much easier for readers to interpret. Consistent alignment of columns and spacing will further enhance clarity.

Authors response: the tables are clearly separated by lines.

Discussion
Please expand the Discussion to address the limitations of using visual inspection alone for parasite detection. While visual examination is acceptable and widely used in field-based parasitology studies, it has well-recognised limitations. Without applying digestion methods (see Canadian Journal of Fisheries and Aquatic Sciences 1986 Vol. 43 Issue 7 Pages 1312-1317) or incubation techniques (see International Journal of Food Microbiology 2016 Vol. 227 Pages 13-16), it is likely that some small or deeply encysted parasites, early developmental stages, or low-intensity infections were not detected. Acknowledging this limitation is important for transparency and will actually strengthen your study. It highlights that the parasite diversity and prevalence in Lake Hawassa may be even higher than reported, reinforcing the significance of your findings and the need for further, more sensitive diagnostic approaches in future research.

Authors response: the limitation of the current finding is clearly stated

Discussion (lines 315-317, 329 to 331 and more):
Some parts of the Discussion section repeat information already presented in the Results. These repeated descriptions should be removed to avoid redundancy. The Discussion should focus on interpreting the findings, comparing them with previous studies, exploring implications, and acknowledging limitations. Please revise by deleting the sections that duplicate the Results, including lines XXX, and ensure the Discussion provides insight rather than repetition.

Authors response: Thank you. We have addressed the issues according to the reviewers’ comments.

Lines 428–431:
Clinostomum is a particularly important parasite, and the Discussion would benefit from addressing aspects beyond simple prevalence comparisons. I encourage the authors to expand this section by briefly discussing:
• Key features of the life cycle (including the role of snails, fish, and piscivorous birds),
• Its pathogenic significance, especially its occurrence in the skull, musculature, and other critical tissues,
• Implications for fish health, fish welfare, and potential zoonotic considerations, and
• Any ecological or economic consequences relevant to Lake Hawassa or similar systems.
Including a short synthesis similar to discussions found in recent studies on Clinostomum—for example, research addressing tissue localisation, pathological effects, and host–parasite interactions—would enrich the manuscript and provide readers with a more comprehensive understanding of the importance of this genus. Some articles that authors may find useful to read are suggested below:
Aquaculture 2021 Vol. 539 Pages 736584 DOI: <https://doi.org/10.1016/j.aquaculture.2021.736584>
Journal of helminthology 2020 Vol. 94
Korean J Parasitol 2019 Vol. 57 Issue 2 Pages 175-177
Parasitology Research 2021 Vol. 120 Pages 2793-2803
Revue de Médecine Vétérinaire 2018 Vol. 7 Pages 147-151
Syst Parasitol 2018 Vol. 95 Issue 6 Pages 543-566
Line 445:
The co-occurrence of Clinostomum and Euclinostomum is an interesting and noteworthy finding. You may wish to highlight this more clearly, as such combinations can offer insights into shared intermediate hosts, environmental conditions, or ecological overlap. The authors may also find relevant comparisons in studies from India (Parasitology Research 2022 Vol. 121 Issue 11 Pages 3083-3089) where similar patterns have been reported. Including a brief mention of that work could help contextualise your findings and strengthen the Discussion.

Figures:
The figures require substantial improvement in multiple aspects. I strongly recommend that the authors revise them with guidance from a senior researcher experienced in parasitological imaging and figure preparation. At present, several issues affect clarity and scientific value:
• In some images, it is unclear what the arrows are pointing to. Please ensure arrows clearly indicate the diagnostic structures or lesions of interest.
• Some figures contain multiple images but lack labels (e.g., A, B, C) or accompanying explanations, making it impossible for the reader to understand what each panel represents.
• Scale bars and magnification details are missing or inconsistent.
• Image quality varies considerably, and some photographs are too dark, blurry, or low resolution for reliable interpretation.
Improving these elements will significantly enhance the scientific credibility of the manuscript. Clear, well-labelled, high-quality figures are essential for parasitology work, particularly when morphological identification is involved.

6. PLOS authors have the option to publish the peer review history of their article ([what does this mean?](https://track.editorialmanager.com/CL0/https:%2F%2Fjournals.plos.org%2Fplosone%2Fs%2Feditorial-and-peer-review-process%23loc-peer-review-history/1/010f019abede830e-7a74245e-0f28-4b00-a4c0-64d8ce610dbb-000000/caJQBQ9QwdYzSDtBZ0J-liu82WYmItONRLeF7ZeYpTY=238)). If published, this will include your full peer review and any attached files.


If you choose “no”, your identity will remain anonymous but your review may still be made public.


**Do you want your identity to be public for this peer review?** For information about this choice, including consent withdrawal, please see our [Privacy Policy](https://track.editorialmanager.com/CL0/https:%2F%2Fwww.plos.org%2Fprivacy-policy/1/010f019abede830e-7a74245e-0f28-4b00-a4c0-64d8ce610dbb-000000/my-dLwMp1U86NMT7VYWSfeLsvwv5ZYYxkkWpAHSjQLY=238).

Reviewer #1: No

Reviewer #2: No

Reviewer #3: **Yes:** José Alberto Iannacone Oliver, Ph.D.

Reviewer #4: No

Dear Author

I sincerely appreciate the opportunity to review this article, which is titled:

Prevalence of fish parasites from Lake Hawassa, Ethiopia

Manuscript Number: PONE-D-25-59453

That is a good, clear title, but it could be significantly improved by adding more specificity about the hosts and the study's key finding, which increases its impact. This is a great starting point for a manuscript, but there are several points that require clarification or correction to meet high scientific standards.

Here is a point-by-point scientific review:

The manuscript is efficient, but the lack of citations for key statistics and claims is a serious issue that must be addressed before submission. You also need to correct the simple grammar and spelling errors noted in Ms. Strengthening the opening statements, maintaining scientific accuracy, and attending to the requirement for citations are the main objectives of this review.

1- Phrasing and Grammar (Line 13):

for scientific writing, the phrase "Fish provides... to human being" is a little too casual.
Author response: Ok, It is revised to be scientifically sound

2- Use common plural or collective terms. "Fish provides quality protein, various vitamins and minerals essential for human consumption (or humans)," is the recommendation.

Author response: Ok, the suggestion is well appreciated and sincerely accepted.

Errors in Grammar and Typography (Lines 15–17): Required: Fix both mistakes.

There are two spelling and grammar mistakes:

A-"The parasite fish of Lake Hawassa are..." (L15)

B-"...limited reports on the type of pastes..." (L17):

Author response: we have corrected both mistakes

The statistic "More than17 % of animal protein for global population is obtained from fish" is accurate and needs to be backed up by a recent, trustworthy source (such as an FAO report). Required: add this assertion, include a citation.
Author response: We have referenced FAO and regional study,

FAO, 2018. Food and Agriculture Organization of the United Nations, *The State of World Fisheries and Aquaculture 2018—Meeting the Sustainable Development Goals*; FAO: Rome, Italy, 2018.

Obiero, K., Meulenbroek, P., Drexler, S., Dagne, A., Akoll, P., Odong,R., Kaunda-Arara, B., Waidbacher, H.,2019. The Contribution of Fish to Food and Nutrition Security in Eastern Africa: Emerging Trends and Future Outlooks, <https://doi.org/10.3390/su11061636>

Given that your study only looks at one lake (Hawassa), the assertion that fish parasites are the "main cause of the significant threat to the fish biodiversity of Ethiopia's freshwater islands" is very strong and needs immediate, specific evidence or citation. Another non-standard term that needs to be defined is "freshwater islands" (e.g., freshwater ecosystems or specific lakes). Needed Action: Modify the assertion or give an immediate explanation. Concentrate the claim on commercial species or aquaculture if those are the main threats.

Author response: While we have explained the statement with recent reference our intention was to provide global scenario in the background instead of only Ethiopian context.

We have also cited

Power, C., Shirakashi, S., Bott, N.J., Nowak, B.F. (2025). Parasites in Aquaculture. In: Smit, N.J., Sures, B. (eds) Aquatic Parasitology: Ecological and Environmental Concepts and Implications of Marine and Freshwater Parasites. Springer, Cham. <https://doi.org/10.1007/978-3-031-83903-0_23> , to explain the issue more

Methodes;

Clarify these points

Sampling Location & Method: Where and how were the fish collected?

Sample Size (N):

What was the total number of fish examined?

Taxonomic Keys: Name the primary identification guides used.

Statistical Detail: Specify the significance level (e.g., P < 0.05) and the software used.

Author response: in methodology we have updated that the samples were collected from different fishing centres/corners of the lake during repeated visiting and the researcher was one responsible for all the data collected and analysed here. Fishermen’s have supported us as they have different meshes.

**Results:**

The numbered of fish samples (N) n=135 (*Oreochromis niloticus*), n=87 (*Clarias gariepinus*), and n=43 (*Barbus intermedius*). **Total N=265**.**Correction:** The text states the overall infection rate is 88.30% (n=234). This indicates that **N=265** fish were examined (135+87+43=265), and 234 were infected. This number (265) should be explicitly stated as the total Sample Size for clarity.

Author response: 265 is the total sample size, Ok. We have explained it in the updated manuscript

-The design of the Study and Sampling Method (Lines 169–173);
- Contradictory Sampling Terminology (Line 171): Critique: In conventional scientific methodology, the term "systematic random sampling technique" is usually ambiguous. Simple random sampling, in which each fish has an equal chance of being selected, and systematic sampling, such as every numbered of fish, are two different approaches.
- Could be rewriting the precise process that is employed in the field.

Author response: we have defined the sampling technique and methodology in the updated manuscript

Have you taken random subsamples from the entire catch? Use either "Simple Random Sampling" or "Random Subsampling." Employ "Systematic Sampling." I suggest precisely define the procedure.

Author response: Systematic Sampling was employed only to select the fish from the net, for example every 10^th^ from the cage/net

Duration Clarity (Line 173):

Remark: The sentence "The assessment was conducted in all for over 10 months" is grammatically incorrect. Correct to: Make the wording simpler." The assessment was conducted over a period of ten months, from October 2023 to July 2024," is the recommendation.

Author response: ok, we have applied in the updated manuscript

3. Justification of the Study Design (Lines 169–170):

The 10-month period may suggest seasonal comparisons (longitudinal element), which are frequently required in parasitology, even though a "cross-sectional study" is suitable for prevalence at a specific moment in time. I suggest rewriting the sentence to account for seasonal variations in prevalence. You should indicate that the cross-sectional data collection was repeated monthly if you are analyzing data by season or month. Reiterate that the goal is to provide a thorough overall analysis if all 10 months of data are combined. Months of data are pooled, reaffirm that the purpose is to provide a comprehensive overall prevalence estimate for the study period Population under Study (Lines 174–183).

Author response: the ten months data collection period is normal data collection period for simple random sampling, duration of almost one year. Our intention was not to include seasonal variation, but we have focused on parasite epidemiology.

- (Lines 181–183): The exact percentages of total production that the primary fish species (*O. niloticus*, *C. gariepinus,* &L. intermedius) contributed are based on a 1996 reference (LFDP, 1996). The ecosystem of Lake Hawassa has been severely impacted, and it is very likely that community structure has changed over the past thirty years. Updated recent reference to confirm or update these proportions (within the last ten years). Add recent references.

Authors response: Ok, <https://doi.org/10.1016/j.heliyon.2023.e18661> is added to describe the regional importance of those fishes

Wubie, A., Teshome, K., Tesfaye, G., 2023. Potential fish yield and biology of some fish in Lake Hertale, Afar regional state, Ethiopia. Heliyon, 9(8):e18661. doi: 10.1016/j.heliyon. 2023.e18661.

-Convention for Scientific Naming (Lines 177–179): The three large fish species and the three small fish species (*Barbus paludinosis, Aplocheilichthys antinorii*, and *Gara quadrimaculata*) are identified by their scientific names. The genus and species name must be written out completely when referring to a species for the first time (e.g., *Oreochromis niloticus*). The genus abbreviation (*O. niloticus*) may be used in later applications. Required make sure the names of all other species (including the minor species mentioned here) adhere to this rule throughout the manuscript but use the abbreviation correctly for *O. niloticus* and *C. gariepinus* later in line (179).

Authors response: the full species name was initially defined in abstract, line 19-20, we have updated it accordingly.

Lines 194-209; Categorization and Sampling
1. Vague Sampling Methodology (Line 194): It is not enough to say that "randomly picked those fishes from different sampling points."
- Required: Describe the precise capture technique (such as electrofishing, cast nets, or gill nets) and how bias was prevented at each sampling location by ensuring true random selection (such as systematic selection of size classes).

Authors response: We have used simple random sampling but systematically to every day to achieve the calculated sample size, as you have suggested. We have updated in the methodology
2. Lines 195-199: Measurement Consistency Only text is mentioned for the final measurement and categorization (L198-199), despite the fact that both Total Length, and Standard Length were initially measured (L195-196).
**- Action Needed:** State clearly which length measurement (Total Length or Standard Length) was used for all analysis and categorization, and why, if both were initially recorded.

Authors response: Although both Total Length (TL) and Standard Length (SL) were initially recorded, standard length (SL), was used for all statical analysis and size categorization in this study. SL was selected because it provides a more consistent and reliable measure of body size, excluding the caudal fin, which can be subject to damage, regeneration, or measurement variability. Using SL therefore minimizes measurement bias and improves comparability among individuals. Total Length was recorded for descriptive purposes and comparison with previous studies.

**Line 197:** **Clarity on Sex Determination Timing** The text implies initial assessment of sex, but confirmation is done "after dissection. **Action Needed:** Rephrase for clarity, e.g., "The sex of each fish was tentatively assessed externally and confirmed post-dissection by noting the presence of testes or ovaries (Imam and Dewu, 2010)."

**Authors response:** ok, we have updated accordingly

**Lines 200-206:** **Lack of Justification for Categorical Breakpoints** While classifying fish by size is crucial (L207-209), the specific breakpoints for Length (Class I, II, III) and Weight (Class I, II, III) for the three species are only referenced to an external paper (Tesfaye et al., 2020). **Could be** Provide a brief biological justification for why these specific size ranges (e.g., 50 cm and 75 cm for *C. gariepinus* length were chosen as breakpoints, beyond citing the reference. Are they linked to sexual maturity or dietary shifts in this specific ecosystem?

**Authors response:** The selected size breakpoints for length and weight classes were not arbitrarily adopted from Tesfaye et al. (2020) but reflect biologically meaningful transitions in growth and life-history stages. For example, in Clarias gariepinus, the length thresholds of 50 cm and 75 cm correspond approximately to ontogenetic stages associated with sexual maturation and trophic shifts. Individuals below 50 cm are typically juveniles or subadults with developing gonads and a diet dominated by invertebrates and small prey. Fish between 50–75 cm generally represents sexually mature adults, while individuals exceeding 75 cm are fully mature, larger-bodied predators with broader dietary niches and higher trophic positioning. These ontogenetic changes influence exposure risk to parasites through altered feeding behaviour, habitat use, and immune competence. Therefore, the selected size classes reflect biologically relevant developmental stages that may affect parasite acquisition dynamics within this ecosystem, rather than being purely arbitrary statistical categories.

Lines 200–206: The categorical breakpoints are not justified: The precise breakpoints for Length (Class I, II, III) and Weight (Class I, II, III) for the three species are only cited in an external paper (Tesfaye et al., 2020), despite the fact that size classification of fish is essential (L207-209).

Needed Action: Beyond simply mentioning the source, give a succinct biological explanation for the selection of these particular size ranges as breakpoints (e.g., 50 cm and75 cm for *C. gariepinus* length). Are they associated with changes in diet or sexual maturity in this particular ecosystem?

**Authors response:** The selected size breakpoints for length and weight classes were not arbitrarily adopted from Tesfaye et al. (2020) but reflect biologically meaningful transitions in growth and life-history stages. For example, in Clarias gariepinus, the length thresholds of 50 cm and 75 cm correspond approximately to ontogenetic stages associated with sexual maturation and trophic shifts. Individuals below 50 cm are typically juveniles or subadults with developing gonads and a diet dominated by invertebrates and small prey. Fish between 50–75 cm generally represents sexually mature adults, while individuals exceeding 75 cm are fully mature, larger-bodied predators with broader dietary niches and higher trophic positioning. These ontogenetic changes influence exposure risk to parasites through altered feeding behaviour, habitat use, and immune competence. Therefore, the selected size classes reflect biologically relevant developmental stages that may affect parasite acquisition dynamics within this ecosystem, rather than being purely arbitrary statistical categories.

- Lines 210-242: Antemortem and Postmortem Identification of Parasite

- Clarify the method in Transport/Euthanasia (Lines 212-213): The fish were "examined alive" (L211) and then transported "in an icebox" (L212). The icebox suggests chilling or freezing, which would kill the fish or severely inhibit parasite motility. **Action Needed**: Specify the exact conditions: Were the fish euthanized immediately after the initial examination and before transport, or were they transported alive in a cooled, aerated environment?

If an icebox was used, state that the fish were immediately sacrificed (euthanized) upon collection to prevent autolysis and preserve parasites.

Authors response: Fish were initially examined alive for external abnormalities immediately upon capture. They were then humanely euthanized at the sampling site and placed in an icebox containing crushed ice for transport to the laboratory. The use of an icebox was solely to retard autolysis and preserve parasite integrity for accurate postmortem examination. Fish were not transported alive.

Line 216: Ectoparasite Harvesting Solution The text mentions harvesting external parasites into "physiological salt solution" and "fresh water."

**Action Needed:** Standardize the solution. Fresh water is highly likely to cause osmotic shock and rupture delicate parasites (like protozoans or monogeneans), making identification difficult. Specify the *concentration* of the physiological saline

**Author response:** Ectoparasites collected from the skin, fins, and gills were immediately transferred into 0.9% physiological saline solution (NaCl) to maintain osmotic balance and preserve parasite morphology during examination. The use of isotonic saline prevented osmotic shock that could distort or rupture delicate protozoans and monogeneans. No fresh water was used during parasite harvesting or processing.

**Line 227:** **Parasite Clearing Concentration (**Using 99% lactophenol for 24 hours to clear the parasite cuticle for identification may be too aggressive and could destroy internal structures. **Correct:** Justify why use of 99% lactophenol concentration. Often, a 70% alcohol fixation followed by a temporary mount in a standard clearing agent is preferred, or a gentler, more diluted concentration is used for prolonged periods.

Authors response: Parasite specimens were first fixed in 70% ethanol to preserve internal morphology. For identification, selected specimens were temporarily mounted and cleared in lactophenol for approximately 24 hours to enhance visualization of sclerotized and internal structures.

Furthermore, I recommend you focus on revising the transport conditions and correcting the severe temperature error in the digestion protocol

-**Lines: 230 and 233;** Major **Typographical Error in Digestion Temperature;** the artificial digestion temperatures of 420C and 400C are physically and biologically impossible. **Correction:** These should be corrected to biological temperatures, likely 42C and 40C, respectively. This is a critical error in the methodology that must be fixed.

Authors response: it is typo error, we have corrected in the updated manuscript, the maximum digestion temperature is 42^0^C.

-**Line 234:** **Insufficient Digestion Time;** Digesting 100g of fish flesh at 40C for only 20 minutes is highly unlikely to achieve "fully homogenise the tissue sample."

- **Correction:** Justify the 20-minute duration. Typical tissue digestion protocols for larval parasite recovery often require 2 to 6 hours, sometimes with intermediate steps or agitation, to ensure maximum yield. If a very short time was used, the recovery efficiency should be discussed or validated.

Authors response: Ok. We have updated the digestion protocol

-**Lines 228, 236:** **Target of Digestion**

**-**The digestion method is used to harvest "any encysted larva in the fish muscle" (L228), and the straining retained material "was examined for any larvae of nematode" (L236).

**Ok.** Thanks

**Could be corrected:** The subsequent morphological checks (L240-242) clearly point to Anisakid nematodes (like *Anisakis* or *Contracaecum*). State that the method was specifically optimized for the recovery of infective larval nematodes (e.g., L3 stages of Anisakidae) from the musculature is relevant here to show why this muscle digestion is necessary.

Authors response: we have used the suggestion and updated accordingly

I recommend: How common, how many and how strong (Lines 267–274)
- Ecological metrics don't have any measures of dispersion (Lines 270–272): Critique: This is the biggest problem. Mean Intensity (MI) and Mean Abundance (MA) are ecological statistics that change a lot. They are given as point estimates, with a minimum of 5.37 and a maximum of 8.08. Without a measure of variability, these numbers don't mean anything statistically or can be compared.

Action Needed: You need to report both Mean Intensity and Mean Abundance with a measure of dispersion, which is usually the Standard Deviation (SD) or the Standard Error (SE).
- Suggestion: Show the data for each of the three fish species as MI ± SD and MA ± SD.

Authors response: we have updated, Thank you

Unclear Minimum/Maximum Reporting (Lines 270–272):
Critique: The minimum and maximum values for MI and MA are presented across species without comprehensive context. It is clear that *C. gariepinus* had the lowest MI (5.37) and *B. intermedius* had the highest (8.08), but this should be made clear and checked against the reported values.
What needs to be done: Clearly state: "*C. gariepinus* had the lowest mean intensity (MI = 5.37 pm SD), and *B. intermedius* had the highest (MI = 8.08 pm SD)."

authors response: Thank you, we have included the suggestion

Lines 270–272: Ignoring Dispersion Measures for Ecological Metrics

-This is the biggest error. Ecological statistics that vary greatly include Mean Intensity (MI) and Mean Abundance (MA). They are given as point estimates, with a minimum of 5.37 and a maximum of 8.08. Without a measure of variability, these metrics lack statistical significance and comparability.
-Action Required: A measure of dispersion, usually the Standard Deviation (SD) or the Standard Error (SE), must be included with both Mean Intensity and Mean Abundance. I Suggested: for each of the three fish species, display the data as MI -SD and MA -SD.

authors response: Thank you, we have included the suggestion

Lines: 273–274: Reference to the Critical Table. Could be corrected the text in (Table 1) specifically cites for the statistical data and makes reference to the Chi-square test result.
- The raw counts for prevalence (infected fish / total fish) for each species, which were used to create the chi 2 statistic, must be included in Table 1. The sentence that introduces the statistic must come before or right after the table.

Ok. We have updated the manucrip

Line (274): Reporting P-Values, while it is acceptable to report P>0.05, it is preferable to report the precise $P$-value for non-significant results, unless it is extremely large (e.g., P = 0.123).
- Required correction: Substitute the actual P-value for P>0.05, if it is available.

Authors response: we have updated accordingly

Line 289: Typographical Error in Anatomical Location

Remark: The text incorrectly identifies the *Clinostomum* infection site as "branchial cavity, pericardial cavity, premium and ocular cavity." Most likely, the word "premium" is misspelled.
-Required correction: To match the exact description later given in Table 3, this should be changed to a standard anatomical term, most likely "peritoneum," "pharyngeal muscles," or "cranium and pharyngeal teeth"

Authors response: we have updated the manuscript accordingly, thanks

Line (308). Generally

All figured contain photos are undistinct and very low quality, replace the Fig. by more distinct photos

All figures could be distinct morphological characters to confirmed the key of parasites identifications

*Authors response: we have improved the image quality and the indicators.*

Internal Figure Reference Error in Line 290: Remark: The text notes that *Euclinostomum* is common and that it affects different organs (figure 3). There was confusion because Figure 3 was already cited for *Clinostomum* (Line 288). Required correction: to guarantee that figures are uniquely cited in the text, confirm and update the figure reference for *Euclinostomum* (for example, if Figure 4 depicts *Euclinostomum,* then refer to Figure 4 here).

Authors response: figure 3 depicts *Clinostomum* while figure 4 indicates *Euclinostomum*
-Misspelling in Parasite Name (Lines 293 and 299):
Remark: The parasite is frequently spelled incorrectly as "Contracecum" (Line 299) and *"Contacaecum"* (e.g., Line 293), throughout the manuscript, the spelling "*Contracaecum"* needs to be corrected.

Authors response: We have corrected the parasite name in the manuscript

Line 295: Inaccurate dominant helminth identification: "Digenean encysted metacercaria" (68.97% prevalence) is the term used in the text to describe the dominant parasite in *C. gariepinus*. Since *O. niloticus* was found to contain certain trematodes (*Clinostomum, Euclinostomum*), the inability to identify the dominant digenean in *C. gariepinus* at least to genus

Level indicates challenging morphology or insufficient analysis.
- Could be explaining in brief why this dominant parasite was unable to be identified down to the genus level (for example, "due to heavy encapsulation preventing morphological observation," or "given the high intensity and difficulty of differentiation, these were categorized generally as Digenean metacercariae").

Authors response: The dominant helminth in Clarias gariepinus was recorded as digenean encysted metacercaria (68.97% prevalence). Identification to genus level was not possible due to heavy encapsulation within host tissue, which obscured key morphological features required for precise taxonomic determination. Given the high intensity and overlapping morphology of the cysts, these parasites were conservatively categorized as digenean metacercariae. In contrast, O. niloticus harbored trematodes such as Clinostomum and Euclinostomum, which were identifiable based on well-preserved sclerotized structures.

-Lines 297–298: Methodology Placement: Your calculation method is clarified by the statement that "prevalence report is calculated considering multiple parasite infestation from single host..."
-Required correction: Transfer this explanation to the Methods section, where you specify how prevalence, mean intensity, and mean abundance are calculated (more precisely, in the 2.5.3 Data Analysis sub-section).

Author response: Thank you for the suggestion. We have moved the suggested text to the Methods section accordingly.
