# Supplementary material for: Prevalence of fish parasites from Lake Hawassa, Ethiopia
Source: PLoS One. 2026 Sep 11;21(9):e0357816. doi: 10.1371/journal.pone.0357816 (PMC13568496; doi:10.1371/journal.pone.0357816)
Supplement: Supplementary file 6 [file pone.0357816.s007.docx]

PONE-D-25-59453R1

Prevalence of fish parasites from Lake Hawassa, Ethiopia

PLOS One

Dear Dr. EFA,

Thank you for submitting your manuscript to PLOS ONE. After careful consideration, we feel that it has merit but does not fully meet PLOS ONE’s publication criteria as it currently stands. Therefore, we invite you to submit a revised version of the manuscript that addresses the points raised during the review process.

Please submit your revised manuscript by May 23 2026 11:59PM. If you will need more time than this to complete your revisions, please reply to this message or contact the journal office at [plosone@plos.org](mailto:plosone@plos.org). When you're ready to submit your revision, log on to <https://www.editorialmanager.com/pone/> and select the 'Submissions Needing Revision' folder to locate your manuscript file.

Please include the following items when submitting your revised manuscript:

- A letter that responds to each point raised by the academic editor and reviewer(s). You should upload this letter as a separate file labeled 'Response to Reviewers'.
- A marked-up copy of your manuscript that highlights changes made to the original version. You should upload this as a separate file labeled 'Revised Manuscript with Track Changes'.
- An unmarked version of your revised paper without tracked changes. You should upload this as a separate file labeled 'Manuscript'.

If you would like to make changes to your financial disclosure, please include your updated statement in your cover letter. Guidelines for resubmitting your figure files are available below the reviewer comments at the end of this letter.

If applicable, we recommend that you deposit your laboratory protocols in [protocols.io](http://protocols.io/) to enhance the reproducibility of your results. Protocols.io assigns your protocol its own identifier (DOI) so that it can be cited independently in the future. For instructions see: [https://journals.plos.org/plosone/s/submission-guidelines#loc-laboratory-protocols](https://track.editorialmanager.com/CL0/https:%2F%2Fjournals.plos.org%2Fplosone%2Fs%2Fsubmission-guidelines%23loc-laboratory-protocols/1/010f019d6c109bd5-d5d3a533-6f05-4834-8fc7-100692d283e5-000000/J0aAK5vAoJ8XakHxruMyCnRM6SEVJJTOW_h0UVc7Kbc=258). Additionally, PLOS ONE offers an option for publishing peer-reviewed Lab Protocol articles, which describe protocols hosted on [protocols.io](http://protocols.io/). Read more information on sharing protocols at [https://plos.org/protocols?utm_medium=editorial-email&utm_source=authorletters&utm_campaign=protocols](https://track.editorialmanager.com/CL0/https:%2F%2Fplos.org%2Fprotocols%3Futm_medium=editorial-email%26utm_source=authorletters%26utm_campaign=protocols/1/010f019d6c109bd5-d5d3a533-6f05-4834-8fc7-100692d283e5-000000/W9uHFkN61DrrWFtDRJpQafavrUCNSy_l-OtHbaVOIQ0=258).

As the corresponding author, your ORCID iD is verified in the submission system and will appear in the published article. PLOS supports the use of ORCID, and we encourage all coauthors to register for an ORCID iD and use it as well. Please encourage your coauthors to verify their ORCID iD within the submission system before final acceptance, as unverified ORCID iDs will not appear in the published article. *Only* the individual author can complete the verification step; PLOS staff *cannot* verify ORCID iDs on behalf of authors.

We look forward to receiving your revised manuscript.

Kind regards,

Clement Ameh Yaro, Ph.D

Academic Editor

PLOS One

Journal Requirements:

If the reviewer comments include a recommendation to cite specific previously published works, please review and evaluate these publications to determine whether they are relevant and should be cited. There is no requirement to cite these works unless the editor has indicated otherwise. 


[Note: HTML markup is below. Please do not edit.] Reviewers' comments:

Reviewer's Responses to Questions

**Comments to the Author**

1. If the authors have adequately addressed your comments raised in a previous round of review and you feel that this manuscript is now acceptable for publication, you may indicate that here to bypass the “Comments to the Author” section, enter your conflict of interest statement in the “Confidential to Editor” section, and submit your "Accept" recommendation.

Reviewer #1: All comments have been addressed

Reviewer #2: (No Response)

Reviewer #3: (No Response)

Reviewer #4: (No Response)

2. Is the manuscript technically sound, and do the data support the conclusions?

The manuscript must describe a technically sound piece of scientific research with data that supports the conclusions. Experiments must have been conducted rigorously, with appropriate controls, replication, and sample sizes. The conclusions must be drawn appropriately based on the data presented.

Reviewer #1: Yes

Reviewer #2: No

Reviewer #3: Partly

Reviewer #4: No

3. Has the statistical analysis been performed appropriately and rigorously?

Reviewer #1: Yes

Reviewer #2: No

Reviewer #3: No

Reviewer #4: I Don't Know

4. Have the authors made all data underlying the findings in their manuscript fully available?

The [PLOS Data policy](http://track.editorialmanager.com/CL0/http:%2F%2Fwww.plosone.org%2Fstatic%2Fpolicies.action%23sharing/1/010f019d6c109bd5-d5d3a533-6f05-4834-8fc7-100692d283e5-000000/FoghdN59Mh4hwZR_wjbL3gG3mkuXizd2K_NwlPInhso=258) requires authors to make all data underlying the findings described in their manuscript fully available without restriction, with rare exception (please refer to the Data Availability Statement in the manuscript PDF file). The data should be provided as part of the manuscript or its supporting information, or deposited to a public repository. For example, in addition to summary statistics, the data points behind means, medians and variance measures should be available. If there are restrictions on publicly sharing data—e.g. participant privacy or use of data from a third party—those must be specified.

Reviewer #1: Yes

Reviewer #2: No: We have made the data that support this finding available and submit additional file in Excell format.

Reviewer #3: No: We have made the data that support this finding available and submit additional file in Excell format.

Reviewer #4: Yes

5. Is the manuscript presented in an intelligible fashion and written in standard English?

PLOS ONE does not copyedit accepted manuscripts, so the language in submitted articles must be clear, correct, and unambiguous. Any typographical or grammatical errors should be corrected at revision, so please note any specific errors here.

Reviewer #1 and #3: Yes

Reviewer #2 and #4: No:

**We kindly request the reviewer to examine the edits and revisions we have made to the manuscript. We have implemented substantial and rigorous changes throughout the revised version, and we hope this will minimize the need for extensive language editing by the technical team.**

6. Review Comments to the Author

Please use the space provided to explain your answers to the questions above. You may also include additional comments for the author, including concerns about dual publication, research ethics, or publication ethics. (Please upload your review as an attachment if it exceeds 20,000 characters)

Reviewer #1: I would like to thank the authors for their response; the manuscript has been significantly improved by incorporating the suggested changes."

Reviewer #2: This manuscript reports on parasite prevalence, intensity, and abundance across three commercially important fish species from Lake Hawassa, Ethiopia. While the fieldwork addresses a genuine knowledge gap and the dataset has intrinsic value, the manuscript cannot be accepted or sent for major revision in its current form. The combination of critical data inconsistencies, invalid statistical calculations, and a serious research integrity concern collectively disqualify this submission. Rejection is recommended.

Authors response: **We kindly request the reviewer to examine the edits and revisions we have made to the manuscript. We have implemented substantial and rigorous changes throughout the revised version, and we hope this will minimize the need for extensive language editing by the technical team.**

Causes of Rejection:

1. Fundamental Statistical Errors

The sub-group prevalence figures reported throughout Table 2 were calculated using the number of infected fish as the denominator rather than the total number of examined fish. This is a foundational error in prevalence calculation that inflates every sub-group estimate in the manuscript and renders the primary analytical results unreliable.

Authors response: in calculating the prevalence, abundance and mean intensity we have cited and widely used reference El-Shahawy et al., 2017 who also uses the same formula. Also, in sample calculation we have used collective sampling on three commercially important fish species in the area each accounting 135 *O. niloticus*, 87 *B. intermedius* and 43 *C. gariepinus*.

For example, the C. gariepinus sub-analyses use n=81 as the denominator when the correct denominator is n=87. This error is systematic, not isolated, and cannot be corrected editorially, it requires complete recalculation from the raw dataset upward.

**Authors response: As previously indicated, Table 1 presents the overall prevalence. In contrast, Table 2 specifically summarizes data from infected fish only. Therefore, the number of infected fish was intentionally used as the denominator in Table 2, following the established formula and analytical approach described in the manuscript (please refer to Table 2).**

2. Internally Contradictory Numerical Results

Mean intensity values of 5.37 and 8.08 are cited in the Results text (lines 284–285) but appear nowhere in Table 1, which reports 7.92 and 8.59 for the same species. No explanation is offered. The reader cannot determine which figures are correct.

Authors response: **We respectfully request the reviewer to reassess the revised version. The editorial error in lines 284–285 has been corrected, and the figure reported in Table 1 and discussion part is accurate.**
-The chi-square P-value for species-level parasite variation is reported simultaneously as P=0.06 in the text (line 287) and P=0.24 in Table 1. These values lead to different scientific conclusions and cannot both be correct.

**Authors response: We kindly ask again the reviewer to recheck the edits and revisions that have currently been made. We have corrected the editorial error in lines 284–285, and the figure presented in Table 1 is correct.**

The overall prevalence is reported as 88.30% throughout the manuscript but 88.03% in the conclusion (line 565).

Authors response: **We kindly ask again the reviewer to recheck the edits and revisions that have currently been made in the conclusion.** The corrected figure is 234/265=88.30%

-Sample sizes for B. intermedius and C. gariepinus are transposed in the Results section (line 277–278) relative to all other sections, creating fundamental confusion about the study population.

-These are not typographical errors. They reflect insufficient verification of results before submission and undermine confidence in all reported findings.

Authors response: the two species mentioned in the above line is not very large like for O. niloticus and that is why relatively lower number were sampled during random selection. We have reported the exact number we have sampled and explain the selection procedure in methods and justification

The two species mentioned above have smaller population sizes compared to O. niloticus. Consequently, fewer individuals were sampled during random selection. We have reported the exact number of individuals sampled for each species and provided a detailed explanation of the selection procedure in the Methods section, along with the justification for the sample sizes chosen.

5. Absence of Seasonal Analysis Despite a 10-Month Study
The study spans October 2023 to July 2024, covering both wet and dry seasons in the Ethiopian Rift Valley; periods known to differ substantially in water temperature, intermediate host abundance, and parasite transmission dynamics. No temporal or seasonal breakdown of parasite data is presented or discussed. This omission is not a limitation of the study design but of the analysis, and it represents a missed opportunity that significantly reduces the manuscript's scientific contribution and generalizability.
Author response: We have been on field, lab and writing the draft thesis for about 10 months, we did not hypothesised the seasonality of parasite infection/infestation in our justification and we accept the limitation.
6. Additional Concerns Noted for Authors' Consideration Upon Resubmission Elsewhere
-No confidence intervals are reported for any prevalence estimate
-Chi-square tests were applied to cells with expected frequencies below 5; Fisher's exact test is required in these cases
-No multivariate analysis accounting for collinearity between host size variables

Author response: We have reanalysed and provided the update in the revised manuscript.
-The terms infection and infestation are used interchangeably throughout; these are technically distinct and should be applied correctly

Authors response: We have corrected the use of terms in the edited manuscript.
-All microscopy figures lack scale bars — a mandatory requirement in parasitological publications

Authors response: We acknowledge the limitation and correct in other submission
-The Limitations section appears twice with different content (lines 558–561 and 593–596) and must be consolidated

Authors response: We have consolidated the limitation section, now found at the end of the discussions

-A CNN travel article (lines 732–735) is cited as a scientific reference and must be replaced with peer-reviewed literature

Authors response: we have removed the CNN report with peer reviewed reference

-The Discussion is largely a comparison of prevalence percentages across studies without mechanistic explanation. The roles of intermediate host ecology, lake eutrophication, seasonal transmission dynamics, and One Health implications of zoonotic parasites in a population known to consume raw fish should be substantively developed in any future submission

Authors response: We appreciate the reviewer’s valuable comment. In the revised discussion, we expanded the interpretation beyond simple prevalence comparisons by incorporating the ecological role of intermediate hosts, environmental contamination, zoonotic transmission pathways, and associated public health implications. Specifically, we discussed how the consumption of raw or undercooked fish can facilitate transmission of zoonotic parasites to humans, particularly in endemic settings with poor sanitation and close interaction between aquatic ecosystems, animals, and humans. We also strengthened the discussion with supporting literature on parasite ecology, transmission dynamics, and One Health perspectives to improve the scientific depth of the manuscript..

Reviewer #3: All in the MS. Thoroughly review each part of the manuscript. There are many errors and incomplete sections in the introduction, methods, results, discussion, and conclusions.

Authors response: We have thoroughly addressed each section of this manuscript.

Reviewer #4: Overall comment: While the manuscript addresses an important topic, the flow across sections, particularly within the Introduction and Discussion, would benefit from further refinement. At present, the narrative appears somewhat fragmented, with ideas not always presented in a clear and logical progression. To enhance readability and coherence, the authors are encouraged to introduce subheadings within the Introduction and Discussion. This would help organise key themes, guide the reader more effectively through the manuscript, and strengthen the overall structure and impact of the study.

Introduction: The Introduction would benefit from a clearer focus and stronger contextualisation. At present, it presents largely general and somewhat fragmented global information on fish parasites, without adequately grounding the study in the local context. In particular, there is no synthesis of previously reported parasites from Lake Hawassa or, more broadly, Ethiopia. Including a concise overview of existing records in the study region would significantly strengthen the rationale and highlight the knowledge gap this study aims to address.

I recommend restructuring the Introduction to:

(i) reduce generalised global information,
(ii) (ii) incorporate relevant regional and national studies, and
(iii) (iii) clearly articulate what is already known versus what remains unknown in Lake Hawassa.

Author response: We have thoroughly addressed the very important issues mentioned here and elsewhere, and the revised manuscript has been significantly improved.
Lines 12–13: These statements are more appropriate for the Introduction rather than the Abstract. The Abstract should remain concise and focused on the study objectives, methods, key results, and conclusions. I recommend removing these lines to improve clarity and maintain the appropriate structure of the Abstract.

Author response: Ok, we have used that sentence at convenient place.
Line 17: The term “economic heartbeat” is somewhat informal for a scientific manuscript. Consider replacing it with more precise wording (e.g., “key economic resource” or “plays a central role in the local economy”) to maintain an academic tone.

Author response: Ok, actually we have modified the context and did not use the word in the updated manuscript.

Lines 19–20: The term “host factors” is too broad and should be specified. Please clarify which host-related variables were investigated (e.g., size, age, sex, condition factor, or other biological or ecological traits) to improve clarity and interpretability of the study design.

Author response: Ok. We have defined what are those host factors

Lines 77–78: The sentence requires revision for clarity and grammatical accuracy. It may be rephrased as: “The Nilo-Sudanic fauna includes species represented by genera such as Alestes, Labeo, Bagrus, Citharinus, Hydrocynus, Hyperopisus and Protopterus.”

Authors response: corrected

Line 85: The genus names Barbus and Garra should be italicised in accordance with standard scientific nomenclature conventions.

Authors response: corrected

Lines 107–108: The reference Chai et al., 2005 appears outdated for supporting this statement. Please consider citing more recent literature to ensure the information reflects current knowledge in the field.

Authors response: Corrected. We have incorporated current and relevant literature, including studies published in 2025.

Lines 111–113: The statement that these are “the most common parasitic agents of fish” is misleading. The taxa listed (e.g., Opisthorchis, Anisakis, Diphyllobothrium) appear to be presented as examples rather than representative of the most common fish parasites globally. I recommend revising the wording to reflect that these are illustrative examples (e.g., “include” or “such as”) rather than definitive statements about prevalence. You could write as: ““Fish harbour a diverse range of parasitic taxa, including trematodes (flukes, e.g., Opisthorchis spp.), nematodes (roundworms, e.g., Anisakis spp.), and cestodes (tapeworms, e.g., Diphyllobothrium spp.).” A relevant reference to support this statement would be Fishes 2019 Vol. 4 Issue 1 Pages 9; doi: <https://doi.org/10.3390/fishes4010009>

Authors response: We have used the most relevant citation including those suggested by the reviewer where appropriate

Lines 120–124: must be deleted. This section contains overly generalised statements that are not universally supported. The relationship between host factors (e.g., sex, size) and parasitism is highly context-dependent and varies across host species, parasite taxa, and environmental conditions. For example, differences between males and females, or associations with body size, are not consistent across systems and should not be presented as general rules. I recommend revising this section to avoid overgeneralisation and to frame these factors as potential influences that may vary among studies. The statement regarding compromised immunity in females during the reproductive season also requires stronger justification and should be supported by appropriate, context-specific references or moderated accordingly.

Authors response: we have reframed the statement to specific parasite and fish group as the study indicated

Lines 128–130: This statement is inaccurate and overgeneralised. It is not correct to suggest that fish-borne parasitic larvae ingested by humans generally develop only into morphologically mature but sexually immature worms. The outcome depends on the parasite taxon. Some fish-borne parasites may remain larval in humans, whereas others can develop into adult stages and establish patent infections. I recommend revising this sentence to reflect the diversity of transmission pathways and host outcomes more accurately, and to avoid oversimplification of zoonotic potential. You could write “Larval stages of some fish-borne parasites, including certain nematodes and trematodes, may be transmitted to humans through the consumption of raw or undercooked fish, posing important zoonotic risks.” And you could reference articles such as Fishes 2019 Vol. 4 Issue 1 Pages 9; doi:10.3390/fishes4010009 and Veterinary Quarterly 2022 Vol. 42 Issue 1 Pages 95-118

Authors response: We have used the most relevant citation including those suggested by the reviewer where appropriate.

Lines 131–135: The points raised are valid; however, the references used are somewhat dated. There is a substantial body of more recent literature addressing parasite transmission dynamics, host–parasite interactions, and the role of trophic ecology. I recommend incorporating more up-to-date references to better reflect current understanding in the field. Consider these or anything more recent:
Parasitology 2024 Vol. 151 Issue 8 Pages 864-874
Canadian Journal of Zoology 2023; DOI: 10.1139/cjz-2022-0126
Scientific Reports 2023 Vol. 13 Issue 1 Pages 10291; <https://doi.org/10.1038/s41598-023-37428-y>

Authors response: We have used the most relevant citation including those suggested by the reviewer where appropriate.

Line 146: the reference is outdated

Authors response: We have used the most relevant citation including those suggested by the reviewer where appropriate.

Materials and methods: Lines 174–182: The content in this section does not appear to describe methodological procedures and is more appropriate for the Introduction. I recommend relocating this text to the Introduction and ensuring that the Methods section is limited to study design, sampling, and analytical approaches.

Authors response: we have relocated the section to let the methods section fully focus on study design, sampling, and analytical approaches

Lines 184–189: The sample size calculation is clearly described; however, it appears that a collective sample size was applied across all fish species. Please explicitly specify the number of specimens examined for each fish species included in the study. This information is essential for interpreting species-specific prevalence, intensity, and comparative analyses.

Authors response: Yes, we have used collective sample size and disturbed the sample size to the three commercially important fishes in the area

1. *O. niloticus*, n=135,
2. *B. intermedius* also known as *Labeobarbus intermedius*, n=87,
3. *C. gariepinus*, n=43.

Line 192: The term “different sampling points” is vague and requires clarification. Please specify the exact sampling locations (e.g., named sites, coordinates, or habitat types) within the study area. In addition, it is unclear how these sampling points were selected (e.g., random, stratified, based on accessibility or ecological characteristics). Clarifying the selection criteria is important for assessing potential sampling bias and ensuring reproducibility.

Author response: **We used multiple sampling sites and collected samples from different parts of the lake during every sampling period.** The selection of sampling sites was mainly based on accessibility and the feasibility of obtaining the necessary field support.

Lines 207–213: This interpretation is too strong based solely on body length data. Without ageing the fish, or providing species-specific evidence on maturity and developmental stage, it is not possible to conclude that shorter fish represent less mature individuals while larger fish represent fully mature adults with broader dietary niches. In addition, differences in parasite burden between size classes cannot be interpreted simply as a function of development or exposure, as smaller fish may also differ in condition, growth, or health status. I recommend moderating this section substantially and avoiding biological interpretations that are not directly supported by the data.

Author response: **In this classification we have used the standard previously used or adopted and published. Each of the three fish species used are classified into class I, class II and class III but the size measurement differs for each species. In line with this conclusion** Lima et al, (2012) stated that classifying fish species based on size and body weight during the parasite studies is crucial because this factor can influence parasite infection patterns, host vulnerability and the overall dynamics of parasite host interaction impacting both fish health and environmental ecosystem.

Line 215: Please specify the fish species examined and the number of individuals for each species. This information is essential for interpreting the results and for enabling species-specific comparisons and should be clearly presented, either in the text or as a summary table.

Author response:  **we have clarified the points raised in the updated manuscript**
Lines 219–225: The euthanasia procedure is now clearly described and appreciated. The methods used appear appropriate for rapid loss of consciousness. For future studies, the authors may also consider the use of anaesthetic agents (e.g., clove oil or other approved fish anaesthetics), which are commonly recommended in many guidelines to further minimise stress and handling-related impacts prior to euthanasia.

Authors response: we thank you and correctly apply in future studies
Lines 239–250: The use of the pepsin–HCl artificial digestion method is appropriate and well described, and it is commendable that the authors have applied a recognised technique for detecting encysted larvae in muscle tissue. However, it appears that this method (or equivalent digestion/incubation approaches see International Journal of Food Microbiology 2016 Vol. 227 Pages 13-16 and Canadian Journal of Fisheries and Aquatic Sciences 1986 Vol. 43 Issue 7 Pages 1312-1317) was not applied to other organs, particularly the gastrointestinal tract, where many parasites are commonly located and are deeply embedded in the tissue. This may have limited the detection of a broader range of parasites and potentially led to an underestimation of parasite diversity and burden. I recommend that the authors acknowledge this as a limitation of the study and briefly discuss how it may have influenced their findings particularly for parasites that preferentially inhabit non-muscle tissues.”

Authors response: for parasites that preferentially inhabit non-muscle tissues. We kindly ask the reviewer to recheck the edits and revisions we have currently made. In response to the comment, we have acknowledged the limitation associated with the use of the pepsin–HCl artificial digestion method. Specifically, we clarified that this method was not applied to other organs, particularly the gastrointestinal tract, where many parasites are commonly located and may be deeply embedded within tissues. Consequently, this methodological limitation may have reduced the detection of a broader range of parasites and could have led to an underestimation of parasite diversity and burden. We have also briefly discussed how this limitation may have influenced the findings, especially

Lines 253–255: This statement appears misplaced, as it follows the section on parasite identification and does not directly contribute to that context. The justification for muscle digestion is methodological and would be more appropriate within the earlier description of the digestion procedure. I recommend either relocating this sentence to the Methods section where the technique is first introduced or removing it to improve the logical flow and coherence of the manuscript.

Lines 260–267: The reference cited for these parasitological parameters appears to be incorrect. The standard reference is Bush et al. (1997). Please revise accordingly and ensure consistent citation of this foundational work. Bush AO, Lafferty KD, Lotz JM, Shostak AW. Parasitology meets ecology on its own terms: Margolis et al revisited. J Parasitol. 1997 Aug;83(4):575-83.

Ok. We have used the correct refence for the analysis we have used.
Lines 277–280: The description of sampled fish species and size ranges is clear; however, it is important to note whether fish age was determined. If age data were not collected, this should be explicitly stated in the Methods. In addition, I recommend acknowledging in the Discussion that the absence of age determination limits interpretation of parasite–host relationships, particularly where size is used as a proxy for developmental stage or exposure.

Authors response: We have acknowledged the limitation and addressed the discussion accordingly.

Results and Table 1: The presentation of prevalence across fish species is clear; however, the authors should interpret these values with caution. As digestion/incubation methods (see International Journal of Food Microbiology 2016 Vol. 227 Pages 13-16 and Canadian Journal of Fisheries and Aquatic Sciences 1986 Vol. 43 Issue 7 Pages 1312-1317) were not applied to all organs (particularly the gastrointestinal tract), it is likely that some parasites were not detected. This may have led to an underestimation of the true parasite diversity and prevalence. I recommend that the authors acknowledge this limitation in the Results (briefly) and more explicitly in the Discussion, noting that the reported prevalence values likely represent conservative estimates.

Authors response: We have acknowledged the limitation and addressed the discussion accordingly.

Table 2: The analysis relating parasitic infestation to sex, body weight, and length is informative; however, these results should be interpreted with caution in the absence of age determination. Body size alone is not a reliable proxy for age, maturity, or cumulative exposure to parasites. In addition, parasite biomass itself may confound weight-based interpretations. Heavily infected fish may appear heavier not because they are in better condition, but due to the physical mass of the parasites they harbour. For example, studies on clupeids from the Caspian Sea (Parasitology Research 2018 Vol. 17 Issue 10 Pages 3327-3331) have shown that 3-year-old fish infected with pathogenic Corynosoma spp. appeared heavier than uninfected fish of the same age class, with the increased weight attributable to parasite load rather than improved host condition. This article also might be very useful: Int J Parasitol 2020 Vol. 50 Issue 10 Pages 755-761. I recommend that the authors acknowledge these limitations and avoid overinterpreting associations between parasite burden and host size metrics. Incorporating this consideration into the Discussion would strengthen the biological interpretation of the findings.

Authors response: we have addressed it in the updated manuscript.

Table 4: Myxobolus species are NOT protozoa. Dactylogyrus species are not Trematode either!

Authors response: We have reclassified the two into appropriate genera into Monogenea and Myxosporea.

Lines 467–475: This is a useful comparison with previous studies, and the discussion on variation in prevalence across water bodies is appreciated. To further strengthen this section, the authors may consider briefly linking these findings to the ecological and pathological significance of these parasites in definitive hosts. In particular, digenean flukes such as Clinostomum and Euclinostomum spp. are known to infect fish-eating birds as definitive hosts, where they can have important health and ecological implications. Including a short statement on their pathogenicity and role in avian hosts would provide a more complete picture of the parasite’s life cycle and broader significance.

Authors response: We have addressed the issue in the updated manuscript with appropriate reference
