# Supplementary material for: Prevalence of fish parasites from Lake Hawassa, Ethiopia
Source: PLoS One. 2026 Sep 11;21(9):e0357816. doi: 10.1371/journal.pone.0357816 (PMC13568496; doi:10.1371/journal.pone.0357816)
Supplement: Supplementary file 7 [file pone.0357816.s008.docx]

PONE-D-25-59453R2

Prevalence of fish parasites from Lake Hawassa, Ethiopia

comments:

Response/Clarfication to Reviewer's to comments

1. If the authors have adequately addressed your comments raised in a previous round of review and you feel that this manuscript is now acceptable for publication, you may indicate that here to bypass the “Comments to the Author” section, enter your conflict-of-interest statement in the “Confidential to Editor” section, and submit your "Accept" recommendation.

Reviewer #2: All comments have been addressed. Thank

Reviewer #4: (No Response)

Authors response: We sincerely thank the reviewer for the careful evaluation of our manuscript and for confirming that all comments have been satisfactorily addressed. We appreciate your constructive feedback, which has helped improve the quality and clarity of our manuscript.

2. Is the manuscript technically sound, and do the data support the conclusions?

The manuscript must describe a technically sound piece of scientific research with data that supports the conclusions. Experiments must have been conducted rigorously, with appropriate controls, replication, and sample sizes. The conclusions must be drawn appropriately based on the data presented.

Reviewer #2: Yes

Reviewer #4: Partly

Authors response: We sincerely thank the reviewer for the careful evaluation of our manuscript and for confirming that all comments have been satisfactorily addressed. We appreciate your constructive feedback, which has helped improve the quality and clarity of our manuscript.

3. Has the statistical analysis been performed appropriately and rigorously?

Reviewer #2: Yes

Reviewer #4: Yes

Authors response: We sincerely thank the reviewer for the careful evaluation of our manuscript and for confirming that all comments have been satisfactorily addressed. We appreciate your constructive feedback, which has helped improve the quality and clarity of our manuscript.

4. Have the authors made all data underlying the findings in their manuscript fully available?

The [PLOS Data policy](http://track.editorialmanager.com/CL0/http:%2F%2Fwww.plosone.org%2Fstatic%2Fpolicies.action%23sharing/1/010f019f1140f22f-82454f66-16f6-4269-b6f9-c2e2ce699795-000000/cZrlVnbm3Ynwn-IcHFnxwL9MjMqza-gDzmOkvimWKBI=258) requires authors to make all data underlying the findings described in their manuscript fully available without restriction, with rare exception (please refer to the Data Availability Statement in the manuscript PDF file). The data should be provided as part of the manuscript or its supporting information, or deposited to a public repository. For example, in addition to summary statistics, the data points behind means, medians and variance measures should be available. If there are restrictions on publicly sharing data—e.g. participant privacy or use of data from a third party—those must be specified.

Reviewer #2: Yes

Reviewer #4: No

Authors response: We appreciate the reviewer's comment. All data supporting the findings of this study have been made available within the manuscript and are also provided as an additional supplementary file. Furthermore, we have included a Data Availability Statement indicating that we are happy to respond to any specific requests or questions regarding the data and to provide any additional information required.

5. Is the manuscript presented in an intelligible fashion and written in standard English?

PLOS ONE does not copyedit accepted manuscripts, so the language in submitted articles must be clear, correct, and unambiguous. Any typographical or grammatical errors should be corrected at revision, so please note any specific errors here.

Reviewer #2: Yes

Reviewer #4: No

Authors Response: We have made substantial improvements to the English language throughout the manuscript and have proofread it twice to correct any grammatical errors and improve its overall clarity and readability.

6. Review Comments to the Author

Please use the space provided to explain your answers to the questions above. You may also include additional comments for the author, including concerns about dual publication, research ethics, or publication ethics. (Please upload your review as an attachment if it exceeds 20,000 characters)

**Reviewer #2:** -Nomenclature consistency: "B. intermedius" and "L. intermedius" are used interchangeably throughout — pick one (suggest Labeobarbus intermedius, noting "also known as B. intermedius" on first use) and apply consistently.

Authors reply: **For consistency, we have used only one scientific name throughout the manuscript:** L. intermedius**. At its first mention, we defined it as being synonymous with B. intermedius, and thereafter we consistently used L. intermedius throughout the text.**

-Table 2, C. gariepinus Total Length: p=0.0492 is statistically significant, but the text (line 295) states the distribution "showed not statistically significant association" for all variables. Either flag/discuss this one significant result briefly in Results/Discussion or double-check the value.
Authors reply: After rechecking our data, we identified an editorial error in reporting the p-value for total length in Table 2. This was a typographical error in the manuscript only; the underlying analysis and results remain unchanged. The p-value has now been corrected in Table 2

Line 41 typo: "was to be 8.59, 7.92 and 6.79" → "was found to be 8.59, 7.92 and 6.79".
Authors reply: We have addressed the suggestions.

-Line 98: Parasitic species can be found everywhere and on every living organism. need citation: <https://www.nature.com/articles/s41598-026-46340-0>

Authors reply: We have addressed this suggestion and incorporated the relevant references into the revised manuscript.
Line 121-122: Fish is a good host for parasite multiplication that can be zoonotic to humans or other animals that feed fishes. needs updated citation:<https://www.tandfonline.com/doi/abs/10.1080/10454438.2026.2662934>
Authors reply: We have addressed this suggestion and incorporated the relevant references into the revised manuscript.
-Line 272–273: Overall length range "10cm to 80cm" — worth confirming this matches the per-species length classes in Table 2 (max categories are ">25cm"/">26cm"/">70cm"), since 80cm seems outside those bins.

Authors response: We have corrected the values from **less than 10** to **greater than 70**. Thank you for bringing this to our attention.

-Figure 1 caption: "Barbus species, the middle" — specify full species name (B. intermedius) for consistency with the rest of the text.
Authors response: OK. We have addressed the suggestion.

-Table 4 header: "Parasite class" column — "Hurindae" should be "Hirudinea" (correct spelling).
Authors response: OK. We have addressed the issue
-Line 320 (Table 3 reference): "Contacaecum" → "Contracaecum" (typo, appears twice in that paragraph).
Authors response: OK. We have addressed the issue.
-Welfare/spelling: "welbeing" (abstract, line 21) → "wellbeing".
Authors response: OK. We have addressed the issue.
-Consistent terminology: "infection/infestation" used interchangeably per reviewer's earlier note — a brief definitional sentence early in Methods clarifying usage would help, though this is optional polish.

Authors response: OK. Throughout the manuscript, the terms **"infection"** and **"infestation"** have been used interchangeably in sections that indicates both endoparasites and ectoparasites. To improve clarity and maintain consistency, we have revised the text, using **"parasitic infection for** endoparasites **and infestation for** ectoparasites**.**

**Reviewer #4:** Lines 29–33: The term "overall infection/infestation rate" is ambiguous and should be replaced with a clearly defined parasitological parameter. If the authors are referring to the proportion of fish harbouring at least one parasite species, the term overall prevalence would be more appropriate. The authors should also clearly state how the value of 88.30% was calculated and whether it represents infection with any parasite taxon or a specific subset of parasites. Consistent use of standard parasitological terminology (e.g., prevalence, mean intensity, mean abundance) throughout the manuscript is recommended. The manuscript would benefit from following the recommendations of Bush et al. regarding standard ecological and parasitological terminology, particularly for prevalence, intensity, and abundance measures.

Authors response: We have clearly reported the overall prevalence, representing the proportion of fish infected with any parasite. Specifically, of the 265 fish examined, 88.30% (n = 234) harboured at least one parasite, either as a single infection or as part of a multiple-parasite infection. Furthermore, throughout the manuscript we have adhered to the standard ecological and parasitological terminology and definitions recommended by Bush et al.

Introduction (first 3 paragraphs) and elsewhere: The manuscript opens with a discussion of the importance of aquaculture; however, the study was conducted in a natural lake ecosystem rather than an aquaculture setting. As currently written, the relevance of these introductory paragraphs to the study is unclear. If the lake is used for aquaculture, stocking programmes, or other aquaculture-related activities, this should be clearly explained and linked to the study objectives. If not, I recommend revising the introduction to focus more directly on the ecological, fisheries, conservation, or public health significance of parasite infections in wild fish populations within the study system. This would provide a stronger rationale and improve the overall flow of the manuscript.

Despite the growing importance of fisheries and aquaculture in Ethiopia, parasitic infections in wild fish populations remain a significant but often overlooked challenge. Fish parasites can reduce growth, survival, and reproductive performance, leading to declines in fish productivity and biodiversity while compromising the sustainability of inland fisheries. In Ethiopia's major lakes and reservoirs, where wild fish constitute the primary source of fish production, parasitic diseases can negatively affect fish health, increase post-harvest losses, and reduce the economic returns of fishing communities. Some fish parasites also possess zoonotic potential, posing public health risks through the consumption of raw or inadequately cooked fish. Therefore, understanding the diversity, prevalence, and distribution of fish parasites in Ethiopia's wild fish populations is essential for fisheries management, aquatic ecosystem conservation, food safety, and the development of effective disease surveillance and control strategies.

Introduction: The Introduction would benefit from a concise review of the parasites previously reported from the host species. At present, I could not readily determine what is already known about the parasite fauna of this fish, whether similar studies have been conducted elsewhere, and what specific knowledge gap this study addresses. Including a brief summary of previous records would help establish the novelty and significance of the present work.

Authors reply: We have refined the introduction and incorporated relevant previous studies conducted in Ethiopia to provide better context and strengthen the rationale for the study.

Materials and Methods: Throughout the Materials and Methods section, parasites are referred to by their final taxonomic identities (e.g. Diplostomum spp. and other identified taxa). This information represents a study result and should therefore be presented in the Results section, together with the morphological and/or molecular evidence supporting those identifications. In the Materials and Methods, it would be more appropriate to use broader terms such as "digenean trematodes", "nematodes", "cestodes", "helminths", or "parasite specimens" and then describe the identification procedures used. The specific parasite identities should be introduced only after the supporting evidence has been presented in the Results.

Authors response: We have not used any confirmed parasite names in the Materials and Methods section, except for Diplostomum spp. The term Diplostomum spp. was used only in the specific section describing the methods for identifying this parasite and distinguishing it from other parasites. Elsewhere in the Materials and Methods section, we intentionally avoided using confirmed parasite names because species identification was based on the results obtained after the analyses.

Line 245: citation “El-245 Shahawy et al., 2017)” should be deleted

Authors response: we have deleted “El-Shahawy et al., 2017, instead we have used the standard ecological and parasitological terminology and definitions recommended by Bush et al.

Parasite identification and presentation of results: A major concern is that the Results section provides little evidence supporting the identification of the reported parasite taxa. While parasites are assigned to genera such as Diplostomum, Clinostomum, Euclinostomum, Contracaecum, Myxobolus, and others, the manuscript does not adequately describe the diagnostic features used to reach these identifications. Readers are therefore unable to independently assess the reliability of the taxonomic determinations.

Authors response: Thank you for this valuable comment. We agree that the original manuscript did not provide sufficient information on the diagnostic criteria used for parasite identification, which may have limited readers' ability to assess the reliability of our taxonomic determinations. Specifically, we have added a detailed description of the morphological characteristics and taxonomic keys used to identify each parasite taxon. Identification was based on established morphological features, including body shape, size, location within the host, cyst morphology (where applicable), and other diagnostic characters observed under stereomicroscopy and light microscopy, following standard taxonomic references. We have also cited the appropriate identification guides and published literature used for each parasite group.

In addition, the quality and presentation of the figures require substantial improvement. The images are of insufficient quality to demonstrate the key morphological characters used for identification. Many diagnostic structures are either not visible or not clearly indicated. Furthermore, the figures lack essential elements expected in taxonomic and parasitological studies, including scale bars and clear annotations highlighting important morphological features. As currently presented, the images do not provide adequate support for the parasite identifications reported in the manuscript.

Authors response: We agree that the original figures did not adequately illustrate the diagnostic morphological characteristics required to support parasite identification. In response to this comment, we have substantially improved the quality and presentation of all relevant figures. Although we were unable to return to the field to collect fresh parasite specimens with scale bars and higher-resolution imaging, we have carefully enhanced the available images to better highlight the key morphological features necessary for parasite identification. We believe that the revised figures now more clearly reflect the diagnostic characteristics and provide stronger visual support for our findings.

The authors should either provide a more detailed account of the diagnostic criteria used for identification, supported by appropriate references, or include higher-quality photomicrographs showing the relevant taxonomic characters, with scale bars and annotations where appropriate. Without such evidence, it is difficult to assess the accuracy of the reported parasite identifications.

Authors response:

Thank you for this valuable comment. We have revised the manuscript to strengthen the description of our diagnostic criteria and have cited the appropriate taxonomic references, including Bush et al., to support parasite identification. Parasites were identified based on established morphological characteristics, including body shape, size, site of infection within the host, cyst morphology (where applicable), and other diagnostic features observed under stereomicroscopy and light microscopy, following standard taxonomic guidelines.

Although we were unable to return to the field to collect fresh parasite specimens and obtain higher-resolution images with scale bars, we have carefully enhanced the quality of the available images to improve the visibility of the key morphological features used for parasite identification. We acknowledge this as a limitation of the study and have clarified this in the revised manuscript.
