# Supplementary material for: Prevalence of fish parasites from Lake Hawassa, Ethiopia
Source: PLoS One. 2026 Sep 11;21(9):e0357816. doi: 10.1371/journal.pone.0357816 (PMC13568496; doi:10.1371/journal.pone.0357816)
Supplement: Peer review history

# Peer review: Decision Letter 0

**DOI:** 10.1371/journal.pone.0357816.r001

**Authors:** Clement Yaro

26 Nov 2025

Dear Dr. EFA,

Thank you for submitting your manuscript to PLOS ONE. After careful consideration, we feel that it has merit but does not fully meet PLOS ONE’s publication criteria as it currently stands. Therefore, we invite you to submit a revised version of the manuscript that addresses the points raised during the review process.

Please submit your revised manuscript by Jan 10 2026 11:59PM. If you will need more time than this to complete your revisions, please reply to this message or contact the journal office at plosone@plos.org. When you're ready to submit your revision, log on to https://www.editorialmanager.com/pone/ and select the 'Submissions Needing Revision' folder to locate your manuscript file.

- A rebuttal letter that responds to each point raised by the academic editor and reviewer(s). You should upload this letter as a separate file labeled 'Response to Reviewers'.
- A marked-up copy of your manuscript that highlights changes made to the original version. You should upload this as a separate file labeled 'Revised Manuscript with Track Changes'.
- An unmarked version of your revised paper without tracked changes. You should upload this as a separate file labeled 'Manuscript'.

If you would like to make changes to your financial disclosure, please include your updated statement in your cover letter. Guidelines for resubmitting your figure files are available below the reviewer comments at the end of this letter.

If applicable, we recommend that you deposit your laboratory protocols in protocols.io to enhance the reproducibility of your results. Protocols.io assigns your protocol its own identifier (DOI) so that it can be cited independently in the future. For instructions see: https://journals.plos.org/plosone/s/submission-guidelines#loc-laboratory-protocols. Additionally, PLOS ONE offers an option for publishing peer-reviewed Lab Protocol articles, which describe protocols hosted on protocols.io. Read more information on sharing protocols at https://plos.org/protocols?utm_medium=editorial-email&utm_source=authorletters&utm_campaign=protocols.

We look forward to receiving your revised manuscript.

Kind regards,

Clement Ameh Yaro, Ph.D

Academic Editor

PLOS ONE

Journal requirements:

When submitting your revision, we need you to address these additional requirements.

1. Please ensure that your manuscript meets PLOS ONE’s style requirements, including those for file naming. The PLOS ONE style templates can be found at

https://journals.plos.org/plosone/s/file?id=wjVg/PLOSOne_formatting_sample_main_body.pdf and

https://journals.plos.org/plosone/s/file?id=ba62/PLOSOne_formatting_sample_title_authors_affiliations.pdf

2. In your Methods section, please provide additional information regarding the permits you obtained for the work. Please ensure you have included the full name of the authority that approved the field site access and, if no permits were required, a brief statement explaining why.

3. To comply with PLOS One submissions requirements, in your Methods section, please provide additional information regarding the experiments involving animals and ensure you have included details on (1) methods of sacrifice, (2) methods of anesthesia and/or analgesia, and (3) efforts to alleviate suffering.

4. When completing the data availability statement of the submission form, you indicated that you will make your data available on acceptance. We strongly recommend all authors decide on a data sharing plan before acceptance, as the process can be lengthy and hold up publication timelines. Please note that, though access restrictions are acceptable now, your entire data will need to be made freely accessible if your manuscript is accepted for publication. This policy applies to all data except where public deposition would breach compliance with the protocol approved by your research ethics board. If you are unable to adhere to our open data policy, please kindly revise your statement to explain your reasoning and we will seek the editor’s input on an exemption. Please be assured that, once you have provided your new statement, the assessment of your exemption will not hold up the peer review process.

5. We note that Figure 1 in your submission contain [map/satellite] images which may be copyrighted. All PLOS content is published under the Creative Commons Attribution License (CC BY 4.0), which means that the manuscript, images, and Supporting Information files will be freely available online, and any third party is permitted to access, download, copy, distribute, and use these materials in any way, even commercially, with proper attribution. For these reasons, we cannot publish previously copyrighted maps or satellite images created using proprietary data, such as Google software (Google Maps, Street View, and Earth). For more information, see our copyright guidelines: http://journals.plos.org/plosone/s/licenses-and-copyright.

We require you to either (1) present written permission from the copyright holder to publish these figures specifically under the CC BY 4.0 license, or (2) remove the figures from your submission:

1. You may seek permission from the original copyright holder of Figure 1 to publish the content specifically under the CC BY 4.0 license.

We recommend that you contact the original copyright holder with the Content Permission Form (http://journals.plos.org/plosone/s/file?id=7c09/content-permission-form.pdf) and the following text:

“I request permission for the open-access journal PLOS ONE to publish XXX under the Creative Commons Attribution License (CCAL) CC BY 4.0 (http://creativecommons.org/licenses/by/4.0/). Please be aware that this license allows unrestricted use and distribution, even commercially, by third parties. Please reply and provide explicit written permission to publish XXX under a CC BY license and complete the attached form.”

Please upload the completed Content Permission Form or other proof of granted permissions as an "Other" file with your submission.

In the figure caption of the copyrighted figure, please include the following text: “Reprinted from [ref] under a CC BY license, with permission from [name of publisher], original copyright [original copyright year].”

2. If you are unable to obtain permission from the original copyright holder to publish these figures under the CC BY 4.0 license or if the copyright holder’s requirements are incompatible with the CC BY 4.0 license, please either i) remove the figure or ii) supply a replacement figure that complies with the CC BY 4.0 license. Please check copyright information on all replacement figures and update the figure caption with source information. If applicable, please specify in the figure caption text when a figure is similar but not identical to the original image and is therefore for illustrative purposes only.

The following resources for replacing copyrighted map figures may be helpful:

USGS National Map Viewer (public domain): http://viewer.nationalmap.gov/viewer/

The Gateway to Astronaut Photography of Earth (public domain): http://eol.jsc.nasa.gov/sseop/clickmap/

Maps at the CIA (public domain): https://www.cia.gov/library/publications/the-world-factbook/index.html and https://www.cia.gov/library/publications/cia-maps-publications/index.html

NASA Earth Observatory (public domain): http://earthobservatory.nasa.gov/

Landsat: http://landsat.visibleearth.nasa.gov/

USGS EROS (Earth Resources Observatory and Science (EROS) Center) (public domain): http://eros.usgs.gov/#

Natural Earth (public domain): http://www.naturalearthdata.com/

6. We note that Figure 2 in your submission contain copyrighted images. All PLOS content is published under the Creative Commons Attribution License (CC BY 4.0), which means that the manuscript, images, and Supporting Information files will be freely available online, and any third party is permitted to access, download, copy, distribute, and use these materials in any way, even commercially, with proper attribution. For more information, see our copyright guidelines: http://journals.plos.org/plosone/s/licenses-and-copyright.

We require you to either (1) present written permission from the copyright holder to publish these figures specifically under the CC BY 4.0 license, or (2) remove the figures from your submission:

1. You may seek permission from the original copyright holder of Figure 2 to publish the content specifically under the CC BY 4.0 license.

We recommend that you contact the original copyright holder with the Content Permission Form (http://journals.plos.org/plosone/s/file?id=7c09/content-permission-form.pdf) and the following text:

“I request permission for the open-access journal PLOS ONE to publish XXX under the Creative Commons Attribution License (CCAL) CC BY 4.0 (http://creativecommons.org/licenses/by/4.0/). Please be aware that this license allows unrestricted use and distribution, even commercially, by third parties. Please reply and provide explicit written permission to publish XXX under a CC BY license and complete the attached form.”

Please upload the completed Content Permission Form or other proof of granted permissions as an "Other" file with your submission.

In the figure caption of the copyrighted figure, please include the following text: “Reprinted from [ref] under a CC BY license, with permission from [name of publisher], original copyright [original copyright year].”

2. If you are unable to obtain permission from the original copyright holder to publish these figures under the CC BY 4.0 license or if the copyright holder’s requirements are incompatible with the CC BY 4.0 license, please either i) remove the figure or ii) supply a replacement figure that complies with the CC BY 4.0 license. Please check copyright information on all replacement figures and update the figure caption with source information. If applicable, please specify in the figure caption text when a figure is similar but not identical to the original image and is therefore for illustrative purposes only.

If the reviewer comments include a recommendation to cite specific previously published works, please review and evaluate these publications to determine whether they are relevant and should be cited. There is no requirement to cite these works unless the editor has indicated otherwise.

[Note: HTML markup is below. Please do not edit.]

Reviewers' comments:

Reviewer’s Responses to Questions

**Comments to the Author**

1. Is the manuscript technically sound, and do the data support the conclusions?

Reviewer #1: Yes

Reviewer #2: Yes

Reviewer #3: No

Reviewer #4: Partly

**********

2. Has the statistical analysis been performed appropriately and rigorously?

Reviewer #1: Yes

Reviewer #2: Yes

Reviewer #3: No

Reviewer #4: Yes

**********

3. Have the authors made all data underlying the findings in their manuscript fully available?

The PLOS Data policy

Reviewer #1: Yes

Reviewer #2: Yes

Reviewer #3: No

Reviewer #4: Yes

**********

4. Is the manuscript presented in an intelligible fashion and written in standard English?

Reviewer #1: Yes

Reviewer #2: Yes

Reviewer #3: No

Reviewer #4: No

**********

Reviewer #1: Dear Author

I sincerely appreciate the opportunity to review this article, which is titled:

Prevalence of fish parasites from Lake Hawassa, Ethiopia

Manuscript Number: PONE-D-25-59453

That is a good, clear title, but it could be significantly improved by adding more specificity about the hosts and the study’s key finding, which increases its impact. This is a great starting point for a manuscript, but there are several points that require clarification or correction to meet high scientific standards.

Here is a point-by-point scientific review:

The manuscript is efficient, but the lack of citations for key statistics and claims is a serious issue that must be addressed before submission. You also need to correct the simple grammar and spelling errors noted in Ms. Strengthening the opening statements, maintaining scientific accuracy, and attending to the requirement for citations are the main objectives of this review.

Kindly find the attached file

Reviewer #2: This manuscript presents a cross-sectional parasitological survey of three commercially important fish species from Lake Hawassa, Ethiopia. While the topic is relevant and the sample size is adequate, the manuscript requires substantial revision before publication. The study provides valuable baseline data on fish parasite prevalence but suffers from some gaps, incomplete taxonomic identification, and inadequate contextualization of findings.

1- Taxonomic Identification Limitations

The authors acknowledge limiting identification to genus level, citing that "fish harbors mostly larval stages" (lines 242-243). However, this is scientifically problematic:

-Species-level identification is feasible: Modern molecular techniques (PCR, DNA barcoding) can identify larval stages to species level. The authors provide no justification for not attempting molecular identification.

-Public health implications: For zoonotic parasites like Contracaecum spp., species-level identification is crucial as different species have different zoonotic potential.

-Ecological interpretation: Genus-level identification limits ecological and epidemiological interpretations.

Question 1: Why were molecular identification methods not employed, especially given the zoonotic importance of several parasites identified? Were resources unavailable, or was this a deliberate methodological choice?

2- water quality parameters:

The manuscript mentions water quality issues (lines 362-377, 488-491, 708-710) but provides NO water quality data. The authors acknowledge environmental stressors but fail to measure them. Multiple references cited (Memberu et al., 2021; Tibebe et al., 2023) demonstrate poor water quality in Lake Hawassa

Question 2: Were any water quality parameters measured during this study? If not, why was this critical environmental component excluded from a parasitological survey?

3-Temporal Sampling Design

"October 2023 to July 2024" and "conducted in all for over 10 months" (lines 169-173)

-Were fish collected continuously or at intervals?

-What was the sampling frequency?

-Were seasonal variations accounted for?

-Multiple citations (Bakke et al., 2002; Ozer et al., 2004; Khidr et al., 2012; Khan et al., 2018) emphasize seasonal effects on parasite prevalence

-Provide a clear sampling timeline and sample sizes per collection period. Analyze seasonal effects if data permit.

4-Sample Selection

"Systematic random sampling" (line 171): This term is contradictory. Was sampling:

-Systematic? or Random (using random number generators)? or Stratified by species?

-Source of fish unclear: Were fish collected:

=Directly from the lake (by researchers)?

=From fishermen (introducing selectivity bias)?

=From fish markets (introducing preservation/handling artifacts)?

-Sampling locations: No description of geographic sampling within Lake Hawassa

5- some parasitological methods lack details as magnification power, fixation details...etc

6-Results:

-Table 1 vs. Text discrepancy:

-Table 1 reports 788 parasites from O. niloticus (line 277)

-Text reports 787 parasites (lines 30-31, abstract)

Which is correct?

7-Fish morphometrics:

-No mean ± SD for fish weights and lengths per species

-Weight and length ranges provided (lines 199-206) but no central tendencies

-Impossible to assess if samples represent typical population demographics

8- Parasite intensity data:

-Table 1 shows "Number of parasites from each fish: 6-9, 4-6, 5-10"

-These ranges are unclear: Are these per infected fish? Per parasite species?

-Mean intensities (6.79, 7.92, 8.59) don't align logically with these ranges

9- Discussion:

-Comparison-heavy, interpretation-light: The discussion is dominated by comparing prevalence values to other studies (lines 329-343) without explaining WHY differences exist

Ecological context missing:

-No discussion of Lake Hawassa’s specific ecological characteristics

-How do lake depth, temperature, presence of intermediate hosts affect parasite communities?

-What snail species are present (necessary for digenean life cycles)?

Zoonotic risk inadequately addressed:

-Contracaecum spp. are identified as zoonotic (lines 421-427) but no risk assessment is provided

-What are fish consumption practices in the region?

-Are fish cooked adequately?

-No connection to the cultural practices mentioned (lines 84-89)

Typographical errors:

-Line 16: "pastes" → "parasites"

-Line 38-39: "mean intensity...is found to be" → awkward phrasing; use "was"

-Line 64: "Some of the lakes" → "Several lakes" (more formal)

-Line 73: "accounts 90%" → "accounts for 90%"

enhance your discussion with following:

-https://link.springer.com/article/10.1007/s10499-025-02205-3

-https://link.springer.com/article/10.1007/s10499-025-02037-1

-https://www.nature.com/articles/s41598-025-19529-y

Reviewer #3: The authors have important information. However, the way the article is presented does not meet minimum quality standards. I include many observations in the document. The authors should review the document thoroughly.

Reviewer #4: Thank you for the opportunity to review this manuscript. The topic is important and highly relevant, particularly given the need for more research from regions such as Ethiopia, where local data can substantially improve global understanding. The effort invested in this study is evident, and with further refinement the manuscript has the potential to make a valuable contribution.

Below I outline several areas where improvements would strengthen the clarity, scientific quality, and overall impact of the work. The manuscript would benefit from substantial improvement in both English expression and scientific writing. At present, the narrative is difficult to follow, and key messages become obscured by issues of organisation and clarity.

A more structured, focused, and concise writing style is strongly recommended.

Lines 13–14:

Please clarify whether you are referring to 200 species of marine fish, freshwater fish, or both. This distinction is important for understanding the ecological context of the study and the relevance of the lake system.

Lines 12–17:

It would strengthen the introduction to briefly outline the significance of the Lake. A concise sentence or two on its ecological value, socio-economic importance, or role in local fisheries would help readers unfamiliar with the region appreciate why this research matters.

Lines 18–25:

Please specify the number of fish examined and list the species included in the study. Clear reporting of sample size and species composition is essential for evaluating the validity and representativeness of the findings.

Lines 27–28:

The information provided here relates to study procedures and should be relocated to the Methodology section. This will improve the organisation and scientific flow of the manuscript.

Lines 27–39

The reporting in this section requires substantial reorganisation for clarity and scientific accuracy.

-Dactylogyrus is not an internal parasite. Please correct its placement. Dactylogyrus spp. are monogenean ectoparasites located on gills and should not be grouped with internal helminths.

-For scientific clarity, this whole section would be much stronger if reorganised by major taxonomic groups, rather than by host species. Grouping the results this way will make the ecological patterns easier to follow and will align with standard parasitological reporting practices. A suggested structure is:

o Nematodes (e.g., Contracaecum)

o Digenean trematodes (e.g., Clinostomum, Euclinostomum, Diplostomum, encysted metacercariae)

o Monogeneans (Dactylogyrus)

o Myxozoans (Myxobolus spp.)

o Crustacean ectoparasites (Argulus, Dolops)

o Hirudinea (leeches)

-The sentence listing sample sizes (Oreochromis niloticus n=135, Clarias gariepinus n=87, Barbus intermedius n=43) should appear at the beginning of the Results section or in the Methods, not within the middle of the parasite description.

-The overall infection rate (88.30%, n=234) is useful, but please clarify:

o Infection versus infestation terminology

o Whether the 1748 parasites represent total count or mean counts per species

o Whether prevalence and intensity values are calculated per parasite taxon or per host species

Maybe you could write it something like this, please checj for accuracy: A total of 265 fish were examined, including Oreochromis niloticus (n = 135), Clarias gariepinus (n = 87), and Barbus intermedius (n = 43), with an overall infection/infestation rate of 88.3% (n = 234). In total, 1,748 parasites were collected and classified into nematodes, digenean trematodes, monogeneans, myxozoans, crustacean ectoparasites, and leeches. The most common nematodes were Contracaecum spp., particularly prevalent in C. gariepinus (50.57%). Digenean trematodes included Clinostomum, Euclinostomum, Diplostomum, and encysted metacercariae, with the latter reaching 68.97% in C. gariepinus. Monogenean infections with Dactylogyrus spp. occurred mainly in O. niloticus (28.15%). Myxobolus spp., leeches, Argulus, and Dolops species were also recorded across hosts. Mean parasite intensity was 8.59 in B. intermedius, 7.92 in C. gariepinus, and 6.79 in O. niloticus, while mean abundance was 7.40, 7.37, and 5.84, respectively.

Lines 99–116 and elsewhere:

Please ensure that “spp.” is not italicised throughout the manuscript. Only the genus and species names should appear in italics (e.g., Contracaecum spp.), while the abbreviation “spp.” always remains in regular font.

Lines 117 to 118:

please provide a reference

Lines 129–130:

Please clarify whether this statement refers specifically to the situation in Ethiopia. If it does not relate directly to Ethiopia or is not supported by local data, I recommend deleting the sentence to maintain accuracy and avoid overgeneralisation.

Lines 129–142:

You may also wish to consider the impacts of parasites on fish behaviour, as this is an increasingly recognised aspect of fish health and ecology. Behavioural alterations caused by parasitic infections can influence feeding, predator avoidance, reproduction, and overall survival, thereby affecting population dynamics and fisheries productivity. Including a brief acknowledgement of this dimension would enrich the Discussion and provide a more holistic interpretation of your findings. If relevant, you may consult recent literature on parasite-induced behavioural changes in fish for additional context. For example:

Animals 2024 Vol. 14 Issue 9 Pages 1315

Applied Animal Behaviour Science 2022 Pages 105722

Australian Freshwater Sciences Society. Waurn Ponds, 1-4 December. 2019

Behaviour 2012 Vol. 149 Issue 6 Pages 601-622

Int J Parasitol 1995 Vol. 25 Issue 12 Pages 1371-1383

J Exp Biol 2013 Vol. 216 Issue 1 Pages 18-26

L. Rogers, E. Sales, S. Shamsi, R. K. Kopf and R. Freire

Parasitol Res 2021 Vol. 120 Issue 2 Pages 515-523

Line 143:

I recommend replacing the term “developing country” with “low-income country” when referring to Ethiopia. The phrase “developing country” can be overly broad and does not reflect the many areas in which Ethiopia is already well-developed culturally, scientifically, and economically. Using “low-income country” provides clearer, more precise context and aligns with current international terminology.

Tables:

Please add horizontal lines and clearer structural formatting to the tables to improve readability. At present, the data are difficult to follow, and adding lines (for example, separating header rows from data rows and delineating major sections) will make the tables much easier for readers to interpret. Consistent alignment of columns and spacing will further enhance clarity.

Discussion

Please expand the Discussion to address the limitations of using visual inspection alone for parasite detection. While visual examination is acceptable and widely used in field-based parasitology studies, it has well-recognised limitations. Without applying digestion methods (see Canadian Journal of Fisheries and Aquatic Sciences 1986 Vol. 43 Issue 7 Pages 1312-1317) or incubation techniques (see International Journal of Food Microbiology 2016 Vol. 227 Pages 13-16), it is likely that some small or deeply encysted parasites, early developmental stages, or low-intensity infections were not detected. Acknowledging this limitation is important for transparency and will actually strengthen your study. It highlights that the parasite diversity and prevalence in Lake Hawassa may be even higher than reported, reinforcing the significance of your findings and the need for further, more sensitive diagnostic approaches in future research.

Discussion (lines 315-317, 329 to 331 and more):

Some parts of the Discussion section repeat information already presented in the Results. These repeated descriptions should be removed to avoid redundancy. The Discussion should focus on interpreting the findings, comparing them with previous studies, exploring implications, and acknowledging limitations. Please revise by deleting the sections that duplicate the Results, including lines XXX, and ensure the Discussion provides insight rather than repetition.

Lines 428–431:

Clinostomum is a particularly important parasite, and the Discussion would benefit from addressing aspects beyond simple prevalence comparisons. I encourage the authors to expand this section by briefly discussing:

• Key features of the life cycle (including the role of snails, fish, and piscivorous birds),

• Its pathogenic significance, especially its occurrence in the skull, musculature, and other critical tissues,

• Implications for fish health, fish welfare, and potential zoonotic considerations, and

• Any ecological or economic consequences relevant to Lake Hawassa or similar systems.

Including a short synthesis similar to discussions found in recent studies on Clinostomum—for example, research addressing tissue localisation, pathological effects, and host–parasite interactions—would enrich the manuscript and provide readers with a more comprehensive understanding of the importance of this genus. Some articles that authors may find useful to read are suggested below:

Aquaculture 2021 Vol. 539 Pages 736584 DOI: https://doi.org/10.1016/j.aquaculture.2021.736584

Journal of helminthology 2020 Vol. 94

Korean J Parasitol 2019 Vol. 57 Issue 2 Pages 175-177

Parasitology Research 2021 Vol. 120 Pages 2793-2803

Revue de Médecine Vétérinaire 2018 Vol. 7 Pages 147-151

Syst Parasitol 2018 Vol. 95 Issue 6 Pages 543-566

Line 445:

The co-occurrence of Clinostomum and Euclinostomum is an interesting and noteworthy finding. You may wish to highlight this more clearly, as such combinations can offer insights into shared intermediate hosts, environmental conditions, or ecological overlap. The authors may also find relevant comparisons in studies from India (Parasitology Research 2022 Vol. 121 Issue 11 Pages 3083-3089) where similar patterns have been reported. Including a brief mention of that work could help contextualise your findings and strengthen the Discussion.

Figures:

The figures require substantial improvement in multiple aspects. I strongly recommend that the authors revise them with guidance from a senior researcher experienced in parasitological imaging and figure preparation. At present, several issues affect clarity and scientific value:

• In some images, it is unclear what the arrows are pointing to. Please ensure arrows clearly indicate the diagnostic structures or lesions of interest.

• Some figures contain multiple images but lack labels (e.g., A, B, C) or accompanying explanations, making it impossible for the reader to understand what each panel represents.

• Scale bars and magnification details are missing or inconsistent.

• Image quality varies considerably, and some photographs are too dark, blurry, or low resolution for reliable interpretation.

Improving these elements will significantly enhance the scientific credibility of the manuscript. Clear, well-labelled, high-quality figures are essential for parasitology work, particularly when morphological identification is involved.

**********

what does this mean?). If published, this will include your full peer review and any attached files.

If you choose “no”, your identity will remain anonymous but your review may still be made public.

**Do you want your identity to be public for this peer review?**  For information about this choice, including consent withdrawal, please see our Privacy Policy

Reviewer #1: No

Reviewer #2: No

Reviewer #3: **Yes:** José Alberto Iannacone Oliver, Ph.D.

Reviewer #4: No

**********

[NOTE: If reviewer comments were submitted as an attachment file, they will be attached to this email and accessible via the submission site. Please log into your account, locate the manuscript record, and check for the action link "View Attachments". If this link does not appear, there are no attachment files.]

To ensure your figures meet our technical requirements, please review our figure guidelines: https://journals.plos.org/plosone/s/figures

You may also use PLOS’s free figure tool, NAAS, to help you prepare publication quality figures: https://journals.plos.org/plosone/s/figures#loc-tools-for-figure-preparation.

NAAS will assess whether your figures meet our technical requirements by comparing each figure against our figure specifications.

Attachment Submitted filename: PONE-D-25-59453.pdf

Attachment Submitted filename: New Review.docx

Attachment Submitted filename: PONE-D-25-59453.pdf

# Author comment: Author response to Decision Letter 1

**DOI:** 10.1371/journal.pone.0357816.r002

20 Mar 2026

We thank the editor in chief and the reviewers for their well appreciated comments and suggestion. We have addressed all the comments and provided separate the response letter to each reviewers

Attachment Submitted filename: Rebuttal letter_editors_reviewers comment.docx

# Peer review: Decision Letter 1

**DOI:** 10.1371/journal.pone.0357816.r003

**Authors:** Clement Yaro

8 Apr 2026

Dear Dr. EFA,

Thank you for submitting your manuscript to PLOS ONE. After careful consideration, we feel that it has merit but does not fully meet PLOS ONE’s publication criteria as it currently stands. Therefore, we invite you to submit a revised version of the manuscript that addresses the points raised during the review process.

Please submit your revised manuscript by May 23 2026 11:59PM. If you will need more time than this to complete your revisions, please reply to this message or contact the journal office at plosone@plos.org. When you're ready to submit your revision, log on to https://www.editorialmanager.com/pone/ and select the 'Submissions Needing Revision' folder to locate your manuscript file.

- A letter that responds to each point raised by the academic editor and reviewer(s). You should upload this letter as a separate file labeled 'Response to Reviewers'.
- A marked-up copy of your manuscript that highlights changes made to the original version. You should upload this as a separate file labeled 'Revised Manuscript with Track Changes'.
- An unmarked version of your revised paper without tracked changes. You should upload this as a separate file labeled 'Manuscript'.

If you would like to make changes to your financial disclosure, please include your updated statement in your cover letter. Guidelines for resubmitting your figure files are available below the reviewer comments at the end of this letter.

If applicable, we recommend that you deposit your laboratory protocols in protocols.io to enhance the reproducibility of your results. Protocols.io assigns your protocol its own identifier (DOI) so that it can be cited independently in the future. For instructions see: https://journals.plos.org/plosone/s/submission-guidelines#loc-laboratory-protocols. Additionally, PLOS ONE offers an option for publishing peer-reviewed Lab Protocol articles, which describe protocols hosted on protocols.io. Read more information on sharing protocols at https://plos.org/protocols?utm_medium=editorial-email&utm_source=authorletters&utm_campaign=protocols.

As the corresponding author, your ORCID iD is verified in the submission system and will appear in the published article. PLOS supports the use of ORCID, and we encourage all coauthors to register for an ORCID iD and use it as well. Please encourage your coauthors to verify their ORCID iD within the submission system before final acceptance, as unverified ORCID iDs will not appear in the published article. *Only*  the individual author can complete the verification step; PLOS staff *cannot*  verify ORCID iDs on behalf of authors.

We look forward to receiving your revised manuscript.

Kind regards,

Clement Ameh Yaro, Ph.D

Academic Editor

PLOS One

Journal Requirements:

If the reviewer comments include a recommendation to cite specific previously published works, please review and evaluate these publications to determine whether they are relevant and should be cited. There is no requirement to cite these works unless the editor has indicated otherwise.

[Note: HTML markup is below. Please do not edit.]

Reviewers' comments:

Reviewer’s Responses to Questions

**Comments to the Author**

Reviewer #1: All comments have been addressed

Reviewer #2: (No Response)

Reviewer #3: (No Response)

Reviewer #4: (No Response)

**********

2. Is the manuscript technically sound, and do the data support the conclusions?

Reviewer #1: Yes

Reviewer #2: No

Reviewer #3: Partly

Reviewer #4: No

**********

3. Has the statistical analysis been performed appropriately and rigorously?

Reviewer #1: Yes

Reviewer #2: No

Reviewer #3: No

Reviewer #4: I Don't Know

**********

4. Have the authors made all data underlying the findings in their manuscript fully available?

The PLOS Data policy

Reviewer #1: Yes

Reviewer #2: No

Reviewer #3: No

Reviewer #4: Yes

**********

5. Is the manuscript presented in an intelligible fashion and written in standard English?

Reviewer #1: Yes

Reviewer #2: No

Reviewer #3: Yes

Reviewer #4: No

**********

Reviewer #1: I would like to thank the authors for their response; the manuscript has been significantly improved by incorporating the suggested changes."

Reviewer #2: This manuscript reports on parasite prevalence, intensity, and abundance across three commercially important fish species from Lake Hawassa, Ethiopia. While the fieldwork addresses a genuine knowledge gap and the dataset has intrinsic value, the manuscript cannot be accepted or sent for major revision in its current form. The combination of critical data inconsistencies, invalid statistical calculations, and a serious research integrity concern collectively disqualify this submission. Rejection is recommended.

Causes of Rejection:

1. Fundamental Statistical Errors

The sub-group prevalence figures reported throughout Table 2 were calculated using the number of infected fish as the denominator rather than the total number of examined fish. This is a foundational error in prevalence calculation that inflates every sub-group estimate in the manuscript and renders the primary analytical results unreliable. For example, the C. gariepinus sub-analyses use n=81 as the denominator when the correct denominator is n=87. This error is systematic, not isolated, and cannot be corrected editorially, it requires complete recalculation from the raw dataset upward.

2. Internally Contradictory Numerical Results

-Mean intensity values of 5.37 and 8.08 are cited in the Results text (lines 284–285) but appear nowhere in Table 1, which reports 7.92 and 8.59 for the same species. No explanation is offered. The reader cannot determine which figures are correct.

-The chi-square P-value for species-level parasite variation is reported simultaneously as P=0.06 in the text (line 287) and P=0.24 in Table 1. These values lead to different scientific conclusions and cannot both be correct.

-The overall prevalence is reported as 88.30% throughout the manuscript but 88.03% in the conclusion (line 565).

-Sample sizes for B. intermedius and C. gariepinus are transposed in the Results section (line 277–278) relative to all other sections, creating fundamental confusion about the study population.

-These are not typographical errors. They reflect insufficient verification of results before submission and undermine confidence in all reported findings.

3. Serious Concern

Lines 603–606 contain the following statement:

"Parent/legal guardian provided informed consent on behalf of participants of age below eighteen years whereas oral consent was received from those above eighteen-year fishermen."

This is a human subjects informed consent statement in a manuscript reporting exclusively on fish parasitology. Its presence is unexplained and has no legitimate place in this study. This either indicates that sections of the manuscript were carelessly assembled from other documents; raising concerns about the originality and careful preparation of the methods section broadly, or that material from a separate human-subjects study was erroneously incorporated. Either explanation constitutes a serious lapse in manuscript integrity that the editorial office must address independently of the scientific review.

4. Biologically Implausible Finding Presented Without Justification

Table 3 reports Contracaecum species localized to the heart and pericardial cavity of O. niloticus. Cardiac localization of anisakid larvae in teleost fish is extraordinarily rare and, if genuine, would represent a significant and novel parasitological finding warranting dedicated documentation including histopathology, photographic evidence, and targeted discussion. The authors present this without any commentary, supporting evidence, or citation. This strongly suggests an anatomical misidentification or data recording error. Publishing an uncorroborated claim of this nature would mislead the scientific record.

5. Absence of Seasonal Analysis Despite a 10-Month Study

The study spans October 2023 to July 2024, covering both wet and dry seasons in the Ethiopian Rift Valley; periods known to differ substantially in water temperature, intermediate host abundance, and parasite transmission dynamics. No temporal or seasonal breakdown of parasite data is presented or discussed. This omission is not a limitation of the study design but of the analysis, and it represents a missed opportunity that significantly reduces the manuscript’s scientific contribution and generalizability.

6. Additional Concerns Noted for Authors' Consideration Upon Resubmission Elsewhere

-No confidence intervals are reported for any prevalence estimate

-Chi-square tests were applied to cells with expected frequencies below 5; Fisher’s exact test is required in these cases

-No multivariate analysis accounting for collinearity between host size variables

-The terms infection and infestation are used interchangeably throughout; these are technically distinct and should be applied correctly

-All microscopy figures lack scale bars — a mandatory requirement in parasitological publications

-The Limitations section appears twice with different content (lines 558–561 and 593–596) and must be consolidated

-A CNN travel article (lines 732–735) is cited as a scientific reference and must be replaced with peer-reviewed literature

-The Discussion is largely a comparison of prevalence percentages across studies without mechanistic explanation. The roles of intermediate host ecology, lake eutrophication, seasonal transmission dynamics, and One Health implications of zoonotic parasites in a population known to consume raw fish should be substantively developed in any future submission

Reviewer #3: All in the MS. Thoroughly review each part of the manuscript. There are many errors and incomplete sections in the introduction, methods, results, discussion, and conclusions.

Reviewer #4: Overall comment: While the manuscript addresses an important topic, the flow across sections, particularly within the Introduction and Discussion, would benefit from further refinement. At present, the narrative appears somewhat fragmented, with ideas not always presented in a clear and logical progression. To enhance readability and coherence, the authors are encouraged to introduce subheadings within the Introduction and Discussion. This would help organise key themes, guide the reader more effectively through the manuscript, and strengthen the overall structure and impact of the study.

Introduction: The Introduction would benefit from a clearer focus and stronger contextualisation. At present, it presents largely general and somewhat fragmented global information on fish parasites, without adequately grounding the study in the local context.

In particular, there is no synthesis of previously reported parasites from Lake Hawassa or, more broadly, Ethiopia. Including a concise overview of existing records in the study region would significantly strengthen the rationale and highlight the knowledge gap this study aims to address.

I recommend restructuring the Introduction to:

(i) reduce generalised global information,

(ii) incorporate relevant regional and national studies, and

(iii) clearly articulate what is already known versus what remains unknown in Lake Hawassa.

Lines 12–13: These statements are more appropriate for the Introduction rather than the Abstract. The Abstract should remain concise and focused on the study objectives, methods, key results, and conclusions. I recommend removing these lines to improve clarity and maintain the appropriate structure of the Abstract.

Line 17: The term “economic heartbeat” is somewhat informal for a scientific manuscript. Consider replacing it with more precise wording (e.g., “key economic resource” or “plays a central role in the local economy”) to maintain an academic tone.

Lines 19–20: The term “host factors” is too broad and should be specified. Please clarify which host-related variables were investigated (e.g., size, age, sex, condition factor, or other biological or ecological traits) to improve clarity and interpretability of the study design.

Lines 77–78: The sentence requires revision for clarity and grammatical accuracy. It may be rephrased as: “The Nilo-Sudanic fauna includes species represented by genera such as Alestes, Labeo, Bagrus, Citharinus, Hydrocynus, Hyperopisus and Protopterus.”

Line 85: The genus names Barbus and Garra should be italicised in accordance with standard scientific nomenclature conventions.

Lines 107–108: The reference Chai et al., 2005 appears outdated for supporting this statement. Please consider citing more recent literature to ensure the information reflects current knowledge in the field.

Lines 111–113: The statement that these are “the most common parasitic agents of fish” is misleading. The taxa listed (e.g., Opisthorchis, Anisakis, Diphyllobothrium) appear to be presented as examples rather than representative of the most common fish parasites globally. I recommend revising the wording to reflect that these are illustrative examples (e.g., “include” or “such as”) rather than definitive statements about prevalence. You could write as: ““Fish harbour a diverse range of parasitic taxa, including trematodes (flukes, e.g., Opisthorchis spp.), nematodes (roundworms, e.g., Anisakis spp.), and cestodes (tapeworms, e.g., Diphyllobothrium spp.).” A relevant reference to support this statement would be Fishes 2019 Vol. 4 Issue 1 Pages 9; doi:10.3390/fishes4010009.

Lines 120–124: must be deleted. This section contains overly generalised statements that are not universally supported. The relationship between host factors (e.g., sex, size) and parasitism is highly context-dependent and varies across host species, parasite taxa, and environmental conditions. For example, differences between males and females, or associations with body size, are not consistent across systems and should not be presented as general rules. I recommend revising this section to avoid overgeneralisation and to frame these factors as potential influences that may vary among studies. The statement regarding compromised immunity in females during the reproductive season also requires stronger justification and should be supported by appropriate, context-specific references or moderated accordingly.

Lines 128–130: This statement is inaccurate and overgeneralised. It is not correct to suggest that fish-borne parasitic larvae ingested by humans generally develop only into morphologically mature but sexually immature worms. The outcome depends on the parasite taxon. Some fish-borne parasites may remain larval in humans, whereas others can develop into adult stages and establish patent infections. I recommend revising this sentence to reflect the diversity of transmission pathways and host outcomes more accurately, and to avoid oversimplification of zoonotic potential. You could write “Larval stages of some fish-borne parasites, including certain nematodes and trematodes, may be transmitted to humans through the consumption of raw or undercooked fish, posing important zoonotic risks.” And you could reference articles such as Fishes 2019 Vol. 4 Issue 1 Pages 9; doi:10.3390/fishes4010009 and Veterinary Quarterly 2022 Vol. 42 Issue 1 Pages 95-118

Lines 131–135: The points raised are valid; however, the references used are somewhat dated. There is a substantial body of more recent literature addressing parasite transmission dynamics, host–parasite interactions, and the role of trophic ecology. I recommend incorporating more up-to-date references to better reflect current understanding in the field. Consider these or any thing more recent:

Parasitology 2024 Vol. 151 Issue 8 Pages 864-874

Canadian Journal of Zoology 2023; DOI: 10.1139/cjz-2022-0126

Scientific Reports 2023 Vol. 13 Issue 1 Pages 10291; https://doi.org/10.1038/s41598-023-37428-y

Line 146: the reference is outdated

Materials and methods:

Lines 174–182: The content in this section does not appear to describe methodological procedures and is more appropriate for the Introduction. I recommend relocating this text to the Introduction and ensuring that the Methods section is limited to study design, sampling, and analytical approaches.

Lines 184–189: The sample size calculation is clearly described; however, it appears that a collective sample size was applied across all fish species. Please explicitly specify the number of specimens examined for each fish species included in the study. This information is essential for interpreting species-specific prevalence, intensity, and comparative analyses.

Line 192: The term “different sampling points” is vague and requires clarification. Please specify the exact sampling locations (e.g., named sites, coordinates, or habitat types) within the study area. In addition, it is unclear how these sampling points were selected (e.g., random, stratified, based on accessibility or ecological characteristics). Clarifying the selection criteria is important for assessing potential sampling bias and ensuring reproducibility.

Lines 207–213: This interpretation is too strong based solely on body length data. Without ageing the fish, or providing species-specific evidence on maturity and developmental stage, it is not possible to conclude that shorter fish represent less mature individuals while larger fish represent fully mature adults with broader dietary niches. In addition, differences in parasite burden between size classes cannot be interpreted simply as a function of development or exposure, as smaller fish may also differ in condition, growth, or health status. I recommend moderating this section substantially and avoiding biological interpretations that are not directly supported by the data.

Line 215: Please specify the fish species examined and the number of individuals for each species. This information is essential for interpreting the results and for enabling species-specific comparisons and should be clearly presented, either in the text or as a summary table.

Lines 219–225: The euthanasia procedure is now clearly described and appreciated. The methods used appear appropriate for rapid loss of consciousness. For future studies, the authors may also consider the use of anaesthetic agents (e.g., clove oil or other approved fish anaesthetics), which are commonly recommended in many guidelines to further minimise stress and handling-related impacts prior to euthanasia.

Lines 239–250: The use of the pepsin–HCl artificial digestion method is appropriate and well described, and it is commendable that the authors have applied a recognised technique for detecting encysted larvae in muscle tissue. However, it appears that this method (or equivalent digestion/incubation approaches see International Journal of Food Microbiology 2016 Vol. 227 Pages 13-16 and Canadian Journal of Fisheries and Aquatic Sciences 1986 Vol. 43 Issue 7 Pages 1312-1317) was not applied to other organs, particularly the gastrointestinal tract, where many parasites are commonly located and are deeply embedded in the tissue. This may have limited the detection of a broader range of parasites and potentially led to an underestimation of parasite diversity and burden. I recommend that the authors acknowledge this as a limitation of the study and briefly discuss how it may have influenced their findings particularly for parasites that preferentially inhabit non-muscle tissues.”

Lines 253–255: This statement appears misplaced, as it follows the section on parasite identification and does not directly contribute to that context. The justification for muscle digestion is methodological and would be more appropriate within the earlier description of the digestion procedure. I recommend either relocating this sentence to the Methods section where the technique is first introduced or removing it to improve the logical flow and coherence of the manuscript.

Lines 260–267: The reference cited for these parasitological parameters appears to be incorrect. The standard reference is Bush et al. (1997). Please revise accordingly and ensure consistent citation of this foundational work. Bush AO, Lafferty KD, Lotz JM, Shostak AW. Parasitology meets ecology on its own terms: Margolis et al revisited. J Parasitol. 1997 Aug;83(4):575-83.

Lines 277–280: The description of sampled fish species and size ranges is clear; however, it is important to note whether fish age was determined. If age data were not collected, this should be explicitly stated in the Methods. In addition, I recommend acknowledging in the Discussion that the absence of age determination limits interpretation of parasite–host relationships, particularly where size is used as a proxy for developmental stage or exposure.

Results and Table 1: The presentation of prevalence across fish species is clear; however, the authors should interpret these values with caution. As digestion/incubation methods (see International Journal of Food Microbiology 2016 Vol. 227 Pages 13-16 and Canadian Journal of Fisheries and Aquatic Sciences 1986 Vol. 43 Issue 7 Pages 1312-1317) were not applied to all organs (particularly the gastrointestinal tract), it is likely that some parasites were not detected. This may have led to an underestimation of the true parasite diversity and prevalence. I recommend that the authors acknowledge this limitation in the Results (briefly) and more explicitly in the Discussion, noting that the reported prevalence values likely represent conservative estimates.

Table 2: The analysis relating parasitic infestation to sex, body weight, and length is informative; however, these results should be interpreted with caution in the absence of age determination. Body size alone is not a reliable proxy for age, maturity, or cumulative exposure to parasites. In addition, parasite biomass itself may confound weight-based interpretations. Heavily infected fish may appear heavier not because they are in better condition, but due to the physical mass of the parasites they harbour. For example, studies on clupeids from the Caspian Sea (Parasitology Research 2018 Vol. 17 Issue 10 Pages 3327-3331) have shown that 3-year-old fish infected with pathogenic Corynosoma spp. appeared heavier than uninfected fish of the same age class, with the increased weight attributable to parasite load rather than improved host condition. This article also might be very useful: Int J Parasitol 2020 Vol. 50 Issue 10 Pages 755-761. I recommend that the authors acknowledge these limitations and avoid overinterpreting associations between parasite burden and host size metrics. Incorporating this consideration into the Discussion would strengthen the biological interpretation of the findings.

Table 4: Myxobolus species are NOT protozoa. Dactylogyrus species are not Trematode either!

Lines 467–475: This is a useful comparison with previous studies, and the discussion on variation in prevalence across water bodies is appreciated. To further strengthen this section, the authors may consider briefly linking these findings to the ecological and pathological significance of these parasites in definitive hosts. In particular, digenean flukes such as Clinostomum and Euclinostomum spp. are known to infect fish-eating birds as definitive hosts, where they can have important health and ecological implications. Including a short statement on their pathogenicity and role in avian hosts would provide a more complete picture of the parasite’s life cycle and broader significance.

**********

what does this mean?). If published, this will include your full peer review and any attached files.

If you choose “no”, your identity will remain anonymous but your review may still be made public.

**Do you want your identity to be public for this peer review?**  For information about this choice, including consent withdrawal, please see our Privacy Policy

Reviewer #1: **Yes:** Olfat Anter Mahdy

Reviewer #2: No

Reviewer #3: **Yes:** José Iannacone

Reviewer #4: No

**********

[NOTE: If reviewer comments were submitted as an attachment file, they will be attached to this email and accessible via the submission site. Please log into your account, locate the manuscript record, and check for the action link "View Attachments". If this link does not appear, there are no attachment files.]

To ensure your figures meet our technical requirements, please review our figure guidelines: https://journals.plos.org/plosone/s/figures

You may also use PLOS’s free figure tool, NAAS, to help you prepare publication quality figures: https://journals.plos.org/plosone/s/figures#loc-tools-for-figure-preparation.

NAAS will assess whether your figures meet our technical requirements by comparing each figure against our figure specifications.

Attachment Submitted filename: PONE-D-25-59453_R1.pdf

# Author comment: Author response to Decision Letter 2

**DOI:** 10.1371/journal.pone.0357816.r004

1 Jun 2026

We have addressed all editor and reviewers comments and our manuscript is totally improved from earlier versions

Attachment Submitted filename: Response to reviewers_May27.docx

# Peer review: Decision Letter 2

**DOI:** 10.1371/journal.pone.0357816.r005

**Authors:** Clement Yaro

28 Jun 2026

Dear Dr. EFA,

Thank you for submitting your manuscript to PLOS ONE. After careful consideration, we feel that it has merit but does not fully meet PLOS ONE’s publication criteria as it currently stands. Therefore, we invite you to submit a revised version of the manuscript that addresses the points raised during the review process.

Please submit your revised manuscript by Aug 12 2026 11:59PM. If you will need more time than this to complete your revisions, please reply to this message or contact the journal office at plosone@plos.org. When you're ready to submit your revision, log on to https://www.editorialmanager.com/pone/ and select the 'Submissions Needing Revision' folder to locate your manuscript file.

- A letter that responds to each point raised by the academic editor and reviewer(s). You should upload this letter as a separate file labeled 'Response to Reviewers'.
- A marked-up copy of your manuscript that highlights changes made to the original version. You should upload this as a separate file labeled 'Revised Manuscript with Track Changes'.
- An unmarked version of your revised paper without tracked changes. You should upload this as a separate file labeled 'Manuscript'.

If you would like to make changes to your financial disclosure, please include your updated statement in your cover letter. Guidelines for resubmitting your figure files are available below the reviewer comments at the end of this letter.

If applicable, we recommend that you deposit your laboratory protocols in protocols.io to enhance the reproducibility of your results. Protocols.io assigns your protocol its own identifier (DOI) so that it can be cited independently in the future. For instructions see: https://journals.plos.org/plosone/s/submission-guidelines#loc-laboratory-protocols. Additionally, PLOS ONE offers an option for publishing peer-reviewed Lab Protocol articles, which describe protocols hosted on protocols.io. Read more information on sharing protocols at https://plos.org/protocols?utm_medium=editorial-email&utm_source=authorletters&utm_campaign=protocols.

As the corresponding author, your ORCID iD is verified in the submission system and will appear in the published article. PLOS supports the use of ORCID, and we encourage all coauthors to register for an ORCID iD and use it as well. Please encourage your coauthors to verify their ORCID iD within the submission system before final acceptance, as unverified ORCID iDs will not appear in the published article. *Only*  the individual author can complete the verification step; PLOS staff *cannot*  verify ORCID iDs on behalf of authors.

We look forward to receiving your revised manuscript.

Kind regards,

Clement Ameh Yaro, Ph.D

Academic Editor

PLOS One

**Journal Requirements:**

If the reviewer comments include a recommendation to cite specific previously published works, please review and evaluate these publications to determine whether they are relevant and should be cited. There is no requirement to cite these works unless the editor has indicated otherwise.

[Note: HTML markup is below. Please do not edit.]

Reviewers' comments:

Reviewer’s Responses to Questions

**Comments to the Author**

Reviewer #2: All comments have been addressed

Reviewer #4: (No Response)

**********

2. Is the manuscript technically sound, and do the data support the conclusions?

Reviewer #2: Yes

Reviewer #4: Partly

**********

3. Has the statistical analysis been performed appropriately and rigorously?

Reviewer #2: Yes

Reviewer #4: Yes

**********

4. Have the authors made all data underlying the findings in their manuscript fully available?

The PLOS Data policy

Reviewer #2: Yes

Reviewer #4: No

**********

5. Is the manuscript presented in an intelligible fashion and written in standard English?

Reviewer #2: Yes

Reviewer #4: No

**********

**Reviewer #2:**  -Nomenclature consistency: "B. intermedius" and "L. intermedius" are used interchangeably throughout — pick one (suggest Labeobarbus intermedius, noting "also known as B. intermedius" on first use) and apply consistently.

-Table 2, C. gariepinus Total Length: p=0.0492 is statistically significant, but the text (line 295) states the distribution "showed not statistically significant association" for all variables. Either flag/discuss this one significant result briefly in Results/Discussion, or double-check the value.

-Line 41 typo: "was to be 8.59, 7.92 and 6.79" → "was found to be 8.59, 7.92 and 6.79".

-Line 98: Parasitic species can be found everywhere and on every living organism. need citation: https://www.nature.com/articles/s41598-026-46340-0

Line 121-122: Fish is a good host for parasite multiplication that can be zoonotic to humans or other animals that feed fishes. needs updated citation: https://www.tandfonline.com/doi/abs/10.1080/10454438.2026.2662934

-Line 272–273: Overall length range "10cm to 80cm" — worth confirming this matches the per-species length classes in Table 2 (max categories are ">25cm"/">26cm"/">70cm"), since 80cm seems outside those bins.

-Figure 1 caption: "Barbus species, the middle" — specify full species name (B. intermedius) for consistency with the rest of the text.

-Table 4 header: "Parasite class" column — "Hurindae" should be "Hirudinea" (correct spelling).

-Line 320 (Table 3 reference): "Contacaecum" → "Contracaecum" (typo, appears twice in that paragraph).

-Welfare/spelling: "welbeing" (abstract, line 21) → "wellbeing".

-Consistent terminology: "infection/infestation" used interchangeably per reviewer’s earlier note — a brief definitional sentence early in Methods clarifying usage would help, though this is optional polish.

**Reviewer #4:** Lines 29–33: The term "overall infection/infestation rate" is ambiguous and should be replaced with a clearly defined parasitological parameter. If the authors are referring to the proportion of fish harbouring at least one parasite species, the term overall prevalence would be more appropriate. The authors should also clearly state how the value of 88.30% was calculated and whether it represents infection with any parasite taxon or a specific subset of parasites. Consistent use of standard parasitological terminology (e.g., prevalence, mean intensity, mean abundance) throughout the manuscript is recommended. The manuscript would benefit from following the recommendations of Bush et al. regarding standard ecological and parasitological terminology, particularly for prevalence, intensity, and abundance measures.

Introduction (first 3 paragraphs) and elsewhere: The manuscript opens with a discussion of the importance of aquaculture; however, the study was conducted in a natural lake ecosystem rather than an aquaculture setting. As currently written, the relevance of these introductory paragraphs to the study is unclear. If the lake is used for aquaculture, stocking programmes, or other aquaculture-related activities, this should be clearly explained and linked to the study objectives. If not, I recommend revising the introduction to focus more directly on the ecological, fisheries, conservation, or public health significance of parasite infections in wild fish populations within the study system. This would provide a stronger rationale and improve the overall flow of the manuscript.

Introduction : The Introduction would benefit from a concise review of the parasites previously reported from the host species. At present, I could not readily determine what is already known about the parasite fauna of this fish, whether similar studies have been conducted elsewhere, and what specific knowledge gap this study addresses. Including a brief summary of previous records would help establish the novelty and significance of the present work.

Materials and Methods : Throughout the Materials and Methods section, parasites are referred to by their final taxonomic identities (e.g. Diplostomum spp. and other identified taxa). This information represents a study result and should therefore be presented in the Results section, together with the morphological and/or molecular evidence supporting those identifications. In the Materials and Methods, it would be more appropriate to use broader terms such as "digenean trematodes", "nematodes", "cestodes", "helminths", or "parasite specimens" and then describe the identification procedures used. The specific parasite identities should be introduced only after the supporting evidence has been presented in the Results.

Line 245: citation “El-245 Shahawy et al., 2017)” should be deleted

Parasite identification and presentation of results: A major concern is that the Results section provides little evidence supporting the identification of the reported parasite taxa. While parasites are assigned to genera such as Diplostomum, Clinostomum, Euclinostomum, Contracaecum, Myxobolus, and others, the manuscript does not adequately describe the diagnostic features used to reach these identifications. Readers are therefore unable to independently assess the reliability of the taxonomic determinations.

In addition, the quality and presentation of the figures require substantial improvement. The images are of insufficient quality to demonstrate the key morphological characters used for identification. Many diagnostic structures are either not visible or not clearly indicated. Furthermore, the figures lack essential elements expected in taxonomic and parasitological studies, including scale bars and clear annotations highlighting important morphological features. As currently presented, the images do not provide adequate support for the parasite identifications reported in the manuscript.

The authors should either provide a more detailed account of the diagnostic criteria used for identification, supported by appropriate references, or include higher-quality photomicrographs showing the relevant taxonomic characters, with scale bars and annotations where appropriate. Without such evidence, it is difficult to assess the accuracy of the reported parasite identifications.

**********

what does this mean?). If published, this will include your full peer review and any attached files.

If you choose “no”, your identity will remain anonymous but your review may still be made public.

**Do you want your identity to be public for this peer review?**  For information about this choice, including consent withdrawal, please see our Privacy Policy

Reviewer #2: No

Reviewer #4: No

**********

[NOTE: If reviewer comments were submitted as an attachment file, they will be attached to this email and accessible via the submission site. Please log into your account, locate the manuscript record, and check for the action link "View Attachments". If this link does not appear, there are no attachment files.]

To ensure your figures meet our technical requirements, please review our figure guidelines: https://journals.plos.org/plosone/s/figures

You may also use PLOS’s free figure tool, NAAS, to help you prepare publication quality figures: https://journals.plos.org/plosone/s/figures#loc-tools-for-figure-preparation.

NAAS will assess whether your figures meet our technical requirements by comparing each figure against our figure specifications.

# Author comment: Author response to Decision Letter 3

**DOI:** 10.1371/journal.pone.0357816.r006

14 Aug 2026

We have carefully addressed all the comments and suggestions raised by each of the reviewers and the Editor-in-Chief. We greatly appreciate the opportunity to work with PLOS ONE and value the constructive feedback provided throughout the review process. This has helped us further improve the quality and clarity of our manuscript.

Attachment Submitted filename: Authors response_August 6.docx

# Peer review: Decision Letter 3

**DOI:** 10.1371/journal.pone.0357816.r007

**Authors:** Clement Yaro

23 Aug 2026

Prevalence of fish parasites from Lake Hawassa, Ethiopia

PONE-D-25-59453R3

Dear Dr. EFA,

We’re pleased to inform you that your manuscript has been judged scientifically suitable for publication and will be formally accepted for publication once it meets all outstanding technical requirements.

Within one week, you’ll receive an e-mail detailing the required amendments. When these have been addressed, you’ll receive a formal acceptance letter and your manuscript will be scheduled for publication.

An invoice will be generated when your article is formally accepted. Please note, if your institution has a publishing partnership with PLOS and your article meets the relevant criteria, all or part of your publication costs will be covered. Please make sure your user information is up-to-date by logging into Editorial Manager at Editorial Manager® and clicking the ‘Update My Information' link at the top of the page. For questions related to billing, please contact billing support.

If your institution or institutions have a press office, please notify them about your upcoming paper to help maximize its impact. If they’ll be preparing press materials, please inform our press team as soon as possible -- no later than 48 hours after receiving the formal acceptance. Your manuscript will remain under strict press embargo until 2 pm Eastern Time on the date of publication. For more information, please contact onepress@plos.org.

Kind regards,

Clement Ameh Yaro, Ph.D

Academic Editor

PLOS One

Additional Editor Comments (optional):

Reviewers' comments:

Reviewer’s Responses to Questions

**Comments to the Author**

Reviewer #2: All comments have been addressed

**********

2. Is the manuscript technically sound, and do the data support the conclusions?

Reviewer #2: Yes

**********

3. Has the statistical analysis been performed appropriately and rigorously?

Reviewer #2: Yes

**********

4. Have the authors made all data underlying the findings in their manuscript fully available?

The PLOS Data policy

Reviewer #2: Yes

**********

5. Is the manuscript presented in an intelligible fashion and written in standard English?

Reviewer #2: Yes

**********

Reviewer #2: All comments have been addressed adequately by authors, accordingly I accept it for possible publication

**********

what does this mean?). If published, this will include your full peer review and any attached files.

If you choose “no”, your identity will remain anonymous but your review may still be made public.

**Do you want your identity to be public for this peer review?**  For information about this choice, including consent withdrawal, please see our Privacy Policy

Reviewer #2: No

**********

# Editor report: Acceptance letter

**DOI:** 10.1371/journal.pone.0357816.r008

**Authors:** Clement Yaro

PONE-D-25-59453R3

PLOS One

Dear Dr. EFA,

I'm pleased to inform you that your manuscript has been deemed suitable for publication in PLOS One. Congratulations! Your manuscript is now being handed over to our production team.

At this stage, our production department will prepare your paper for publication. This includes ensuring the following:

* All references, tables, and figures are properly cited

* All relevant supporting information is included in the manuscript submission,

* There are no issues that prevent the paper from being properly typeset

You will receive further instructions from the production team, including instructions on how to review your proof when it is ready. Please keep in mind that we are working through a large volume of accepted articles, so please give us a few days to review your paper and let you know the next and final steps.

Lastly, if your institution or institutions have a press office, please let them know about your upcoming paper now to help maximize its impact. If they'll be preparing press materials, please inform our press team within the next 48 hours. Your manuscript will remain under strict press embargo until 2 pm Eastern Time on the date of publication. For more information, please contact onepress@plos.org.

You will receive an invoice from PLOS for your publication fee after your manuscript has reached the completed accept phase. If you receive an email requesting payment before acceptance or for any other service, this may be a phishing scheme. Learn how to identify phishing emails and protect your accounts at https://explore.plos.org/phishing.

If we can help with anything else, please email us at customercare@plos.org.

Thank you for submitting your work to PLOS One and supporting open access.

Kind regards,

PLOS One Editorial Office Staff

on behalf of

Dr. Clement Ameh Yaro

Academic Editor

PLOS One
